# Bioactive Diterpenes, Norditerpenes, and Sesquiterpenes from a Formosan Soft Coral *Cespitularia* sp.

**DOI:** 10.3390/ph14121252

**Published:** 2021-12-01

**Authors:** You-Cheng Lin, Chi-Chien Lin, Yi-Chia Chu, Chung-Wei Fu, Jyh-Horng Sheu

**Affiliations:** 1 Doctoral Degree Program in Marine Biotechnology, National Sun Yat-sen University, Kaohsiung 804, Taiwan; d045620002@nsysu.edu.tw; 2 Institute of Biomedical Science, National Chung-Hsing University, Taichung 402, Taiwan; lincc@dragon.nchu.edu.tw (C.-C.L.); girl770409@smail.nchu.edu.tw (Y.-C.C.); 3 Department of Marine Biotechnology and Resources, National Sun Yat-sen University, Kaohsiung 804, Taiwan; m095020006@nsysu.edu.tw; 4 Department of Medical Research, China Medical University Hospital, China Medical University, Taichung 404, Taiwan; 5 Graduate Institute of Natural Products, Kaohsiung Medical University, Kaohsiung 807, Taiwan; 6 Frontier Center for Ocean Science and Technology, National Sun Yat-sen University, Kaohsiung 804, Taiwan

**Keywords:** verticillane, *Cespitularia*, anti-inflammatory activity

## Abstract

Chemical investigation of the soft coral *Cespitularia* sp. led to the discovery of twelve new verticillane-type diterpenes and norditerpenes: cespitulins H–O (**1**–**8**), one cyclic diterpenoidal amide cespitulactam L (**9**), norditerpenes cespitulin P (**10**), cespitulins Q and R (**11** and **12**), four new sesquiterpenes: cespilins A–C (**13**–**15**) and cespitulolide (**16**), along with twelve known metabolites. The structures of these metabolites were established by extensive spectroscopic analyses, including 2D NMR experiments. Anti-inflammatory effects of the isolated compounds were studied by evaluating the suppression of pro-inflammatory protein tumor necrosis factor-α (TNF-α) and nitric oxide (NO) overproduction, and the inhibition of the gene expression of inducible nitric oxide synthase (iNOS) and cyclooxygenase-2 (COX-2), in lipopolysaccharide-induced dendritic cells. A number of these metabolites were found to exhibit promising anti-inflammatory activities.

## 1. Introduction

In the inflammatory stimuli, the inflammatory mediators such as tumor necrosis factor-α (TNF-α), prostaglandin E2 (PGE2), and nitric oxide (NO) are known to be secreted through lipopolysaccharide (LPS)-induced activation of macrophages [1,2,3] and dendritic cells [4,5,6,7]. Furthermore, the overexpression of two inducible proteins, inducible nitric oxide synthase (iNOS) and cyclooxygenase-2 (COX-2) produced the excess amount of NO and PGE2 in the inflammatory process. It has been well known that natural products have a great potential in drug discovery, thus the anti-inflammatory activity screening by evaluating the suppression of TNF-α and NO overproduction, and the inhibition of iNOS and COX-2 protein and gene expression, in LPS-induced macrophages or dendritic cells (DCs) is one of the important methods for searching for anti-inflammatory agents from natural compounds [8,9,10,11,12,13,14].

**Figure 1:**
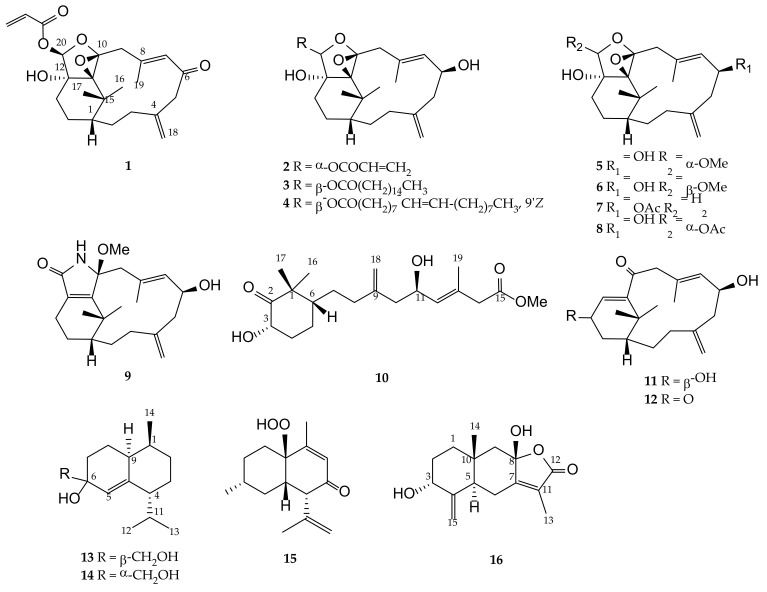
Structures of new compounds **1**–**16**.

**Figure 2:**
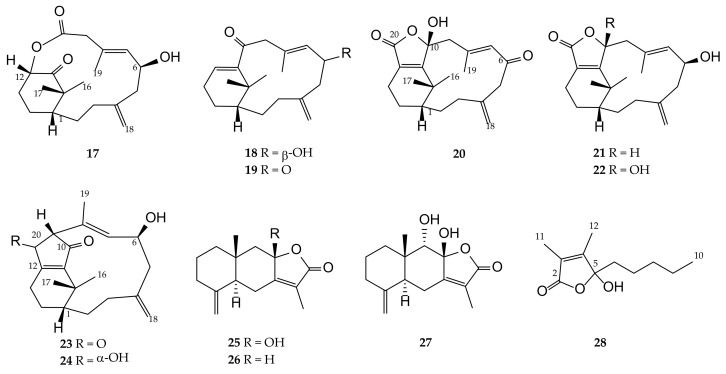
Structures of known compounds **17**–**28**.

Soft corals, in particular, those belonging to the genus *Cespitularia* (family Xeniidae), have afforded a series of verticillane-type diterpenes and some eudesmane-type sesquiterpenoids [15]. Secondary metabolites obtained from these soft corals have been shown to exhibit interesting biological activities, including cytotoxic [16,17,18,19,20,21,22,23,24,25], anti-inflammatory [25,26,27], antimicrobial [22], and antiviral [28] activities. Following the above findings, and with the aim of discovering bioactive compounds from marine invertebrates for further biomedical studies, we carried out the chemical investigation of the EtOAc extract of a Formosan soft coral *Cespitularia* sp. to search the bioactive principles, as preliminary bioassay showed that this crude extract exhibited significant activity to suppress the release of TNF-α and NO, and inhibited the upregulation of pro-inflammatory iNOS and COX-2 gene in lipopolysaccharide (LPS)-induced DCs. This study has led to the isolation of twenty-eight compounds, including eight new verticillane-type diterpenes cespitulins H–O (**1**–**8**), one new cyclic verticillane-type diterpenoidal amide cespitulactam L (**9**), three new verticillane-type norditerpenes cespitulins P–R (**10**–**12**), three new cadinane-type sesquiterpenes cespilins A–C (**13**–**15**), and one new eudesmane-type sesquiterpenoid cespitulolide (**16**) (Figure 1), along with twelve known compounds, cespitularin Q (**17**) [18,26], cespitularin E (**18**) [17], cespihypotin D (**19**) [29], cespihypotin F (**20**) [28], cespitularin O (**21**) [18], cespitularin D (**22**) [17,20,21], cespitularin I (**23**) [18,26], cespitularin F (**24**) [17,20,21,22,26], atractylenolide III (**25**) [30,31,32,33], atractylenolide II (**26**) [31,33], atractylenolide V (**27**) [32], and 5-hydroxy-3,4-dimethyl-5-pentylfuran-2(5*H*)-one (hydroxydihydrobovolide) (**28**) [34] (Figure 2). The structures of the compounds were elucidated on the basis of extensive spectroscopic analyses (IR, MS, 1D, and 2D NMR) and by comparison of the spectroscopic data with those of related known compounds.

Additionally, in order to discover bioactive substances for future medicinal application, the anti-inflammatory activities of the inhibition of TNF-α and NO, and the suppression of iNOS and COX-2 gene expression in LPS-induced DCs of the isolated compounds **1**–**28** were also evaluated and are reported herein.

## 2. Results

### 2.1. Structure Elucidation of the Verticillane-Type Diterpenes ***1**–**9***

From the previously published reports [15,16,17,18,19,20,21,22,26,27,28,29], it was found that there is no unambiguous evidence for determining the absolute configuration of the verticillane-type compounds.

Cespitulin H (**1**) was isolated as a white amorphous powder and its molecular formula was established as C_23_H_30_O_6_ by HRESIMS (*m/z* 425.1932 [M + Na]^+^), accounting for nine degrees of unsaturation. The IR spectrum of **1** exhibited the absorption peaks of hydroxy (3480 cm^–1^) and carbonyl (1741 cm^–^^1^) groups. Assignment of two germinal methyls (*δ*_C_ 26.8 and 24.9, both CH_3_; *δ*_H_ 1.35 and 0.72, both s), a methyl (*δ*_C_ 19.2, CH_3_; *δ*_H_ 2.11, s), a vinyl group (*δ*_C_ 132.6, CH_2_ and 127.6, CH; *δ*_H_ 6.15, br d, *J* = 17.5 Hz, 5.16, dd, *J* = 10.5, 1.0 Hz and 5.77, dd, *J* = 17.5, 10.5 Hz), a 1,1-disubstituted double bond (*δ*_C_ 144.1, C and 116.5, CH_2_; *δ*_H_ 5.09 and 4.77, both s), a trisubstituted double bond (*δ*_C_ 148.5, C and 129.4, CH; *δ*_H_ 6.13, s), an acetal (*δ*_C_ 101.8, CH; *δ*_H_ 5.96, s), three other *sp*^3^ oxygenated carbons (*δ*_C_ 94.6, 80.0, and 72.8, C), a conjugated ester carbonyl (*δ*_C_ 164.9, C), and a conjugated ketone (*δ*_C_ 197.7, C) of verticillane-type diterpene were supported by analysis of the ^13^C and ^1^H NMR signals along with heteronuclear single quantum coherence (HSQC) spectrum (Table 1 and Table 2).

**Figure 3:**
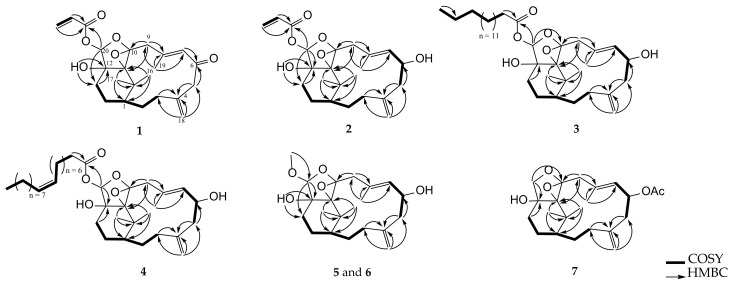
Selected COSY and HMBC correlations of **1**–**7**.

The planar structure of **1** was further determined by analysis of correlations spectroscopy (COSY) and heteronuclear multiple bond correlation (HMBC) correlations (Figure 3). The HMBC correlations of H_2_-9 (*δ*_H_ 3.00 and 2.21, both d, *J* = 16.0 Hz) to C-10 (*δ*_C_ 94.6, C) and C-11 (*δ*_C_ 72.8, C), assigned a possible 10,11-tetrasubstituted epoxide moiety. Additionally, HMBC correlations of a hydroxy proton (*δ*_H_ 2.25, br s) to both C-12 (*δ*_C_ 80.0, C) and C-13 (*δ*_C_ 26.8, CH_2_), as well as an acetal proton H-20 (*δ*_H_ 5.96, s) to both C-12 and ester carbonyl carbon (*δ*_C_ 164.9, C, C-21), positioned a hydroxy group at C-12 and an acrylate group at C-20. The above findings and the remaining one degree of unsaturation were used to establish a polyoxygenated epoxytetrahydrofuran ring, as shown in formula of **1**.

**Figure 4:**
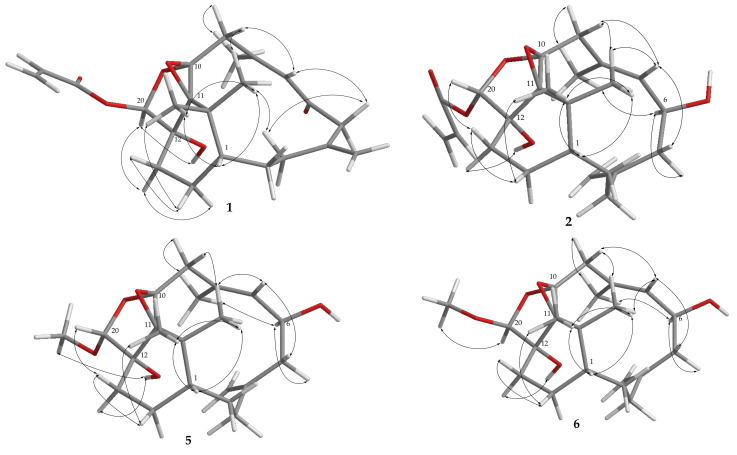
Selected NOE correlations of compounds **1**, **2**, **5**, and **6**.

The relative stereochemistry of **1** was determined by the analysis of nuclear Overhauser effect spectroscopy (NOESY) correlations and molecular modeling from energy-minimized (MM2) force field calculation. Assuming the β-orientation of H-1 (*δ*_H_ 1.19, m), the NOE correlations of H-1 with both H_3_-16 (*δ*_H_ 0.72, s) and H_3_-17 (*δ*_H_ 1.35, s) indicted the upward orientation of both H_3_-16 and H_3_-17. One proton of H_2_-14 (*δ*_H_ 2.16, m) exhibited NOE correlations with one proton of H_2_-13 (*δ*_H_ 1.46, m) and H_3_-17; thus the above methylene protons were characterized as H-14β and H-13β, while the rest protons were assigned as H-14α (*δ*_H_ 1.11, ddd, *J* = 14.0, 6.0, 3.5 Hz) and H-13α (*δ*_H_ 1.57, td, *J* = 14.0, 3.5 Hz). Subsequently, H-20 (*δ*_H_ 5.96, s) exhibited NOE interactions with both H-13α and 12-OH (*δ*_H_ 2.25, br s), revealing that H-20 and the 12-hydroxy group were positioned on the α face. Moreover, H-7 (*δ*_H_ 6.13, br s) exhibited an NOE response with one proton of H-9 (*δ*_H_ 2.21, d, *J* = 16.0 Hz), while H_3_-19 (*δ*_H_ 2.11, s) showed an NOE interaction with the other proton of H-9 (*δ*_H_ 3.00, d, *J* = 16.0 Hz) but not with H-7, confirming the *E* geometry of trisubstituted double bond at C-7/C-8. The above NOE results were shown to be well matched with a molecular model of minimized energy generated from MM2 calculation in Figure 4. Additionally, conformational searching of compound **1** by molecular mechanics model with MMFF force field calculation in the Spartan’14 program [35] was further performed. In a relative energy window of 0−3 Kcal/mol, the results of the calculation displayed nine lowest energy conformers for **1** (Table S2 and Figure S113) which were shown to fit from the observed NOE correlations. From the above findings, the relative configuration of **1** was elucidated as that for formula **1**.

Cespitulin I (**2**) appeared as a white amorphous powder with the molecular formula C_23_H_32_O_6_ as indicated by the HRESIMS (*m/z* 427.2089 [M + Na]^+^) spectrum, suggesting the presence of eight degrees of unsaturation. The IR spectrum showed the absorptions of hydroxy (3440 cm^–1^) and carbonyl (1740 cm^–1^) groups. The NMR data of **2** (Table 1 and Table 2) revealed this compound to be a tricyclic verticillane-type diterpene and should be very similar to cespihypotin H [28] except for the position of a tetrasubstituted epoxide and the hydroxy group in the tetrahydrosubstituted furan ring. Similar to **1**, this tetrasubstituted epoxide was located between C-10 (*δ*_C_ 94.7, C) and C-11 (*δ*_C_ 72.4, C), and the hydroxy group was found at C-12 (*δ*_C_ 79.8, C) on the basis of the assistance of HMBC correlations. The position of the acrylate group at C-20 was also confirmed by the HMBC correlations from H-20 (*δ*_H_ 5.70, s) and H-22 (*δ*_H_ 6.14, dd, *J* = 17.2, 10.4 Hz) to C-21 (*δ*_C_ 164.8, C). These observations, together with analysis of other COSY and HMBC correlations, enabled the gross structure of **2** to be established reasonably (Figure 3).

The relative configurations of the six chiral centers at C-1, C-6, C-10, C-11, C-12, and C-20 in **2** were also determined from key NOE correlations with an MM2 force field calculation (Figure 4). One proton of H_2_-9 (*δ*_H_ 2.55, d, *J* = 14.4 Hz) showed NOE interaction with the known β-oriented H_3_-16 (*δ*_H_ 0.94, s) and suggested as H-9β, while the other proton at C-9 was assigned as H-9α (*δ*_H_ 3.10, d, *J* = 14.4 Hz). Similar to **1**, the *E* geometry of 7,8-trisubstituted double bond was confirmed, as the NOE correlations of H-9β with H-7 and H-9α with H_3_-19 (*δ*_H_ 1.81, s) were found and also from the observation of an upfield chemical shift of C-19 at 17.2 ppm [9]. Moreover, H-6 (*δ*_H_ 4.49, t, *J* = 8.0 Hz) displayed NOE correlations with one proton of H_2_-5 (*δ*_H_ 2.65, dd, *J* = 12.5, 3.2 Hz) and H_3_-19, while H-7 was found to correlate with the other proton of H_2_-5 (*δ*_H_ 2.24, m), reflecting the β-orientation of hydroxy group at C-6. Furthermore, the NOE correlation observed between the β-oriented H_3_-17 and one proton of H_2_-14 (*δ*_H_ 2.28, m), which also correlated with one proton of H_2_-13 (*δ*_H_ 1.71, td, *J* = 14.0, 3.6 Hz), suggested the *β*-orientation of these two methylene protons at C-14 and C-13, respectively. H-13β exhibited an NOE correlation with H-20 (*δ*_H_ 5.70, s), while 12-OH (*δ*_H_ 2.64, br s) correlated with H-13α (*δ*_H_ 1.58, m) but not with H-20, revealing that the acrylate group at C-20 was α-oriented. By conformational searching for **2** using MMFF molecular mechanics model, 13 lowest conformers (Table S2 and Figure S114) of **2** were found and also could explain the observed NOE correlations. From these results and other detailed NOE correlations, the relative configuration of **2** was determined.

Cespitulin J (**3**) was isolated as a colorless oil. The HRESIMS (*m/z* 611.4282 [M + Na]^+^) and NMR data (Table 1 and Table 2) of **3** exhibited a molecular formula of C_36_H_60_O_6_, acquiring seven degrees of unsaturation. The IR spectrum suggested the presence of hydroxy (3446 cm^–1^) and ester carbonyl (1758 cm^–1^) groups. Comparison of the NMR spectroscopic data of **3** and **2** indicated that the structure of **3** was highly similar to that of **2**, with the exception of an acrylate ester group in **2** being replaced by a long-chain ester moiety in **3**. Furthermore, it is reasonable to elucidate the hexadecanoyl ester group at C-20 (*δ*_C_ 100.8, CH) by HRESIMS and 2D NMR spectroscopic data, including HMBC and COSY correlations. Thus, the structural framework of **3** was established to be a verticillane-type diterpene, including a polyoxygenated epoxytetrahydrofuran ring, too (Figure 3). The analysis of the NOESY spectrum revealed that **3** possessed the same relative configurations at C-1, C-6, C-10, C-11, and C-12 as those of compound **2**. A difference in the stereochemistry of H-20 between **2** and **3** was demonstrated with the assistance of the NOESY experiment which revealed that H-20 (*δ*_H_ 5.63, s) had an NOESY correlation with 12-OH (*δ*_H_ 2.52, br s), indicating that H-20 of **3** should be α-oriented and accomplished the elucidation of the relative configuration of **3**.

Cespitulin K (**4**) was obtained as a colorless oi1 that gave a sodiated adduct ion peak at *m*/*z* 637.4440 [M + Na]^+^ in the HRESIMS spectrum, suggesting the molecular formula C_38_H_62_O_6_ with eight degrees of unsaturation. IR absorptions at 3420 and 1748 cm^–1^ showed the presence of hydroxy and ester carbonyl functionalities, too. The ^13^C and ^1^H NMR spectroscopic data (Table 1 and Table 2) of **4** were found to be very similar to those of **3**, with the exception that the hexadecanoyl ester at C-20 in **3** was converted to the octadecenoyl ester group in **4** by the HRESIMS data and 2D NMR (HMBC and COSY) correlations (Figure 3) of **3**. The remaining one degree of unsaturation has arisen from the *cis* C-9′/C-10′ double bond of the octadecenoyl ester group in **4** by comparison of ^13^C NMR spectroscopic data of this ester side chain at C-20 with those reported previously [36,37]. Finally, the *Z* geometry of the 9′, 10′-double bond was also deduced from a 10.5 Hz coupling constant between H-9′ and H-10′ in the ^1^H NMR spectrum.

The relative configuration of **4** was also determined by a NOESY experiment. The NOE correlations of H-1(*δ*_H_ 1.49, m), H_2_-5 (*δ*_H_ 2.66, dd, *J* = 12.5, 2.5 Hz and 2.25, m), H-6 (*δ*_H_ 4.50, m), H-7 (*δ*_H_ 5.47, d, *J* = 8.0 Hz), H_2_-9 (*δ*_H_ 3.09 and 2.54, both d, *J* = 14.0 Hz), H_3_-16 (*δ*_H_ 0.98, s), H_3_-17 (*δ*_H_ 1.33, s), H_3_-19 (*δ*_H_ 1.81, s), H-20 (*δ*_H_ 5.63, s), and 12-OH (*δ*_H_ 2.53, br s) were almost the same as those of **3**, suggesting the same configurations at the corresponding carbons in both **3** and **4**. On the basis of the above results, the relative configuration of **4** was established.

Cespitulin L (**5**) was isolated as a white amorphous powder. Its molecular formula, C_21_H_32_O_5_, was established by HRESIMS (*m/z* 365.2315 [M + H]^+^), implying six degrees of unsaturation. The IR spectrum showed the presence of the hydroxy moiety (3445 cm^–1^). The ^13^C and ^1^H NMR spectroscopic data revealed that **5** was found to possess a 10,20-ether linkage tetrahydrosufuran ring (*δ*_C_ 104.6, CH, C-20 and 94.3, C, C-10; *δ*_H_ 4.36, s, H-20) and a 10,11-tetrasubstituted epoxide (*δ*_C_ 72.8, C, C-11), as well as the same verticillane core skeleton of compounds **2**–**4** (Table 1 and Table 2). The presence of a methoxy group at C-20 of **5** was further established by an HMBC correlation from H_3_-21 (*δ*_H_ 3.47, s) to C-20 (Figure 3). These results suggested that the relative configuration of **5** was nearly the same as those of **2**–**4**. Further, the 20-acetal proton (*δ*_H_ 4.36, s) was found to show an NOE interaction with H-13β (*δ*_H_ 1.70, br d, *J* = 14.0 Hz), while the 12-OH (*δ*_H_ 3.29, s) exhibited interactions with both H-13α (*δ*_H_ 1.58, m) and H_3_-21, indicating the β-orientation of H-20 and the α-orientation of the 21-methoxy group (Figure 4).

Cespitulin M (**6**) was found to possess the same molecular formula, C_21_H_32_O_5_, as that of **5** from the HRESIMS data (387.2142 [M + Na]^+^). Analysis of the 1D NMR spectroscopic data (Table 1 and Table 3) and the 2D NMR (HSQC, COSY, and HMBC) correlations enabled the planar structure of **6** to be established the same as **5** (Figure 3). The ^13^C NMR spectroscopic data of **6** were nearly similar to those of **5**, with the exception of downfield shifts observed at C-12 (∆*δ*_C_ +1.6) and C-20 (∆*δ*_C_ +4.5) relative to **5**, revealing that **6** should be the C-12 or C-20 isomer of **5**. Further analysis of NOE correlations revealed that **6** possessed the identical relative configurations at C-1, C-6, C-10, C-11, and C-12 as those of **5**. A difference in relative configuration for C-20 of the tetrahydrofuran ring between **5** and **6** was characterized by a comparison of their key NOE correlations (Figure 4).

Cespitulin N (**7**) had the molecular formula C_22_H_32_O_5_ as determined by HRESIMS (*m/z* 399.2142 [M + Na]^+^). The IR spectrum of **7** showed the presence of hydroxy (3446 cm^–1^) and ester carbonyl (1733 cm^–1^) groups. All the proton and carbon signals of **7** were assigned from the ^13^C and ^1^H NMR spectroscopic data (Table 3 and Table 4), along with HSQC spectrum, which established the structure of **7** as a tetracyclic verticillane-type diterpene with an acetoxy group (*δ*_C_ 170.2, C and 21.3, CH_3_; *δ*_H_ 2.02, s). In addition, the NMR data of **7** were found to resemble those of cespitulin G [27]. Detailed analysis of 2D NMR spectra (COSY, HMBC, and NOESY), revealed that **7** possesses a 10,20-ether linkage trihydrosubstituted furan ring (*δ*_C_ 95.9, C, C-10 and 75.5, CH_2_, C-20; *δ*_H_ 3.59 and 3.43, both d, *J* = 9.0 Hz), a 10,11-epoxide (*δ*_C_ 74.0, C, C-11), and a hydroxy group at C-12 (*δ*_C_ 78.7, C; *δ*_H_ 1.96, br d, *J* = 2.0 Hz, 12-OH). Furthermore, key NOE correlations of the 12-OH to H-13α (*δ*_H_ 1.76, m) and H-20α (*δ*_H_ 3.43, d, *J* = 9.0 Hz) indicated that the 12-hydroxy group should be positioned on the α face.

**Figure 5:**
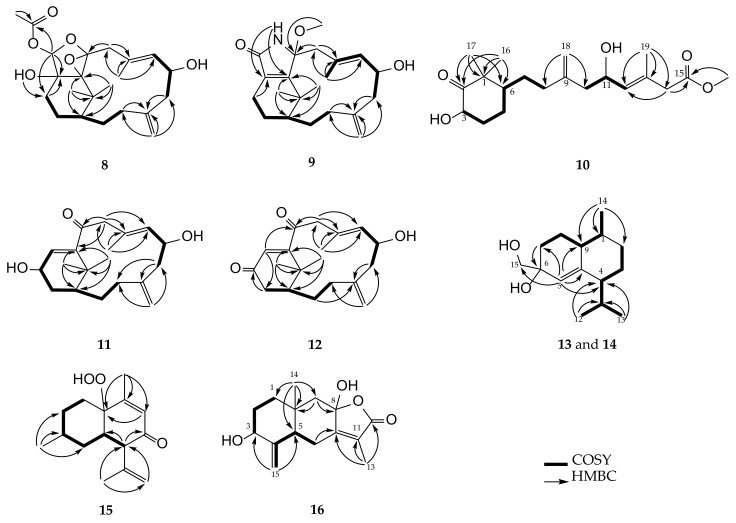
Selected COSY and HMBC correlations of **8**–**16**.

**Figure 6:**
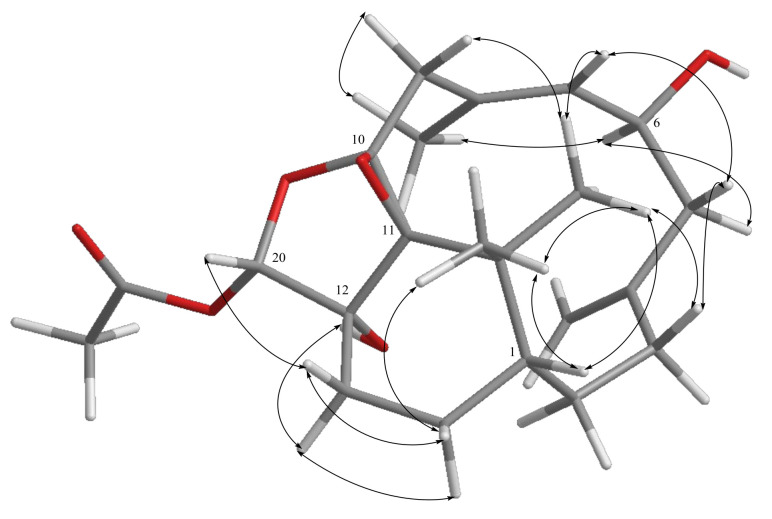
Selected NOE correlations of compound **8**.

The protonated adduct ion peak [M + H]^+^ of cespitulin O (**8**) at *m/z* 393.2267 in HRESIMS indicated a molecular formula C_22_H_32_O_6_. The IR absorptions showed the presence of the hydroxy (3419 cm^–1^) and an ester carbonyl (1733 cm^–1^) groups. The ^13^C and ^1^H spectroscopic data (Table 3 and Table 4) of **8** were very similar to those of cespihypotin I [28] and had the same molecular formula. However, the gross structure of **8** was established as a 10,11-epoxy-10,11,12,20-tetrahydrosubstituted-furanyl diterpene containing an acetoxy group at C-20 by the results of 2D NMR experiments (including COSY and HMBC correlations, Figure 5). Further analysis of the NOE correlations revealed that H-20 (*δ*_H_ 5.76, s) showed NOE interaction with H-13β (*δ*_H_ 1.81, m), while 12-OH (*δ*_H_ 2.68, br s) with H-13α (*δ*_H_ 1.62, m), confirming the β-orientation of the acetoxy group at C-20 (Figure 6).

**Figure 7:**
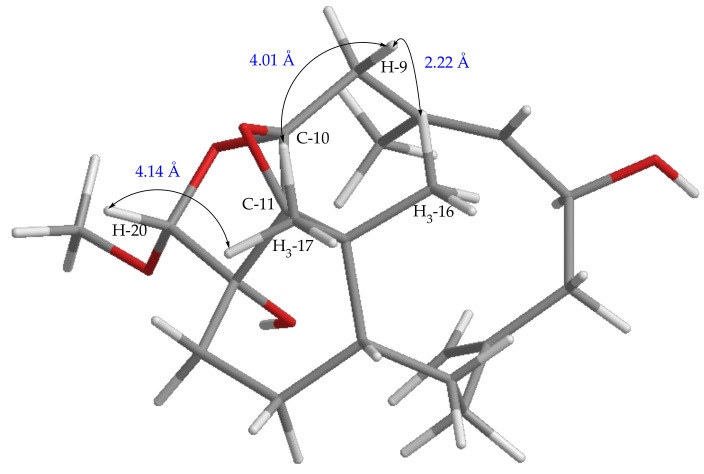
The distance H_3_-16/H-9, H_3_-17/H-9, and H_3_-17/H-20 of the relative configuration of C-10 and C-11 in compound **5**.

**Figure 8:**
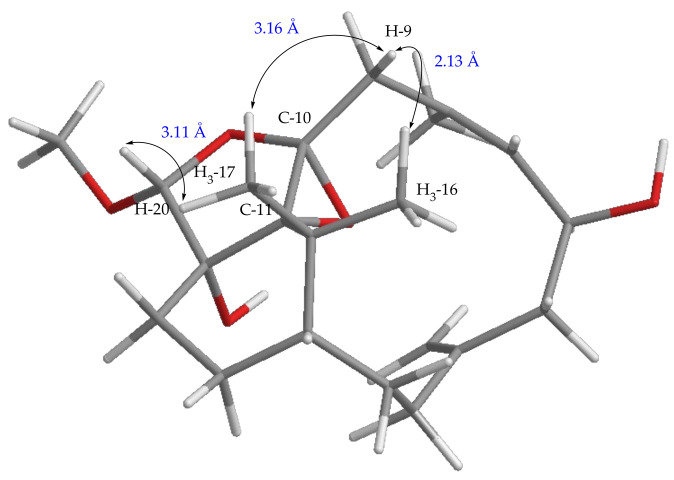
The distance H_3_-16/H-9, H_3_-17/H-9, and H_3_-17/H-20 of the relative configuration of C-10 and C-11 in isomeric compound **5**.

The assignment of the relative stereochemistry of the nonprotonated carbons C-10 and C-11 of the epoxy ring in compounds **1**–**8** was also based on the observed NOE correlations and molecular model calculation. For example, the relative configurations of C-10 and C-11 of compound **5** as shown in Figure 7 were assigned on the basis that the distance between H_3_-16 and one proton of H_2_-9 is 2.22 Å and that between H_3_-17 and this proton of H_2_-9 is 4.01 Å in the molecular model generated from MM2 calculation, which well match the NOE correlation observed between H_3_-16 and this proton of H_2_-9, and not fit the correlations between H_3_-17 and the same proton at C-9. Moreover, for the conformer of the isomeric 10,11-epoxide (Figure 8), the NOE correlations for H_3_-17/H-20, and between both H_3_-16 and H_3_-17 with this H-9 should be found as the distances of H_3_-17/H-20, H_3_-16, and H_3_-17 with this H-9 proton were calculated to be 3.11, 2.13, and 3.16 Å, respectively. However, only the NOE correlation between H_3_-16 and this specific H-9 was detected, suggesting the relative configuration of **5** and the other related compounds should be the same as those described in Figure 1.

**Figure 9:**
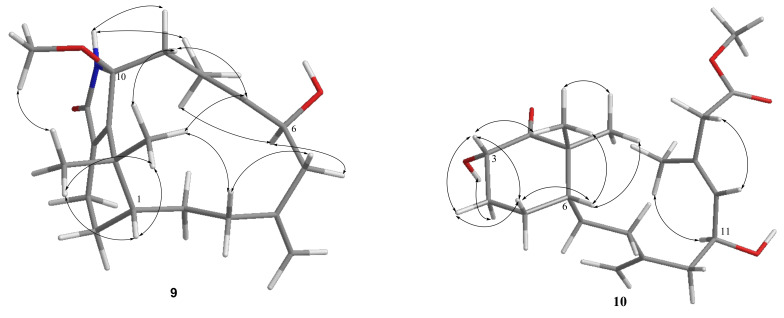
Selected NOE correlations of compounds **9** and **10**.

The HRESIMS data (*m/z* 368.2195 [M + Na]^+^) of cespitulactam L (**9**) established the molecular formula C_21_H_31_O_3_N, consistent with seven degrees of unsaturation. The IR spectrum suggested the presence of hydroxy and/or amide (3245 cm^–^^1^) and conjugated carbonyl (1698 cm^–^^1^) groups. Compound **9** and cespitulactam F [22] were found to have the same α,β-unsaturated lactam ring by comparison of their 1D and 2D NMR spectroscopic data. Likewise, the ^1^H and ^13^C NMR data of **9** (Table 3 and Table 4) were highly similar with those of cespitulactam F, with the difference that the presence of a methoxy group (*δ*_C_ 50.4, CH_3_; *δ*_H_ 3.13, s) at C-10 (*δ*_C_ 93.9, C) in **9** was found, instead of a hydroxy group in cespitulactam F. Cespitulactam L (**9**) is the 10-methoxy derivative of cespitulactam F. The relative stereochemistry of **9** was deduced from the analysis of the observed NOE correlations. The known β-oriented H_3_-17 (*δ*_H_ 1.24, s) exhibited NOE interactions with the methoxy protons (*δ*_H_ 3.13, s), indicating the β-orientation of 10-methoxy group. By the biogenetic consideration and other detailed NOE correlations (Figure 9), cespitulactam L (**9**) was found to possess the same relative configuration as that of cespitulactam F.

### 2.2. Structure Elucidation of a Novel Norditerpene ***10*** and the Verticillane-Type Norditerpenes ***11*** and ***12***

Compound **10** exhibited a sodiated ion peak at *m*/*z* 375.2141 [M + Na]^+^ in the HRESIMS, establishing a molecular formula C_20_H_32_O_5_ and implying five degrees of unsaturation. The presence of the hydroxy, ester carbonyl, and ketone groups was observed by IR absorptions at 3445, 1732, and 1715 cm^–1^, respectively. The ^13^C and ^1^H NMR data (Table 5) of **10** revealed that three degrees of unsaturation were contributed from a 1,1-disubstituted double bond (*δ*_C_ 147.1, C and 113.2, CH_2_; *δ*_H_ 4.87, s), a trisubstituted double bond (*δ*_C_ 131.7, CH and 122.2, C; *δ*_H_ 5.32, d, *J* = 8.5 Hz), and an ester carbonyl (*δ*_C_ 171.4, C). The remaining two degrees of unsaturation were arisen from a 2-hydroxy-6,6-dimethylcyclohexan-1-one moiety by inspection of 2D NMR correlations (Figure 5). The NMR spectroscopic data of **10** resemble those of known norditerpenoid cespitularin Q (**17**) [18,26], except for the presence of a methoxy group (*δ*_C_ 51.8, CH_3_; *δ*_H_ 3.69, s) at C-15 (*δ*_C_ 171.4, C) and a hydroxy group at C-3 (*δ*_C_ 71.4, CH) in **10**, and also the absence of a 14-membered lactone ring linkage between C-10 (*δ*_C_ 169.7, C) and C-12 (*δ*_C_ 72.2, CH) which is present in **17**, indicating that a linear terpenoidal ester **10** might be arisen from cespitularin Q by hydrolysis and further esterification. It was also found that the molecular skeleton of **10** is nearly the same as that of retinoids with missing of the methyl group at C-5, while normal retinoids are originated from the oxidative cleavage of β-carotene [38]. In the NOESY spectrum, a strong interaction between H-6β (*δ*_H_ 1.79, m) and H_3_-17 (*δ*_H_ 1.32, s) showed the β-orientation of H_3_-17. Further, H-3 (*δ*_H_ 4.45, m) showed NOE correlations with both H_3_-17 and one proton of H-4 (*δ*_H_ 2.31, m), as did the 3-OH (*δ*_H_ 3.67, br d, *J* = 3.5 Hz) with the other proton of H-4 (*δ*_H_ 1.58, m), reflecting that H-3 should be β-oriented while the hydroxy group at C-3 was assigned as α-oriented (Figure 9). By the analysis of the above NOE correlations and the biosynthetic relation of **10** and **17**, the relative configuration of **10** was elucidated and named cespitulin P.

The new norditerpene cespitulin Q (**11**) was obtained as a colorless oil, which showed the pseudomolecular ion peak [M + H]^+^ at *m*/*z* 305.2108 in HRESIMS, appropriate for the molecular formula of C_19_H_28_O_3_ and six degrees of unsaturation. The IR absorptions at 3418 and 1699 cm^–1^ indicated the presence of the hydroxy and carbonyl groups, respectively. The carbon NMR signals (Table 4) at *δ*_C_ 202.6 (C), 150.3 (C), and 134.6 (CH), as well as the proton NMR signal (Table 3) at *δ*_H_ 6.10 (d, *J* = 3.5 Hz), were characteristic resonances for an α,β-unsaturated ketone unit in **11**.

**Figure 10:**
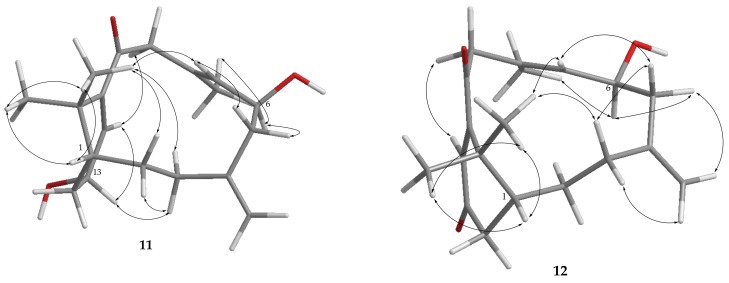
Selected NOE correlations of compounds **11** and **12**.

The analyses of COSY and HMBC correlations were used to establish the planar structure of **11** (Figure 5). Moreover, the NMR spectroscopic data of **11** were found to close to those of known metabolite cespitularin E (**18**) [17], with the exception of the carbon signal of C-13 resonating at *δ*_C_ 23.9 (CH_2_) in **18** was downfield shifted to *δ*_C_ 65.9 (CH) in **11**, suggesting that **11** is the C-13 oxidation derivative of cespitularin E (**18**). From the NOE correlations (Figure 10) of **11**, one of the methylene protons at C-3 (*δ*_H_ 2.59, dd, *J* = 15.0, 11.0 Hz) displayed an NOE correlation with the β-oriented H_3_-16 (*δ*_H_ 1.09, s), which correlated with the known β-oriented H-1 (*δ*_H_ 1.81, m), and thus was characterized as H-3β, while the other (*δ*_H_ 1.86, m) was assigned as H-3α. The NOE correlation between H-13 (*δ*_H_ 4.50, m) and H-3α determined the β-orientation of the hydroxy group at C-13. From the all NOE correlations observed, the relative configuration of **11** was thus established.

The molecular formula of cespitulin R (**12**) was found to be C_19_H_26_O_3_, as deduced by HRESIMS (*m*/*z* 325.1777 [M + Na]^+^). IR absorptions at 3420 and 1748 cm^–1^ of the corresponding hydroxy and carbonyl moieties were also confirmed. Comparison of the ^1^H and ^13^C NMR spectroscopic data (Table 3 and Table 4) of **11** and **12** suggested that both compounds are the same bicyclic verticillane-type norditerpenes, except that a hydroxy group at C-13 (*δ*_C_ 65.9, CH; *δ*_H_ 4.50, m) in **11** was replaced by a ketone (*δ*_C_ 199.0, C) in **12**. The planar structure of **12** was further determined from analysis of the HMBC and COSY correlations, as shown in Figure 5. From the above results and on the basis of the analysis of NOE correlations (Figure 10), the relative configuration of cespitulin R (**12**) was established.

### 2.3. Structure Elucidation of the Cadinane-Type Sesquiterpenes ***13**–**15*** and the Eudesmane-Type Sesquiterpenoid ***16***

The molecular formula C_15_H_26_O_2_ of cespilin A (**13**) was revealed from the HRESIMS spectrum (*m*/*z* 261.1824 [M + Na]^+^). The IR spectrum of **13** showed the presence of the hydroxy group at 3392 cm^–1^. The ^13^C NMR (Table 6) and ^1^H NMR (Table 7), with the assistance of HSQC spectra, showed signals of three methyls, five methylenes (including one oxymethylene), five methines, and two nonprotonated carbons. The gross structure of **13** was determined by the analysis of COSY and HMBC correlations (Figure 5). The cadinane skeleton of **13**, including placement of a hydroxy group and a hydroxymethyl group at C-6 (*δ*_C_ 71.2, C), was established mainly by the HMBC correlations from H_3_-14 (*δ*_H_ 0.84, d, *J* = 6.8 Hz) to C-1 (*δ*_C_ 34.7, CH), C-2 (*δ*_C_ 29.1, CH_2_), and C-9 (*δ*_C_ 36.6, CH); isopropyl methyls (*δ*_H_ 0.92 and 0.78, both d, *J* = 6.8 Hz) to C-4 (*δ*_C_ 51.1, CH); olefinic proton H-5 (*δ*_H_ 5.45, s) to C-4, C-7 (*δ*_C_ 31.1, CH_2_), C-9, C-10 (*δ*_C_ 147.0, C), and C-15 (*δ*_C_ 68.9, CH_2_), and H_2_-7 (*δ*_H_ 1.83, m, and 1.46, d, *J* = 9.2 Hz) to C-6.

**Figure 11:**
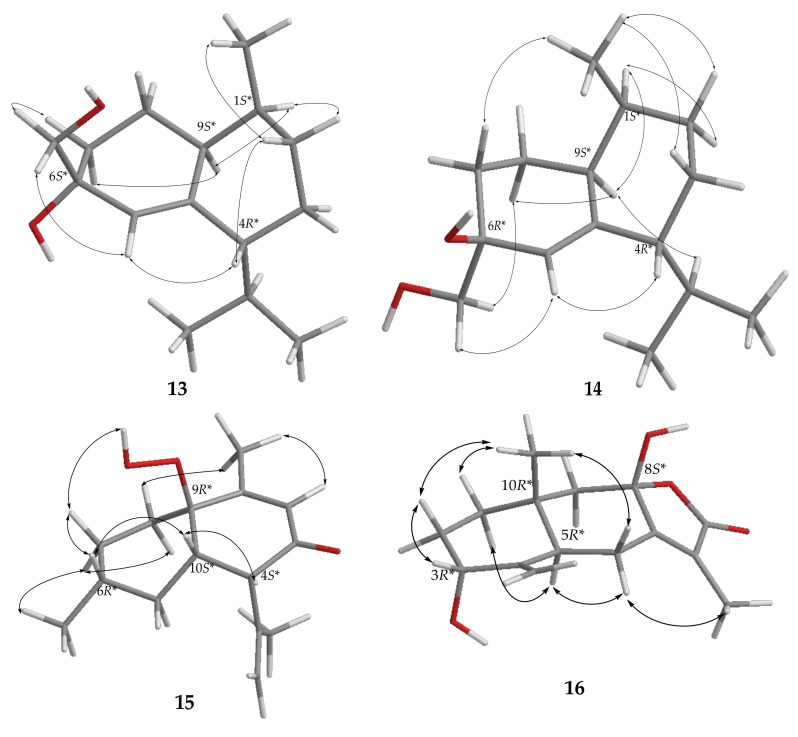
Selected NOE correlations of compounds **13**–**16**.

The relative stereochemistry of **13** was examined mainly with the assistance of an NOE experiment. It was found that H_3_-14 (*δ*_H_ 0.84, d, *J* = 6.8 Hz) showed an NOE correlation with one proton of H_2_-2 (*δ*_H_ 1.31, br t, *J* = 10.8 Hz), which further correlated with H-4 (*δ*_H_ 1.63, m); therefore, assuming the β-orientation of H_3_-14, the above methylene proton and H-4 should also be positioned on the β face, while H-1 (*δ*_H_ 1.96, m), the other proton of H_2_-2 (*δ*_H_ 1.82, m), and isopropyl group at C-4 were positioned on the α face. Furthermore, H-1 exhibited NOE correlations with both H-2α and H-9 (*δ*_H_ 2.31, br d, *J* = 4.4 Hz), revealing the α-orientation of H-9. Subsequently, H_2_-15 (*δ*_H_ 3.54 and 3.46, both dd, *J* = 8.4, 4.8 Hz) showed an NOE correlation with one proton of H_2_-7 (*δ*_H_ 1.46, d, *J* = 9.2 Hz), while the α-oriented H-9 which further correlated with another proton of H-7 (*δ*_H_ 1.83, m), suggesting that the 6-hydroxymethyl group should be placed on the β face, and in contrast, 6-hydroxy group should be positioned on the α face. Consequently, the relative configuration of **13** was elucidated as 1S*,4R*,6S*, and 9S* (Figure 11).

The HRESIMS data of cespilin B (**14**) (*m*/*z* 261.1824 [M + Na]^+^) established a molecular formula of C_15_H_26_O_2_, the same as that of **13**. Analysis of 2D NMR spectroscopic data, including HSQC, COSY, and HMBC, revealed that **14** should possess the same molecular skeleton as that of **13** (Figure 5). Additionally, the NMR data (Table 6 and Table 7) of **14** were highly similar in all aspects to those of **13**, implying that **14** is a structurally similar isomer of **13**. Comparison of the NOE correlations of both **13** and **14** revealed that both compounds possess the same 1S*, 4R*, and 9S* relative configurations. From the NOE correlations of the α-oriented H-9 (*δ*_H_ 2.24, td, *J* = 13.2, 4.8 Hz) with H-8α (*δ*_H_ 1.64, m), as well as H_2_-15 (*δ*_H_ 3.49 and 3.43, both d, *J* = 10.8 Hz) further correlated with H-8α, the 6-hydroxymethyl group should be placed on the α face, while the hydroxy group at C-6 should be β-oriented. Compound **14** was thus found to be the C-6 epimer of **13**, and the relative configuration was assigned to be 1S*, 4R*, 6R*, and 9S* (Figure 11).

Cespilin C (**15**) has the molecular formula C_15_H_22_O_3_ as shown by HRESIMS spectrum (*m*/*z* 273.1464 [M + Na]^+^). The IR spectrum of **15** showed the absorption of an α,β-unsaturated ketone (1683 cm^–1^) which was further characterized from the corresponding ^13^C NMR signals (Table 6) of *δ*_C_ 197.7 (C), 162.7 (C), and 128.6 (CH). The NMR signals at *δ*_C_ 84.0 (C) and *δ*_H_ 7.42 (1H, br s) revealed the presence of a hydroperoxy group at the sp^3^ nonprotonated carbon. Analysis of the COSY spectrum of **15** identified one proton sequence from H-4 to H_2_-8 via H-10, which assembled the major part of the planar structure of **15** with the crucial HMBC correlations as shown in Figure 5.

However, the connection of C-8/C-9 and C-9/C-10 could not be observed by COSY and HMBC correlations; instead, the two single bonds were established to fulfill the cadinane skeleton of **15** by the molecular formula and the tetrahedron nature of sp^3^ carbons. The relative structure of **15** was elucidated by the analysis of NOE correlations (Figure 11). Assuming the β-orientation of H-4 (*δ*_H_ 3.26, d, *J* = 13.5 Hz), NOE correlations of H-4 with H-10 (*δ*_H_ 2.90, br d, *J* = 13.5 Hz) and H-10 with H-6 (*δ*_H_ 1.61, m) implied the β-orientation of both H-6 and H-10, while the α-orientation of the isopropenyl group at C-4 and the methyl group (*δ*_H_ 0.90, d, *J* = 6.5 Hz) at C-6. Subsequently, one proton of H_2_-7 (*δ*_H_ 1.54, m) displayed NOE correlations with both the hydroperoxy proton (*δ*_H_ 7.42, s) and H-6, while the other proton of H_2_-7 (*δ*_H_ 1.22, m) correlated with H_3_-15, reflecting that the 9-hydroperoxy group should be situated on the β-face. Finally, the relative stereochemistry of **15** was thus established as 4S*, 6R*, 9R*, and 10S*.

Compound **16** was isolated as a white amorphous powder. The HRESIMS of **16** exhibited a sodiated pseudomolecular ion peak at *m*/*z* 287.1255 [M + Na]^+^ and revealed a molecular formula of C_15_H_20_O_4_, implying six degrees of unsaturation. The IR absorptions displayed the presence of the hydroxy (3418 cm^–1^) and carbonyl (1732 cm^–1^) groups. A comparison of the NMR data (Table 6 and Table 7) of **16** to those of a known metabolite atractylenolide III (**25**) [30,31,32,33], could well describe the molecular framework of **16** as eudesmane-type sesquiterpenoid. A difference was found that the methylene (H_2_-3) of **25** was substituted with a hydroxy group in **16** (Figure 1 and Figure 5).

The relative configuration of **16** was established by NOESY experiments and NMR spectroscopic data. H_3_-14 (*δ*_H_ 1.03, s) showed an NOE interaction with one proton of H_2_-1 (*δ*_H_ 1.38, m), while H-5 (*δ*_H_ 2.45, br s) displayed an NOE correlation with the other proton of H_2_-1 (*δ*_H_ 1.71, td, *J* = 13.8, 4.2 Hz), therefore, H-5 was suggested to be α-oriented as H_3_-14 was well known β-oriented for the eudesmane-type sesquiterpenoids [15,32]. In addition, NOE correlations were observed for one proton of H_2_-2 (*δ*_H_ 1.87, dt, *J* = 14.4, 3.6 Hz) with both H-3 (*δ*_H_ 4.37, br s) and H_3_-14, could reflect the α-orientation of the hydroxy group at C-3. Additionally, the ^13^C NMR signals of C-7 (*δ*_C_ 159.4, C), C-8 (*δ*_C_ 103.1, C), and C-9 (*δ*_C_ 51.0, CH_2_) in **16** were found to be similar to those of atractylenolide III (**25**) (*δ*_C_ 160.6, C, C-7; 103.2, C, C-8; 51.3, CH_2_, C-9), while in 8-epi-atractylenolide III C-7 (*δ*_C_ 157.7) and C-9 (*δ*_C_ 47.7) were shifted upfield and C-8 (*δ*_C_ 109.1) was shifted downfield, suggesting that the hydroxy group at C-8 in **16** should be positioned on the β face [33]. On the basis of the above analyses, and the other shown in Figure 11, the structure of **16** was thus elucidated to be (3R*,5R*,8S*,10R*)-3α,8β-dihydroxy-eudesma-4(15),7(11)-dien-8,12-olide and named cespitulolide (**16**).

### 2.4. Anti-Inflammatory Activities of the EtOAc Extract and the Isolated Compounds ***1**–**28***

**Figure 12:**
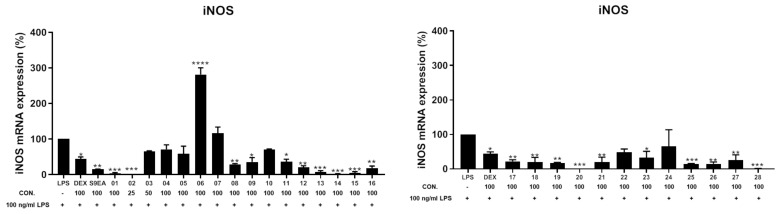
The inhibitory effect of **1**–**28** on LPS-induced iNOS mRNA expression in dendritic cells by the RT-PCR analysis. The values are mean SEM (*n* = 3); * *p* < 0.05, ** *p* < 0.01, *** *p* < 0.001, **** *p* < 0.0001 compared with the LPS alone stimulated group. The relative intensity of the LPS alone stimulated group was taken as 100%. The bar chart shows the results of the EtOAc extract of soft coral Cespitularia sp. (S9-EA) at 100 μg/mL and compounds **1**–**28** (25–100 μM) toward iNOS mRNA expression.

**Figure 13:**
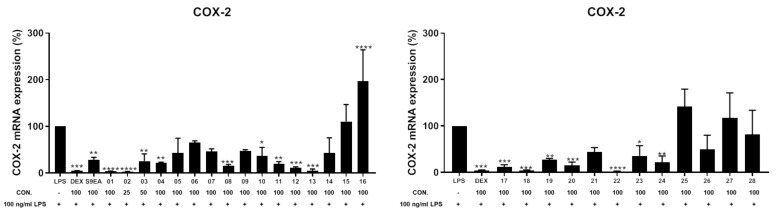
The inhibitory effect of **1**–**28** on LPS-induced COX-2 mRNA expression in dendritic cells by the RT-PCR analysis. The values are mean SEM (*n* = 3); * *p* < 0.05, ** *p* < 0.01, *** *p* < 0.001, **** *p* < 0.0001 compared with the LPS alone stimulated group. The relative intensity of the LPS alone stimulated group was taken as 100%. The bar chart shows the results of the EtOAc extract of soft coral Cespitularia sp. (S9-EA) at 100 μg/mL and compounds **1**–**28** (25–100 μM) toward COX-2 mRNA expression.

The anti-inflammatory activities of the EtOAc extract were screened in terms of the suppression of TNF-α production and NO release, as well as the inhibition of upregulation of pro-inflammatory iNOS and COX-2 gene, in LPS-induced DCs. The results of a preliminary study at a concentration of 100 μg/mL showed that the relative activities of this extract in inhibiting the production of TNF-α and NO were 84.1 ± 4.2 and 76.1 ± 1.4%, respectively, and it could reduce the levels of iNOS and COX-2 gene to 15.9 ± 0.4, and 28.6 ± 4.1%, respectively, too. For the discovery of bioactive compounds with anti-inflammatory abilities by inhibition of TNF-α and NO overproduction, **1**–**28** isolated from this extract were further assayed (Table 8). At a concentration of 100 μM, **1**–**3** could potently inhibit 95.0 ± 0.2, 95.7 ± 0.4, and 95.8 ± 0.1% TNF-α production, respectively, relative to the control cells treated with LPS only. The respective IC_50_ values of **1**–**3**, 47.2, 48.6, and 41.1 μM, were further measured. Compounds **2**, **12**, **19**, and **21** showed significant activities to inhibit NO releasing at 63.3 ± 1.6, 61.1 ± 0.5, 63.7 ± 0.8, and 61.7 ± 1.0%, respectively, at the same concentration. The IC_50_ values of 49.7, 51.9, and 57.4 μM, respectively, of **2**, **19**, and **21** in inhibiting the NO production were also measured. On the other hand, the anti-inflammatory potentials of compounds **1**–**28** in inhibition toward the accumulation of pro-inflammatory iNOS and COX-2 gene expression in the same LPS-induced DCs model were also evaluated (Figure 12 and Figure 13, and Table 9). At a concentration of 25 μM, **2** was found to effectively reduce the gene expression of iNOS and COX-2 to 0.3 ± 0.1 and 2.9 ± 0.6%, respectively, relative to the control cells stimulated with LPS only. Meanwhile, **1**, **13**–**15**, **20**, and **28** were found to conspicuously reduce the gene expression of iNOS to 3.6 ± 1.8, 7.4 ± 2.9, 1.5 ± 0.8, 4.6 ± 2.9, 0.2 ± 0.1, and 1.2 ± 0.5%, respectively, while **1**, **13**, **18**, and **22** could strongly reduce the COX-2 gene level to 4.2 ± 0.1, 4.4 ± 3.5, 4.5 ± 0.5, and 2.1 ± 0.4%, respectively, at a concentration of 100 μM. On the contrary, **6** significantly enhanced the gene expression of iNOS to 281.2 ± 15.4%, and **16** exhibited obvious activity of enhancing 196.9 ± 55.1% COX-2 gene expression, at the same concentration of 100 μM.

## 3. Discussion

The pro-inflammatory cytokine TNF-α, and reactive nitrogen species (RNS), such as NO, are shown to involve in the physiological regulation of immune responses [39,40,41]. The inducible enzymes iNOS and COX-2 are also critical regulators of inflammation [40,42]. iNOS and COX-2 are also known to express together in inflamed responses and overproduction of NO can enhance the expression of COX-2 protein [43]. Generally, the inappropriate production of TNF-α and NO, as well as high iNOS and COX-2 protein and gene expression were found to be related to the pathogenesis of many inflammatory related diseases [44,45,46,47,48,49,50,51] such as AIDS, Alzheimer’s, arthritis, cancer, diabetes, stroke, multiple sclerosis, obesity, and Parkinson’s disease. Therefore, substances with inhibitory ability toward the overproduction of these inflammatory mediators are candidates for the development of new pharmaceutics in the treatment of chronic inflammation and autoimmune diseases [52,53].

The anti-inflammatory potential of all isolated compounds revealed that compounds **1**–**3** and **12**–**15** might represent promising anti-inflammatory agents, in particular, **1** and **2** not only could significantly inhibit the production of TNF-α and NO but also displayed potent suppression to the expression of iNOS and COX-2 gene. Compound **13** might also be regarded as a promising inducible enzyme inhibitor as it can potently inhibit the expression of both iNOS and COX-2 genes.

From the structure−activity relationship (SAR), the tetracyclic verticillane-type diterpenes **1** and **2** were showing significant activities for each biological study relative to **3**–**8**, owing to the presence of an acrylate group at C-20. Furthermore, **2** exhibited stronger anti-inflammatory abilities at lower concentrations (25 μM) than **1** at higher concentrations (100 μM). Thus, the acrylate group at C-20 and the hydroxy group at C-6 in epoxyfuranyl verticillane-type metabolites could effectively enhance the anti-inflammatory activity. The bicyclic verticillane-type norditerpene **12** displayed more effective anti-inflammatory activities than **11**, suggesting that the presence of the α,β-conjugated ketone at C-13 as in **12** could strengthen activities from the allylic hydroxy group at C-13 as in **11**. On the other hand, the cadinane-type squiterpenes **13**–**15** exhibited significant inhibition toward iNOS gene expression, however, the presence of a hydroperoxy group and/or conjugated enone group as shown in **15** might promote the COX-2 gene expression.

## 4. Materials and Methods

### 4.1. General Experimental Procedures

Values of specific optical rotation were determined on a JASCO P-1020 digital polarimeter. UV spectra were recorded on a JASCO V-650 spectrophotometer. IR spectra were measured on a JASCO FT-IR-4100 and Nicolet iS5 FT-IR infrared spectrophotometers. ESIMS and HRESIMS data were obtained with a Bruker APEX II mass spectrometer. NMR spectra were recorded on a JEOL ECZ600R FT-NMR (or a Varian Unity INOVA 500 FT-NMR, or a Varian MR 400 FT-NMR) instrument at 600 MHz (or 500 MHz, or 400 MHz) for ^1^H and 150 MHz (or 125 MHz, or 100 MHz) for ^13^C, respectively. All NMR experiments were measured using CDCl_3_ or benzene-*d*_6_ as the solvent. Silica gel (Merck, 230–400 mesh) and Sephadex LH-20 (GE Healthcare, 25–100 μm) were used for column chromatography. High-performance liquid chromatography (HPLC) was performed on a HiTachi L-7100 HPLC system apparatus with a Supelco C18 (250 mm × 21.2 mm, 5 μm) or Hibar 250-10 C18 (250 mm × 21.2 mm, 5 μm) column. 

### 4.2. Animal Material

The soft coral *Cespitularia* sp. was collected by hand using SCUBA at Green Island, which is located off the southeastern coast of Taiwan, in June 2007, at a depth of 10–15 m, and was stored in a freezer until extraction. A voucher specimen was deposited in the Department of Marine Biotechnology, National Sun Yat-sen University, Kaohsiung, Taiwan.

### 4.3. Extraction and Isolation

The frozen specimens of *Cespitularia* sp. (87.20 g, dry weight) were sliced and exhaustively extracted with EtOAc (5 × 2 L) for 24 h. The solvent-free extract was obtained and further purified by reverse-phase HPLC to afford new compounds **1**–**16** (Figure 1) and known compounds **17**–**28** (Figure 2). The EtOAc extract (4.26 g) was subjected to silica gel open column chromatography (diameter: 8 cm; height: 30 cm) and eluted with a gradient of EtOAc in *n*-hexane (0–100%, stepwise), to furnish 15 fractions, A1–A15. Fraction A7, eluting with *n*-hexane–EtOAc (1:1), was purified over silica gel in open column (diameter: 2.5 cm; height: 50 cm) using *n*-hexane–EtOAc (1:1) to afford five subfractions A7-1–A7-5. Subfractions A7-3, A7-4, and A7-5 were further purified by reversed-phase (RP) HPLC using CH_3_CN–H_2_O (1:1), CH_3_CN–H_2_O (1:1.5), and MeOH–H_2_O (2:1), respectively, to afford **1** (1.5 mg), **15** (0.5 mg), **25** (30.1 mg), and **26** (1.0 mg) from A7-3, **7** (0.5 mg) and **19** (1.5 mg) from A7-4, and **3** (2.0 mg), **4** (0.6 mg), and **18** (1.3 mg) from A7-5. Fraction A-8, eluting with *n*-hexane–EtOAc (1:2), was separated by silica gel column (diameter: 2.5 cm; height: 50 cm) chromatography using *n*-hexane–EtOAc (1:2) to give subfractions A8-1–A8-7. RP-HPLC was further performed to purify subfraction A8-4, using MeOH–H_2_O (1.5:1) to afford **5** (1.4 mg) and **6** (1.1 mg), and MeOH–H_2_O (2:1) to afford **2** (2.2 mg), **27** (3.7 mg), and **28** (1.0 mg). Subfraction A8-5 was further separated by RP-HPLC using MeOH–H_2_O (1.5:1) to yield **10** (0.6 mg), **12** (1.0 mg), and **17** (6.6 mg). Subfraction A8-6 was purified by RP-HPLC using CH_3_CN–H_2_O (1:1.5) to afford **13** (1.2 mg), **14** (0.9 mg), and **20** (1.1 mg). Fraction A-9, eluting with *n*-hexane–EtOAc (1:4), was rechromatographed over silica gel column (diameter: 2.5 cm; height: 50 cm) using *n*-hexane–EtOAc (1:2) as the mobile phase to give nine subfractions, A9-1–A9-9. Subfractions A9-4 and A9-5 were purified by RP-HPLC using CH_3_CN–H_2_O (1:1.5) to afford **8** (1.0 mg) and **21** (1.4 mg), respectively. Subfraction A9-7 was further purified by RP-HPLC using CH_3_CN–H_2_O (1.5:1) to afford **22** (5.8 mg). Fraction A-10, eluting with *n*-hexane–EtOAc (1:8), was separated using sephadex LH-20 column (diameter: 3 cm; height: 100 cm) chromatography with 100% acetone to furnish seven subfractions (A10-1–A10-7). Subfraction A10-3 was purified by RP-HPLC (CH_3_CN–H_2_O, 1:1.5) to afford **23** (0.6 mg), and subfraction A10-5 was chromatographed using RP-HPLC (CH_3_CN–H_2_O, 1:2) to yield **9** (1.2 mg), **11** (1.1 mg), **16** (0.5 mg), and **24** (1.7 mg).

Cespitulin H (**1**): White amorphous powder; ${\left[\alpha \right]}_{D}^{25}$ +162 (*c* 0.43, CHCl_3_); UV (MeOH) λ_max_ (log ε) 239 (3.3) and 213 (3.4); IR(neat) *v*_max_ 3480, 2925, 1741, 1685, 1617, 1456, 1386, and 1170 cm^–1^; ^1^H and ^13^C NMR data, see Table 1 and Table 2; ESIMS *m/z* 425; HRESIMS *m/z* 425.1932 [M + Na]^+^ (calcd for C_23_H_30_O_6_Na, 425.1935).

Cespitulin I (**2**): White amorphous powder; ${\left[\alpha \right]}_{D}^{25}$ –91 (*c* 0.63, CHCl_3_); UV (MeOH) λ_max_ (log ε) 210 (3.4); IR(neat) *v*_max_ 3440, 2925, 1740, 1715, 1634, 1455, 1386, and 1166 cm^–1^; ^1^H and ^13^C NMR data, see Table 1 and Table 2; ESIMS *m/z* 427; HRESIMS *m/z* 427.2089 [M + Na]^+^ (calcd for C_23_H_32_O_6_Na, 427.2091).

Cespitulin J (**3**): Colorless oil; ${\left[\alpha \right]}_{D}^{25}$ +29 (*c* 0.57, CHCl_3_); IR(neat) *v*_max_ 3446, 2923, 2853, 1758, 1636, 1457, 1385, and 1164 cm^–1^; ^1^H and ^13^C NMR data, see Table 1 and Table 2; ESIMS *m/z* 611; HRESIMS *m/z* 611.4282 [M + Na]^+^ (calcd for C_36_H_60_O_6_Na, 611.4282).

Cespitulin K (**4**): Colorless oil; ${\left[\alpha \right]}_{D}^{25}$ +38 (*c* 0.17, CHCl_3_); IR(neat) *v*_max_ 3420, 2924, 2853, 1748, 1636, 1457, 1386, and 1111 cm^–1^; ^1^H and ^13^C NMR data, see Table 1 and Table 2; ESIMS *m/z* 637; HRESIMS *m/z* 637.4440 [M + Na]^+^ (calcd for C_38_H_62_O_6_Na, 637.4439).

Cespitulin L (**5**): White amorphous powder; ${\left[\alpha \right]}_{D}^{25}$ +35 (*c* 0.40, CHCl_3_); IR(neat) *v*_max_ 3445, 2920, 1683, 1652, 1455, 1385, and 1187 cm^–1^; ^1^H and ^13^C NMR data, see Table 1 and Table 2; ESIMS *m/z* 365; HRESIMS *m/z* 365.2315 [M + H]^+^ (calcd for C_21_H_33_O_5_, 365.2323).

Cespitulin M (**6**): White amorphous powder; ${\left[\alpha \right]}_{D}^{25}$ –23 (*c* 0.31, CHCl_3_); IR(neat) *v*_max_ 3446, 2917, 1683, 1652, 1456, 1386, and 1209 cm^–1^; ^1^H and ^13^C NMR data, see Table 1 and Table 3; ESIMS *m/z* 387; HRESIMS *m/z* 387.2142 [M + Na]^+^ (calcd for C_21_H_32_O_5_Na, 387.2142).

Cespitulin N (**7**): White amorphous powder; ${\left[\alpha \right]}_{D}^{25}$ +102 (*c* 0.14, CHCl_3_); IR(neat) *v*_max_ 3446, 2917, 1733, 1652, 1456, 1386, and 1239 cm^–1^; ^1^H and ^13^C NMR data, see Table 3 and Table 4; ESIMS *m/z* 399; HRESIMS *m/z* 399.2142 [M + Na]^+^ (calcd for C_22_H_32_O_5_Na, 399.2142).

Cespitulin O (**8**): Colorless oil; ${\left[\alpha \right]}_{D}^{25}$ –63 (*c* 0.29, CHCl_3_); IR(neat) *v*_max_ 3419, 2922, 1733, 1652, 1456, 1386, and 1224 cm^–1^; ^1^H and ^13^C NMR data, see Table 3 and Table 4; ESIMS *m/z* 393; HRESIMS *m/z* 393.2267 [M + H]^+^ (calcd for C_22_H_33_O_6_, 393.2272).

Cespitulactam L (**9**): Colorless oil; ${\left[\alpha \right]}_{D}^{25}$ –132 (*c* 0.34, CHCl_3_); UV (MeOH) λ_max_ (log ε) 221 (3.4); IR(neat) *v*_max_ 3245, 2919, 1698, 1647, 1457, 1387, and 1204 cm^–1^; ^1^H and ^13^C NMR data, see Table 3 and Table 4; ESIMS *m/z* 368; HRESIMS *m/z* 368.2195 [M + Na]^+^ (calcd for C_21_H_31_O_3_NNa, 368.2196).

Cespitulin P (**10**): Colorless oil; ${\left[\alpha \right]}_{D}^{25}$ +68 (*c* 0.17, CHCl_3_); IR(neat) *v*_max_ 3445, 2917, 1732, 1715, 1651, 1455, 1385, and 1219 cm^–1^; ^1^H and ^13^C NMR data, see Table 5; ESIMS *m/z* 375; HRESIMS *m/z* 375.2141 [M + Na]^+^ (calcd for C_20_H_32_O_5_Na, 375.2142).

Cespitulin Q (**11**): Colorless oil; ${\left[\alpha \right]}_{D}^{25}$ +176 (*c* 0.31, CHCl_3_); UV (MeOH) λ_max_ (log ε) 212 (3.3); IR(neat) *v*_max_ 3418, 2917, 1699, 1652, 1456, 1386, and 1232 cm^–1^; ^1^H and ^13^C NMR data, see Table 3 and Table 4; ESIMS *m/z* 305; HRESIMS *m/z* 305.2108 [M + H]^+^ (calcd for C_19_H_29_O_3_, 305.2111).

Cespitulin R (**12**): White amorphous powder; ${\left[\alpha \right]}_{D}^{25}$ +46 (*c* 0.29, CHCl_3_); UV (MeOH) λ_max_ (log ε) 225 (3.2); IR(neat) *v*_max_ 3420, 2919, 1748, 1684, 1653, 1457, 1387, and 1223 cm^–1^; ^1^H and ^13^C NMR data, see Table 3 and Table 4; ESIMS *m/z* 325; HRESIMS *m/z* 325.1777 [M + Na]^+^ (calcd for C_19_H_26_O_3_Na, 325.1774).

Cespilin A (**13**): Colorless oil; ${\left[\alpha \right]}_{D}^{25}$ +56 (*c* 0.34, CHCl_3_); IR(neat) *v*_max_ 3392, 2926, 2870, 1652, 1456, 1380, and 1050 cm^–1^; ^1^H and ^13^C NMR data, see Table 6 and Table 7; ESIMS *m/z* 261; HRESIMS *m/z* 261.1824 [M + Na]^+^ (calcd for C_15_H_26_O_2_Na, 261.1825).

Cespilin B (**14**): Colorless oil; ${\left[\alpha \right]}_{D}^{25}$ +60 (*c* 0.26, CHCl_3_); IR(neat) *v*_max_ 3357, 2925, 2869, 1652, 1456, 1381, 1060 cm^–1^; ^1^H and ^13^C NMR data, see Table 6 and Table 7; ESIMS *m/z* 261; HRESIMS *m/z* 261.1824 [M + Na]^+^ (calcd for C_15_H_26_O_2_Na, 261.1825).

Cespilin C (**15**): Colorless oil; ${\left[\alpha \right]}_{D}^{25}$ –39 (*c* 0.14, CHCl_3_); UV (MeOH) λ_max_ (log ε) 211 (3.1); IR(neat) *v*_max_ 2924, 2851, 1683, 1653, 1456, 1376, and 1301 cm^–1^; ^1^H and ^13^C NMR data, see Table 6 and Table 7; ESIMS *m/z* 273; HRESIMS *m/z* 273.1464 [M + Na]^+^ (calcd for C_15_H_22_O_3_Na, 273.1461).

Cespitulolide (**16**): White amorphous powder; ${\left[\alpha \right]}_{D}^{25}$ +136 (*c* 0.14, CHCl_3_); UV (MeOH) λ_max_ (log ε) 213 (3.2); IR(neat) *v*_max_ 3418, 2917, 1732, 1651, 1455, 1385, and 1217 cm^–1^; ^1^H and ^13^C NMR data, see Table 6 and Table 7; ESIMS *m/z* 287; HRESIMS *m/z* 287.1255 [M + Na]^+^ (calcd for C_15_H_20_O_4_Na, 287.1254).

### 4.4. In Vitro Anti-Inflammatory Assay

#### 4.4.1. Measurement of Cytokine Production by Dendritic Cells (DCs)

The experiment for measuring cytokine was tested by enzyme-link immunosorbent assay (ELISA) from the previously reported method [6,7]. The DCs were manipulated with lipopolysaccharide (LPS, 100 ng/mL) from *Escherichia coli* 055:B5, and the following treatment with the isolated compounds for 24 h. The optical density of the production of TNF-α was measured at 450 nm using the ELISA reader.

#### 4.4.2. Measurement of Nitric Oxide (NO) Production by DCs

DC cells were seeded in 24-well plates at a density of 1 × 10^6^/mL. DCs were treated with each compound for 1 h and then stimulated with 100 ng/mL LPS for 24 h. The nitrite concentration in the medium was measured as an indicator of NO production through the Griess reaction. Briefly, 100 μL of cell culture supernatant was reacted with 100 μL of Griess reagent (1:1 mixture of 2% sulfanilamide and 0.2% *N*-(1-naphthyl-)ethylenediamine dihydrochloride in water) in 96-well plate at room temperature for 10 min, and absorbance at 540 nm was recorded using sandwich ELISA assays [6,7].

#### 4.4.3. Measurement of Pro-Inflammatory Inducible NO Synthase (iNOS) and Cyclooxygenase-2 (COX-2) Gene Expression by DCs

The suppression activities of compounds were measured by the examining suppression of LPS-induced upregulation of pro-inflammatory iNOS and COX-2 gene expression in DCs using real-time polymerase chain reaction (PCR) [14]. Briefly, DCs (1 × 10^6^/mL) were incubated in 6-well plates and treated with each compound for 1 h, and then were added the LPS (100 ng/mL), stimulating for 24 h. Subsequently, cells were harvested and isolated total RNA using Trizol reagent. A total of 2 μg RNA was reverse-transcribed using M-MLV Reverse Transcriptase to synthesize cDNA (Applied Biosystems). Gene expression levels of iNOS and COX-2 were analyzed using SYBR-Green PCR Master Mix with StepOne PCR System (Applied Biosystems; Thermo Fisher Scientific). Relative gene expression levels were calculated using the 2^−ΔΔCt^ method and normalized to GAPDH; all the primers which were used are listed in Table 10 [54].

### 4.5. Statistical Analysis

The results are expressed as the mean ± SEM, and comparisons were made using one-way ANOVA by Tukey’s post hoc test (Graphpad Prism 5.0, GraphPad Software, San Diego, CA, USA). A probability value of 0.05 or less was considered significant. The software Sigma Plot was used for the statistical analysis.

## 5. Conclusions

In conclusion, our chemical investigation demonstrated that the soft coral *Cespitularia* sp. could be a good source of bioactive substances. Eight new tricyclic verticillane-type diterpenes **1**–**9**, one novel norditerpene **10**, two new dicyclic verticillane-type norditerpenes **11** and **12**, three cadinane-type sesquiterpenes **13**–**15**, and one eudesmane-type sesquiterpenoid **16**, along with twelve known metabolites **17**–**28**, were isolated from this investigation. The structural framework of verticillane-type derivatives was found to be close to the tricyclic taxane skeleton [55] and obtained from marine organisms only in the soft coral genus *Cespitularia* [15]. Furthermore, the cadinane-type sesquiterpenes **13**–**15** were isolated from the soft coral genus *Cespitularia* for the first time. From the results of the evaluated biological activities, it appears that compounds **1**, **2**, and **13** might be promising compounds for further marine anti-inflammatory drug development.

## Figures and Tables

**Table 1:** ^1^^3^C NMR spectroscopic data of compounds **1**–**6**.

No.	1 ^1^	2 ^2^	3 ^3^	4 ^3^	5 ^3^	6 ^3^
1	44.1, CH ^4^	44.0, CH	44.1, CH	44.1, CH	44.2, CH	44.2, CH
2	34.4, CH_2_	33.8, CH_2_	33.8, CH_2_	33.8, CH_2_	34.0, CH_2_	33.6, CH_2_
3	38.7, CH_2_	37.7, CH_2_	37.7, CH_2_	37.7, CH_2_	37.8, CH_2_	37.8, CH_2_
4	144.1, C	145.7, C	145.7, C	145.7, C	145.8, C	145.7, C
5	55.2, CH_2_	45.8, CH_2_	45.8, CH_2_	47.5, CH_2_	45.9, CH_2_	45.6, CH_2_
6	197.7, C	69.2, CH	69.2, CH	69.2, CH	69.3, CH	69.1, CH
7	129.4, CH	133.5, CH	133.5, CH	133.5, CH	133.3, CH	133.2, CH
8	148.5, C	132.4, C	132.0, C	132.4, C	132.8, C	132.8, C
9	41.0, CH_2_	40.7, CH_2_	40.7, CH_2_	40.7, CH_2_	41.0, CH_2_	41.0, CH_2_
10	94.6, C	94.7, C	94.7, C	94.6, C	94.3, C	94.4, C
11	72.8, C	72.4, C	72.4, C	72.4, C	72.8, C	72.9, C
12	80.0, C	79.8, C	79.7, C	79.7, C	78.3, C	79.9, C
13	26.8, CH_2_	26.2, CH_2_	26.2, CH_2_	26.2, CH_2_	31.6, CH_2_	25.2, CH_2_
14	23.8, CH_2_	25.4, CH_2_	25.4, CH_2_	25.4, CH_2_	26.2, CH_2_	25.5, CH_2_
15	38.1, C	37.6, C	37.6, C	37.6, C	37.6, C	37.7, C
16	24.9, CH_3_	25.1, CH_3_	25.2, CH_3_	25.2, CH_3_	25.1, CH_3_	25.3, CH_3_
17	26.8, CH_3_	26.4, CH_3_	26.4, CH_3_	26.4, CH_3_	26.0, CH_3_	26.4, CH_3_
18	116.5, CH_2_	115.8, CH_2_	115.8, CH_2_	115.8, CH_2_	115.7, CH_2_	115.7, CH_2_
19	19.2, CH_3_	17.2, CH_3_	17.4, CH_3_	17.2, CH_3_	17.4, CH_3_	17.4, CH_3_
20	101.8, CH	101.3, CH	100.8, CH	100.8, CH	104.6, CH	109.1, CH
21	164.9, C	164.8, C			56.6, CH_3_	57.5, CH_3_
22	127.6, CH	127.3, CH				
23	132.6, CH_2_	132.7, CH_2_				
1′			172.8, C	172.8, C		
2′			34.1, CH_2_	34.1, CH_2_		
3′			24.7, CH_2_	24.7, CH_2_		
4′-13′			29.7 × 2, 29.6 × 3, 29.4 × 3, 29.2, 29.0, each CH_2_			
4′-7′/12′-15′				29.8, 29.7, 29.5, 29.3 × 2, 29.2 × 2, 29.0, each CH_2_		
8′/11′				27.2/27.1, each CH_2_		
9′-10′				130.1/129.7, each CH		
14′			31.9, CH_2_			
15′			22.7, CH_2_			
16′			14.1, CH_3_	31.9, CH_2_		
17′				22.7, CH_2_		
18′				14.1, CH_3_		

^1^ Spectrum recorded at 500 MHz in benzene-*d*_6_. ^2^ Spectrum recorded at 400 MHz in CDCl_3_. ^3^ Spectrum recorded at 500 MHz in CDCl_3_. ^4^ Multiplicities deduced by the HSQC experiment.

**Table 2:** ^1^H NMR spectroscopic data of compounds **1**–**5**.

No.	1 ^1^	2 ^2^	3 ^3^	4 ^3^	5 ^3^
1	1.19, m	1.48, m	1.49, m	1.49, m	1.46, m
2	1.66, m	1.82, m	1.81, m	1.81, m	1.84, m
	1.38, m	1.11, td (14.0, 5.0)	1.11, td (14.5, 5.0)	1.11, td (14.0, 5.5)	1.09, td (14.5, 4.5)
3	1.94, td (13.0, 4.0) ^4^	2.22, m	2.22 m	2.22, m	2.20, td (14.0, 4.5)
	1.82, td (13.0, 4.0)	2.08, td (14.0, 3.2)	2.08, td (14.0, 4.0)	2.08, m	2.08, dd (14.0, 4.5)
5	2.94, d (11.0)	2.65, dd (12.5, 3.2)	2.66, dd (13.0, 3.0)	2.66, dd (12.5, 2.5)	2.65, dd (13.0, 3.0)
	2.85, d (11.0)	2.24, m	2.25, m	2.25, m	2.22, m
6		4.49, t (8.0)	4.50, quint (3.0)	4.50, m	4.50, br t (8.5)
7	6.13, s	5.47, d (8.0)	5.47, d (8.5)	5.47, d (8.0)	5.46, d (8.0)
9	3.00, d (16.0)	3.10, d (14.4)	3.09, d (14.5)	3.09, d (14.0)	3.03, d (14.5)
	2.21, d (16.0)	2.55, d (14.4)	2.54, d (14.5)	2.54, d (14.0)	2.52, d (14.5)
13	1.57, td (14.0, 3.5)	1.71, td (14.0, 3.6)	1.67, m	1.66, m	1.70, br d (14.0)
	1.46, m	1.58, m	1.50, m	1.50, m	1.58, m
14	2.16, m	2.28, m	2.28, m	2.27, m	2.31, tt (17.5, 3.5)
	1.11, ddd (14.0, 6.0, 3.5)	1.37, m	1.37, m	1.37, m	1.33, m
16	0.72, 3H, s	0.94, 3H, s	0.98, 3H, s	0.98, 3H, s	0.95, 3H, s
17	1.35, 3H, s	1.34, 3H, s	1.33, 3H, s	1.33, 3H, s	1.31, 3H, s
18	5.09, 4.77, both s	4.93, 2H, s	4.94, 2H, s	4.94, 2H, s	4.93, 2H, s
19	2.11, 3H, s	1.81, 3H, s	1.81, 3H, s	1.81, 3H, s	1.83, 3H, s
20	5.96, s	5.70, s	5.63, s	5.63, s	4.36, s
21					3.47, 3H, s
22	5.77, dd (17.5, 10.5)	6.14, dd (17.2, 10.4)			
23	6.15, br d (17.5)	6.47, d (17.2)			
	5.16, dd (10.5, 1.0)	5.94, d (10.4)			
2′			2.36, t (7.5)	2.36, t (7.5)	
3′			1.62, 2H, m	1.63, 2H, m	
4′-15′			1.20–1.31, 20H, m		
4′-7′/12′-17′				1.25–1.34, 20H, m	
8′/11′				2.01, H, m	
9′/10′				5.34, dd (10.5, 6.5)	
16′			0.88, 3H, t (7.0)		
18′				0.88, 3H, t (7.0)	
12-OH	2.25, br s	2.64, br s	2.52, br s	2.53, br s	3.29, br s

^1^ Spectrum recorded at 500 MHz in benzene-*d*_6_. ^2^ Spectrum recorded at 400 MHz in CDCl_3_. ^3^ Spectrum recorded at 500 MHz in CDCl_3_. ^4^ *J* values are in Hz.

**Table 3:** ^1^H NMR spectroscopic data of compounds **6**–**9**, **11**, and **12**.

No.	6 ^1^	7 ^1^	8 ^1^	9 ^1^	11 ^1^	12 ^1^
1	1.45, m	1.46, m	1.49, m	1.54, m	1.81, m	2.15, m
2	1.79, m	1.79, m	1.84, m	1.55, m	1.92, m	2.46, m
	1.11, td (14.0, 5.0) ^2^	1.11, m	1.11, td (14.0, 5.0)	1.36, m	1.52, m	1.73, m
3	2.20, m	2.23, m	2.22, m	2.27, m	2.59, dd	1.94, m
					(15.0, 11.0)	
	2.09, m	2.01, m	2.08, m	2.13, m	1.86, m	1.56, m
5	2.64, dd	2.60, dd	2.65, dd	2.44, dd	2.48, dd	2.43, m
	(13.0, 3.0)	(13.0, 3.5)	(13.0, 3.0)	(13.5, 2.5)	(13.0, 7.0)	
	2.23, m	2.30, d (13.0)	2.24, m	2.33, m	2.28, dd (13.0, 2.5)	2.34, dd (13.5, 3.0)
6	4.51, m	5.49, td (9.0, 3.5)	4.50, br t (8.0)	4.38, m	4.49, m	4.44, m
7	5.46, d (8.0)	5.41, d (9.0)	5.48, d (9.0)	5.55, d (8.0)	5.15, d (6.5)	5.28, d (7.0)
9	3.03, d (14.5)	3.04, d (14.5)	3.05, d (14.5)	3.00, d (14.5)	3.43, d (15.0)	3.43, d (15.5)
	2.54, d (14.5)	2.55, d (14.5)	2.54, d (14.5)	2.66, d (14.5)	3.08, d (15.0)	3.24, d (15.5)
12					6.10, d (3.5)	6.07, s
13	1.67, m	1.76, m	1.81, m	2.34, m	4.50, m	
	1.56, m	1.63, m	1.62, m	2.18, m		
14	2.21, m	2.25, m	2.30, tt	2.19, m	2.13, 2H, m	3.02, dd
			(14.5, 4.0)			(18.5, 7.0)
	1.35, m	1.33, m	1.40, m	1.63, m		2.45, m
16	0.98, 3H, s	1.00, 3H, s	0.98, 3H, s	1.47, 3H, s	1.09, 3H, s	1.23, 3H, s
17	1.31, 3H, s	1.33, 3H, s	1.33, 3H, s	1.24, 3H, s	1.40, 3H, s	1.51, 3H, s
18	4.92, 2H, s	4.96, 4.92, both s	4.94, 2H, s	4.84, 2H, s	4.87, 4.83, both s	4.84, 4.78, both s
19	1.84, 3H, s	1.88, 3H, s	1.83, 3H, s	1.58, 3H, s	1.76, 3H, s	1.78, 3H, s
20	4.47, s	3.59, 3.43, both d (9.0)	5.76, s			
21	3.46, 3H, s			3.13, 3H, s		
22		2.02, 3H, s	2.14, 3H, s			
12-OH	2.63, br s	1.96, br d (2.0)	2.68, br s			
N-H				5.46, br s		

^1^ Spectrum recorded at 500 MHz in CDCl_3_. ^2^ *J* values are in Hz.

**Table 4:** ^1^^3^C NMR spectroscopic data of compounds **7**–**9**, **11**, and **12**.

No.	7 ^1^	8 ^1^	9 ^1^	11 ^1^	12 ^1^
1	44.3, CH ^2^	44.1, CH	44.8, CH	45.8, CH	45.4, CH
2	34.2, CH_2_	33.7, CH_2_	32.4, CH_2_	29.5, CH_2_	30.7, CH_2_
3	37.8, CH_2_	37.7, CH_2_	34.4, CH_2_	31.0, CH_2_	30.6, CH_2_
4	145.3, C	145.5, C	146.1, C	146.0, C	145.4, C
5	43.4, CH_2_	45.8, CH_2_	43.9, CH_2_	44.8, CH_2_	44.2, CH_2_
6	72.2, CH	69.2, CH	68.3, CH	69.0, CH	69.5, CH
7	128.7, CH	133.7, CH	135.7, CH	133.6, CH	135.3, CH
8	134.5, C	132.5, C	131.3, C	130.0, C	129.1, C
9	41.3, CH_2_	40.6, CH_2_	48.5, CH_2_	51.9, CH_2_	52.4, CH_2_
10	95.9, C	95.1, C	93.9, C	202.6, C	202.2, C
11	74.0, C	73.0, C	160.1, C	150.3, C	166.0, C
12	78.7, C	78.5, C	133.8, CH	134.6, CH	128.6, CH
13	31.1, CH_2_	30.6, CH_2_	17.5, CH_2_	65.9, CH	199.0, C
14	26.2, CH_2_	26.0, CH_2_	24.8, CH_2_	34.9, CH_2_	40.0, CH_2_
15	37.5, C	37.4, C	37.7, C	35.8, C	37.0, C
16	25.2, CH_3_	25.0, CH_3_	24.8, CH_3_	23.8, CH_3_	23.6, CH_3_
17	26.3, CH_3_	26.0, CH_3_	34.1, CH_3_	33.4, CH_3_	32.3, CH_3_
18	115.8, CH_2_	115.9, CH_2_	114.8, CH_2_	112.6, CH_2_	113.4, CH_2_
19	17.5, CH_3_	17.4, CH_3_	17.6, CH_3_	18.8, CH_3_	18.9, CH_3_
20	75.5, CH_2_	96.1, CH	171.6, C		
21	170.2, C	169.7, C	50.4, CH_3_		
22	21.3, CH_3_	21.2, CH_3_			

^1^ Spectrum recorded at 500 MHz in CDCl_3_. ^2^ Multiplicities deduced by the HSQC experiment.

**Table 5:** ^1^H and ^13^C NMR spectroscopic data of compound **10**.

No.	10	
	^1^H ^1^	^13^C ^2^
1		48.4, C
2		214.6, C
3	4.45, m	71.4, CH ^4^
4	2.31, m	31.6, CH_2_
	1.58, m	
5	2.13, m	21.0, CH_2_
	1.68, m	
6	1.79, m	46.9, CH
7	1.64, m	25.7, CH_2_
	1.13, m	
8	2.11, m	33.8, CH_2_
	1.92, m	
9		147.1, C
10	2.20, 2H, m	43.9, CH_2_
11	4.47, m	66.1, CH
12	5.32, br d (8.5) ^3^	131.7, CH
13		122.2, C
14	3.02, 2H, br s	44.6, CH_2_
15		171.4, C
16	1.11, 3H, s	21.8, CH_3_
17	1.32, 3H, s	27.0, CH_3_
18	4.87, 2H, s	113.2, CH_2_
19	1.77, 3H, s	16.9, CH_3_
20	3.69, 3H, s	51.8, CH_3_

^1^ Spectrum recorded at 500 MHz in CDCl_3_. ^2^ Spectrum recorded at 125 MHz in CDCl_3_. ^3^ *J* values are in Hz. ^4^ Multiplicities deduced by the HSQC experiment.

**Table 6:** ^1^^3^C NMR spectroscopic data of compounds **13**–**16**.

No.	13 ^1^	14 ^2^	15 ^3^	16 ^2^
1	34.7, CH ^4^	33.6, CH	162.7, C	35.2, CH_2_
2	29.1, CH_2_	28.8, CH_2_	128.6, CH	29.0, CH_2_
3	22.7, CH_2_	22.5, CH_2_	197.7, C	72.7, CH
4	51.1, CH	50.9, CH	57.1, CH	149.8, C
5	125.8, CH	125.4, CH	31.7, CH_2_	45.6, CH
6	71.2, CH	70.8, CH	25.1, CH	24.0, CH_2_
7	31.1, CH_2_	31.4, CH_2_	29.1, CH_2_	159.4, C
8	23.2, CH_2_	22.3, CH_2_	29.8, CH_2_	103.1, C
9	36.6, CH	37.5, CH	84.0, C	51.0, CH_2_
10	147.0, C	146.1, C	36.8, CH	36.6, C
11	26.6, CH	26.8, CH	140.5, C	123.8, C
12	21.2, CH_3_	21.3, CH_3_	18.3, CH_3_	174.7, C
13	21.7, CH_3_	21.6, CH_3_	117.1, CH_2_	8.34, CH_3_
14	14.4, CH_3_	14.4, CH_3_	18.0, CH_3_	15.9, CH_3_
15	68.9, CH_2_	69.9, CH_2_	22.1, CH_3_	110.1, CH_2_

^1^ Spectrum recorded at 400 MHz in CDCl_3_. ^2^ Spectrum recorded at 600 MHz in CDCl_3_. ^3^ Spectrum recorded at 500 MHz in CDCl_3_^4^ Multiplicities deduced by the HSQC experiment.

**Table 7:** ^1^H NMR spectroscopic data of compounds **13**–**16**.

No.	13 ^1^	14 ^2^	15 ^3^	16 ^2^
1	1.96, m	1.97, m		1.71 td (13.8, 4.2)
				1.38, m
2	1.82, m	1.82, m	5.95, d (1.0)	1.87 dt (14.4, 3.6)
	1.31, br t (10.8) ^4^	1.35, dd (10.8, 4.8)		1.78 dtd (14.4, 4.2, 2.4)
3	1.70, m	1.70, m		4.37, br s
	1.66, m	1.65, m		
4	1.63, m	1.64, m	3.26, d (13.5)	
5	5.45, s	5.50, s	1.46, m	2.45, br s
6			1.61, m	2.60, d (10.2)
				2.44, d (10.2)
7	1.83, m	1.67, m	1.54, m	
	1.46, d (9.2)	1.46, dd (3.6, 1.8)	1.22, m	
8	1.80, m	1.64, m	1.91, br d (14.5)	
	1.46, d (9.2)	1.50, m	1.71, td (14.5, 5.0)	
9	2.31, br d (4.4)	2.24, td (13.2, 4.8)		2.26, d (13.8)
				1.65, d (13.8)
10			2.90, br d (13.5)	
11	1.84, m	1.81, m		
12	0.92, 3H, d (6.8)	0.92, 3H, d (6.6)	1.66, 3H, s	
13	0.78, 3H, d (6.8)	0.70, 3H, d (6.6)	5.08, 4.87, both s	1.84, 3H, s
14	0.84, 3H, d (6.8)	0.90, 3H, d (6.6)	2.05, 3H, s	1.03, 3H, s
15	3.49, 2H, qd (10.8, 4.8)	3.49, d (10.8)	0.90, d (6.5)	5.11, s
		3.43, d (10.8)		4.77, s
9-OOH			7.42, br s	

^1^ Spectrum recorded at 400 MHz in CDCl_3_. ^2^ Spectrum recorded at 600 MHz in CDCl_3_. ^3^ Spectrum recorded at 500 MHz in CDCl_3_. ^4^ *J* values are in Hz.

**Table 8:** Inhibitory effects of compounds **1**–**28** on TNF-α expression and NO production in LPS-induced dendritic cells.

No.	TNF-α Expression		NO Production	
	Inh % ^1^		Inh %	
**S9-EA** ^2^	84.1 ± 4.2	****	76.1 ± 1.4	****
**1**	95.0 ± 0.2	****	39.8 ± 0.6	****
**2**	95.7 ± 0.4	****	63.3 ± 1.6	****
**3**	95.8 ± 0.1	****	44.0 ± 0.9	****
**4**	32.6 ± 11.6	*	51.1 ± 0.1	****
**5**	39.5 ± 7.8	**	56.8 ± 0.2	****
**6**	29.8 ± 6.7		43.3 ± 2.0	****
**7**	48.4 ± 10.9	***	48.6 ± 1.5	****
**8**	29.7 ± 17.9		55.6 ± 1.4	****
**9**	46.6 ± 2.6	***	50.7 ± 0.6	****
**10**	44.1 ± 7.1	**	46.7 ± 0.6	****
**11**	34.3 ± 1.7	*	50.8 ± 0.6	****
**12**	44.4 ± 2.7	**	61.1 ± 0.5	****
**13**	48.3 ± 12.6	***	44.5 ± 0.1	****
**14**	33.3 ± 4.8	*	55.6 ± 1.3	****
**15**	19.5 ± 2.9		49.6 ± 0.4	****
**16**	42.2 ± 4.1	**	41.9 ± 1.5	****
**17**	36.1 ± 0.6	****	58.6 ± 0.9	****
**18**	52.3 ± 6.1	****	58.5 ± 1.1	****
**19**	46.3 ± 4.4	****	63.7 ± 0.8	****
**20**	42.7 ± 5.1	****	56.3 ± 0.6	****
**21**	41.9 ± 3.2	****	61.7 ± 1.0	****
**22**	4.9 ± 7.7		38.2 ± 1.1	****
**23**	5.3 ± 8.8		59.3 ± 9.4	****
**24**	−0.6 ± 2.0		30.4 ± 2.9	****
**25**	−4.7 ± 0.9		29.6 ± 8.9	****
**26**	3.8 ± 6.2		9.5 ± 4.3	****
**27**	5.8 ± 3.5		15.3 ± 2.5	
**28**	−3.5 ± 7.4		6.3 ± 6.0	
DEX ^3^	85.6 ± 3.4	****	73.4 ± 1.3	****

^1^ Percentage of inhibition (Inh %) at a concentration 100 μM for **1–28** and 100 μg/mL for S9-EA compared with the control group (100 % for stimulated LPS alone). Results are presented as mean ± SEM. (*n* = 3–4). * *p* < 0.05, ** *p* < 0.01, *** *p* < 0.001, **** *p* < 0.0001. ^2^ S9 EA: the EtOAc extract of soft coral *Cespitularia* sp. ^3^ Positive control: dexamethasone (DEX) at 100 μM.

**Table 9:** Inhibitory effects of compounds **1**–**28** on iNOS and COX-2 mRNA expression in LPS-induced dendritic cells.

No.	iNOS mRNA		COX-2 mRNA	
	Exp % ^1^		Exp %	
**S9-EA** ^2^	15.9 ± 0.4	**	28.6 ± 4.1	**
**1**	3.6 ± 1.8	***	4.2 ± 0.1	****
**2**	0.3 ± 0.1	***	2.9 ± 0.6	****
**3**	64.6 ± 1.8		25.6 ± 12.7	**
**4**	67.4 ± 11.6		21.8 ± 1.1	**
**5**	71.0 ± 10.2		43.1 ± 25.8	
**6**	281.2 ± 15.4	****	65.4 ± 3.1	
**7**	115.9 ± 14.3		46.7 ± 4.4	
**8**	29.0 ± 1.7	**	15.5 ± 2.4	***
**9**	35.2 ± 10.1	*	47.9 ± 1.8	
**10**	70.0 ± 1.8		36.9 ± 14.7	*
**11**	36.0 ± 5.8	*	19.6 ± 4.1	**
**12**	20.5 ± 3.4	**	11.2 ± 1.7	***
**13**	7.4 ± 2.9	***	4.4 ± 3.5	***
**14**	1.5 ± 0.8	***	43.4 ± 26.2	
**15**	4.6 ± 2.9	***	110.5 ± 29.7	
**16**	18.4 ± 4.4	**	196.9 ± 55.1	****
**17**	22.0 ± 3.8	**	11.9 ± 3.7	***
**18**	19.7 ± 11.3	**	4.5 ± 0.5	***
**19**	17.7 ± 1.3	**	27.6 ± 1.9	**
**20**	0.2 ± 0.1	***	14.8 ± 6.1	***
**21**	19.8 ± 11.6	**	43.8 ± 7.9	
**22**	48.2 ± 7.9		2.1 ± 0.4	****
**23**	32.7 ± 14.9	*	35.4 ± 18.0	*
**24**	66.0 ± 38.8		21.7 ± 11.1	**
**25**	13.7 ± 1.9	***	141.5 ± 30.1	
**26**	15.0 ± 4.1	**	49.3 ± 25.0	
**27**	25.4 ± 12.6	**	117.6 ± 43.8	
**28**	1.2 ± 0.5	***	81.8 ± 42.4	
DEX ^3^	44.3 ± 4.3	*	4.5 ± 0.5	***

^1^ Percentage of expression (Exp %) at a concentration 100 μM (except for **2**:25 μM, **3**:50 μM, and S9-EA: 100 μg/mL) compared with the control group (100 % for stimulated LPS alone). Results are presented as mean ± SEM. (*n* = 3–4). * *p* < 0.05, ** *p* < 0.01, *** *p* < 0.001, **** *p* < 0.0001. ^2^ S9 EA: the EtOAc extract of soft coral *Cespitularia* sp. ^3^ Positive control: dexamethasone (DEX) at 100 μM.

**Table 10:** Primers of quantitative RT-PCR.

Gene	Reverse Primer	Forward Primer
iNOS	5′-CCAATGTTTCCCTGACTTTCCCA-3′	5′-CAGAGGGGTAGGCTTGTCTC-3′
COX-2	5′-CAGGAGGATGGAGTTGTTGTAG-3′	5′-ACCAGCAGTTCCAGTATCAGA-3′
GAPDH	5′-TGTCATCATACTTGGCAGGTTTCT-3′	5′-CGTGTTCCTACCCCCAATGT-3′

## Data Availability

Data is contained within the article and Supplementary Material.

## Supplementary Materials

The following are available online at https://www.mdpi.com/article/10.3390/ph14121252/s1, Figure S1: HRESIMS spectrum of **1**, Figure S2: ^1^H NMR spectrum of **1** in C_6_D_6_ at 500 MHz, Figure S3: ^13^C NMR spectrum of **1** in C_6_D_6_ at 125 MHz, Figure S4: HSQC spectrum of **1** in C_6_D_6_, Figure S5: COSY spectrum of **1** in C_6_D_6_, Figure S6: HMBC spectrum of **1** in C_6_D_6_, Figure S7: NOESY spectrum of **1** in C_6_D_6_, Figure S8: HRESIMS spectrum of **2**, Figure S9: ^1^H NMR spectrum of **2** in CDCl_3_ at 400 MHz, Figure S10: ^13^C NMR spectrum of **2** in CDCl_3_ at 100 MHz, Figure S11: HSQC spectrum of **2** in CDCl_3_, Figure S12: COSY spectrum of **2** in CDCl_3_, Figure S13: HMBC spectrum of **2** in CDCl_3_, Figure S14: NOESY spectrum of **2** in CDCl_3_, Figure S15: HRESIMS spectrum of **3**, Figure S16: ^1^H NMR spectrum of **3** in CDCl_3_ at 500 MHz, Figure S17: ^13^C NMR spectrum of **3** in CDCl_3_ at 125 MHz, Figure S18: HSQC spectrum of **3** in CDCl_3_, Figure S19: COSY spectrum of **3** in CDCl_3_, Figure S20: HMBC spectrum of **3** in CDCl_3_, Figure S21: NOESY spectrum of **3** in CDCl_3_, Figure S22: HRESIMS spectrum of **4**, Figure S23: ^1^H NMR spectrum of **4** in CDCl_3_ at 500 MHz, Figure S24: ^13^C NMR spectrum of **4** in CDCl_3_ at 125 MHz, Figure S25: HSQC spectrum of **4** in CDCl_3_, Figure S26: COSY spectrum of **4** in CDCl_3_, Figure S27: HMBC spectrum of **4** in CDCl_3_, Figure S28: NOESY spectrum of **4** in CDCl_3_, Figure S29: HRESIMS spectrum of **5**, Figure S30: ^1^H NMR spectrum of **5** in CDCl_3_ at 500 MHz, Figure S31: ^13^C NMR spectrum of **5** in CDCl_3_ at 125 MHz, Figure S32: HSQC spectrum of **5** in CDCl_3_, Figure S33: COSY spectrum of **5** in CDCl_3_, Figure S34: HMBC spectrum of **5** in CDCl_3_, Figure S35: NOESY spectrum of **5** in CDCl_3_, Figure S36: HRESIMS spectrum of **6**, Figure S37: ^1^H NMR spectrum of **6** CDCl_3_ at 500 MHz, Figure S38: ^13^C NMR spectrum of **6** in CDCl_3_ at 125 MHz, Figure S39: HSQC spectrum of **6** in CDCl_3_, Figure S40: COSY spectrum of **6** in CDCl_3_, Figure S41: HMBC spectrum of **6** in CDCl_3_, Figure S42: NOESY spectrum of **6** in CDCl_3_, Figure S43: HRESIMS spectrum of 7, Figure S44: ^1^H NMR spectrum of **7** in CDCl_3_ at 500 MHz, Figure S45: ^13^C NMR spectrum of **7** in CDCl_3_ at 125 MHz, Figure S46: HSQC spectrum of **7** in CDCl_3_, Figure S47: COSY spectrum of **7** in CDCl_3_, Figure S48: HMBC spectrum of **7** in CDCl_3_, Figure S49: NOESY spectrum of **7** in CDCl_3_, Figure S50: HRESIMS spectrum of **8**, Figure S51: ^1^H NMR spectrum of **8** in CDCl_3_ at 500 MHz, Figure S52: ^13^C NMR spectrum of **8** in CDCl_3_ at 125 MHz, Figure S53**:** HSQC spectrum of **8** in CDCl_3_, Figure S54: COSY spectrum of **8** in CDCl_3_, Figure S55: HMBC spectrum of **8** in CDCl_3_, Figure S56: NOESY spectrum of **8** in CDCl_3_, Figure S57: HRESIMS spectrum of **9**, Figure S58: ^1^H NMR spectrum of **9** in CDCl_3_ at 500 MHz, Figure S59: ^13^C NMR spectrum of **9** in CDCl_3_ at 125 MHz, Figure S60: HSQC spectrum of **9** in CDCl_3_, Figure S61: COSY spectrum of **9** in CDCl_3_, Figure S62: HMBC spectrum of **9** in CDCl_3_, Figure S63: NOESY spectrum of **9** in CDCl_3_, Figure S64: HRESIMS spectrum of **10**, Figure S65: ^1^H NMR spectrum of **10** in CDCl_3_ at 500 MHz, Figure S66: ^13^C NMR spectrum of **10** in CDCl_3_ at 125 MHz, Figure S67: HSQC spectrum of **10** in CDCl_3_, Figure S68: COSY spectrum of **10** in CDCl_3_, Figure S69: HMBC spectrum of **10** in CDCl_3_, Figure S70: NOESY spectrum of **10** in CDCl_3_, Figure S71: HRESIMS spectrum of **11**, Figure S72: ^1^H NMR spectrum of **11** in CDCl_3_ at 500 MHz, Figure S73: ^13^C NMR spectrum of **11** in CDCl_3_ at 125 MHz, Figure S74: HSQC spectrum of **11** in CDCl_3_, Figure S75: COSY spectrum of **1****1** in CDCl_3_, Figure S76: HMBC spectrum of **1****1** in CDCl_3_, Figure S77: NOESY spectrum of **11** in CDCl_3_, Figure S78: HRESIMS spectrum of **12**, Figure S79: ^1^H NMR spectrum of **12** in CDCl_3_ at 500 MHz, Figure S80: ^13^C NMR spectrum of **12** in CDCl_3_ at 125 MHz, Figure S81: HSQC spectrum of **12** in CDCl_3_, Figure S82: COSY spectrum of **12** in CDCl_3_, Figure S83: HMBC spectrum of **12** in CDCl_3_, Figure S84: NOESY spectrum of **12** in CDCl_3_, Figure S85: HRESIMS spectrum of **13**, Figure S86: ^1^H NMR spectrum of **13** in CDCl_3_ at 400 MHz, Figure S87: ^13^C NMR spectrum of **13** in CDCl_3_ at 100 MHz, Figure S88: HSQC spectrum of **13** in CDCl_3_, Figure S89: COSY spectrum of **13** in CDCl_3_, Figure S90: HMBC spectrum of **13** in CDCl_3_, Figure S91: NOESY spectrum of **13** in CDCl_3_, Figure S92: HRESIMS spectrum of **14**, Figure S93: ^1^H NMR spectrum of **14** in CDCl_3_ at 600 MHz, Figure S94: ^13^C NMR spectrum of **14** in CDCl_3_ at 150 MHz, Figure S95: HSQC spectrum of **14** in CDCl_3_, Figure S96: COSY spectrum of **14** in CDCl_3_, Figure S97: HMBC spectrum of **14** in CDCl_3_, Figure S98: NOESY spectrum of **14** in CDCl_3_, Figure S99: HRESIMS spectrum of **15**, Figure S100: ^1^H NMR spectrum of **15** in CDCl_3_ at 500 MHz, Figure S101: ^13^C NMR spectrum of **15** in CDCl_3_ at 125 MHz, Figure S102: HSQC spectrum of **15** in CDCl_3_, Figure S103: COSY spectrum of **15** in CDCl_3_, Figure S104: HMBC spectrum of **15** in CDCl_3_, Figure S105: NOESY spectrum of **15** in CDCl_3_, Figure S106: HRESIMS spectrum of **16**, Figure S107: ^1^H NMR spectrum of **16** in CDCl_3_ at 600 MHz, Figure S108: ^13^C NMR spectrum of **16** in CDCl_3_ at 150 MHz, Figure S109: HSQC spectrum of **16** in CDCl_3_, Figure S110: COSY spectrum of **16** in CDCl_3_, Figure S111: HMBC spectrum of **16** in CDCl_3_, Figure S112: NOESY spectrum of **16** in CDCl_3_, Figure S113: MMFF lowest energy conformers for **1**, Figure S114: MMFF lowest energy conformers for **2**, Table S1: ^1^H and ^13^C NMR spectroscopic data of compounds **17** and **18**, Table S2: Energy analysis for MMFF conformational searching of compounds **1** and **2**.
