# Supplementary material for: Bioactive Diterpenes, Norditerpenes, and Sesquiterpenes from a Formosan Soft Coral Cespitularia sp
Source: Pharmaceuticals (Basel). 2021 Dec 1;14(12):1252. doi: 10.3390/ph14121252 (PMC8708085; doi:10.3390/ph14121252)
Supplement: Supplementary file 1 [file pharmaceuticals-14-01252-s001.zip › SP (Pharmaceuticals)-ces3-supplementary.pdf]

Bioactive Diterpenes, Norditerpenes, and Sesquiterpenes from a Formosan Soft Coral *Cespitularia* sp.

You-Cheng Lin [1], Chi-Chien Lin [2], Yi-Chia Chu [2], Chung-Wei Fu [3], and Jyh-Horng Sheu [1,3,4,5,6,*]

1 Doctoral Degree Program in Marine Biotechnology, National Sun Yat-sen University, Kaohsiung 804, Taiwan ; d045620002@nsysu.edu.tw (Y.-C. L.)
2 Institute of Biomedical Science, National Chung-Hsing University, Taichung 402, Taiwan ; lincc@dragon.nchu.edu.tw (C.-C. L.); girl770409@smail.nchu.edu.tw (Y.-C. C.)
3 Department of Marine Biotechnology and Resources, National Sun Yat-sen University, Kaohsiung 804, Taiwan; m095020006@nsysu.edu.tw (C.-W. F.)
4 Department of Medical Research, China Medical University Hospital, China Medical University, Taichung 404, Taiwan
5 Graduate Institute of Natural Products, Kaohsiung Medical University, Kaohsiung 807, Taiwan
6 Frontier Center for Ocean Science and Technology, National Sun Yat-sen University, Kaohsiung 804, Taiwan

* Correspondence: sheu@mail.nsysu.edu.tw; Tel.: +886-7-5252-000 ext. 5030

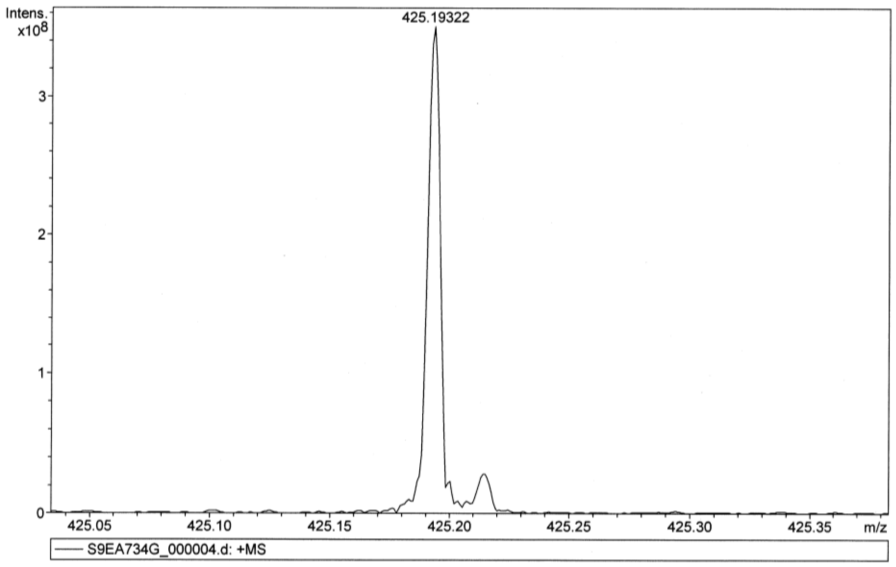

Meas. m/z	#	Formula	Score	m/z	err [mDa]	err [ppm]	mSigma	rdb	e⁻ Conf	N-Rule
425.19322	1	C 23 H 30 Na O 6	100.00	425.19346	0.24	0.56	5.5	8.5	even	ok

Figure S1. HRESIMS spectrum of **1**

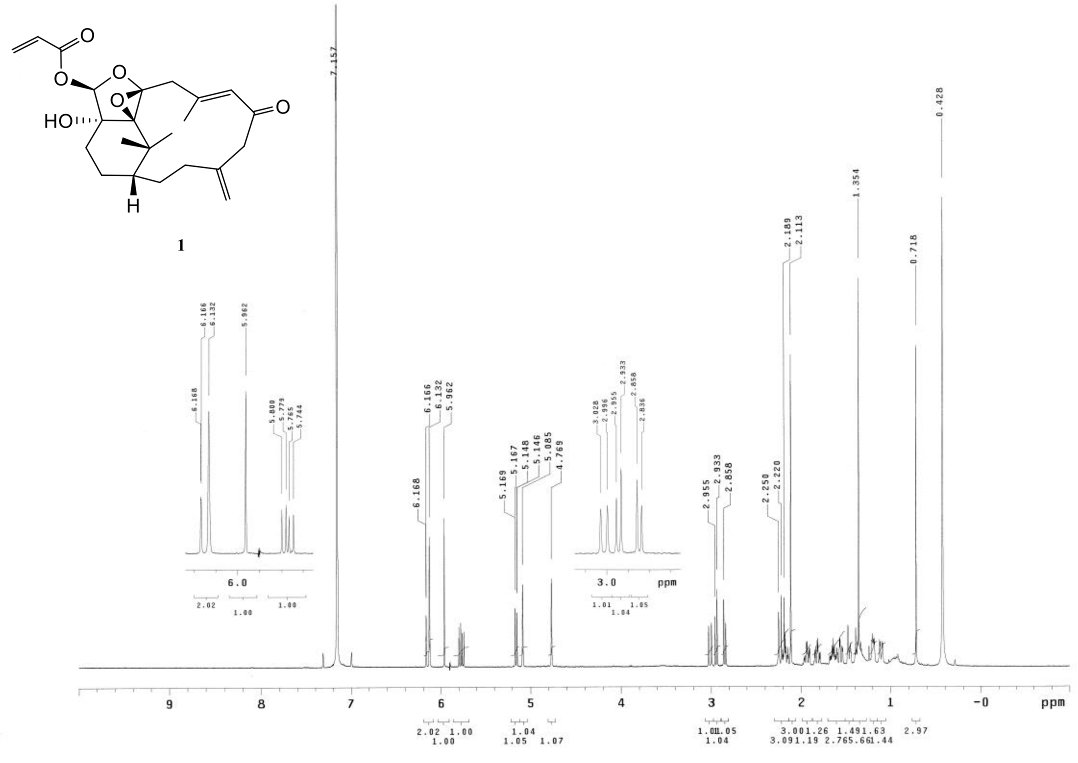

Figure S2. ^{1}H NMR spectrum of **1** in C_6D_6 at 500 MHz

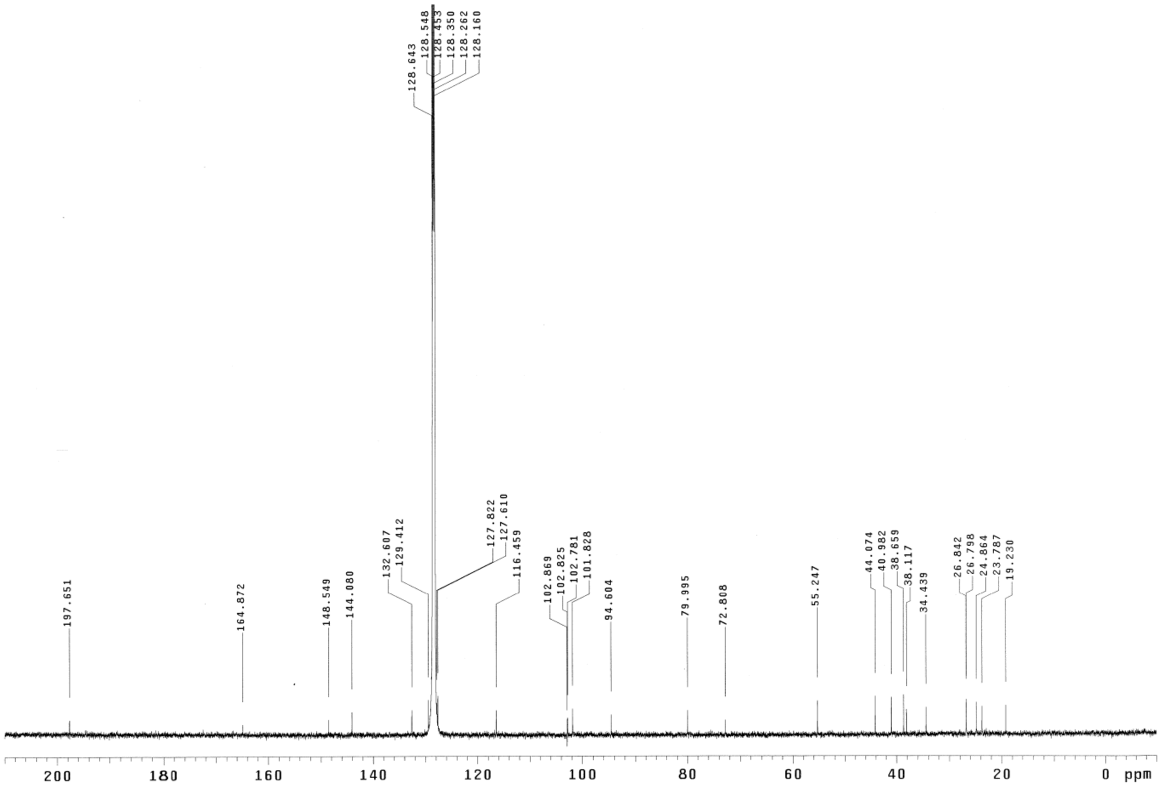

Figure S3. ^{13}C NMR spectrum of 1 in C_6D_6 at 125 MHz

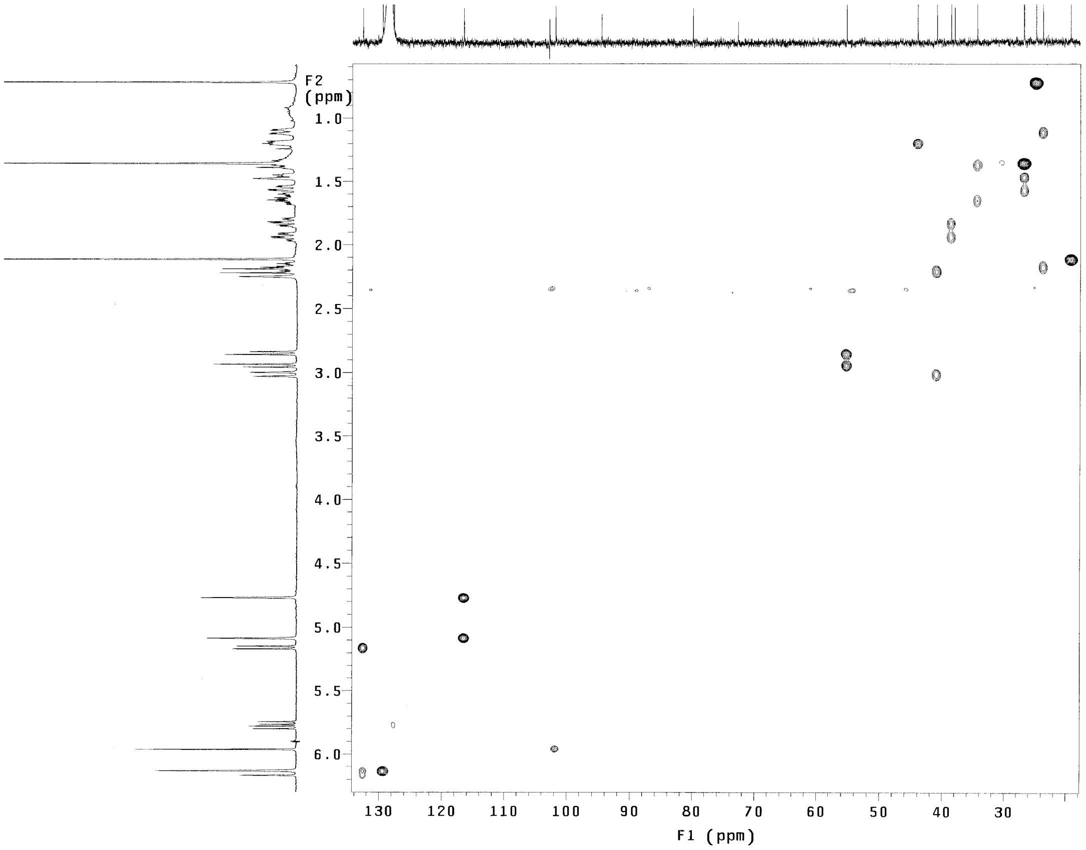

Figure S4. HSQC spectrum of **1** in C_6D_6

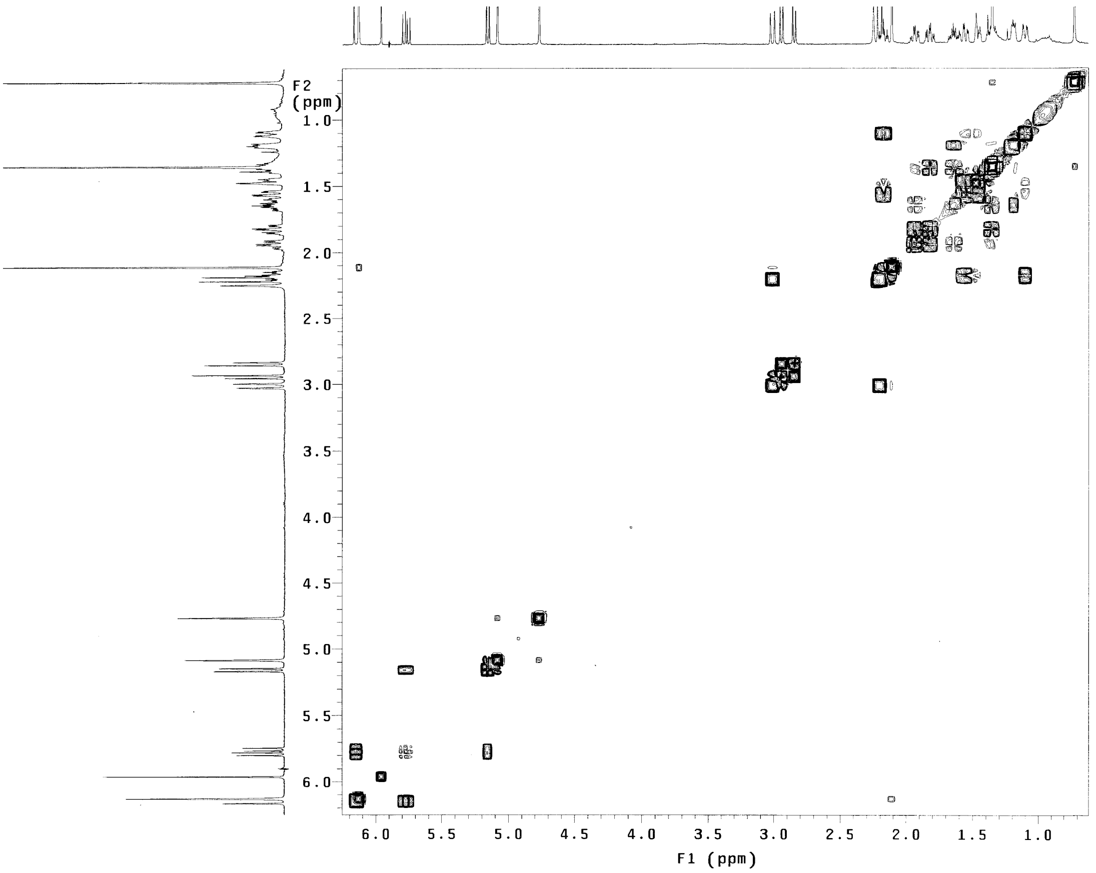

Figure S5. COSY spectrum of **1** in C_6D_6

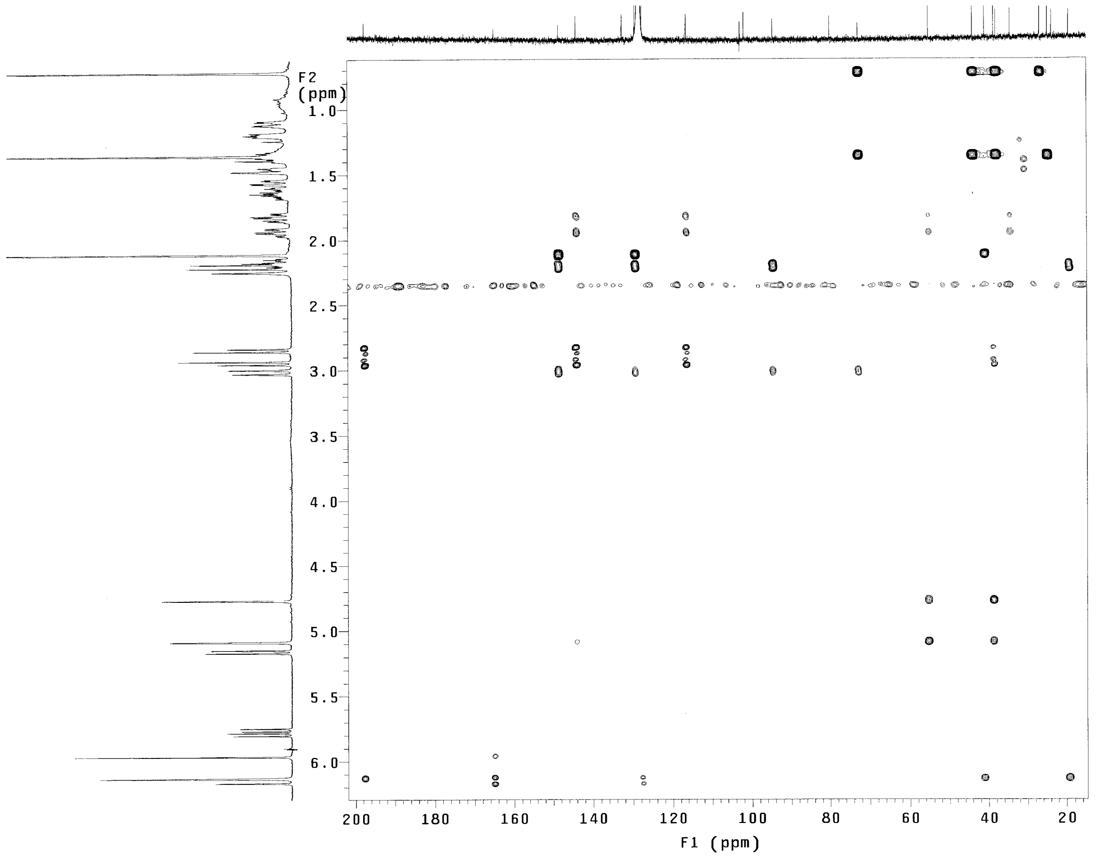

Figure S6. HMBC spectrum of **1** in C_6D_6

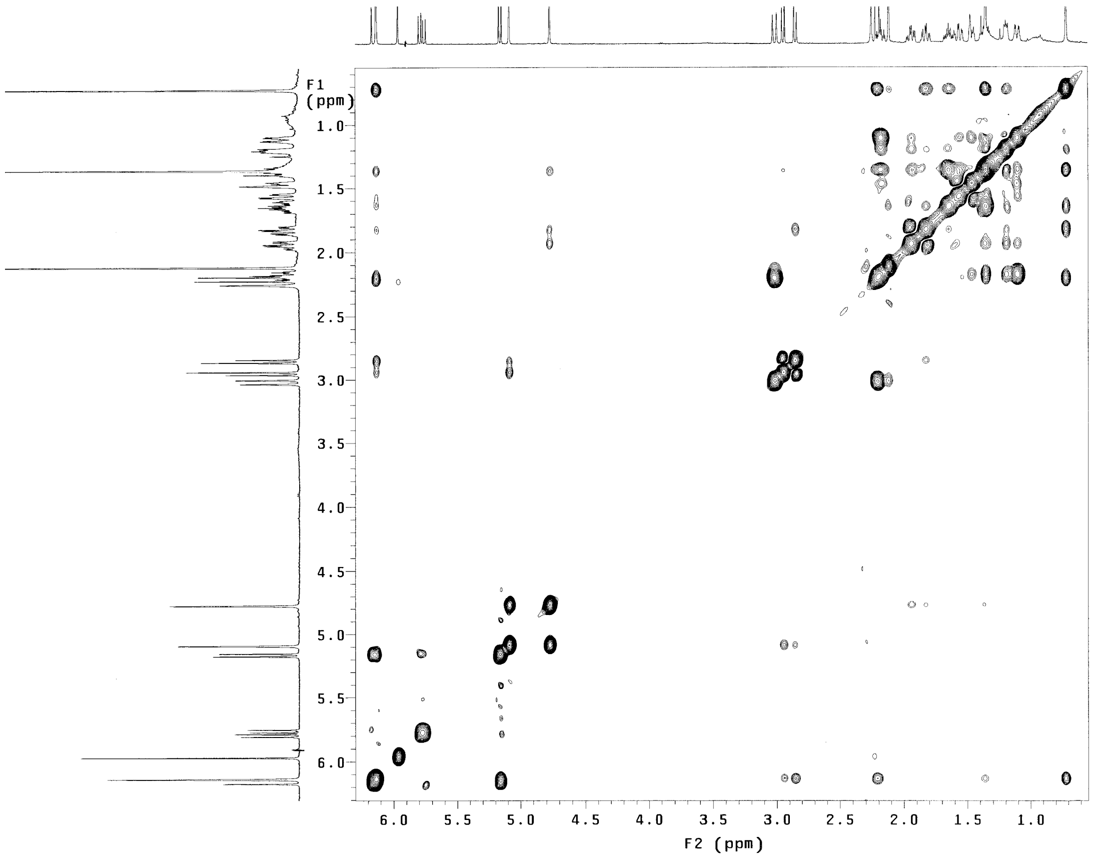

Figure S7. NOESY spectrum of **1** in C_6D_6

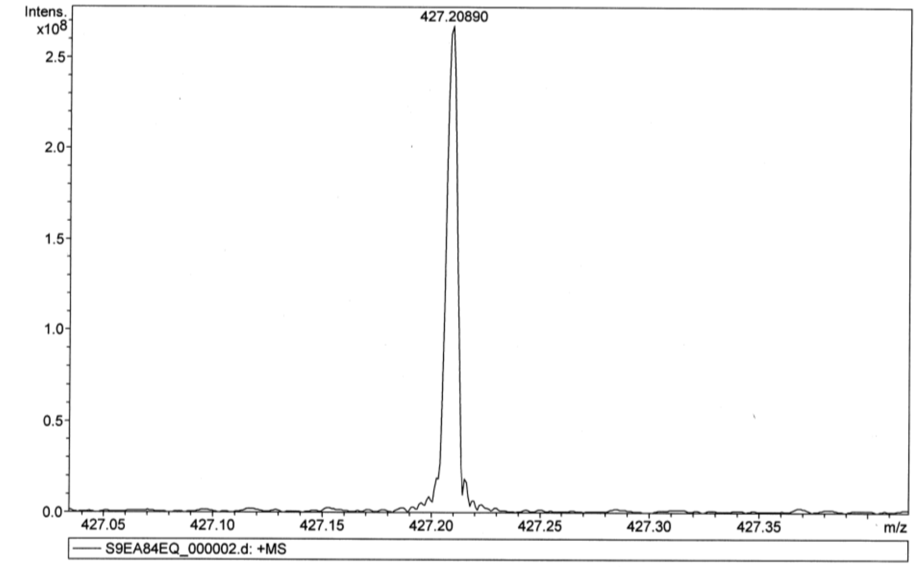

Meas. m/z	#	Formula	Score	m/z	err [mDa]	err [ppm]	mSigma	rdb	e⁻ Conf	N-Rule
427.20890	1	C 23 H 32 Na O 6	100.00	427.20911	0.21	0.50	9.8	7.5	even	ok

Figure S8. HRESIMS spectrum of **2**

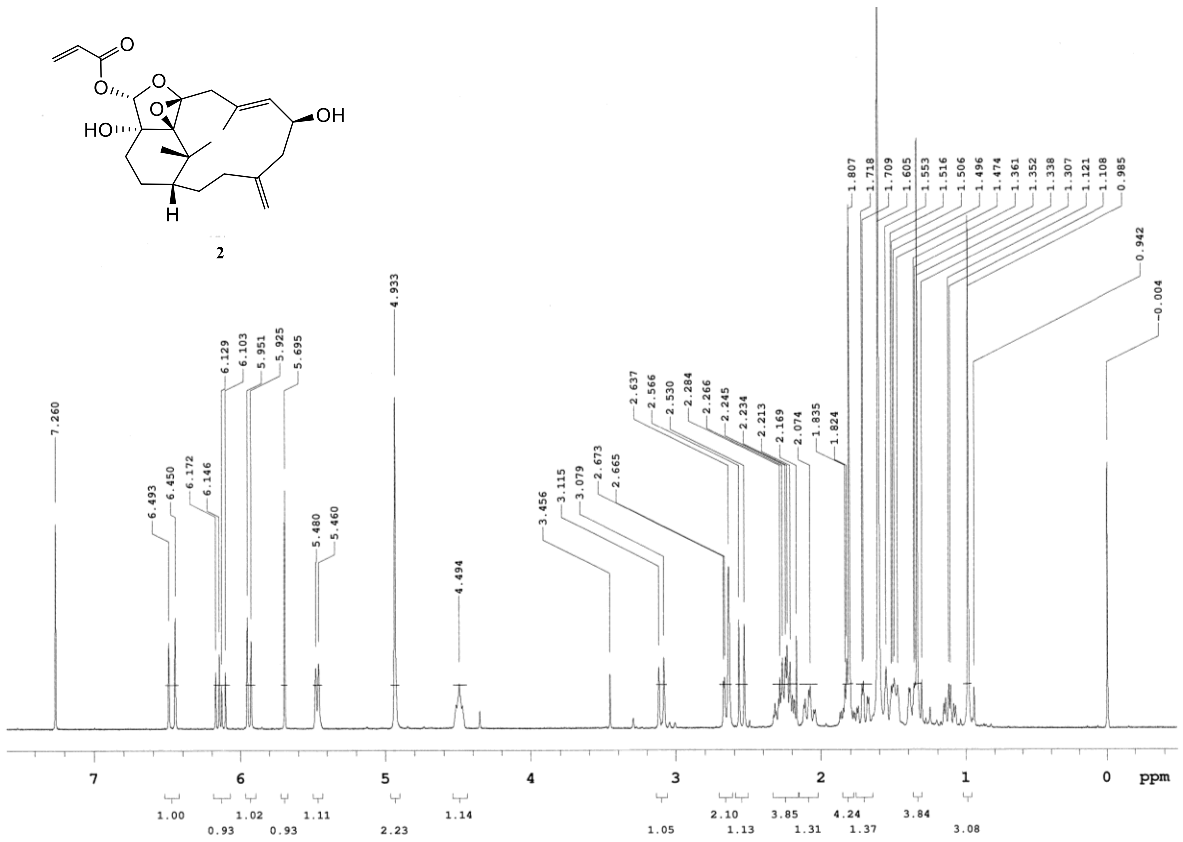

Figure S9. ^{1}H NMR spectrum of **2** in $CDCl_3$ at 400 MHz

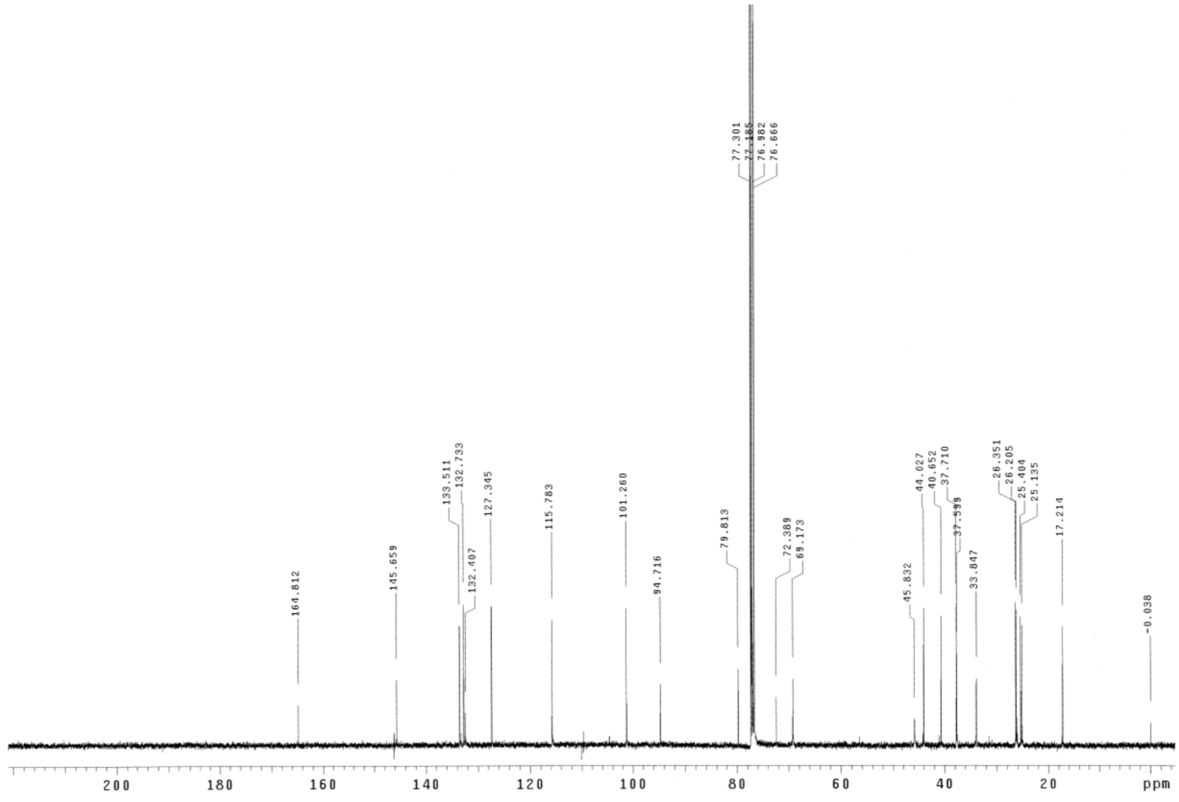

Figure S10. ^{13}C NMR spectrum of **2** in $CDCl_3$ at 100 MHz

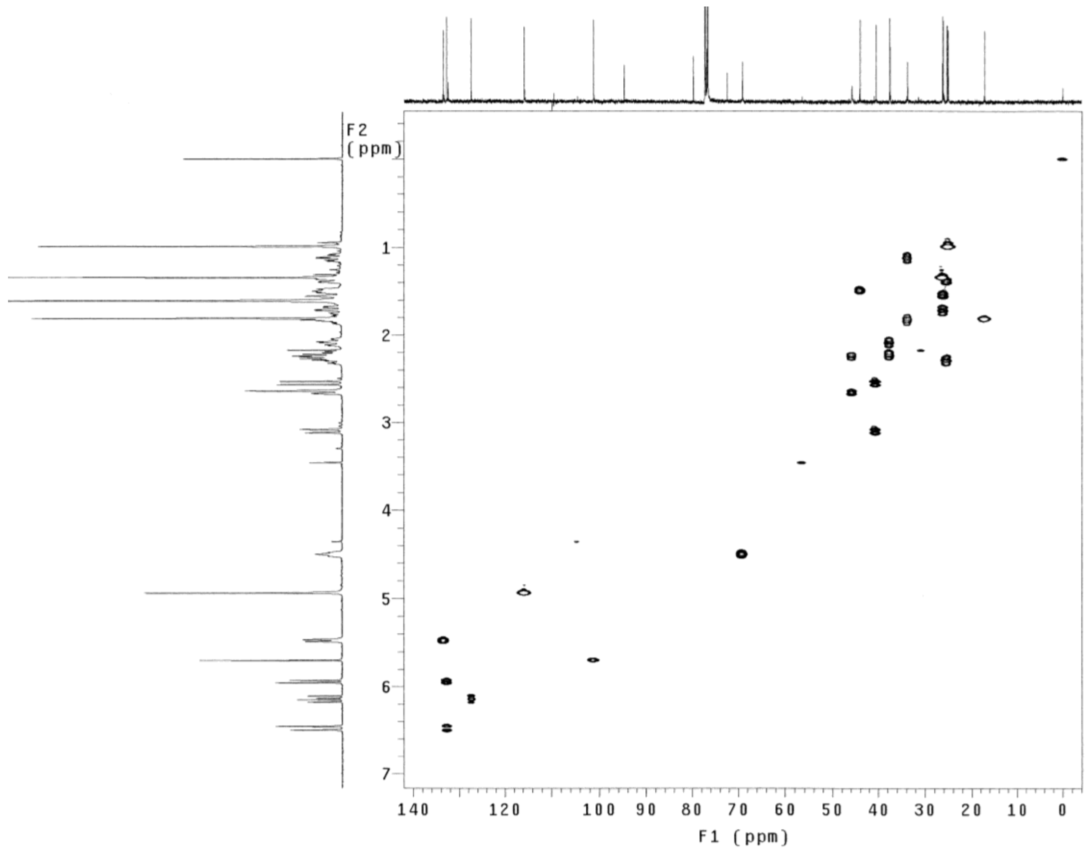

Figure S11. HSQC spectrum of **2** in $CDCl_3$

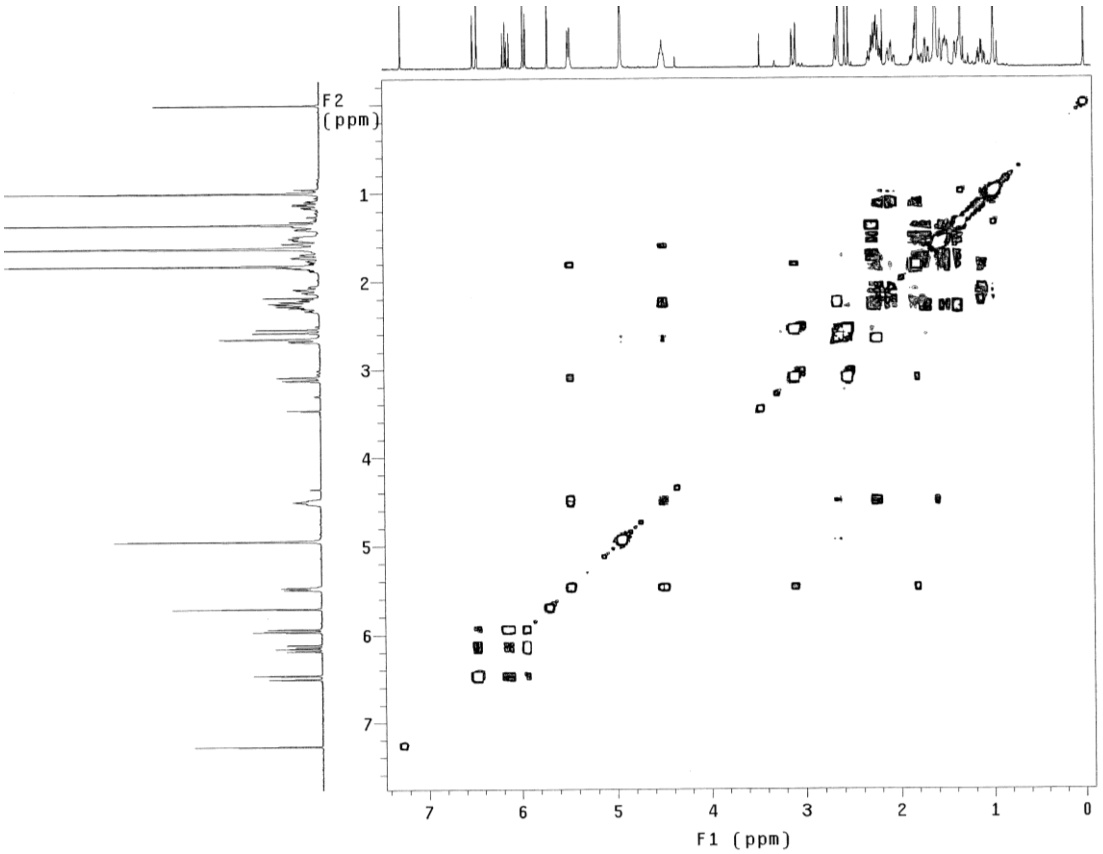

Figure S12. COSY spectrum of **2** in $CDCl_3$

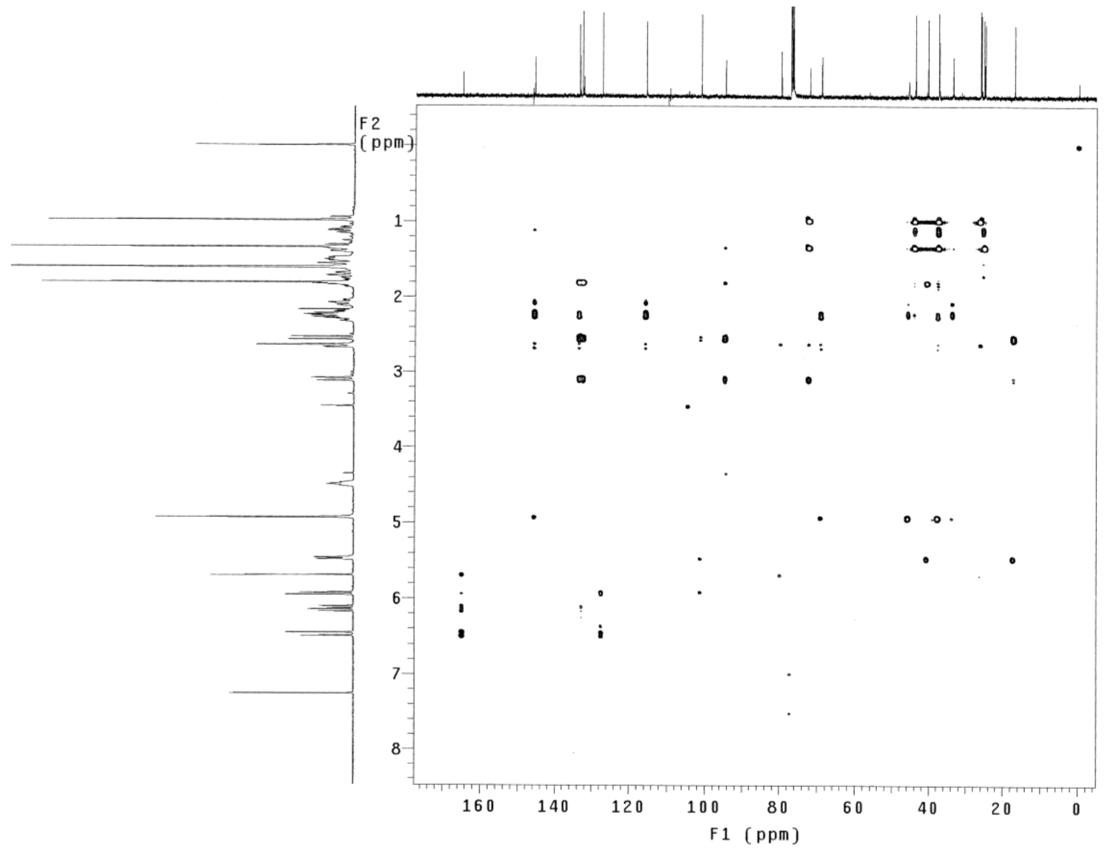

Figure S13. HMBC spectrum of **2** in $CDCl_3$

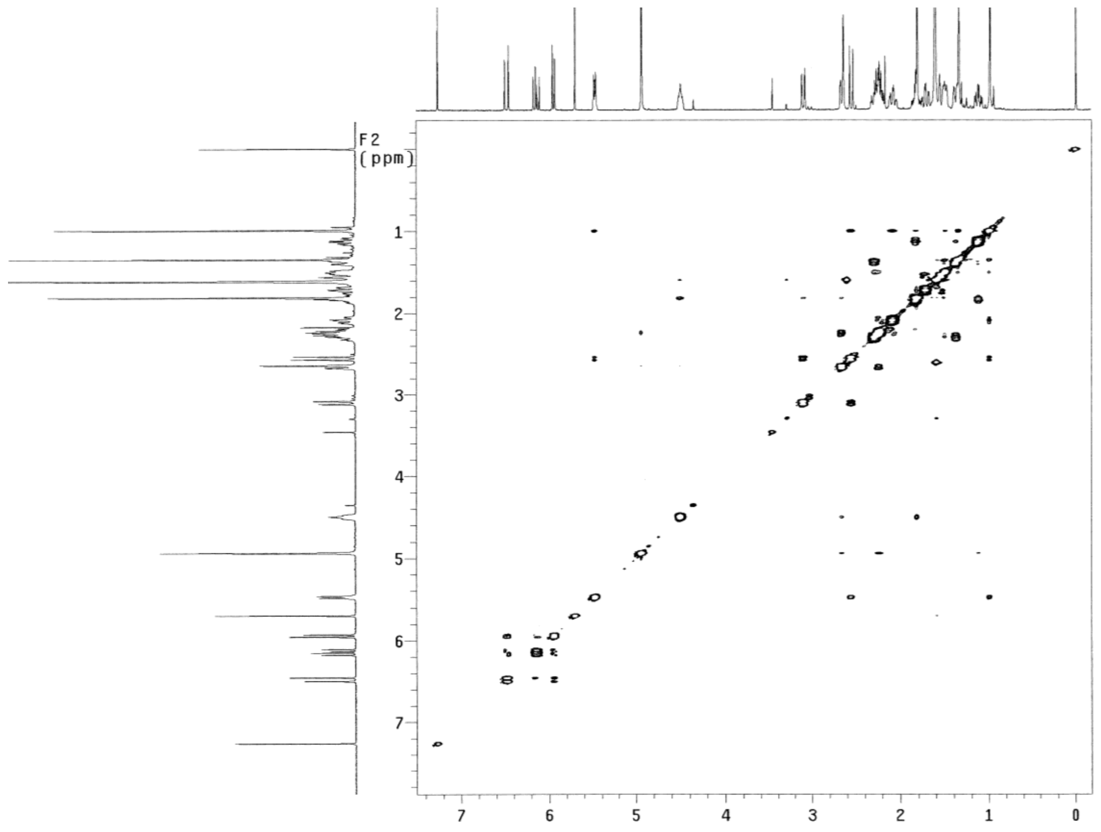

Figure S14. NOESY spectrum of **2** in $CDCl_3$

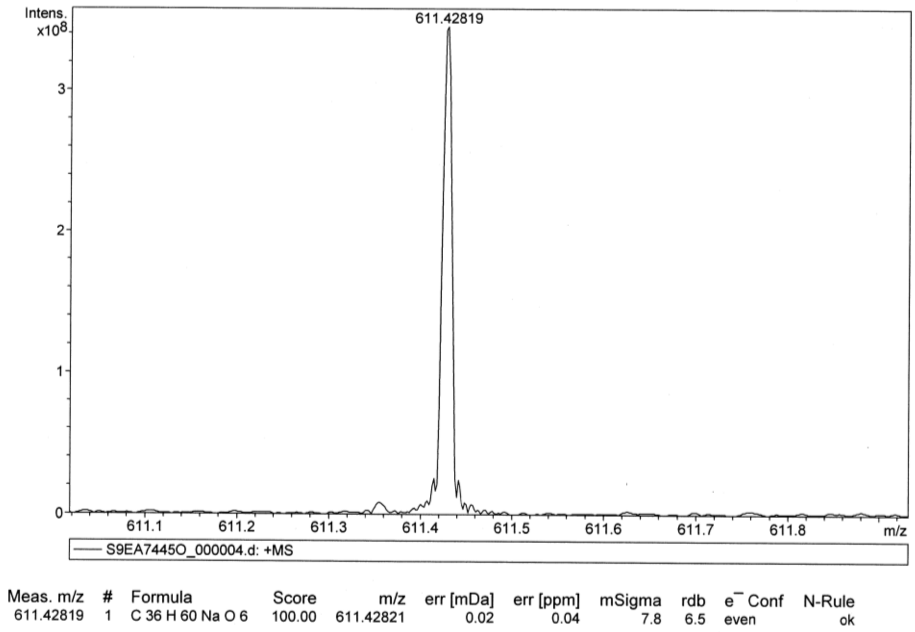

Figure S15. HRESIMS spectrum of **3**

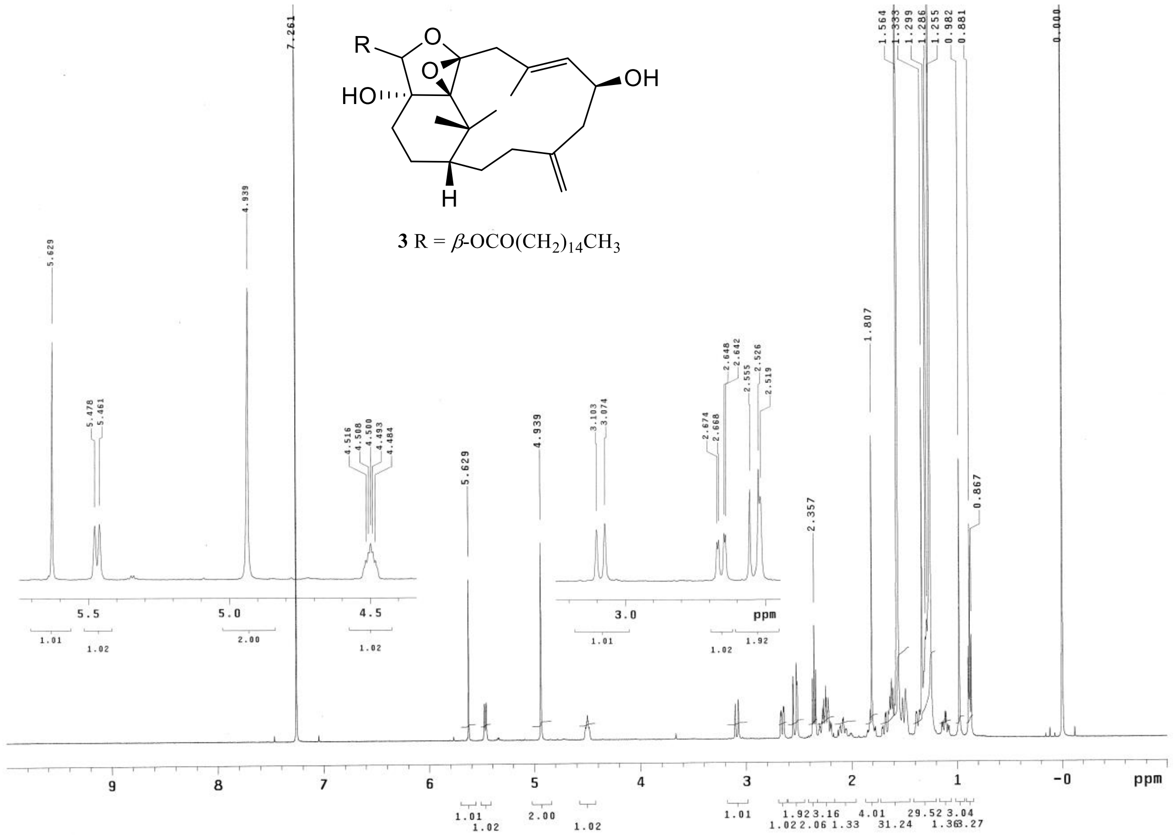

Figure S16. ^{1}H NMR spectrum of **3** in $CDCl_3$ at 500 MHz

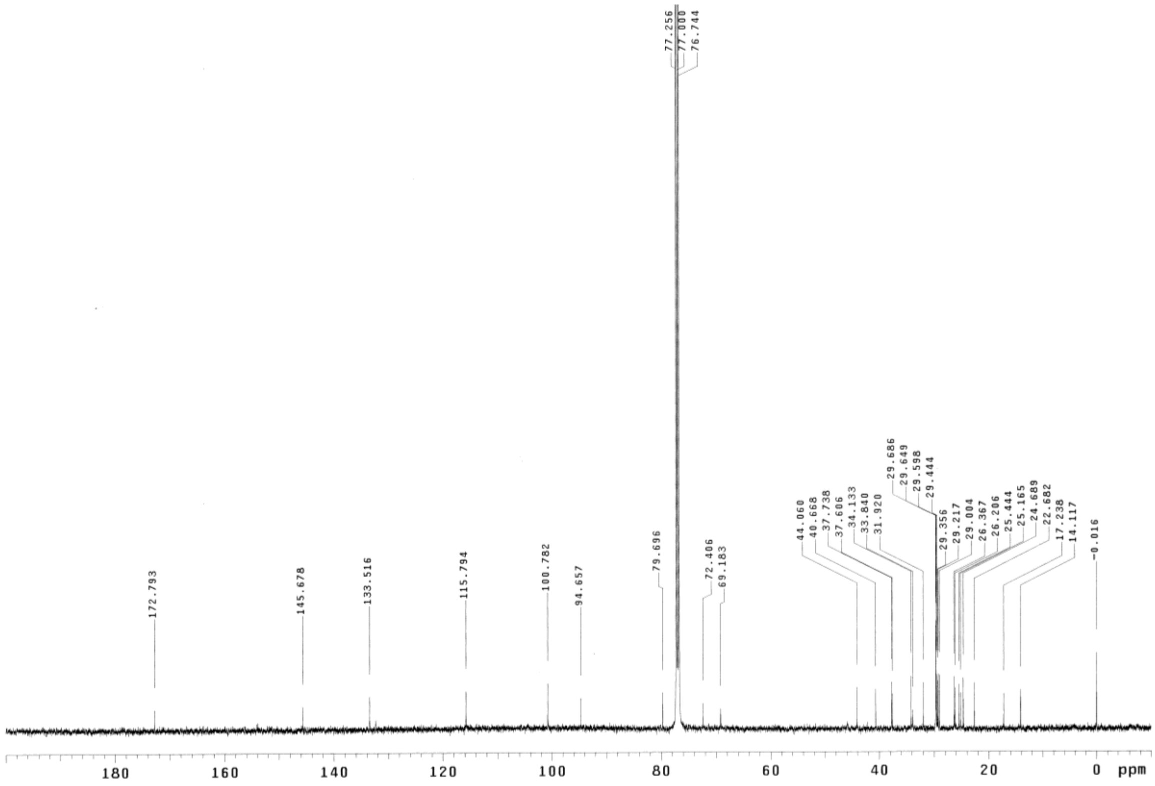

Figure S17. ^{13}C NMR spectrum of **3** in $CDCl_3$ at 125 MHz

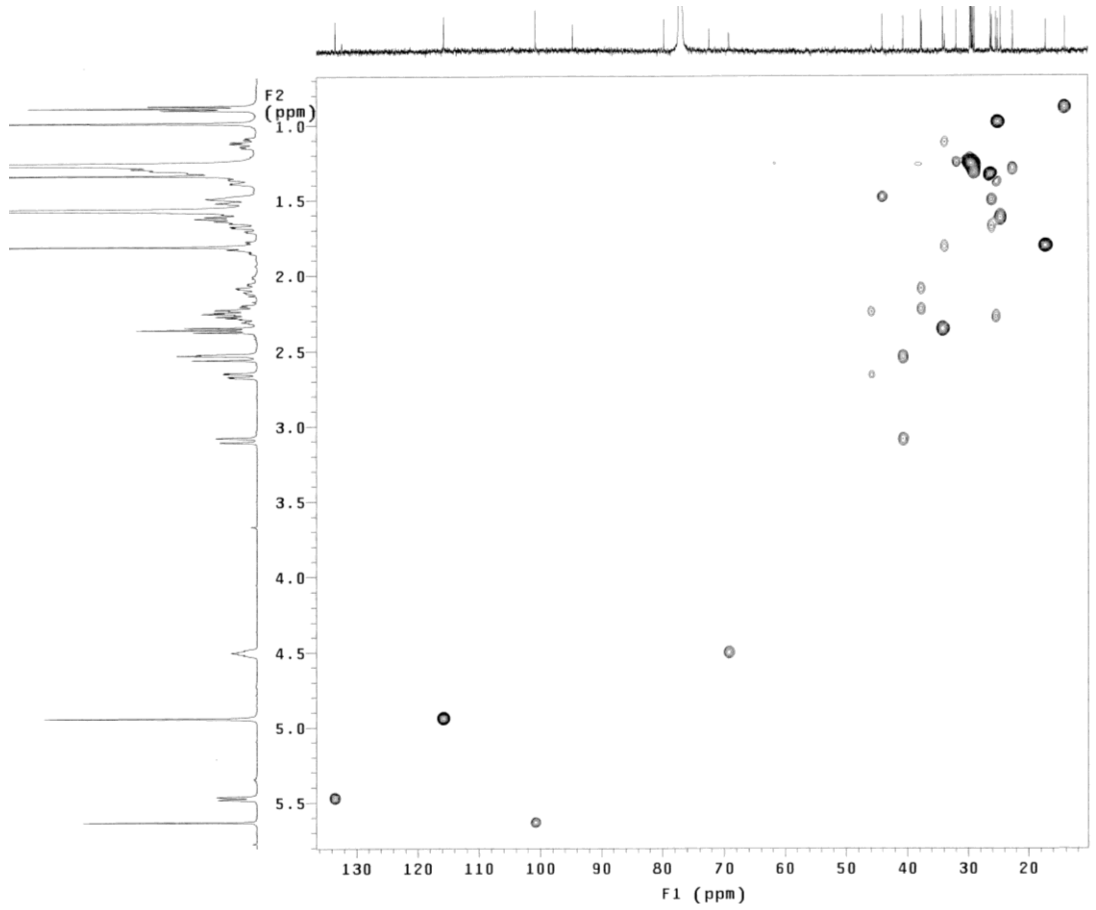

Figure S18. HSQC spectrum of **3** in $CDCl_3$

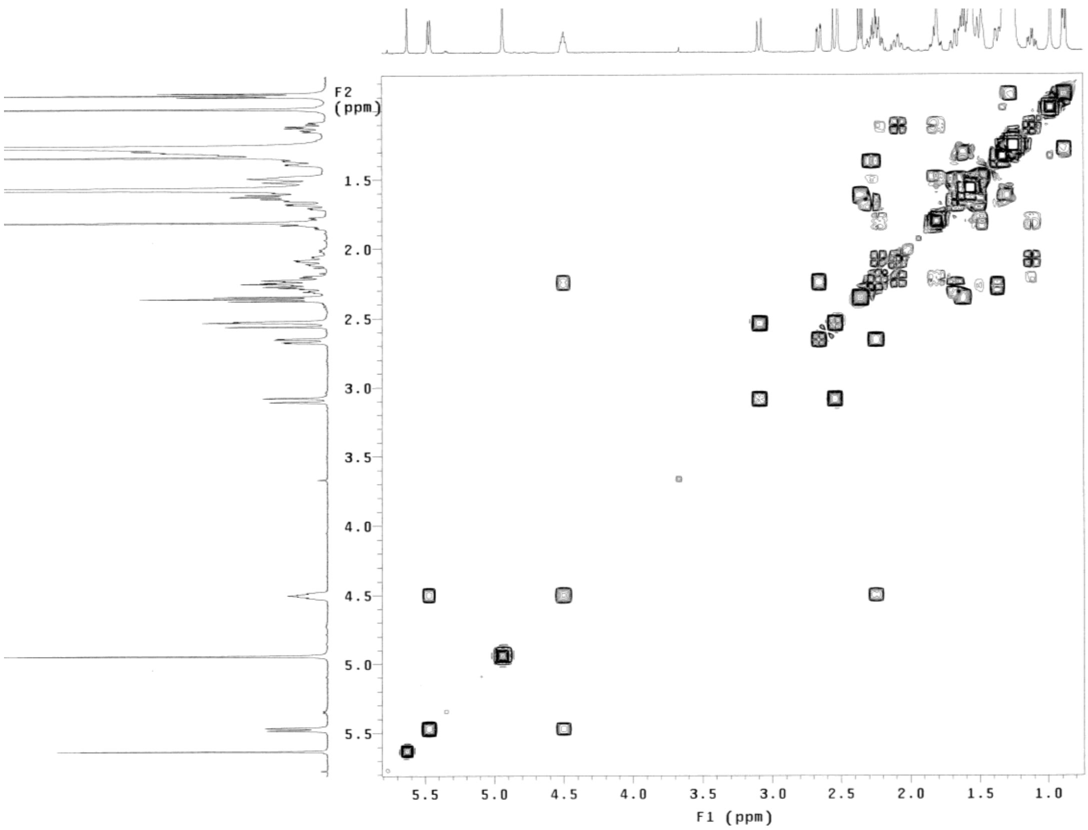

Figure S19. COSY spectrum of **3** in $CDCl_3$

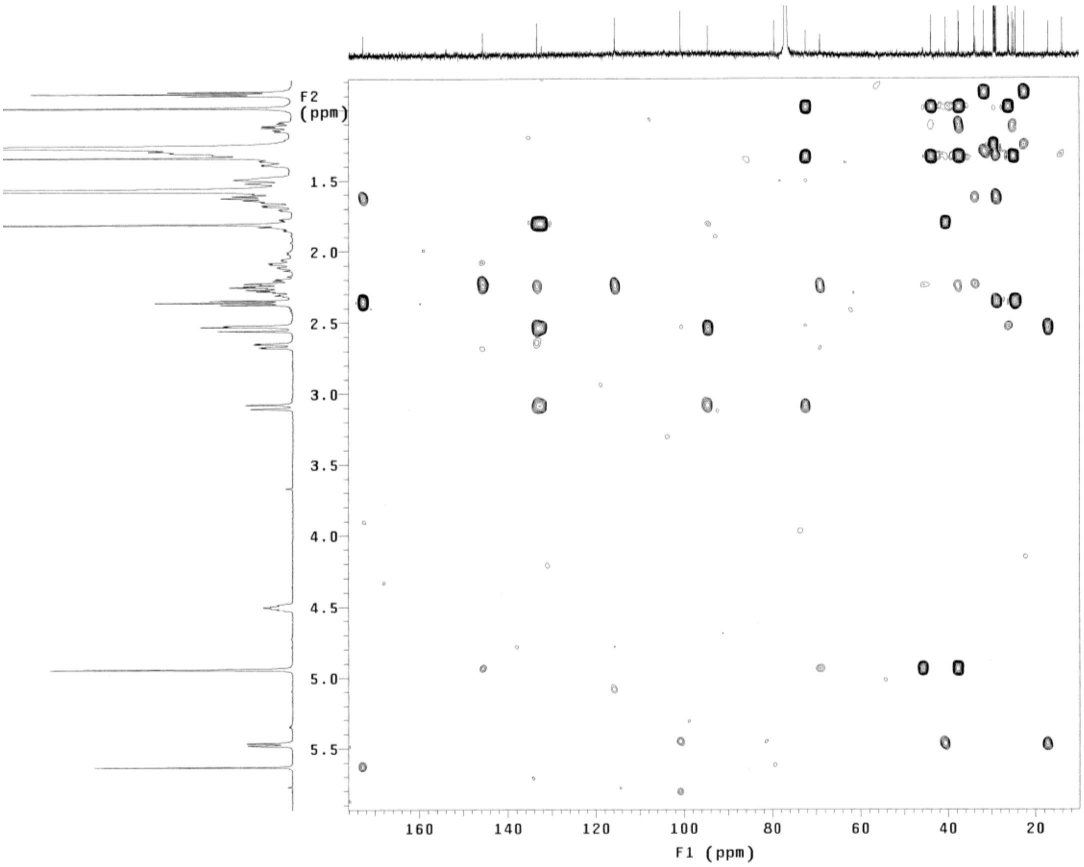

Figure S20. HMBC spectrum of **3** in $CDCl_3$

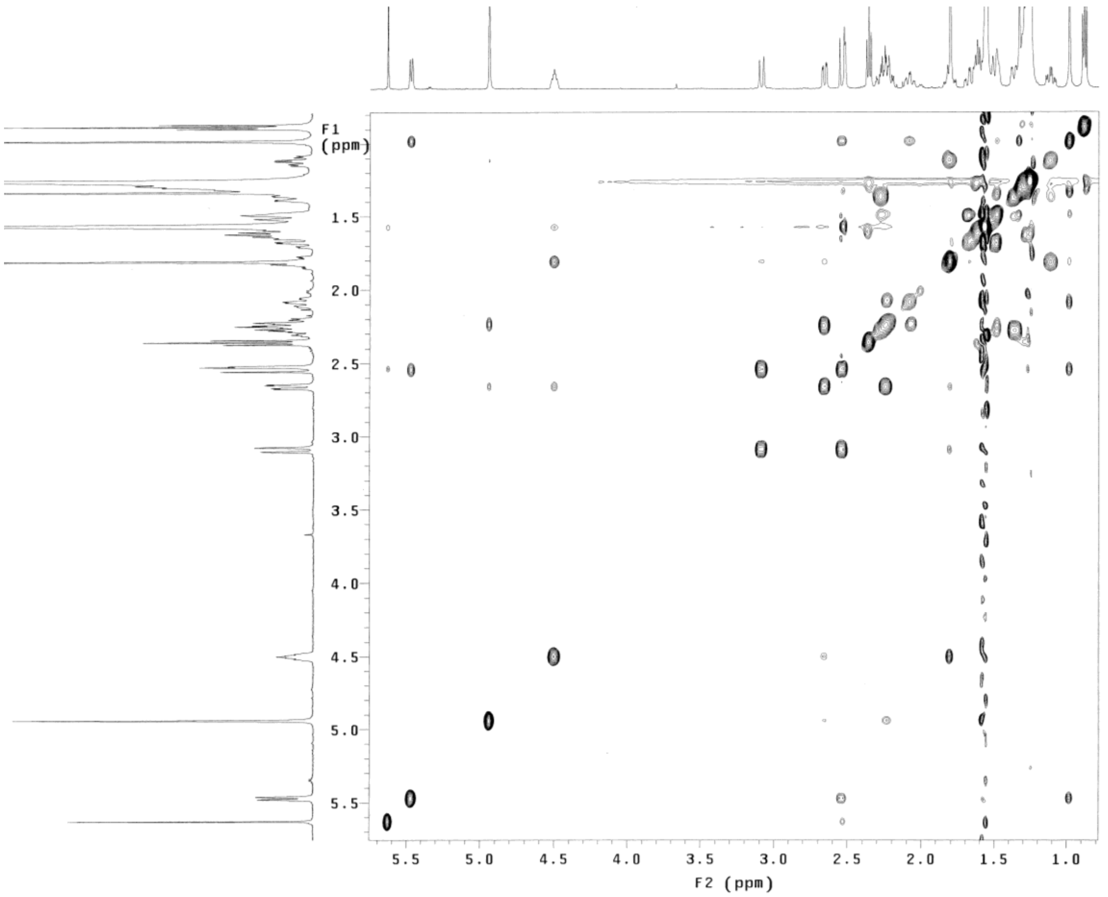

Figure S21. NOESY spectrum of **3** in $CDCl_3$

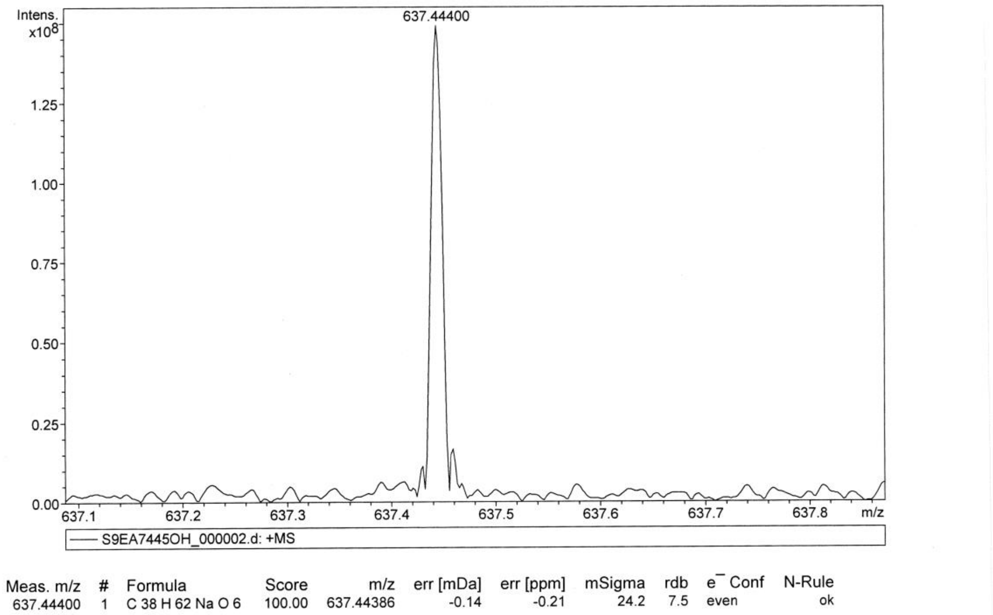

Figure S22. HRESIMS spectrum of **4**

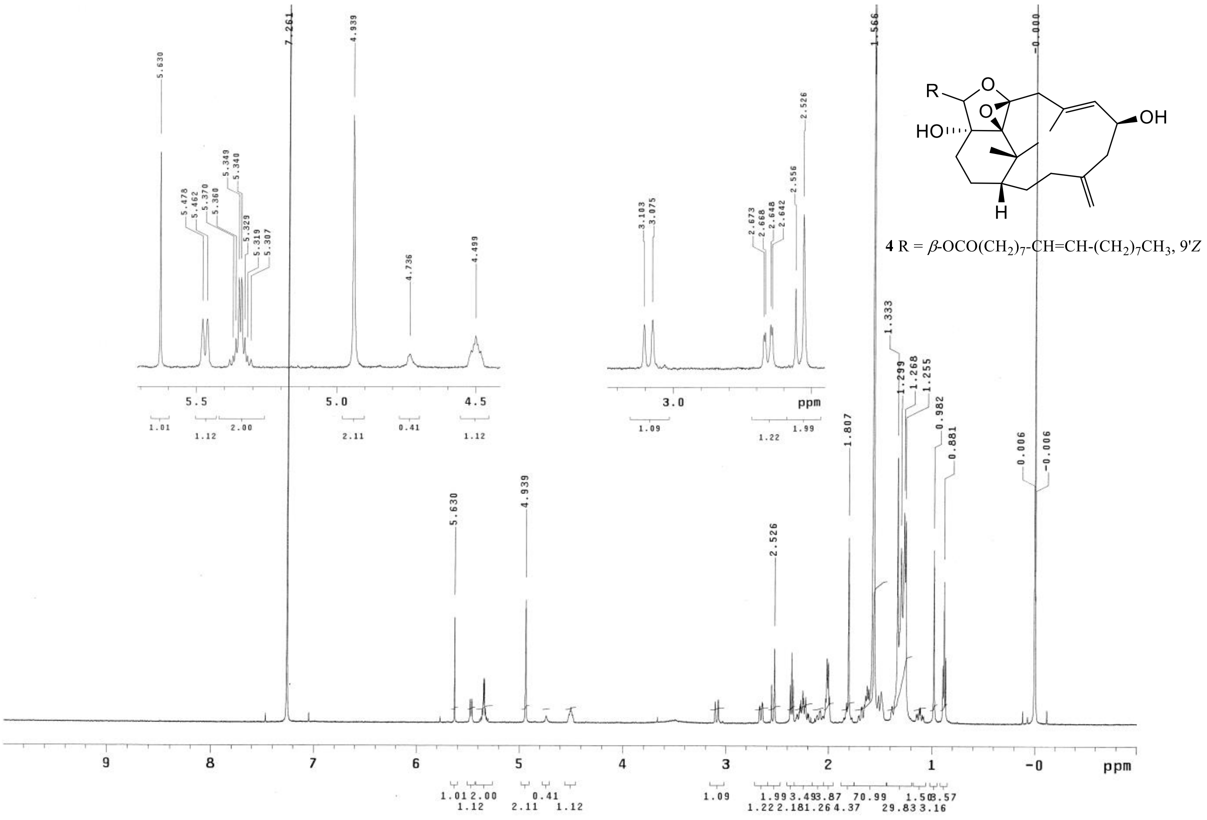

Figure S23. ^{1}H NMR spectrum of **4** in CDCl₃ at 500 MHz

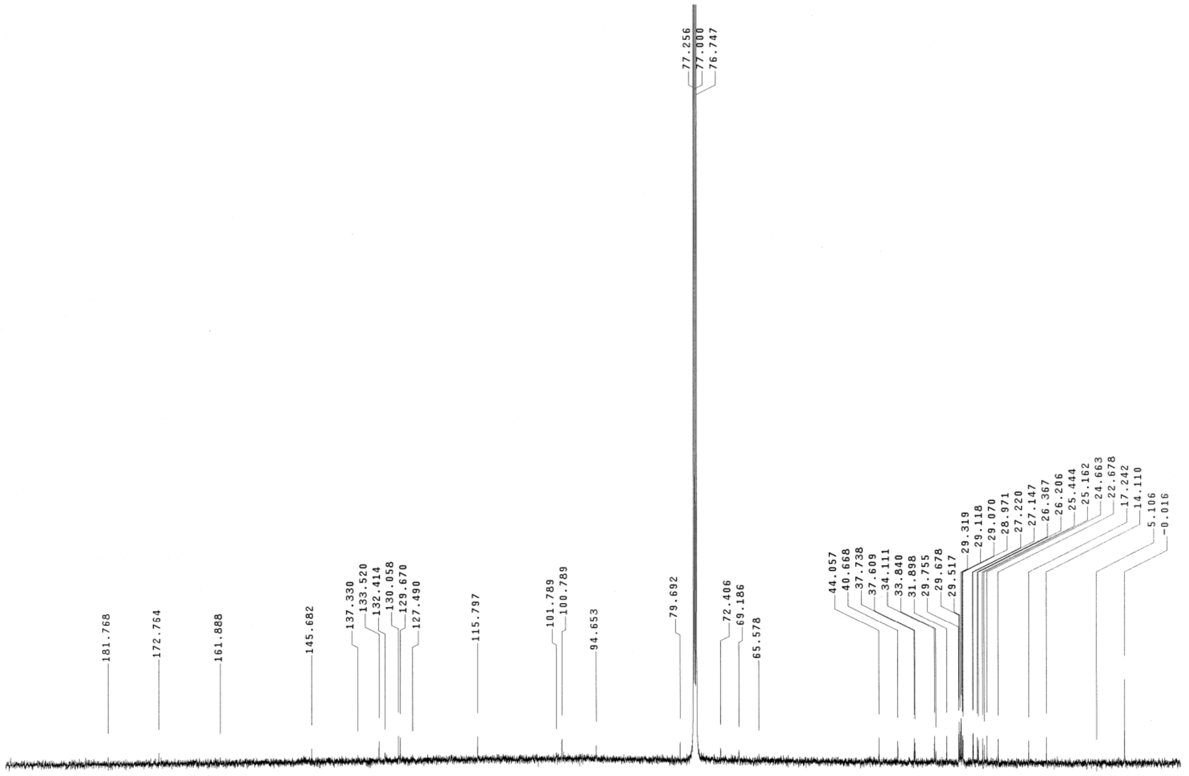

Figure S24. ^{13}C NMR spectrum of **4** in CDCl₃ at 125 MHz

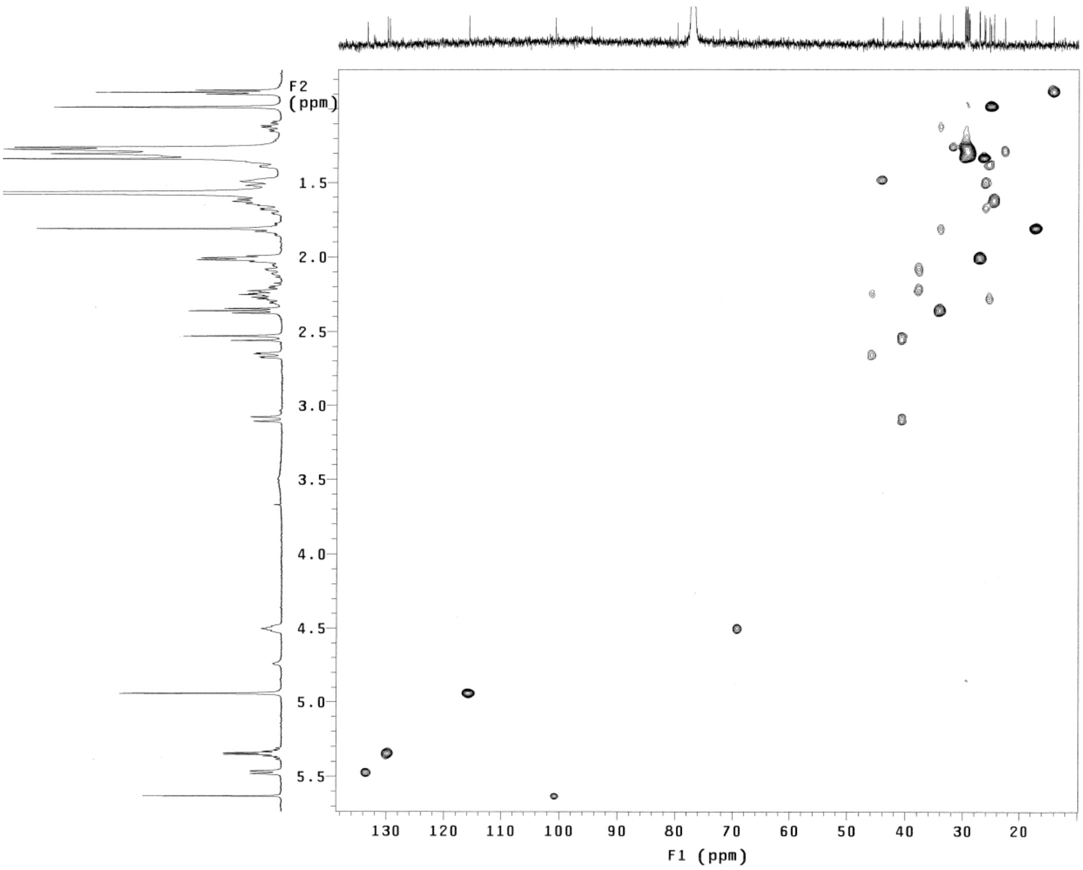

Figure S25. HSQC spectrum of **4** in $CDCl_3$

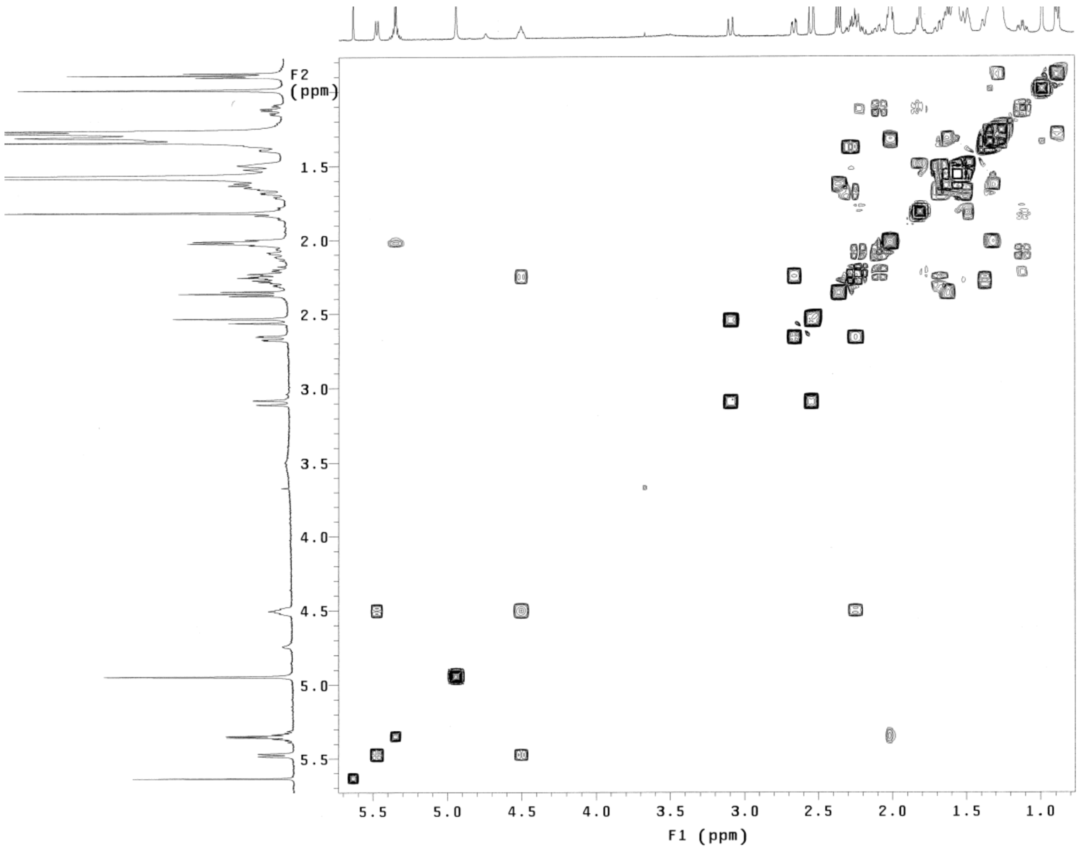

Figure S26. COSY spectrum of **4** in $CDCl_3$

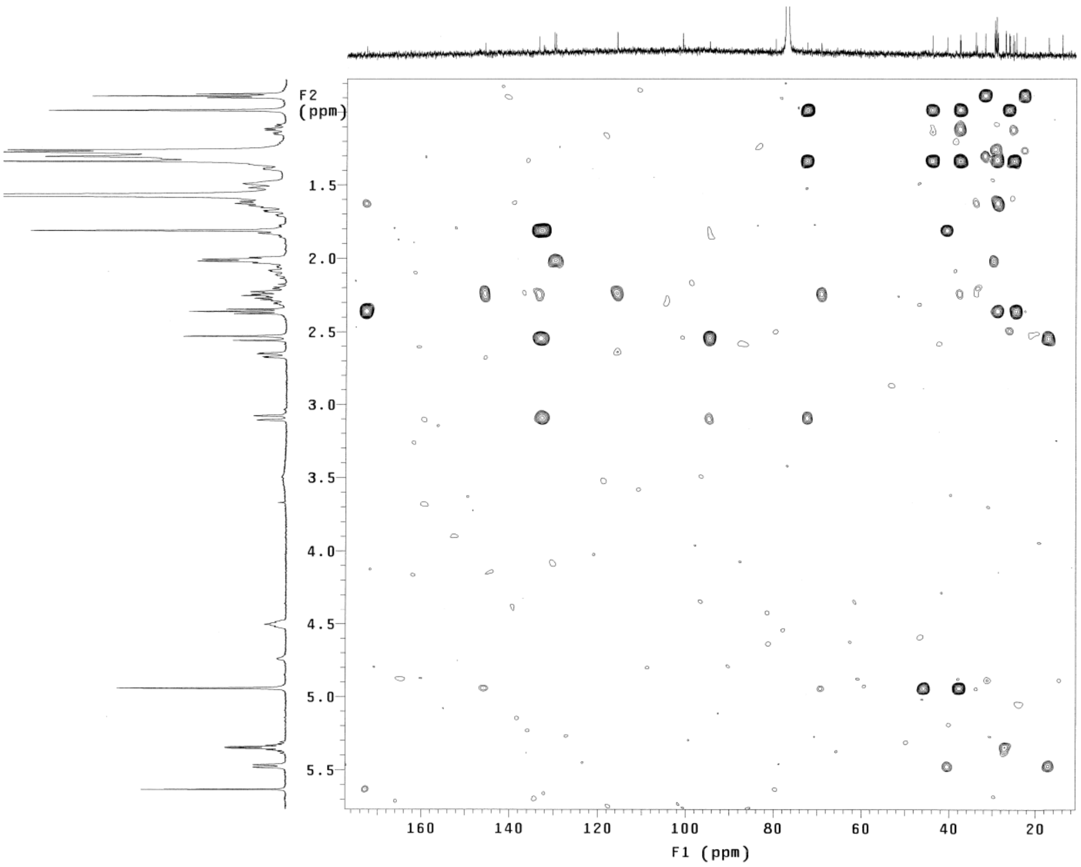

Figure S27. HMBC spectrum of **4** in $CDCl_3$

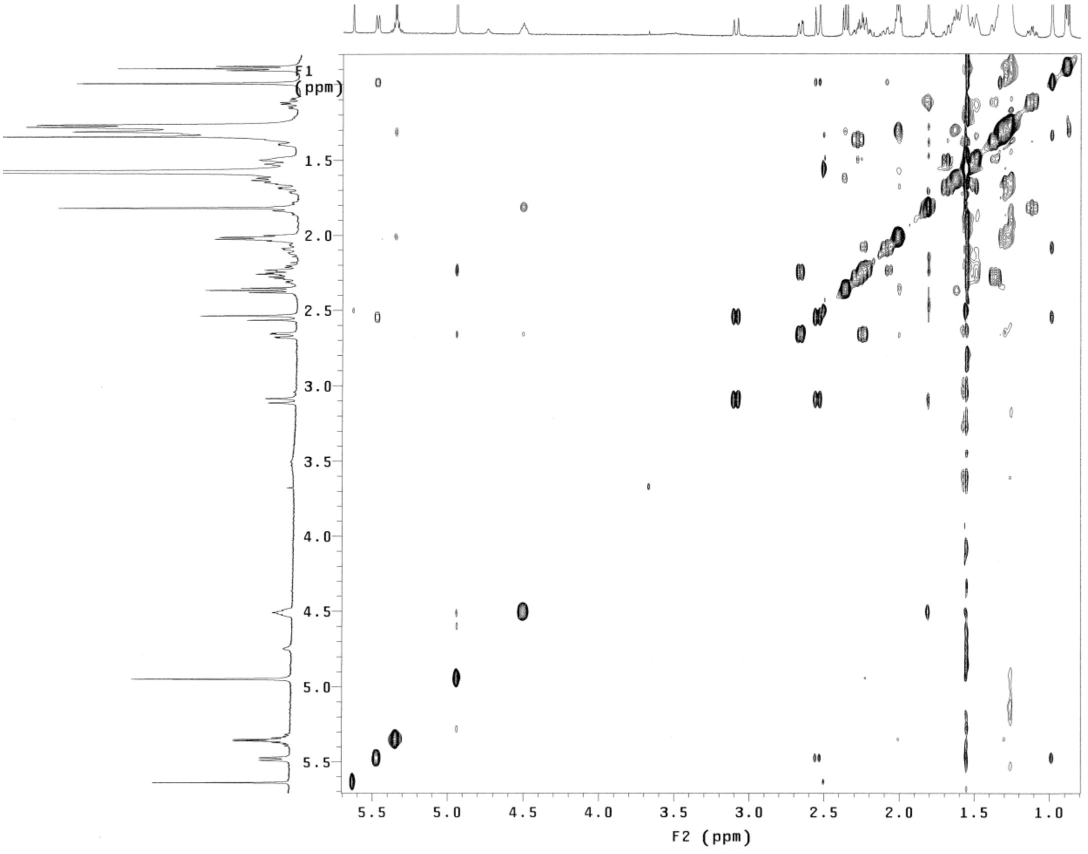

Figure S28. NOESY spectrum of **4** in $CDCl_3$

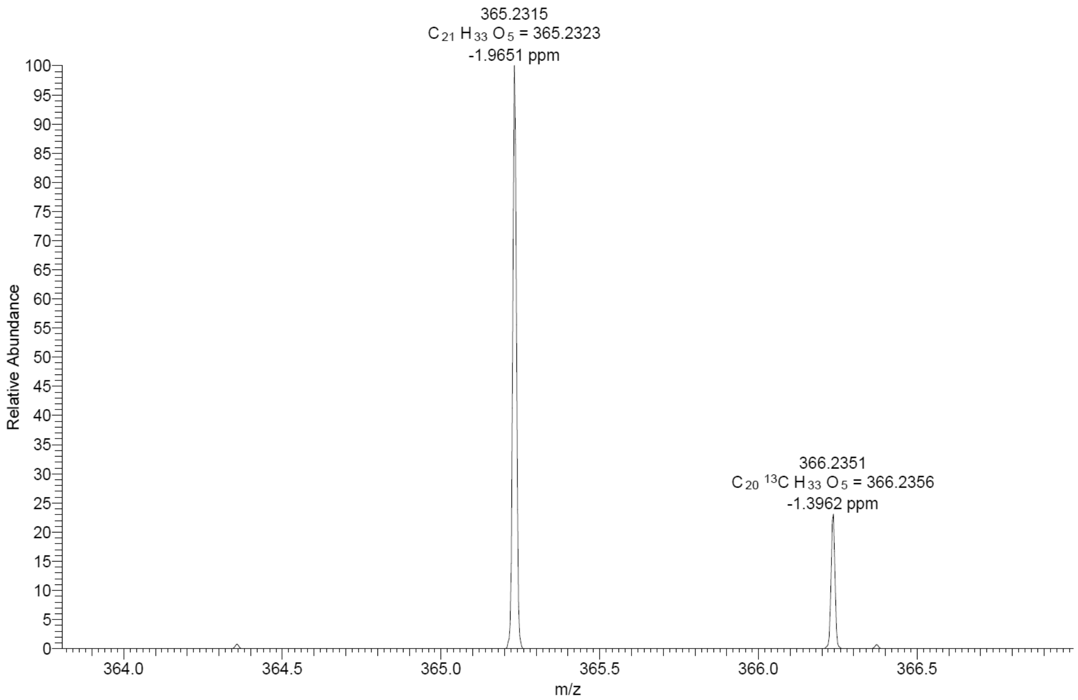

Figure S29. HRESIMS spectrum of **5**

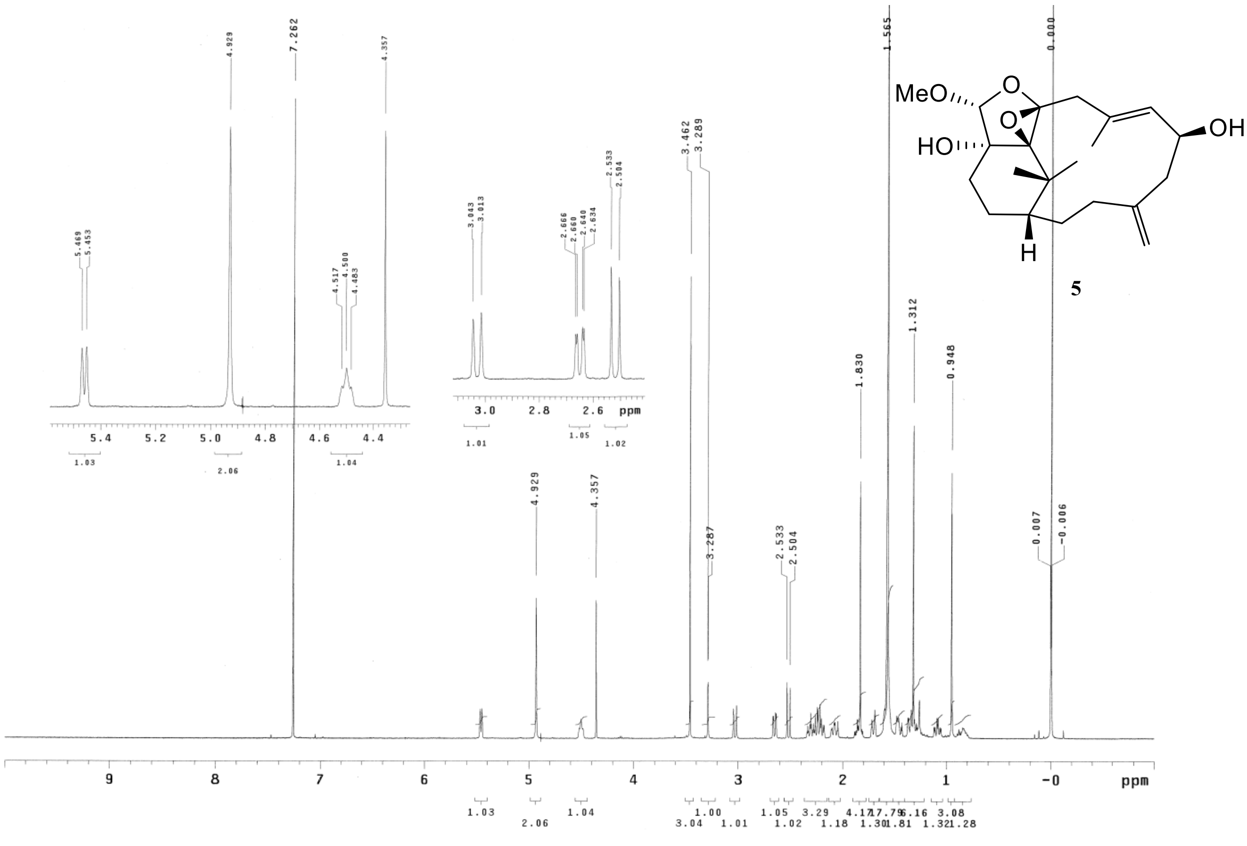

Figure S30. ^{1}H NMR spectrum of **5** in $CDCl_3$ at 500 MHz

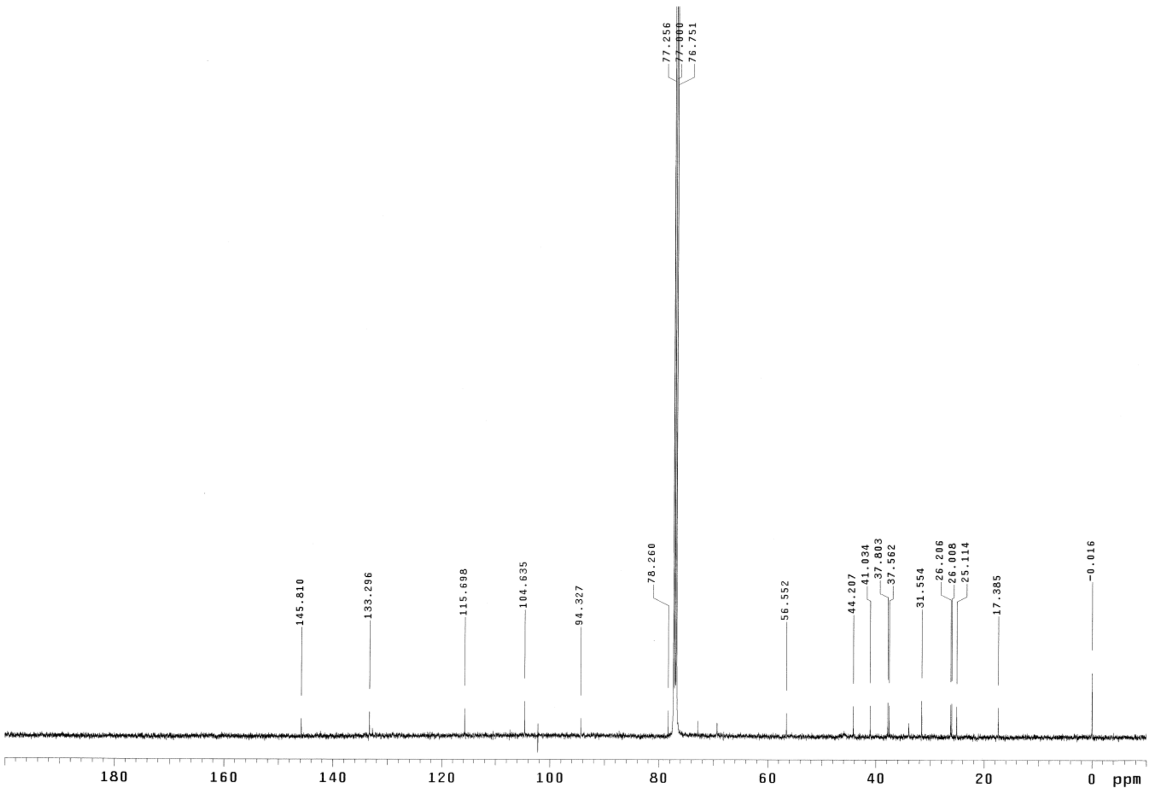

Figure S31. ^{13}C NMR spectrum of **5** in $CDCl_3$ at 125 MHz

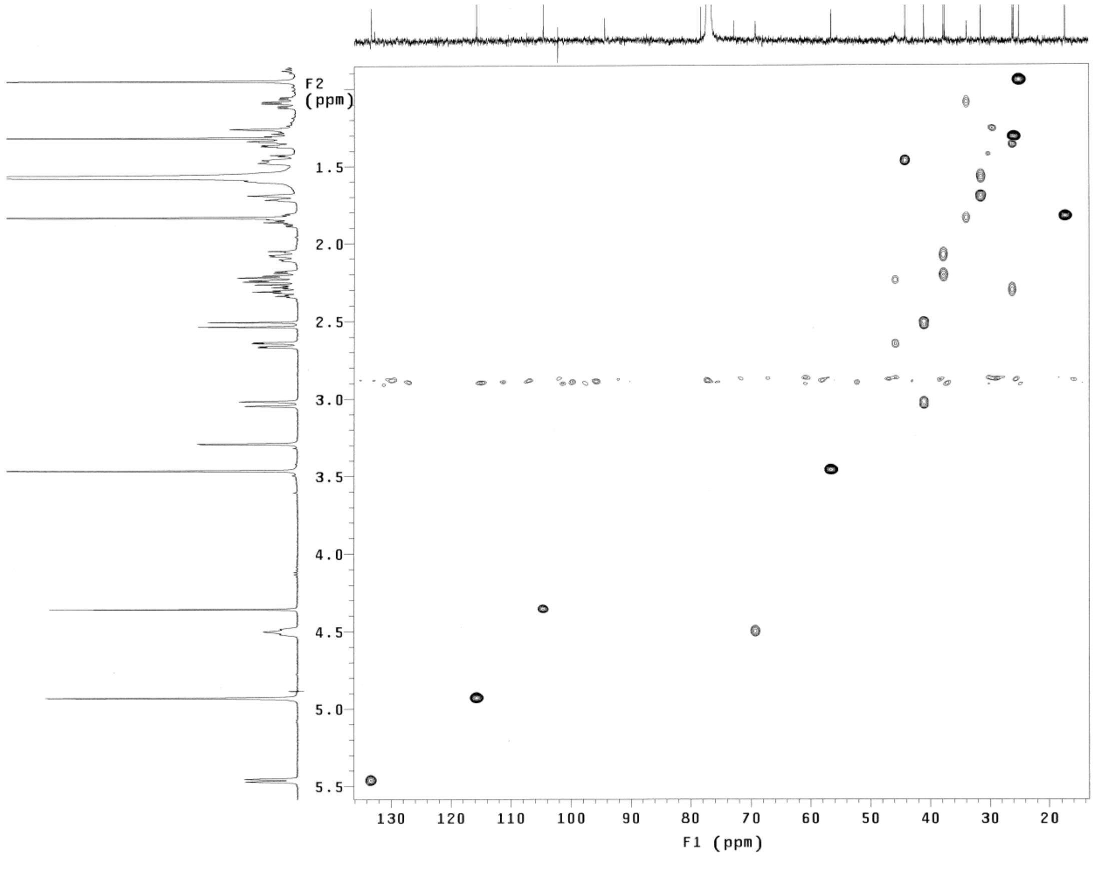

Figure S32. HSQC spectrum of **5** in $CDCl_3$

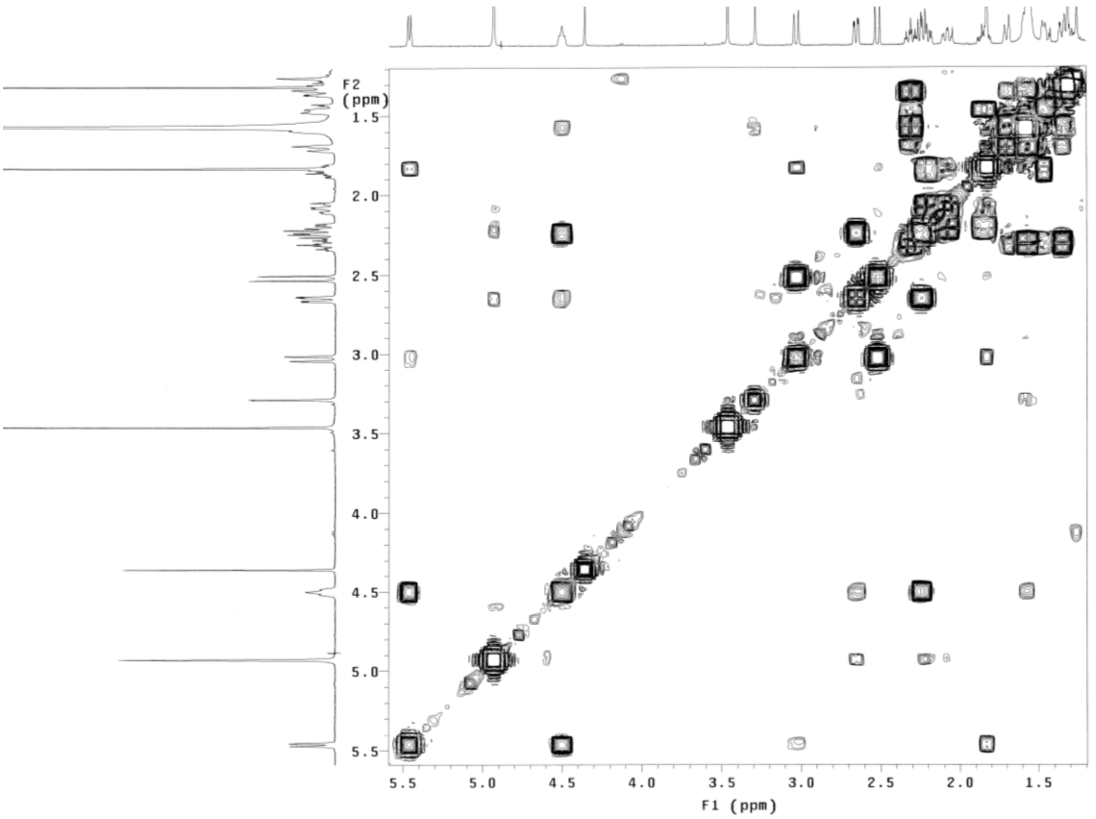

Figure S33. COSY spectrum of **5** in $CDCl_3$

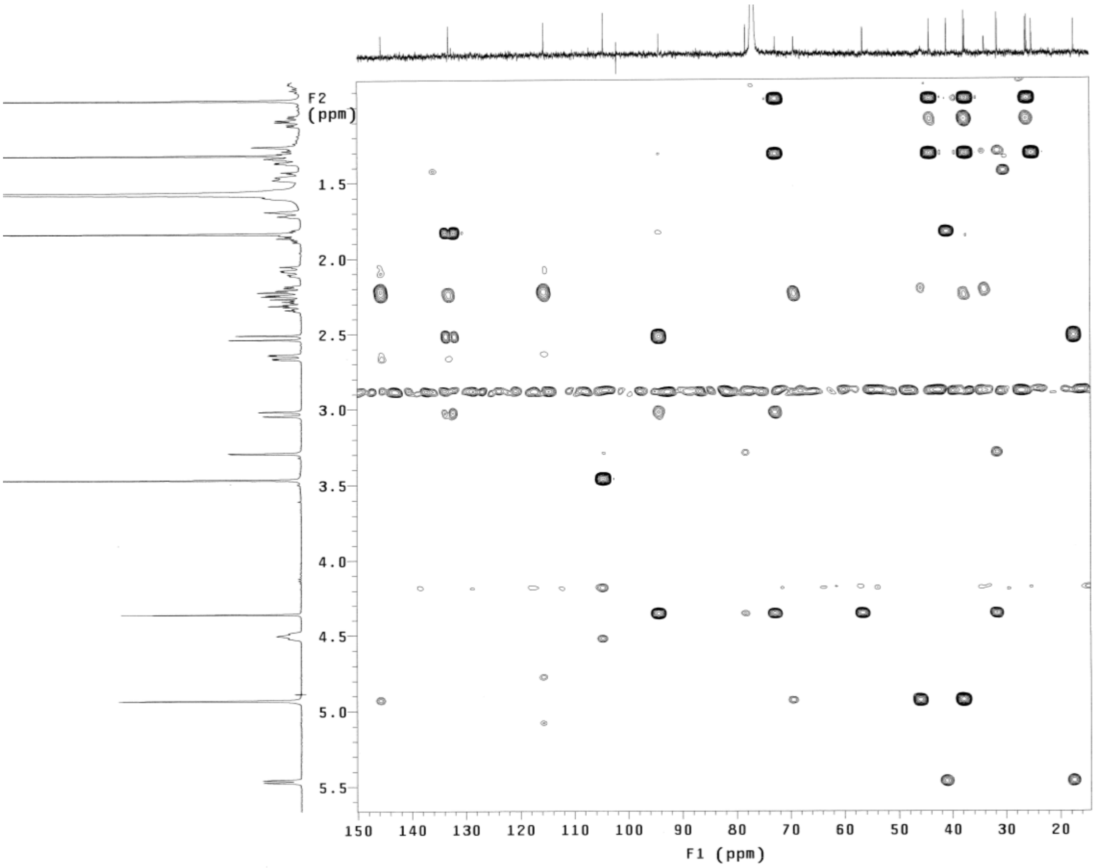

Figure S34. HMBC spectrum of **5** in $CDCl_3$

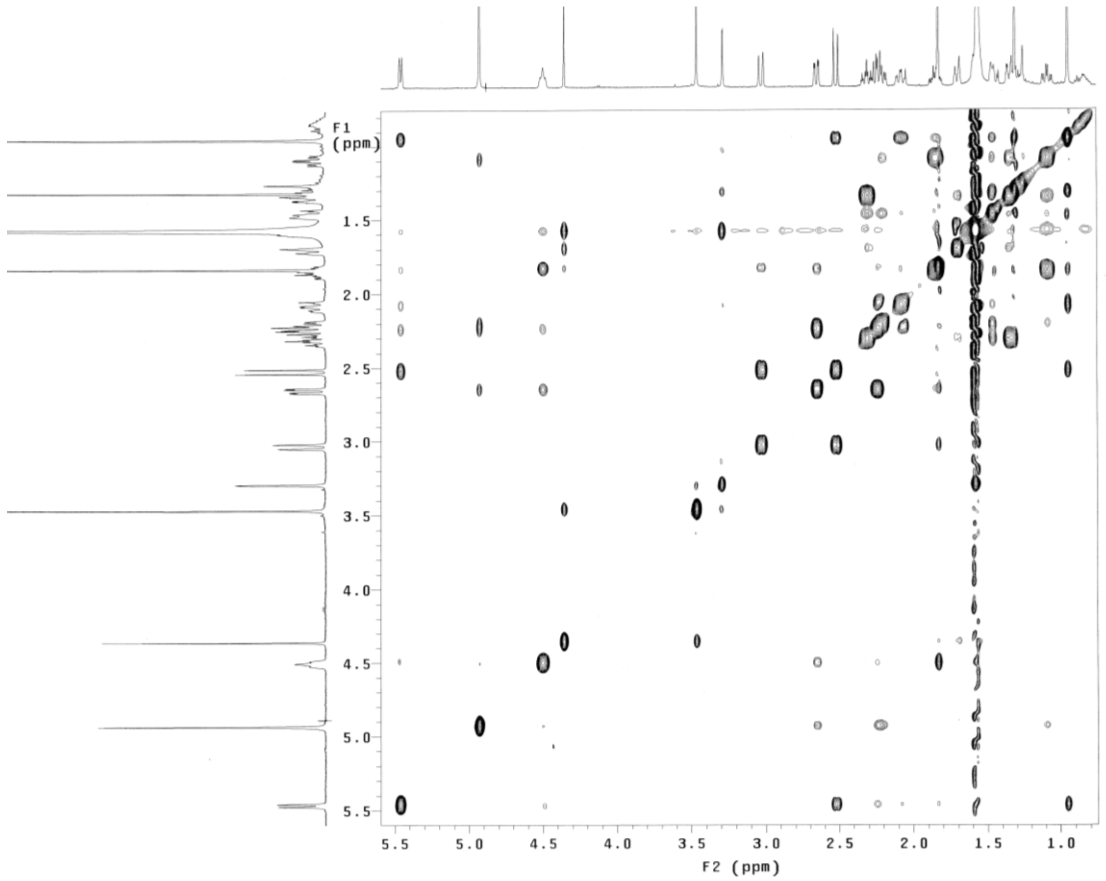

Figure S35. NOESY spectrum of of **5** in $CDCl_3$

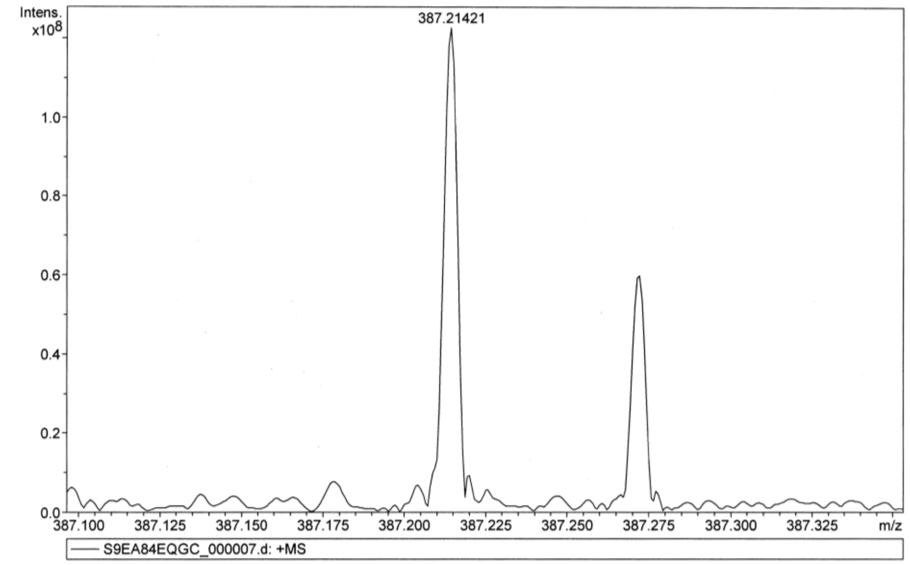

Meas. m/z	#	Formula	Score	m/z	err [mDa]	err [ppm]	mSigma	rdb	e¯ Conf	N-Rule
387.21421	1	C 21 H 32 Na O 5	100.00	387.21420	-0.02	-0.04	32.2	5.5	even	ok

Figure S36. HRESIMS spectrum of **6**

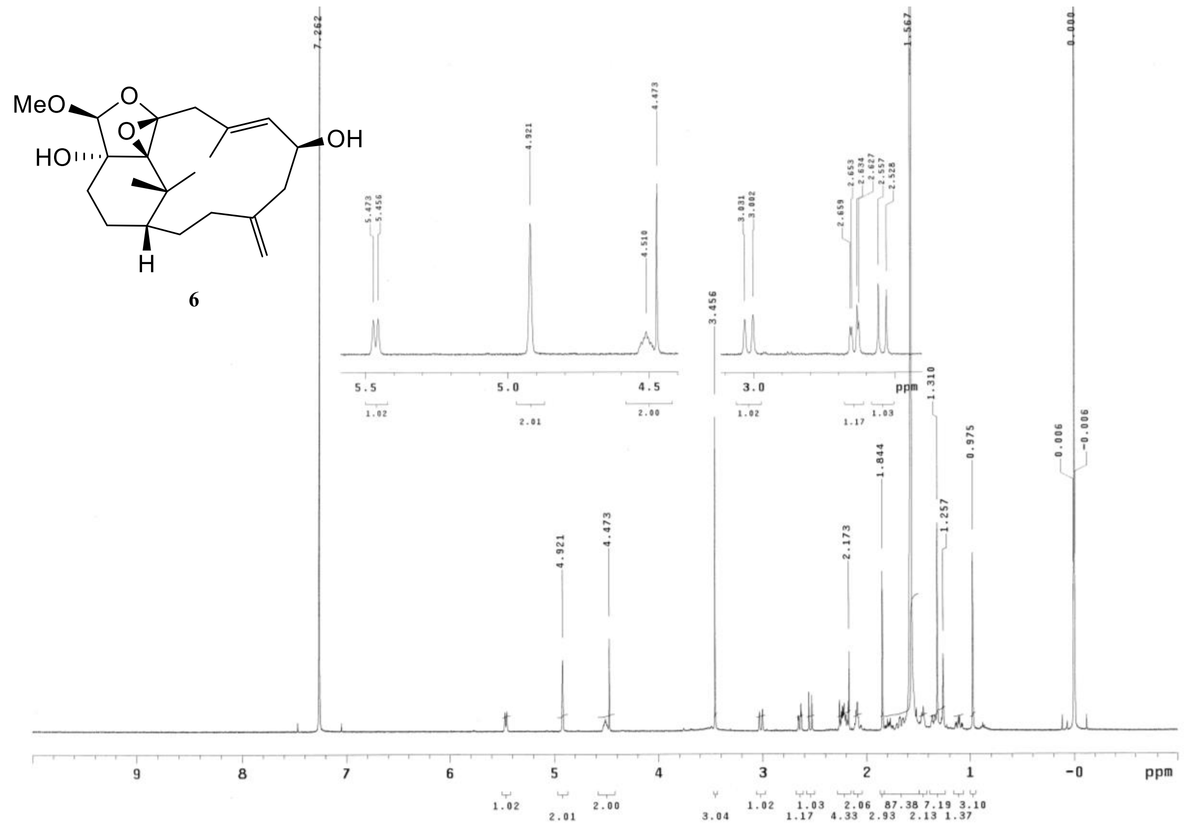

Figure S37. ^{1}H NMR spectrum of **6** $CDCl_3$ at 500 MHz

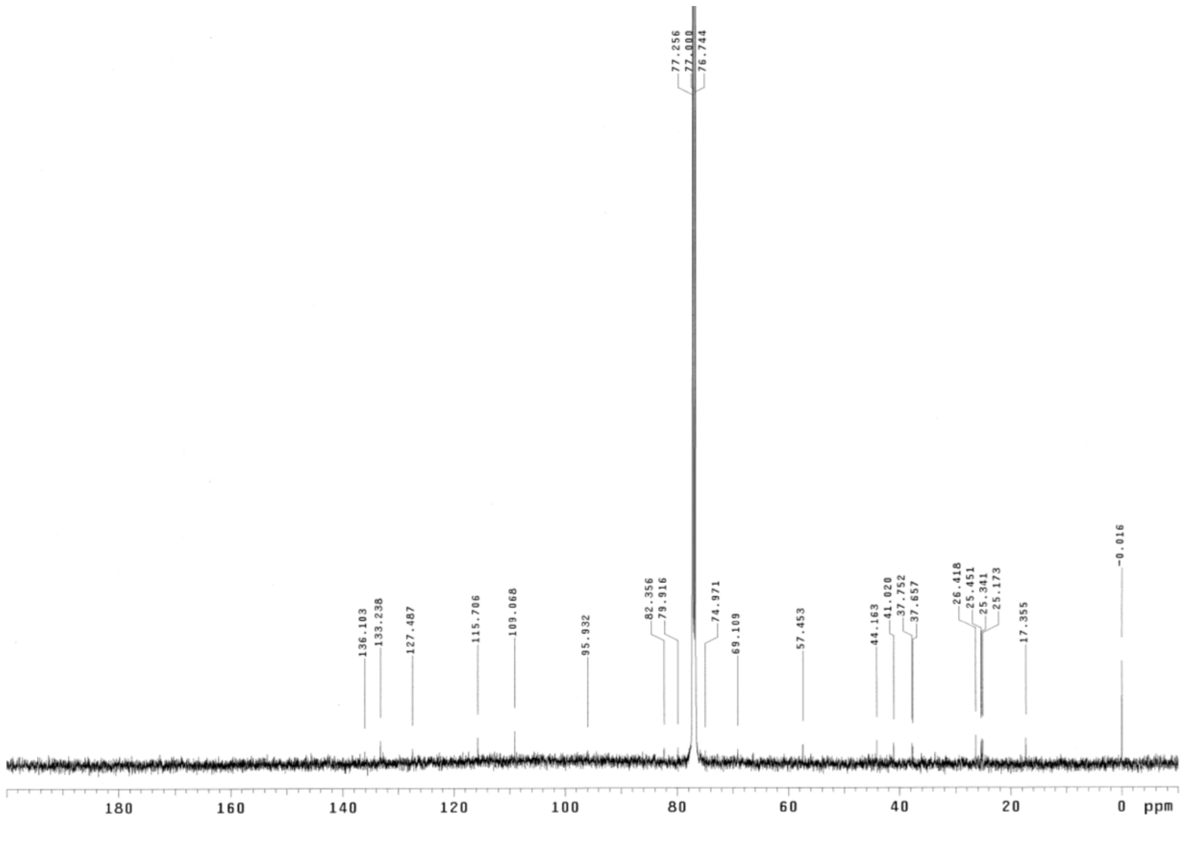

Figure S38. ^{13}C NMR spectrum of **6** in $CDCl_3$ at 125 MHz

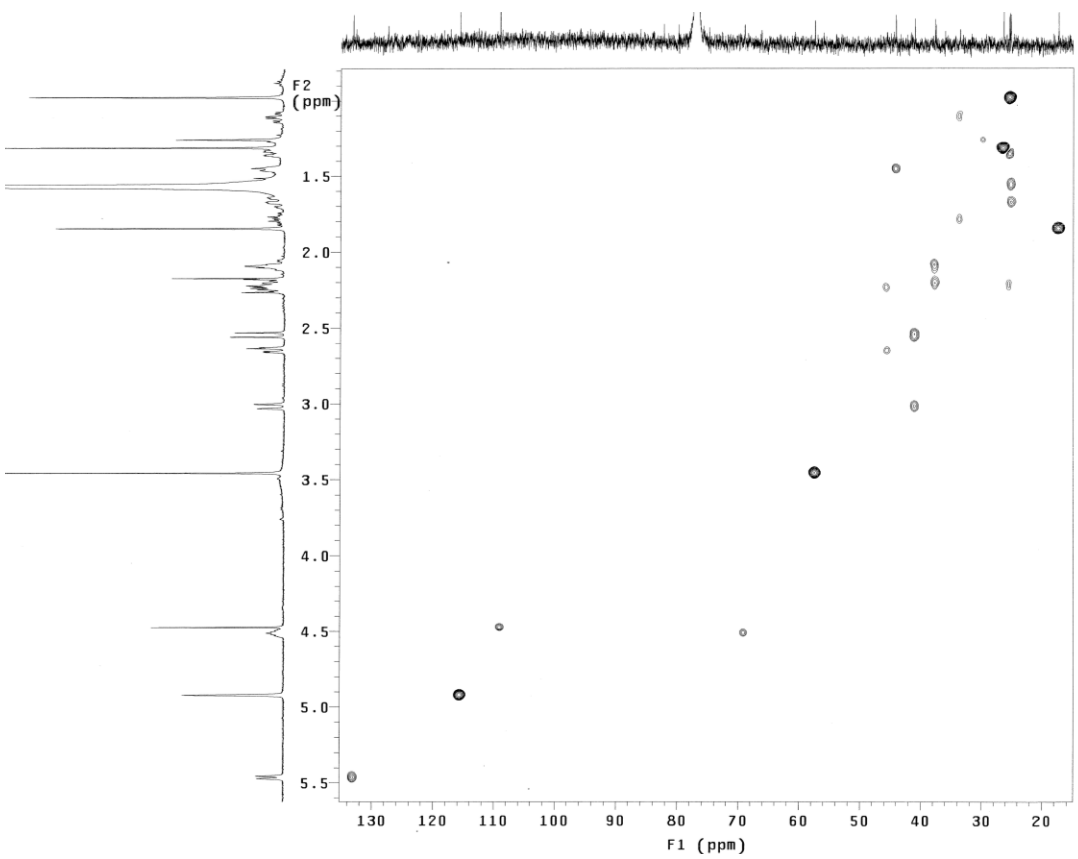

Figure S39. HSQC spectrum of **6** in $CDCl_3$

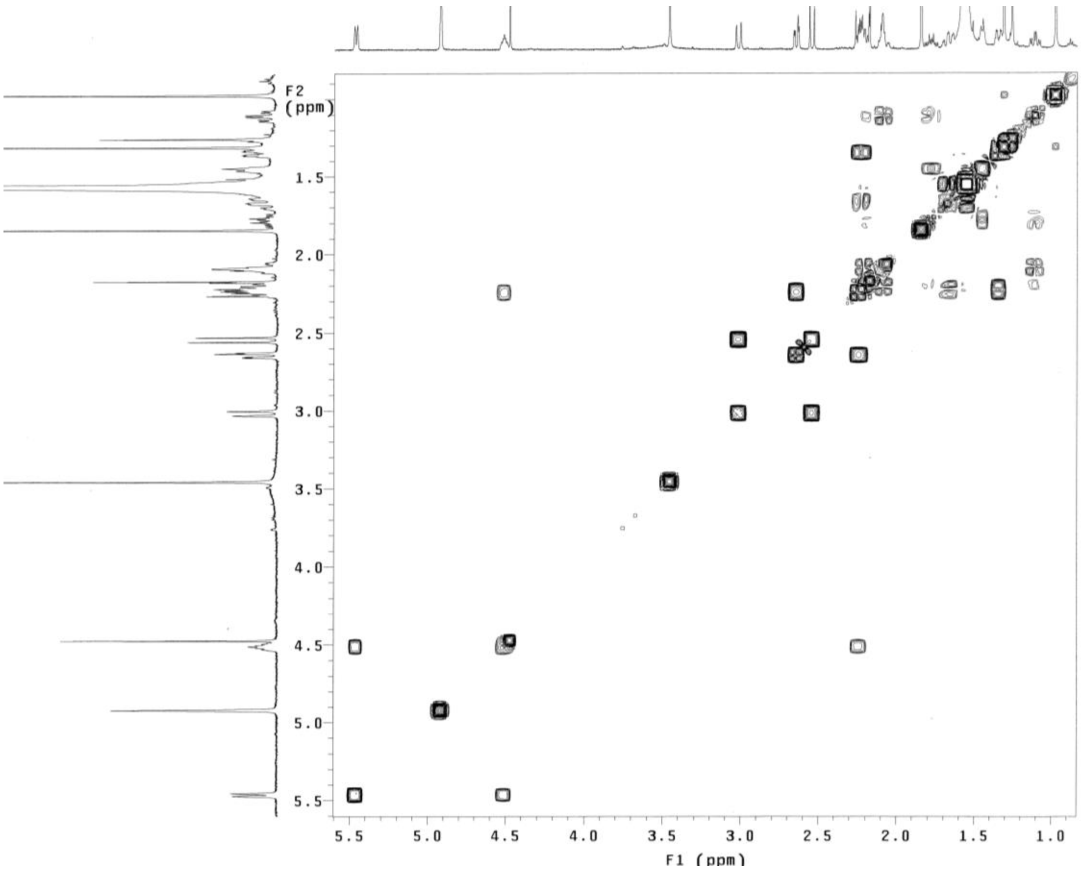

Figure S40. COSY spectrum of **6** in $CDCl_3$

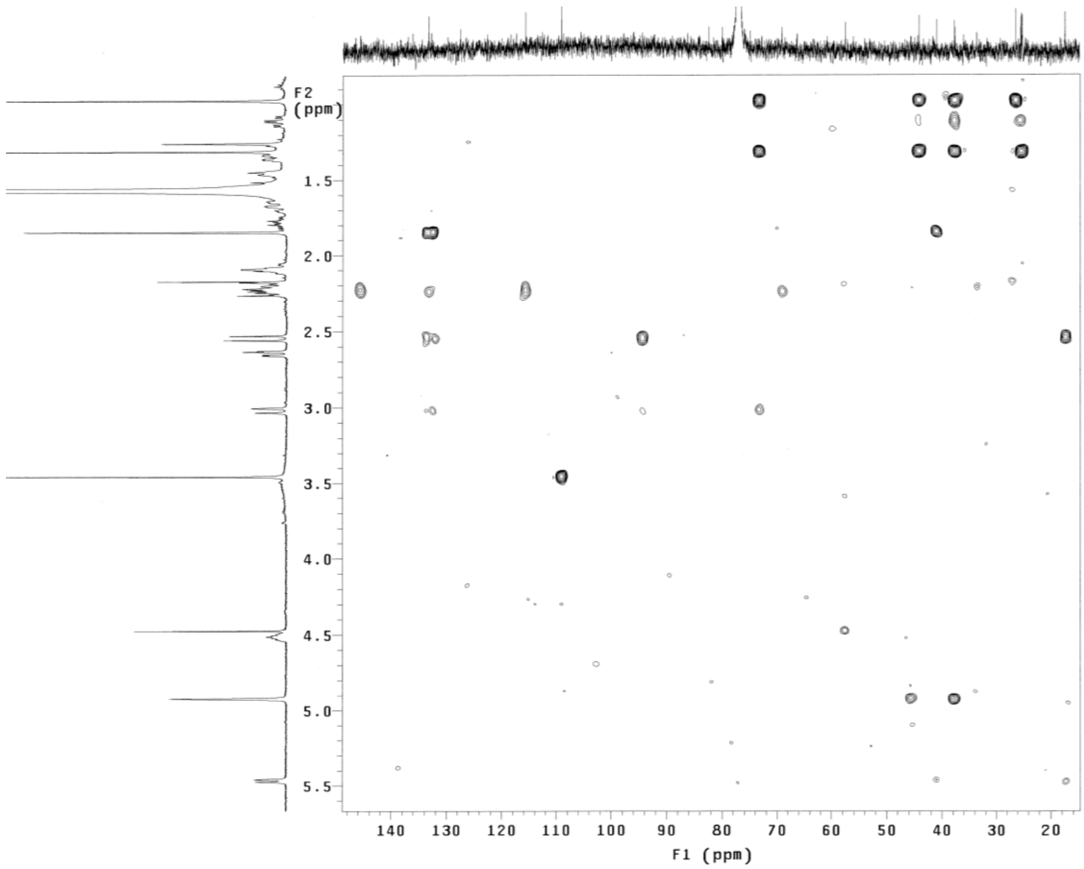

Figure S41. HMBC spectrum of **6** in $CDCl_3$

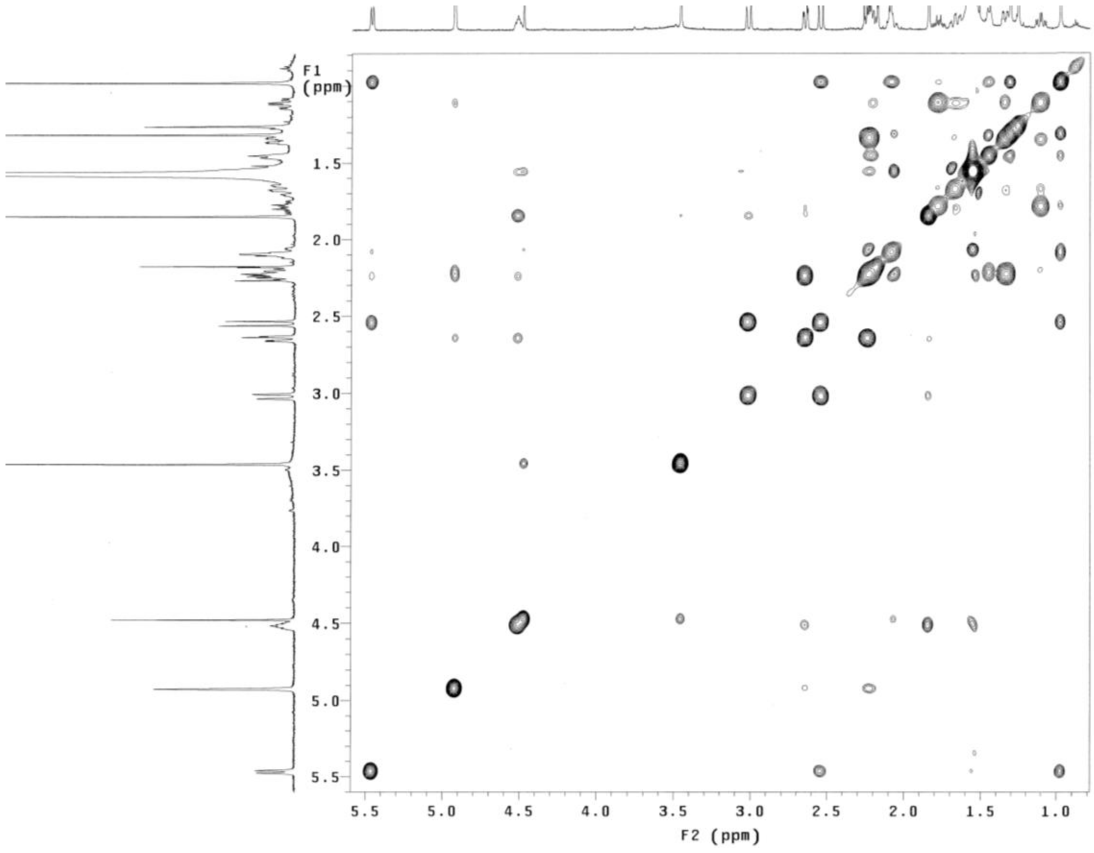

Figure S42. NOESY spectrum of **6** in $CDCl_3$

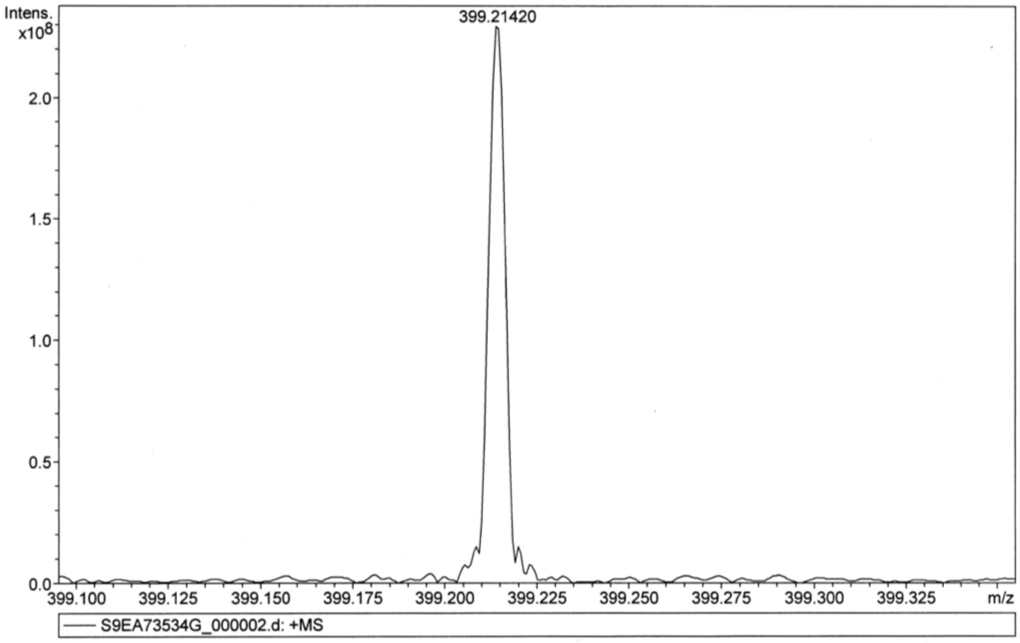

Meas. m/z	#	Formula	Score	m/z	err [mDa]	err [ppm]	mSigma	rdb	e⁻ Conf	N-Rule
399.21420	1	C 22 H 32 Na O 5	100.00	399.21420	-0.01	-0.01	11.1	6.5	even	ok

Figure S43. HRESIMS spectrum of **7**

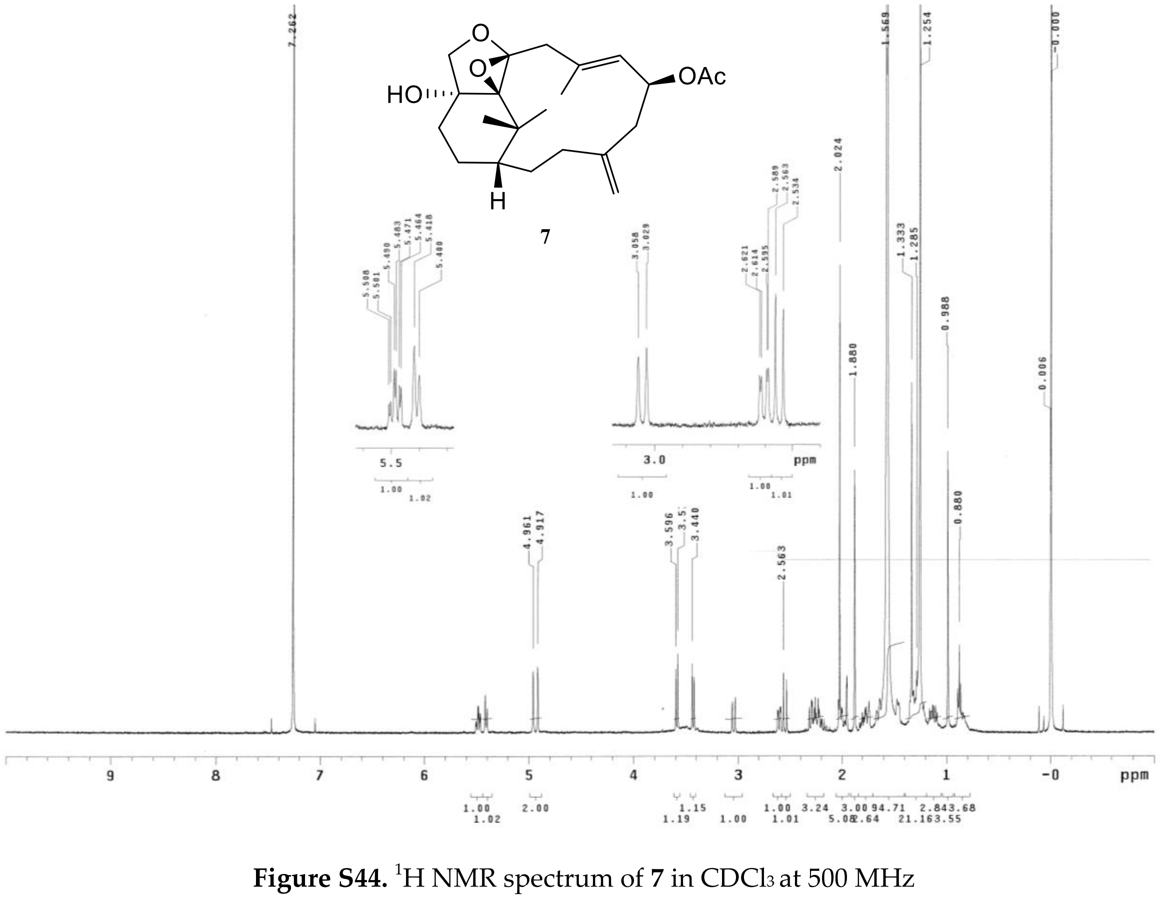

Figure S44. ^{1}H NMR spectrum of **7** in $CDCl_3$ at 500 MHz

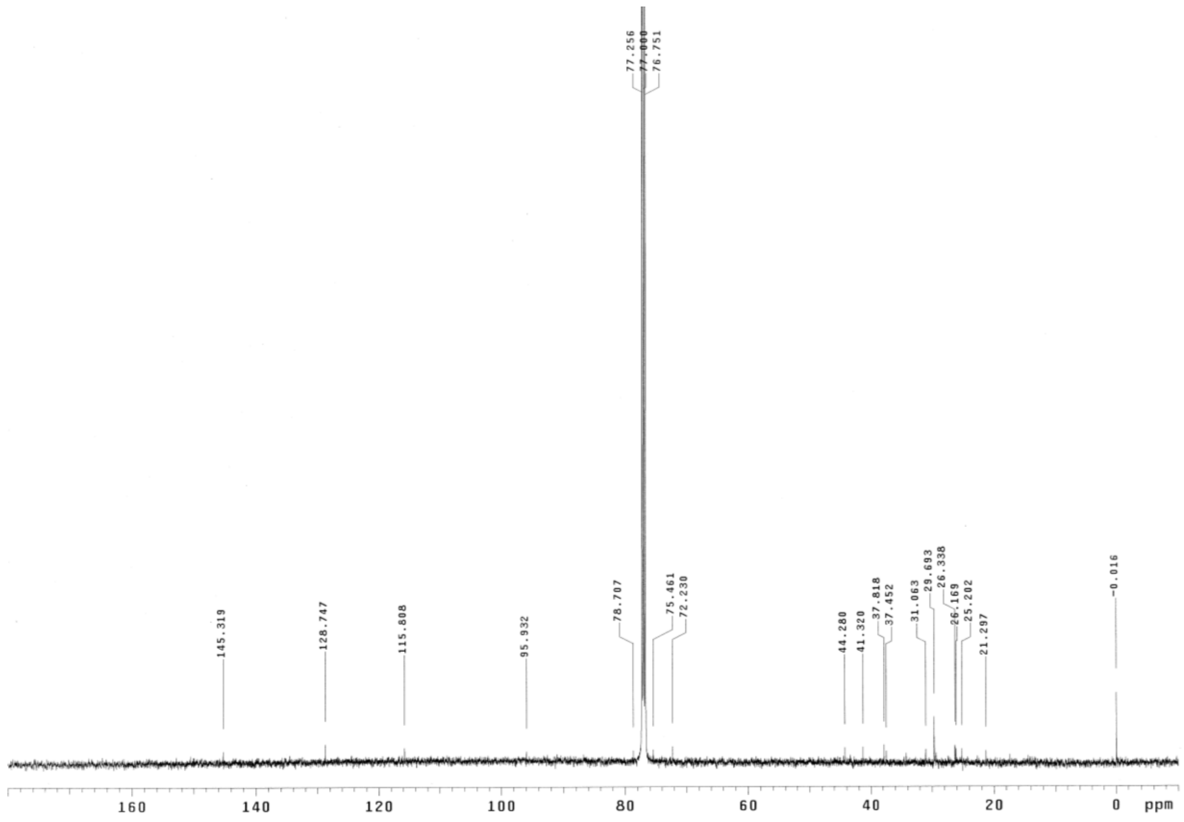

Figure S45. ^{13}C NMR spectrum of **7** in $CDCl_3$ at 125 MHz

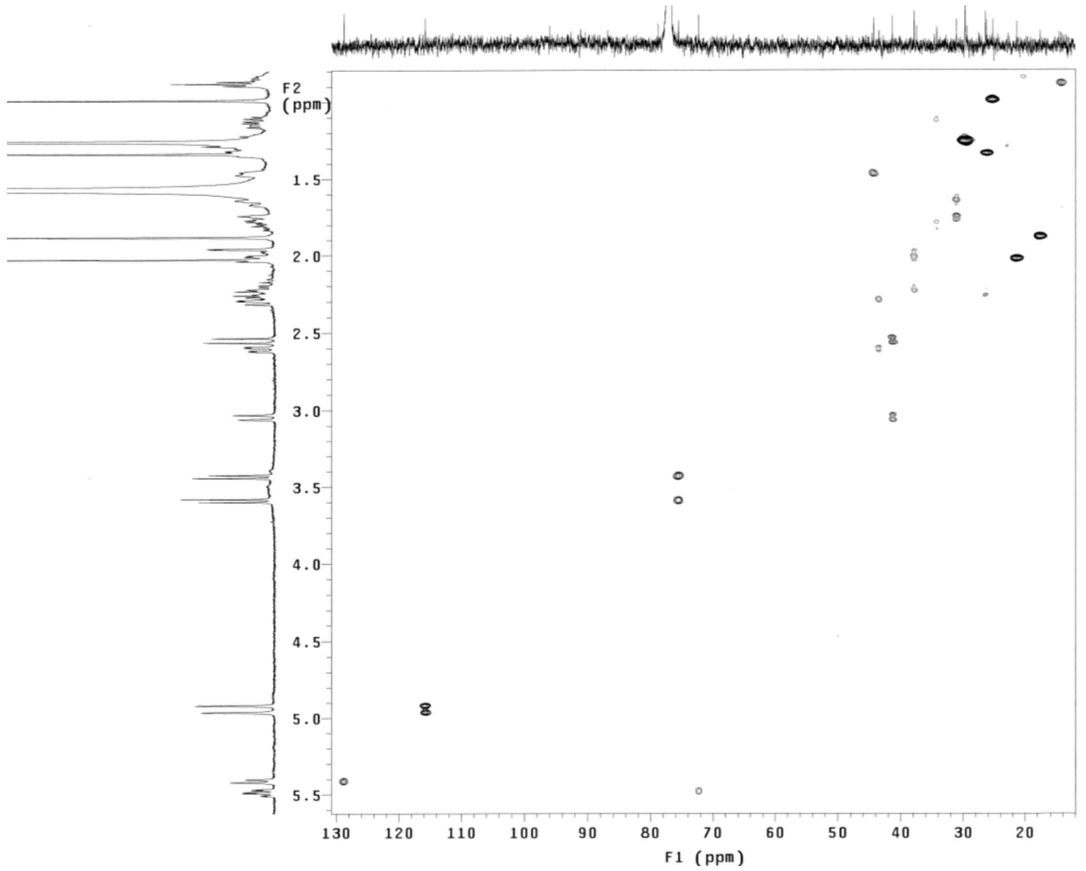

Figure S46. HSQC spectrum of **7** in $CDCl_3$

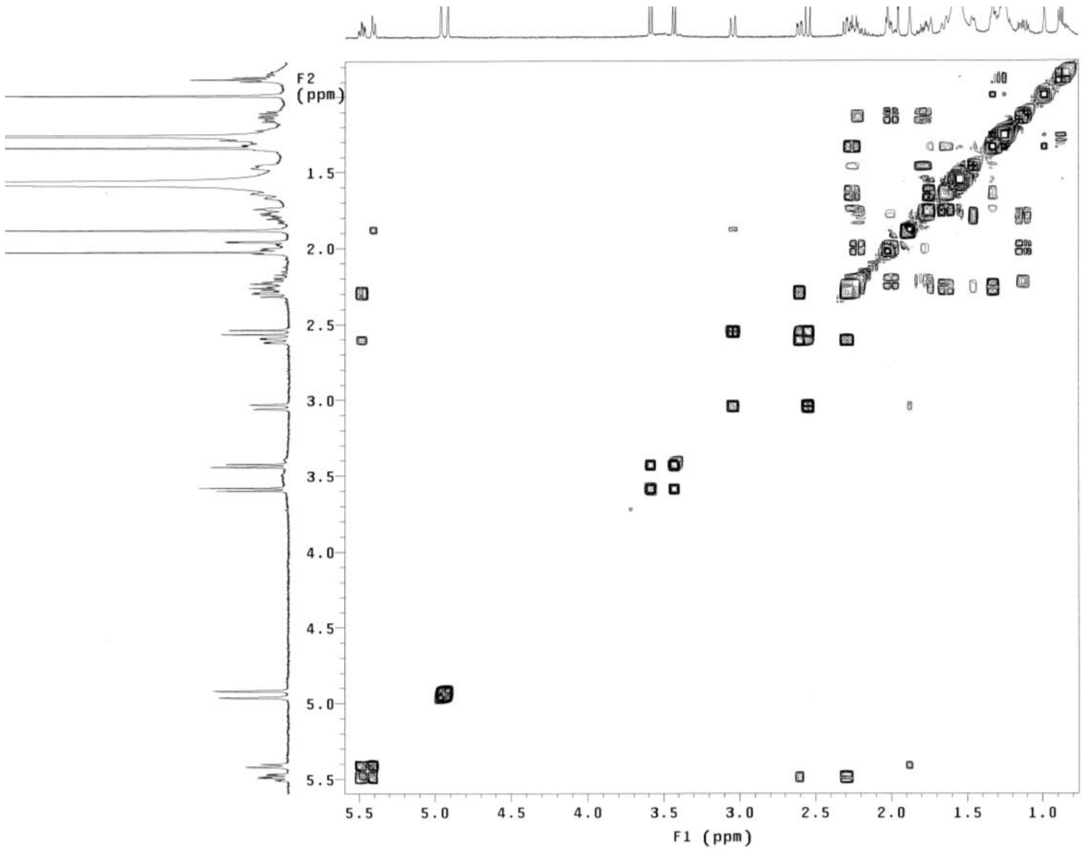

Figure S47. COSY spectrum of **7** in $CDCl_3$

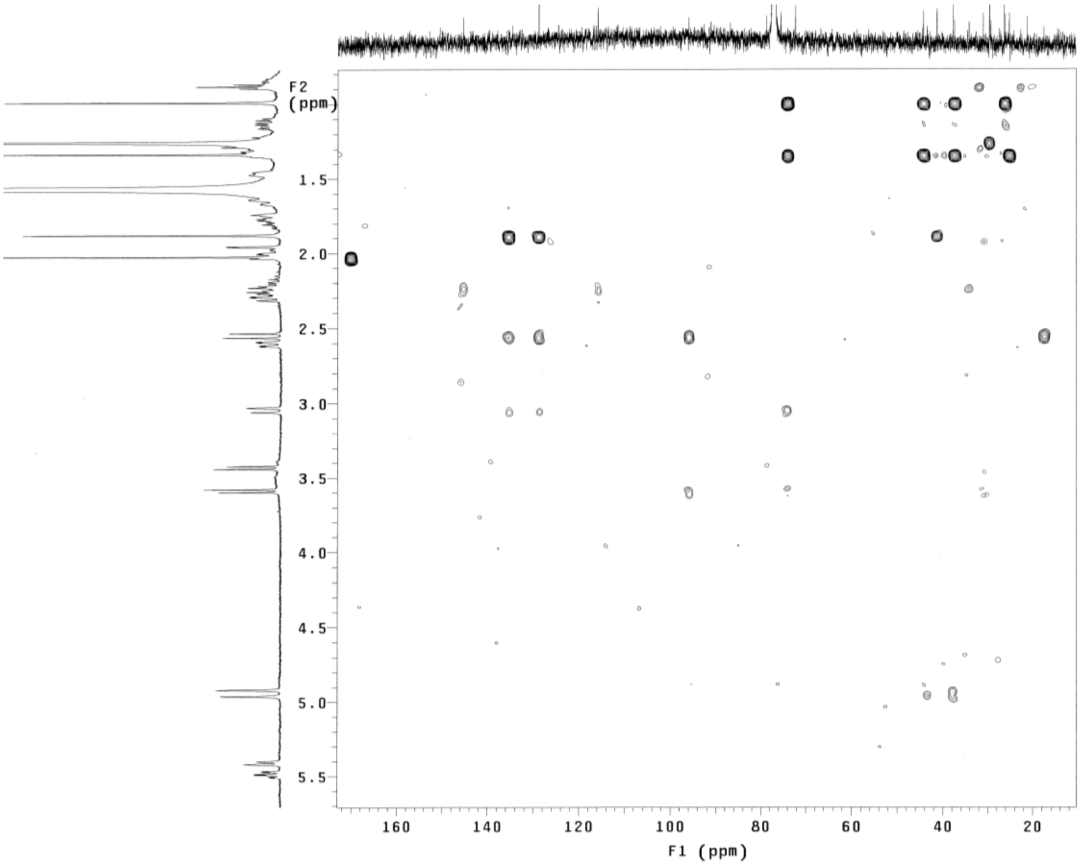

Figure S48. HMBC spectrum of **7** in $CDCl_3$

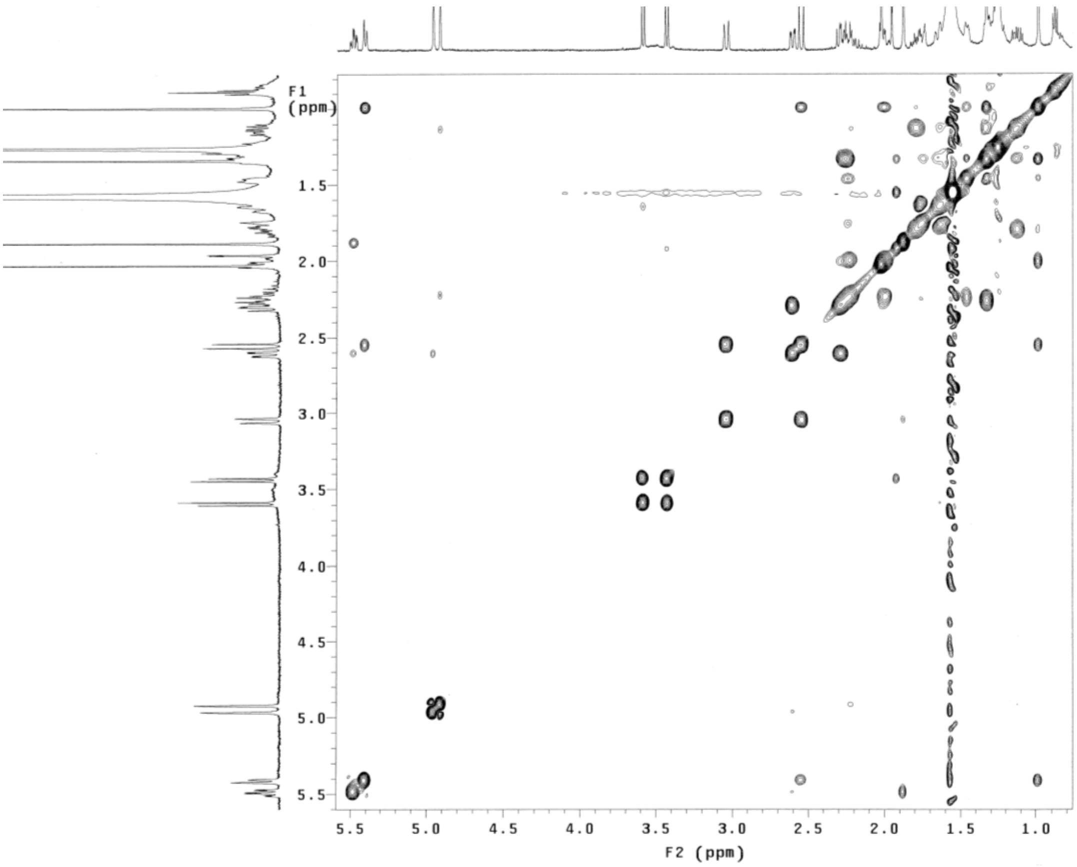

Figure S49. NOESY spectrum of **7** in $CDCl_3$

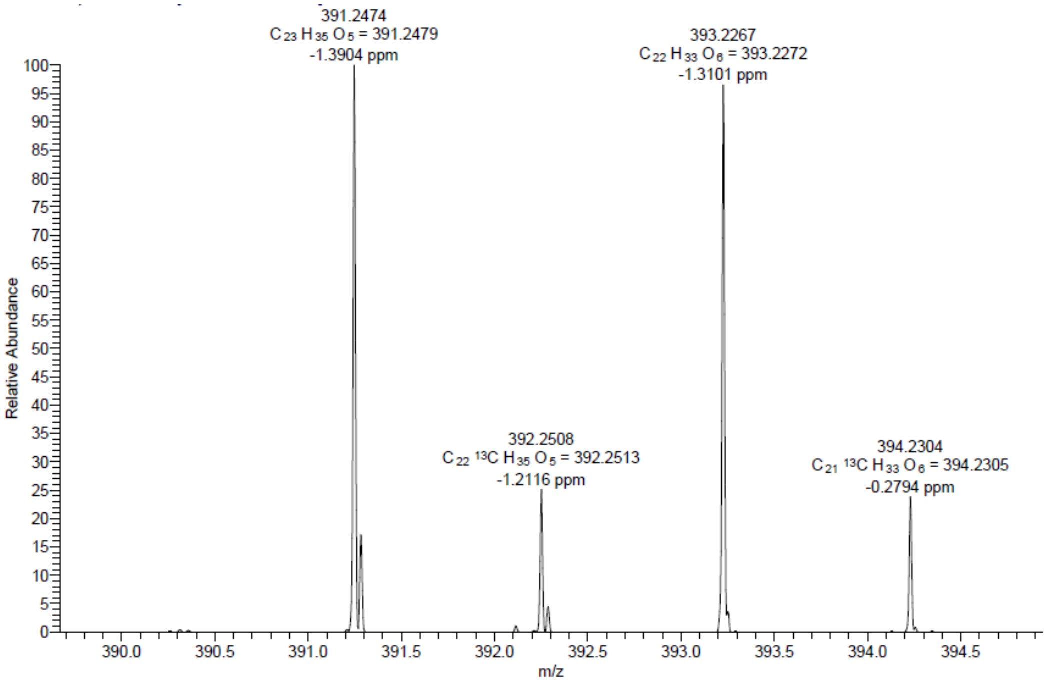

Figure S50. HRESIMS spectrum of **8**

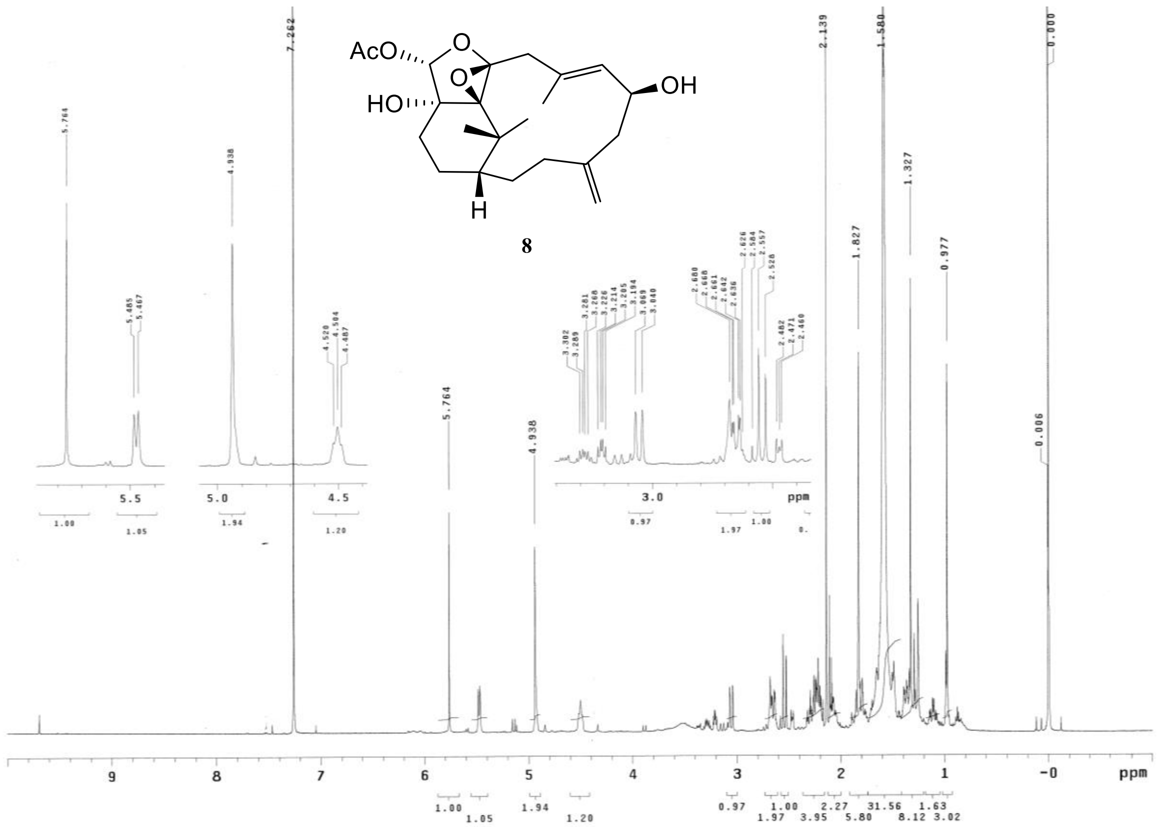

Figure S51. ^{1}H NMR spectrum of **8** in CDCl3 at 500 MHz

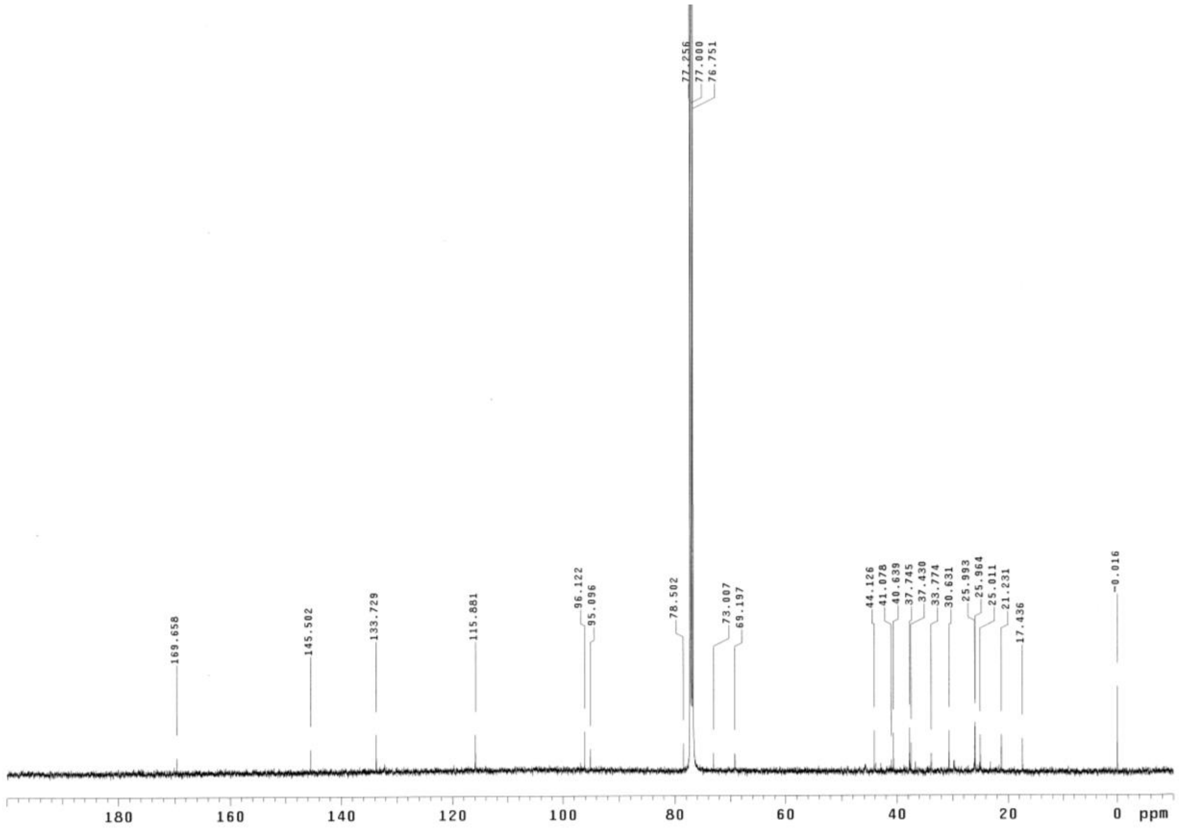

Figure S52. ^{13}C NMR spectrum of **8** in CDCl3 at 125 MHz

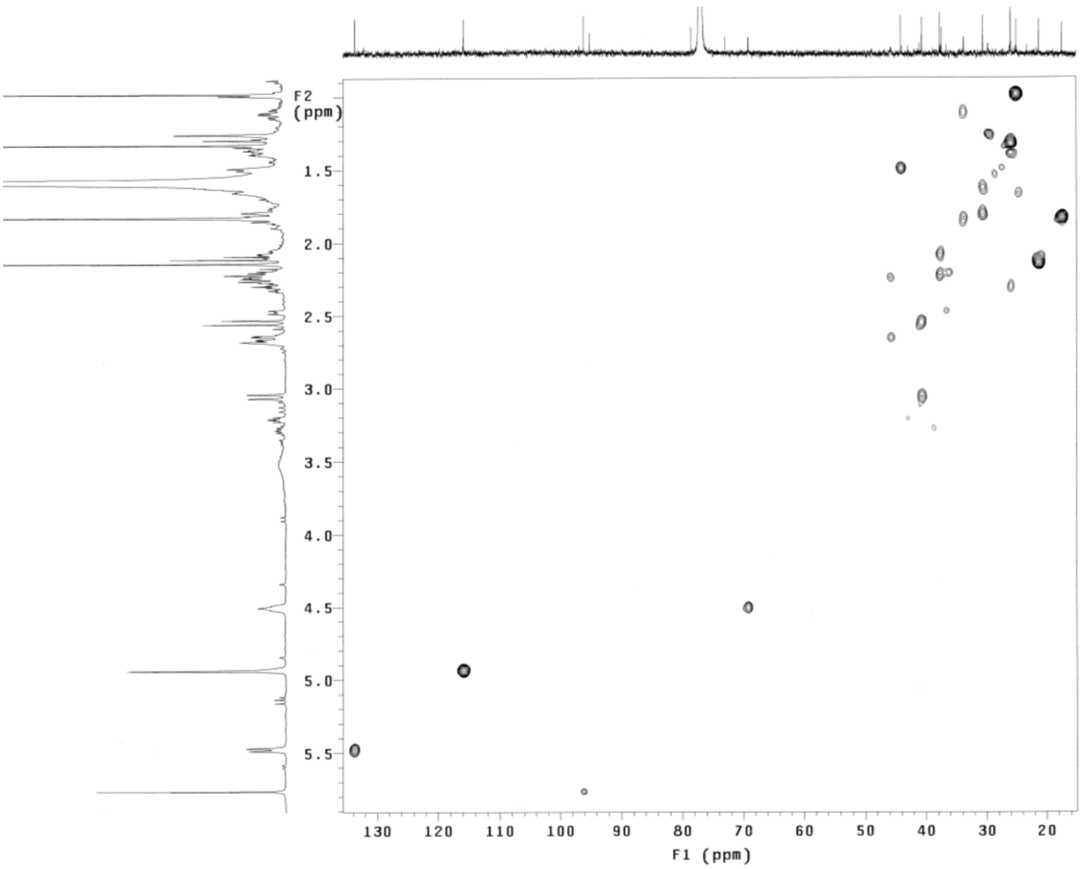

Figure S53. HSQC spectrum of **8** in $CDCl_3$

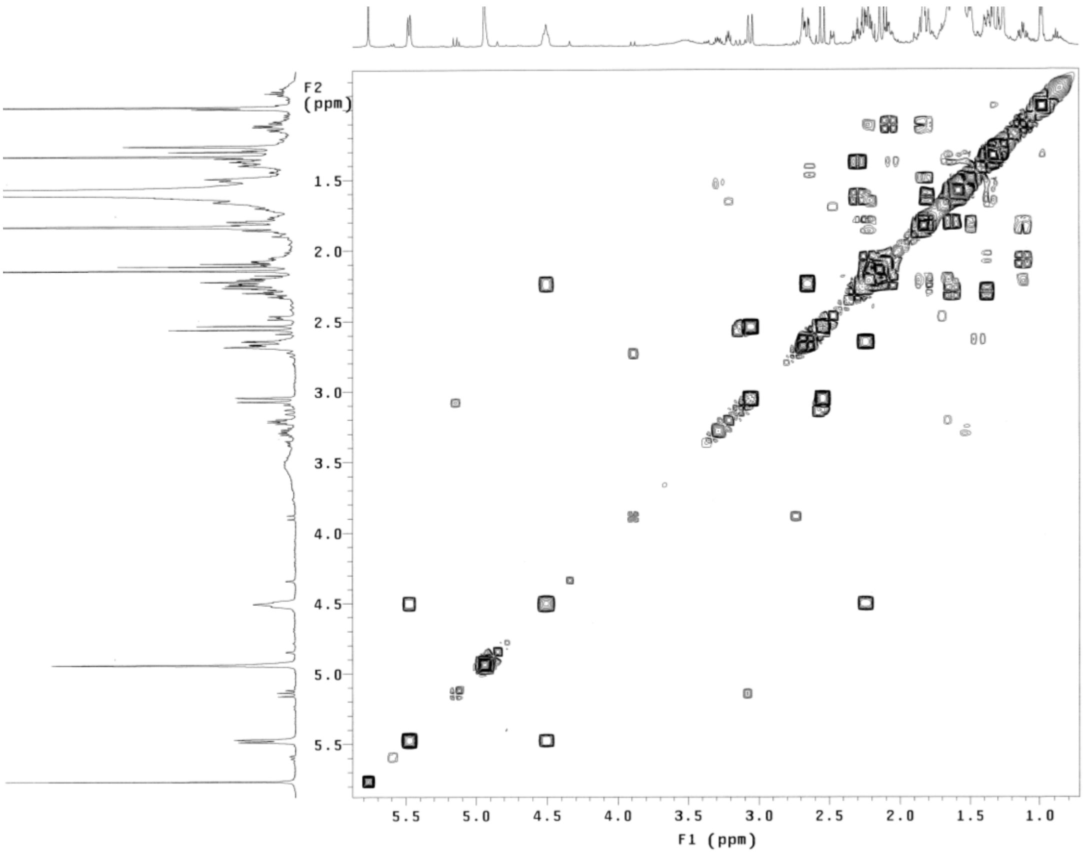

Figure S54. COSY spectrum of **8** in $CDCl_3$

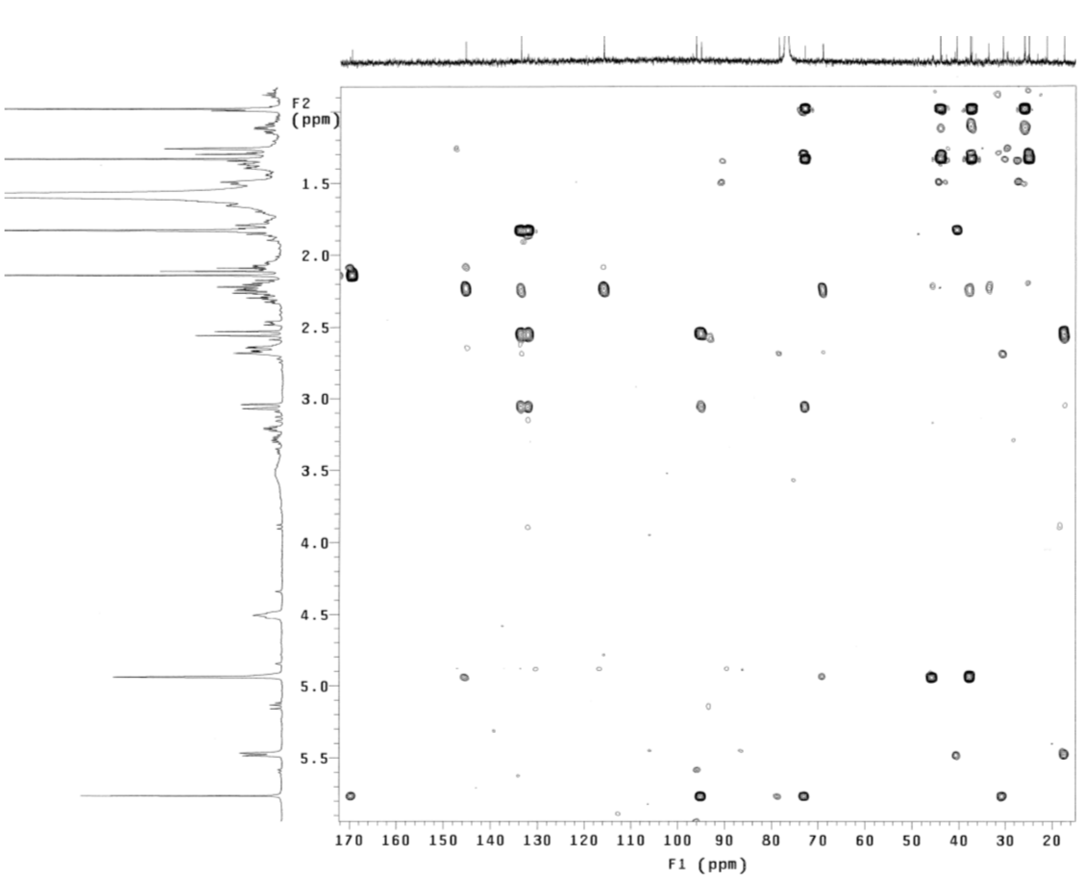

Figure S55. HMBC spectrum of **8** in $CDCl_3$

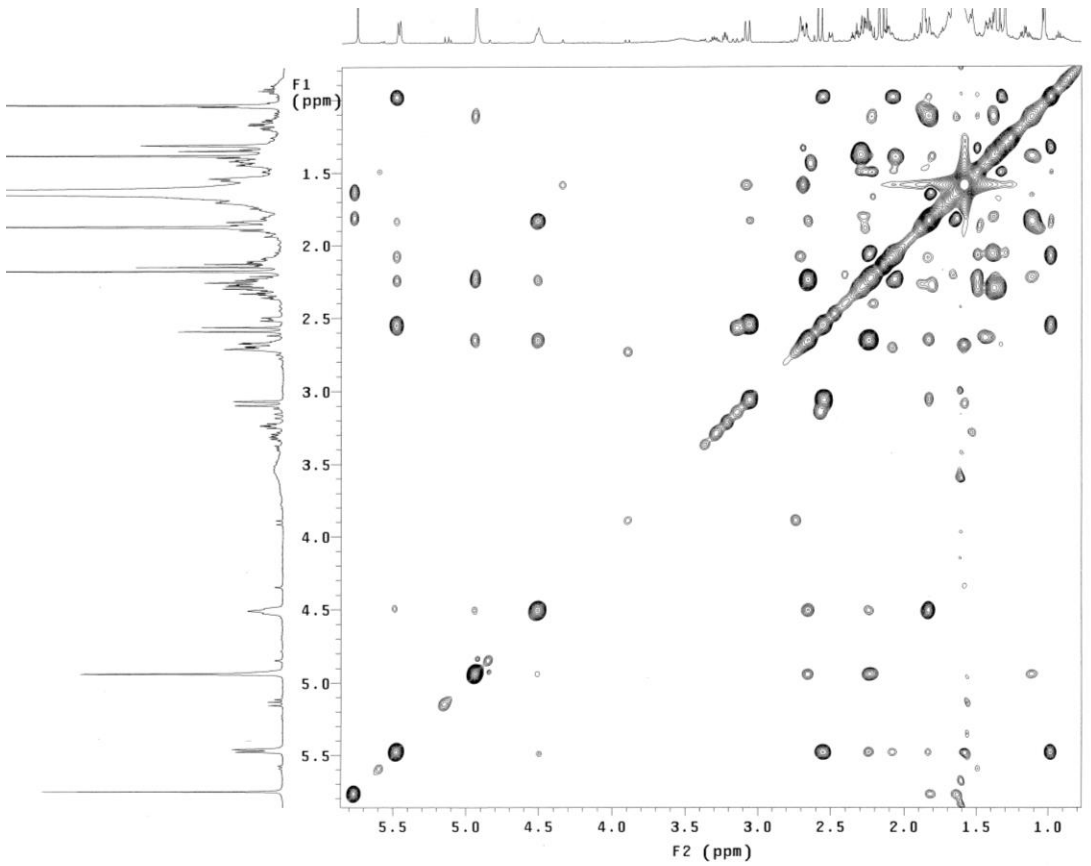

Figure S56. NOESY spectrum of **8** in $CDCl_3$

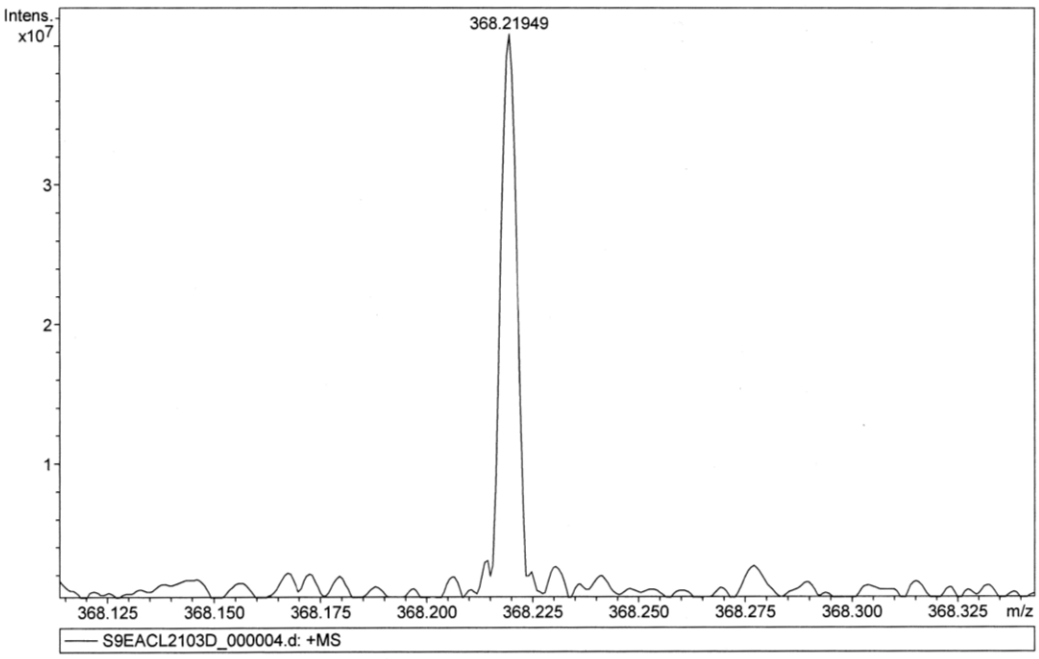

Meas. m/z	#	Formula	Score	m/z	err [mDa]	err [ppm]	mSigma	rdb	e⁻ Conf	N-Rule
368.21949	1	C 21 H 31 N Na O 3	100.00	368.21961	0.13	0.34	25.0	6.5	even	ok

Figure S57. HRESIMS spectrum of **9**

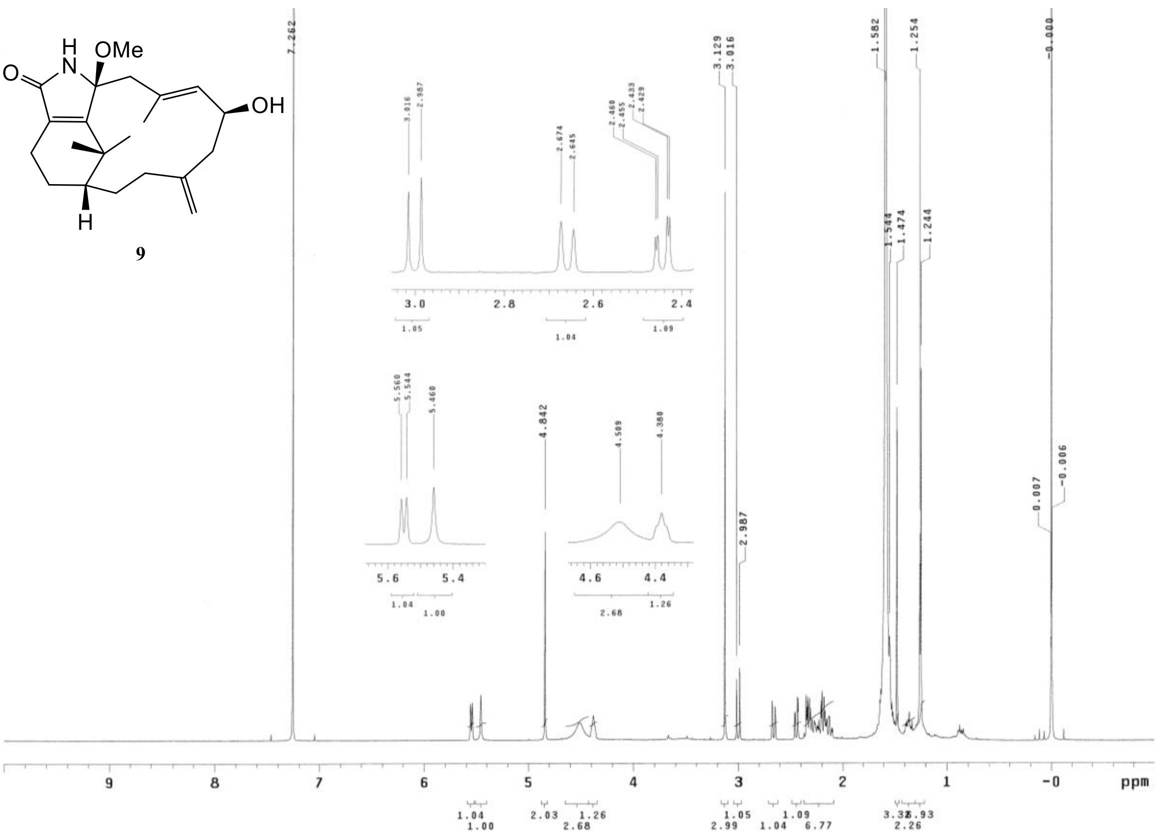

Figure S58. ^{1}H NMR spectrum of **9** in $CDCl_3$ at 500 MHz

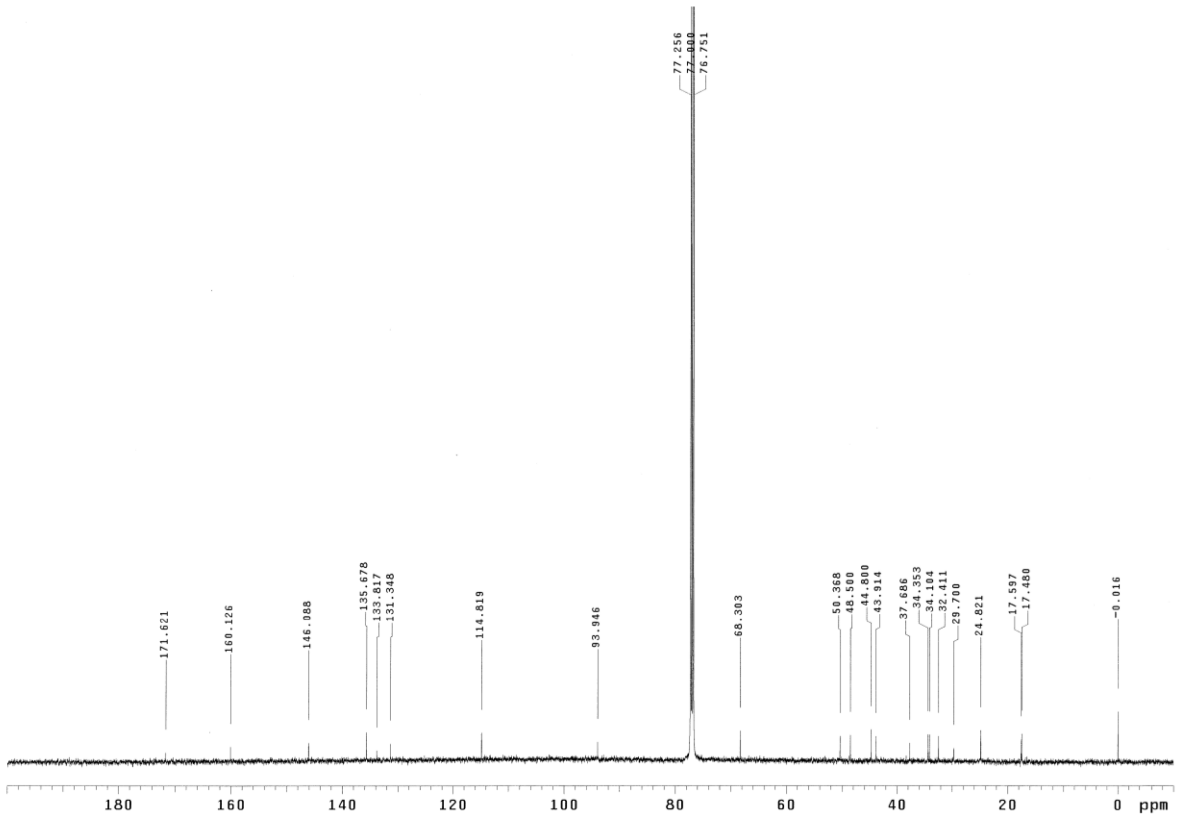

Figure S59. ^{13}C NMR spectrum of **9** in $CDCl_3$ at 125 MHz

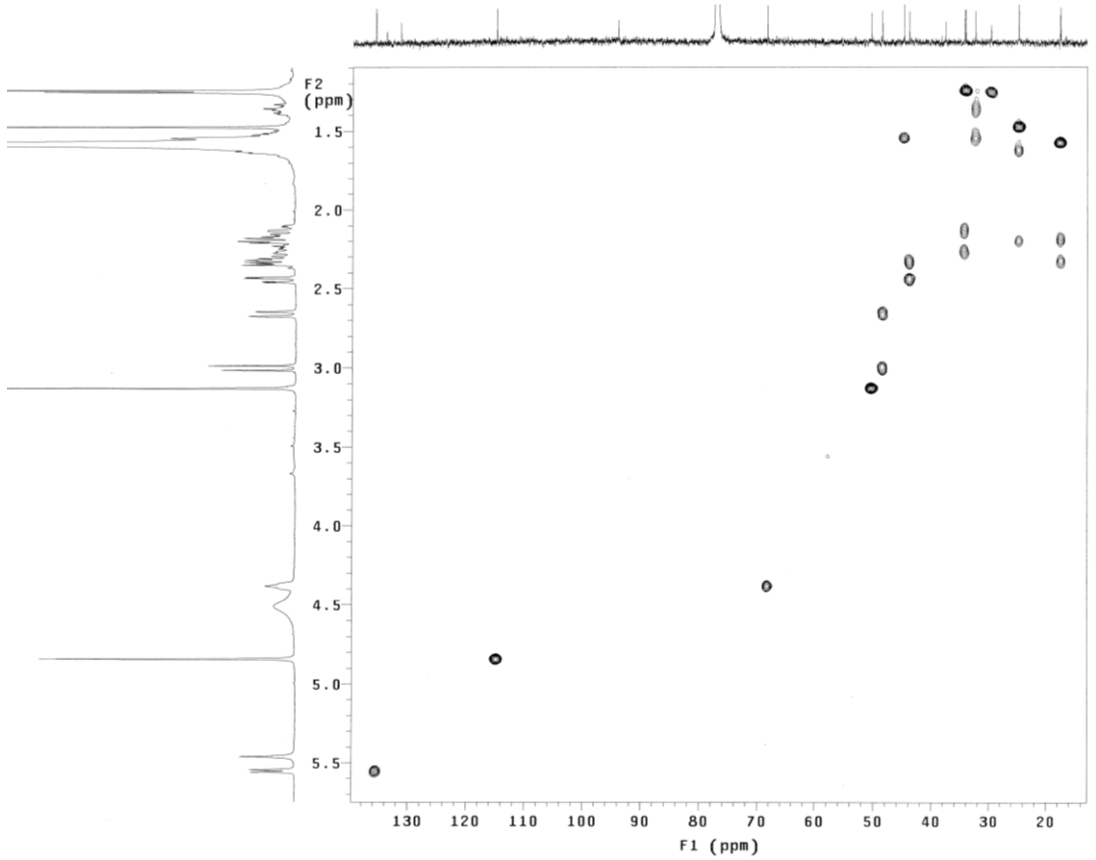

Figure S60. HSQC spectrum of **9** in $CDCl_3$

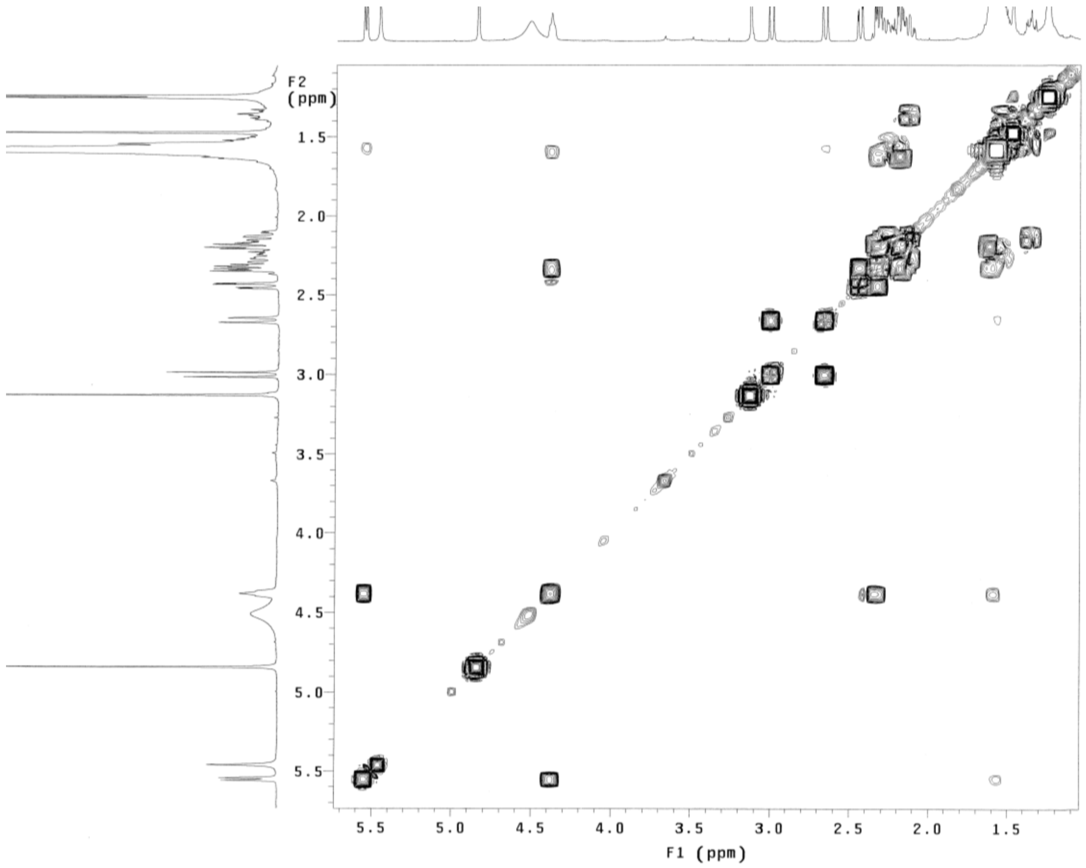

Figure S61. COSY spectrum of **9** in $CDCl_3$

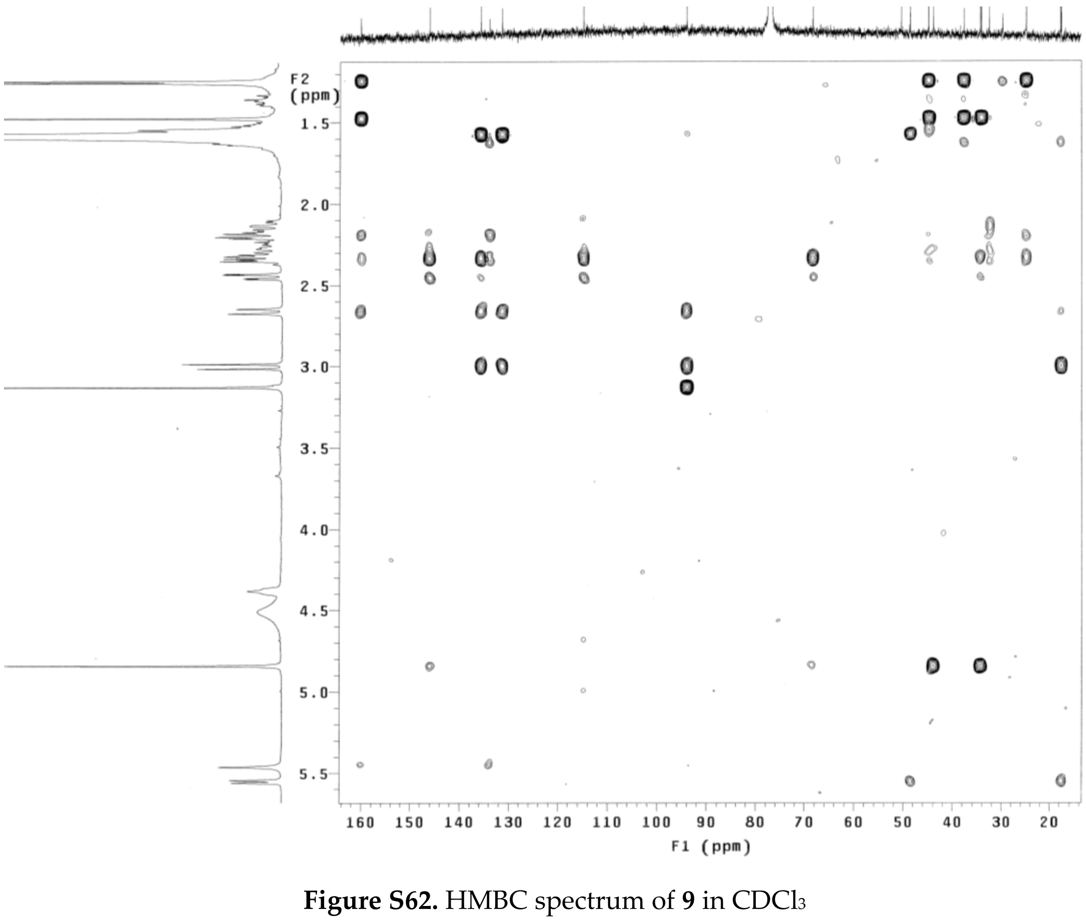

Figure S62. HMBC spectrum of **9** in $CDCl_3$

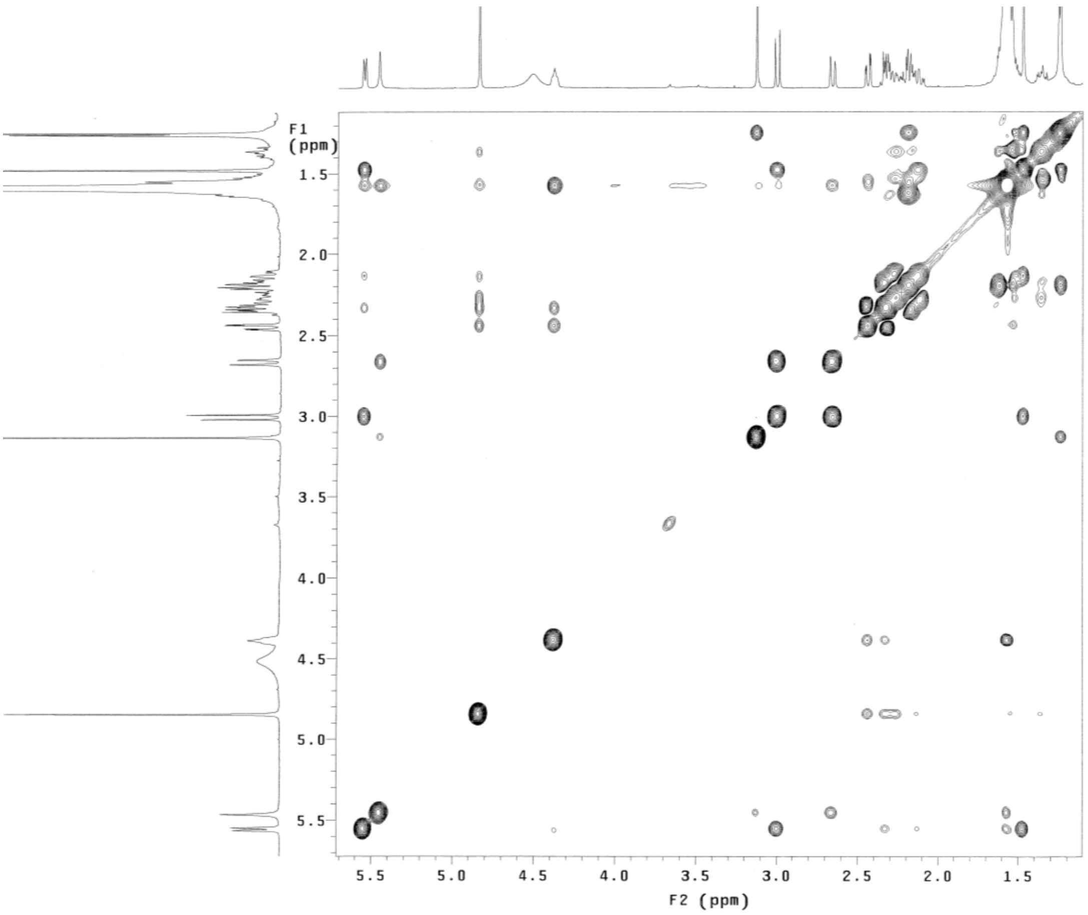

Figure S63. NOESY spectrum of **9** in $CDCl_3$

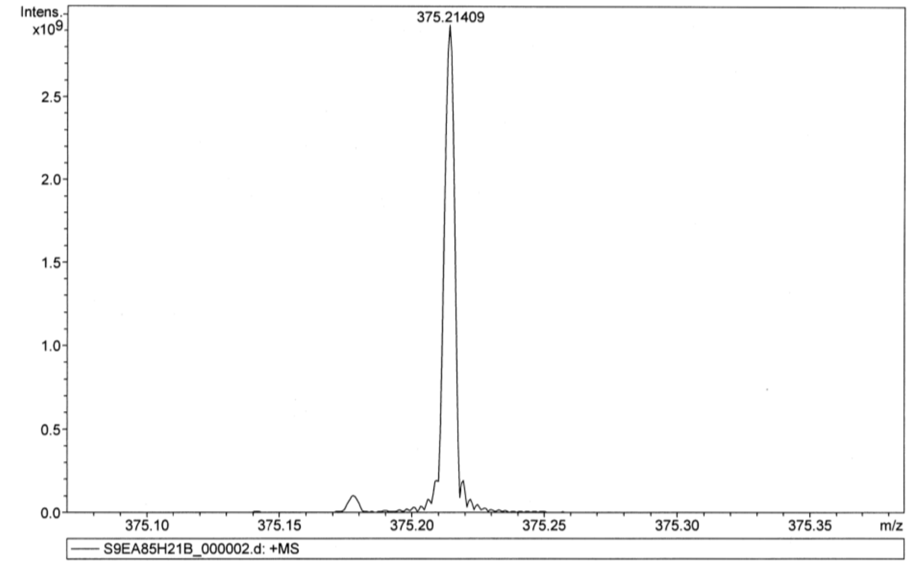

Meas. m/z	#	Formula	Score	m/z	err [mDa]	err [ppm]	mSigma	rdb	e⁻ Conf	N-Rule
375.21409	1	C 20 H 32 Na O 5	100.00	375.21420	0.10	0.28	9.0	4.5	even	ok

Figure S64. HRESIMS spectrum of **10**

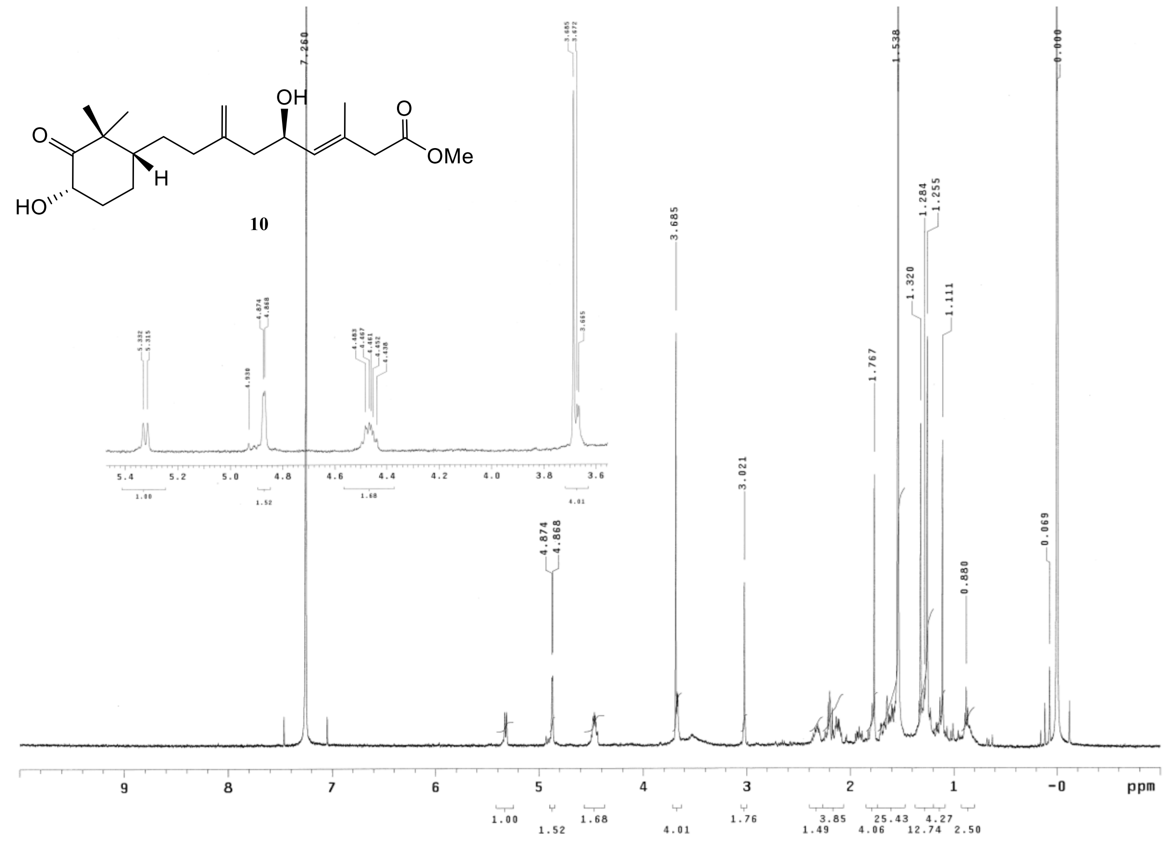

Figure S65. ^{1}H NMR spectrum of **10** in $CDCl_3$ at 500 MHz

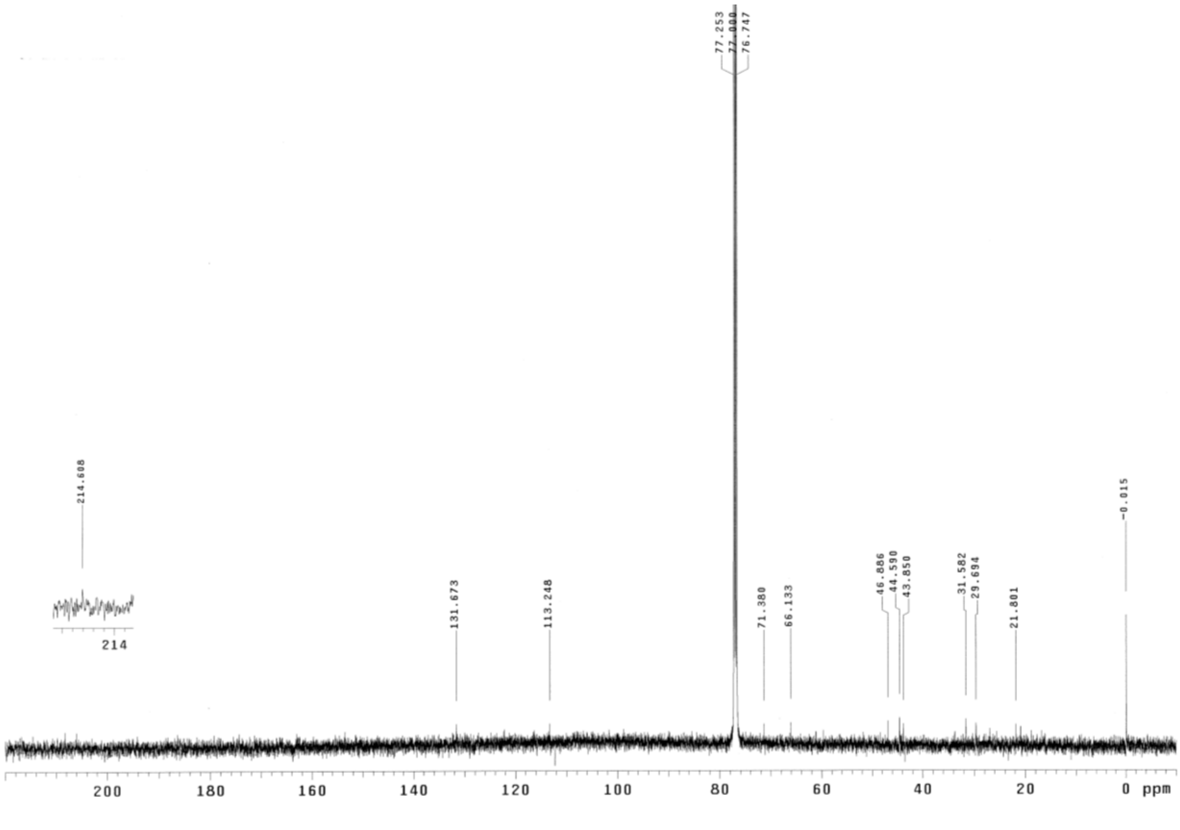

Figure S66. ^{13}C NMR spectrum of **10** in $CDCl_3$ at 125 MHz

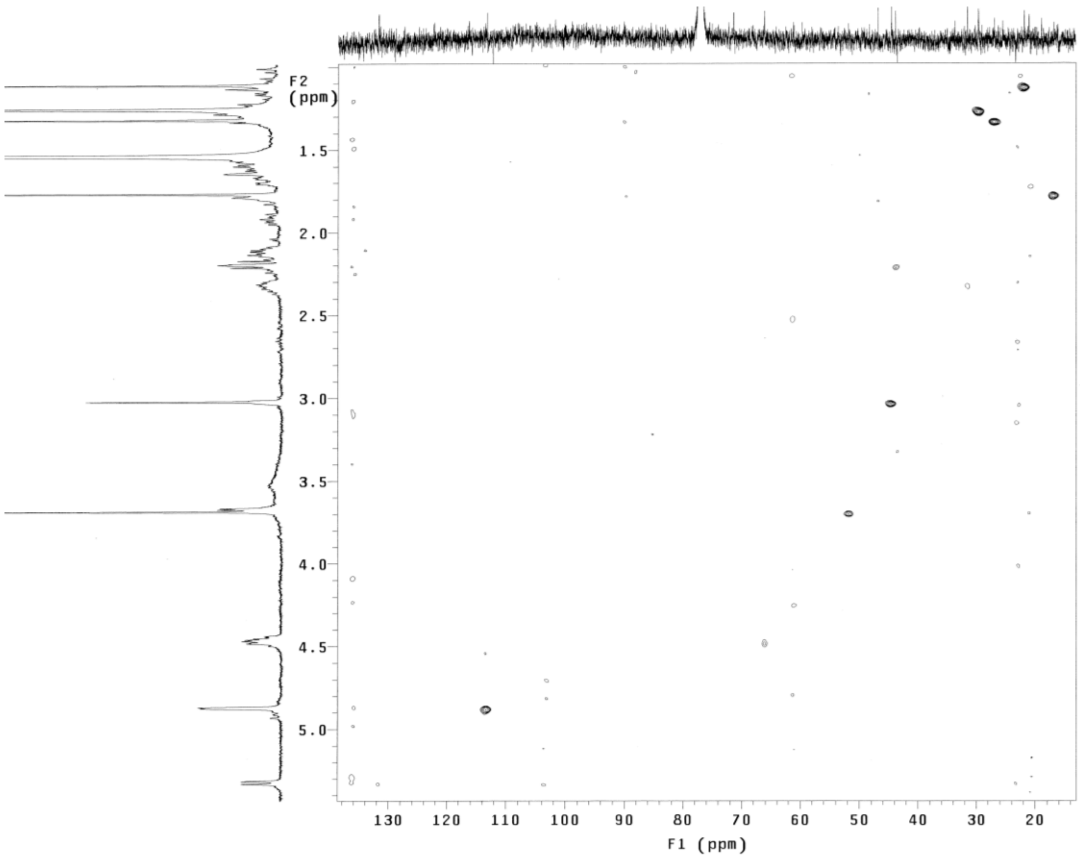

Figure S67. HSQC spectrum of **10** in $CDCl_3$

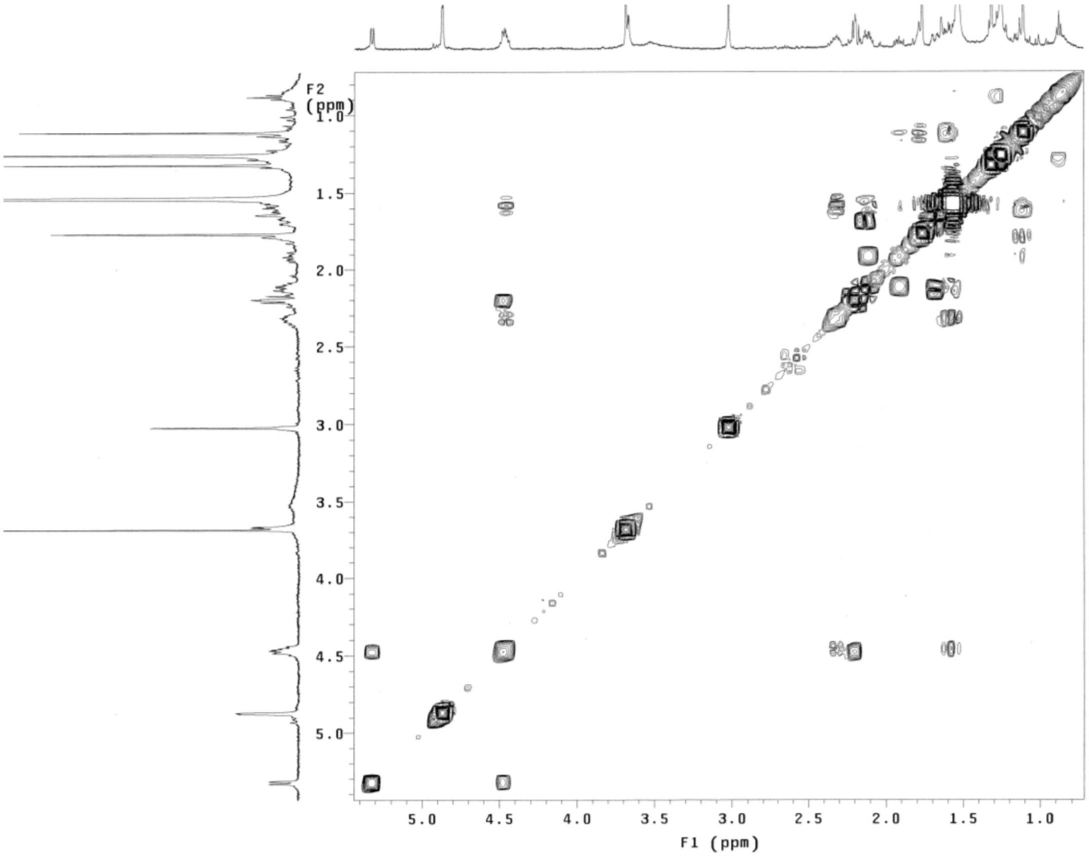

Figure S68. COSY spectrum of **10** in $CDCl_3$

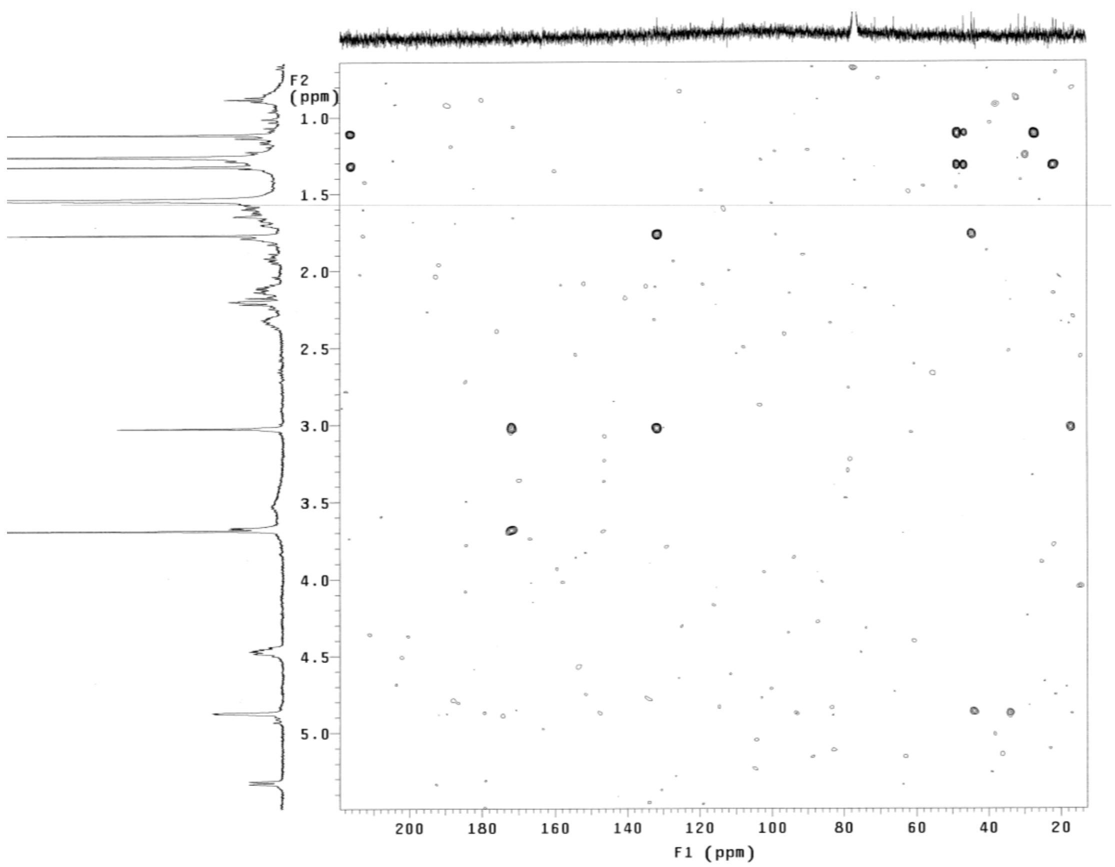

Figure S69. HMBC spectrum of **10** in $CDCl_3$

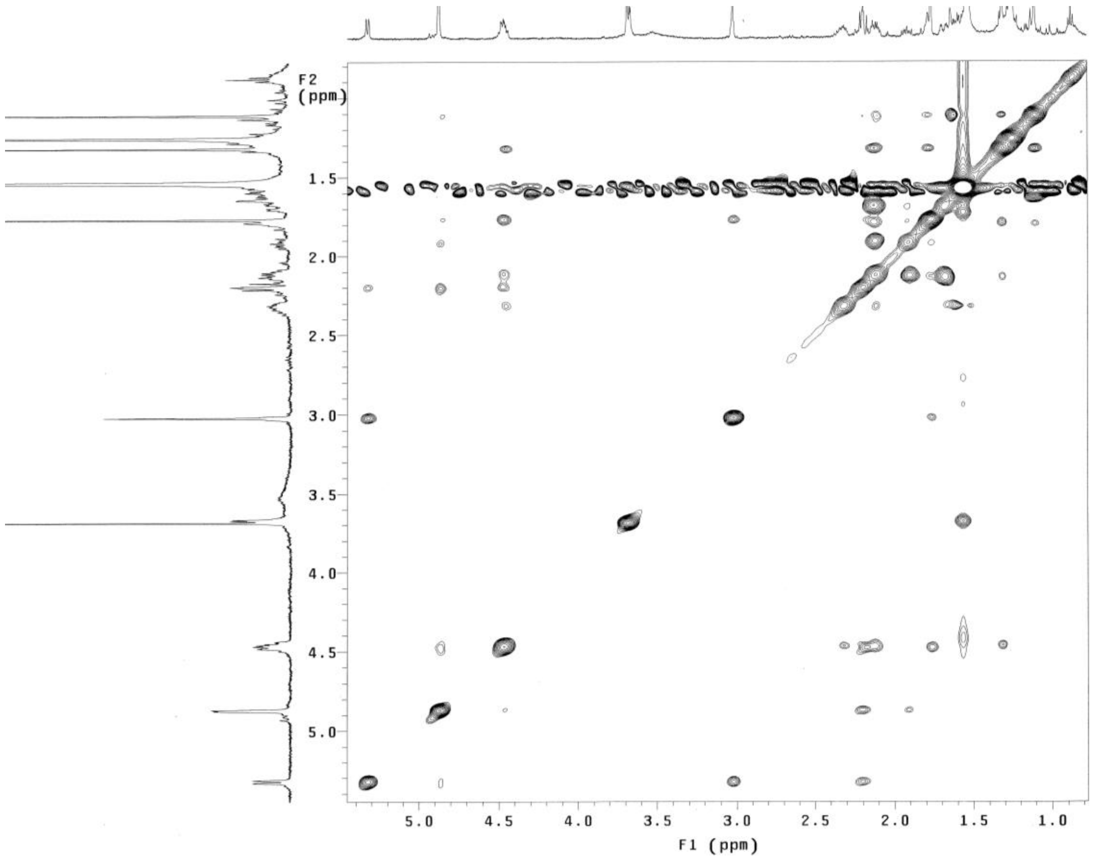

Figure S70. NOESY spectrum of **10** in $CDCl_3$

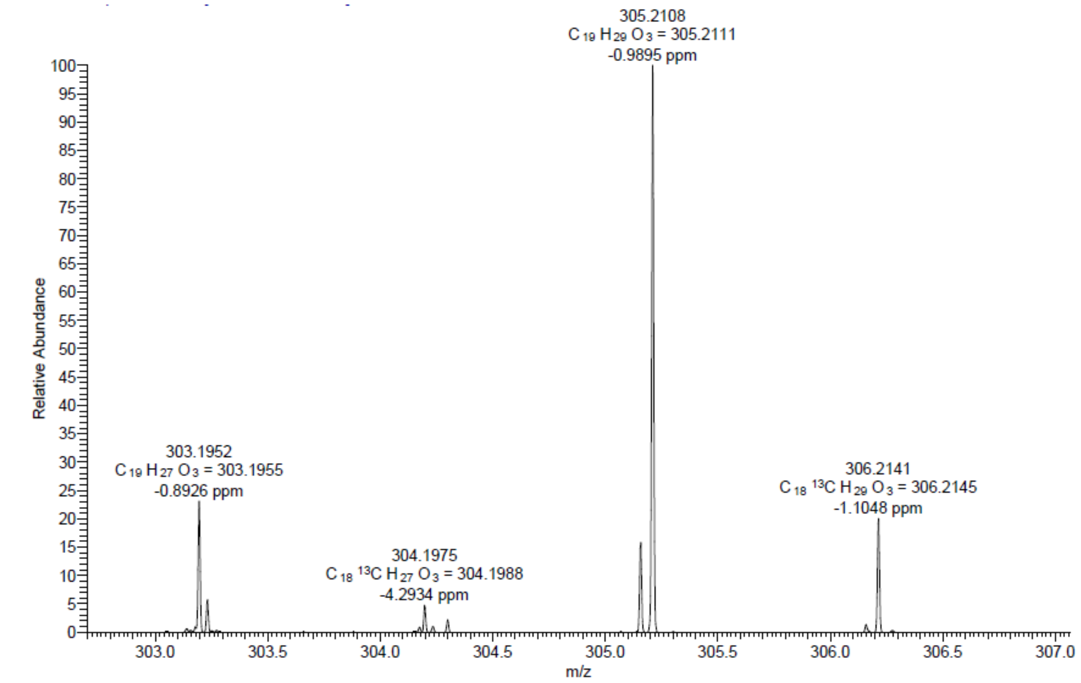

Figure S71. HRESIMS spectrum of **11**

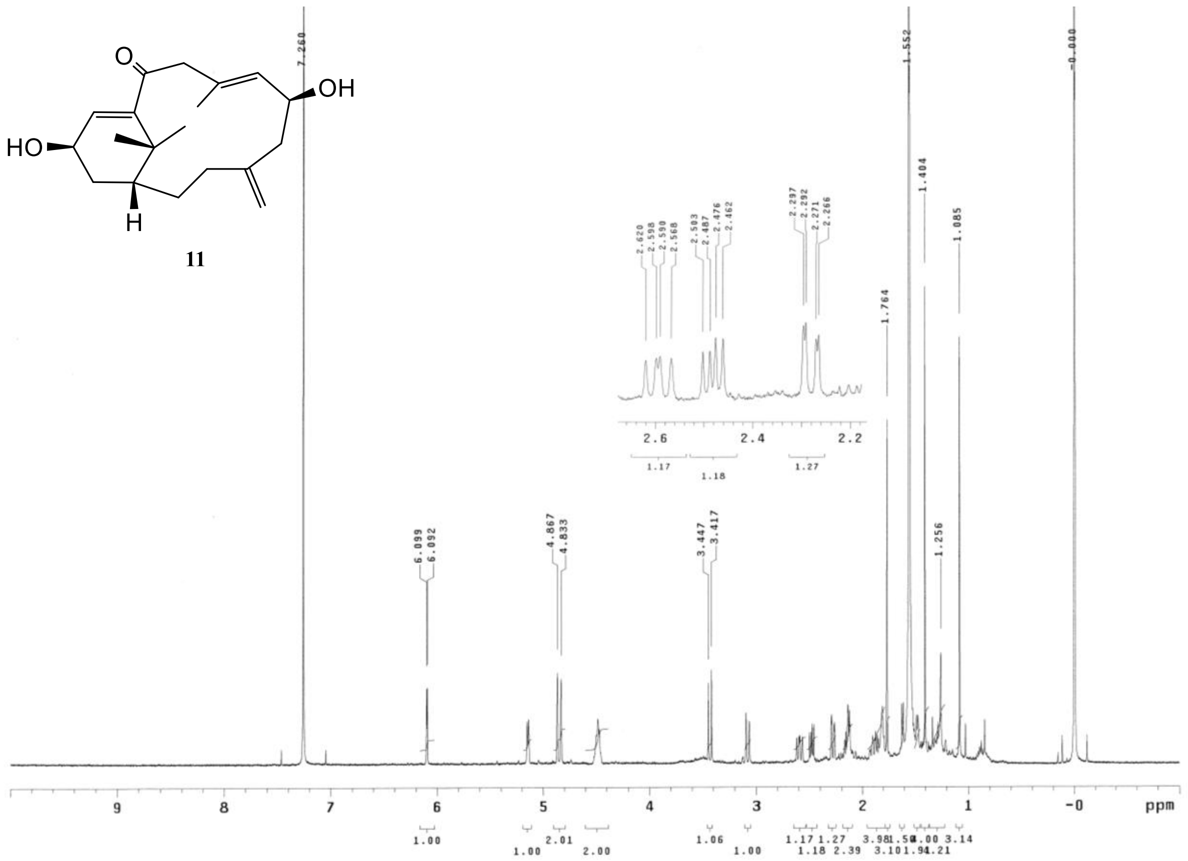

Figure S72. ^{1}H NMR spectrum of **11** in $CDCl_3$ at 500 MHz

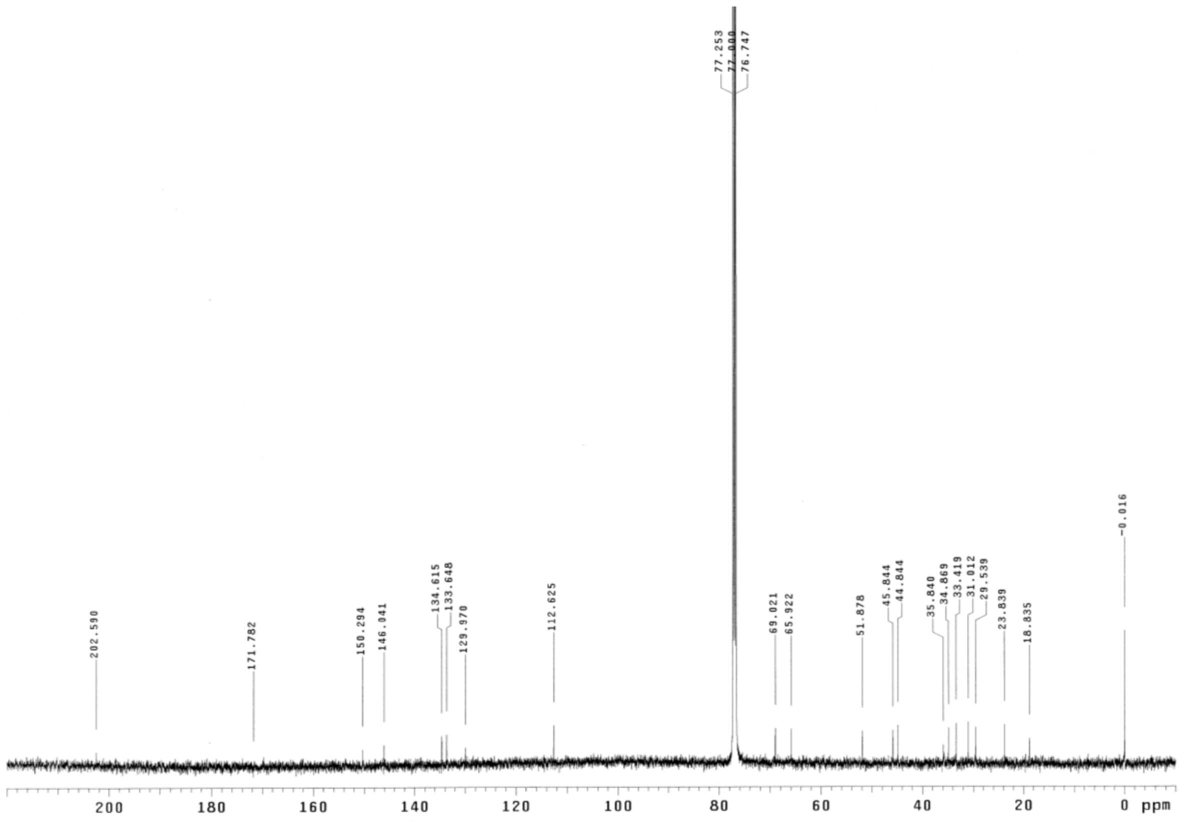

Figure S73. ^{13}C NMR spectrum of **11** in $CDCl_3$ at 125 MHz

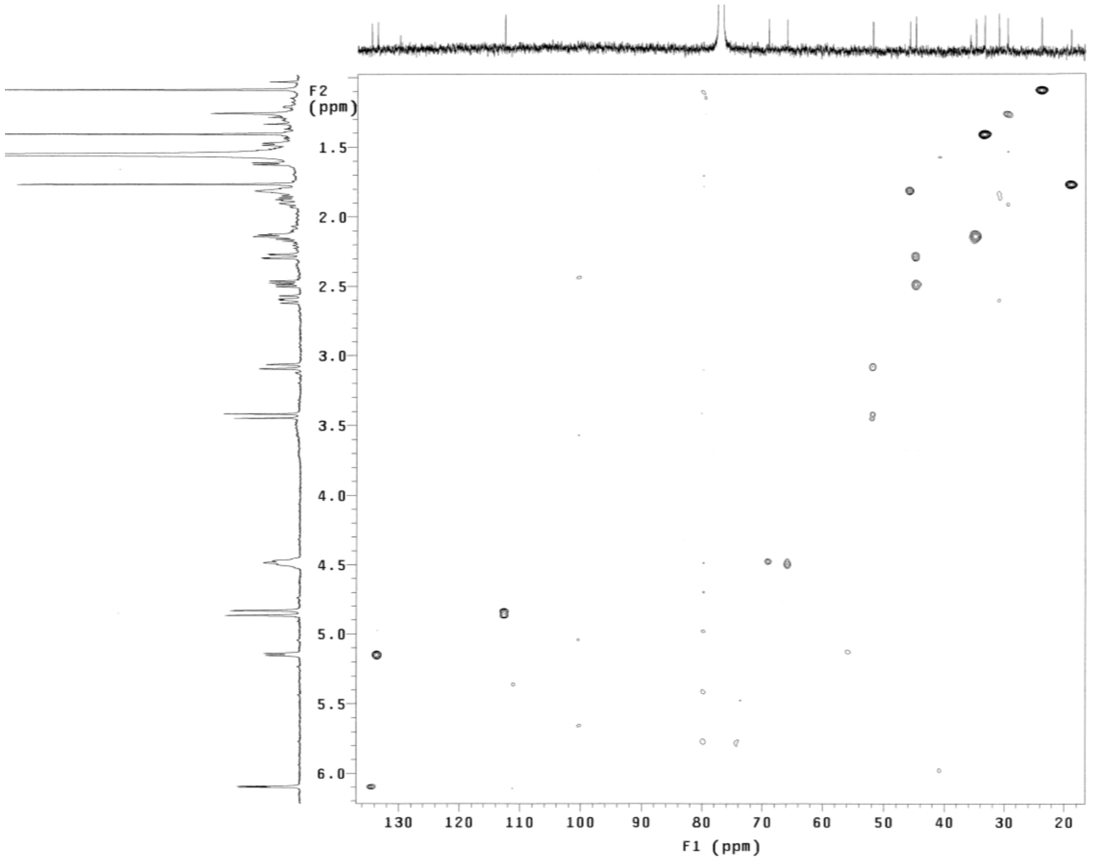

Figure S74. HSQC spectrum of **11** in $CDCl_3$

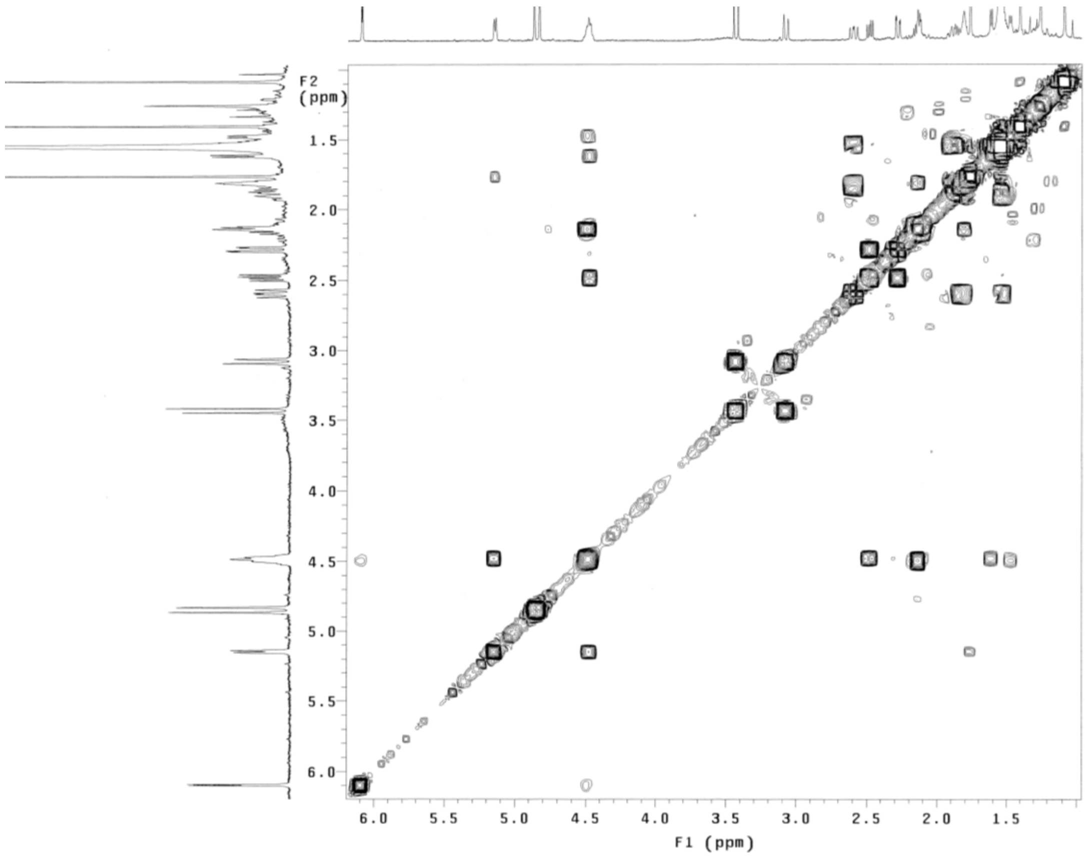

Figure S75. COSY spectrum of **11** in $CDCl_3$

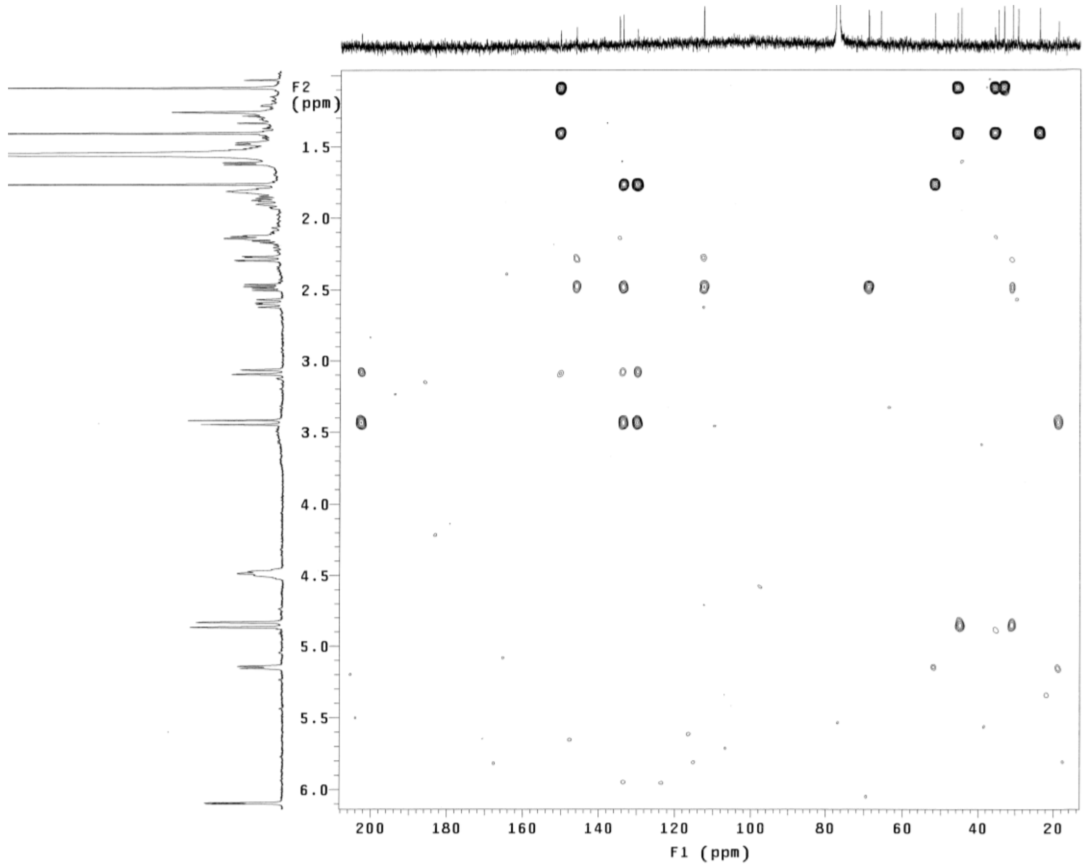

Figure S76. HMBC spectrum of **11** in $CDCl_3$

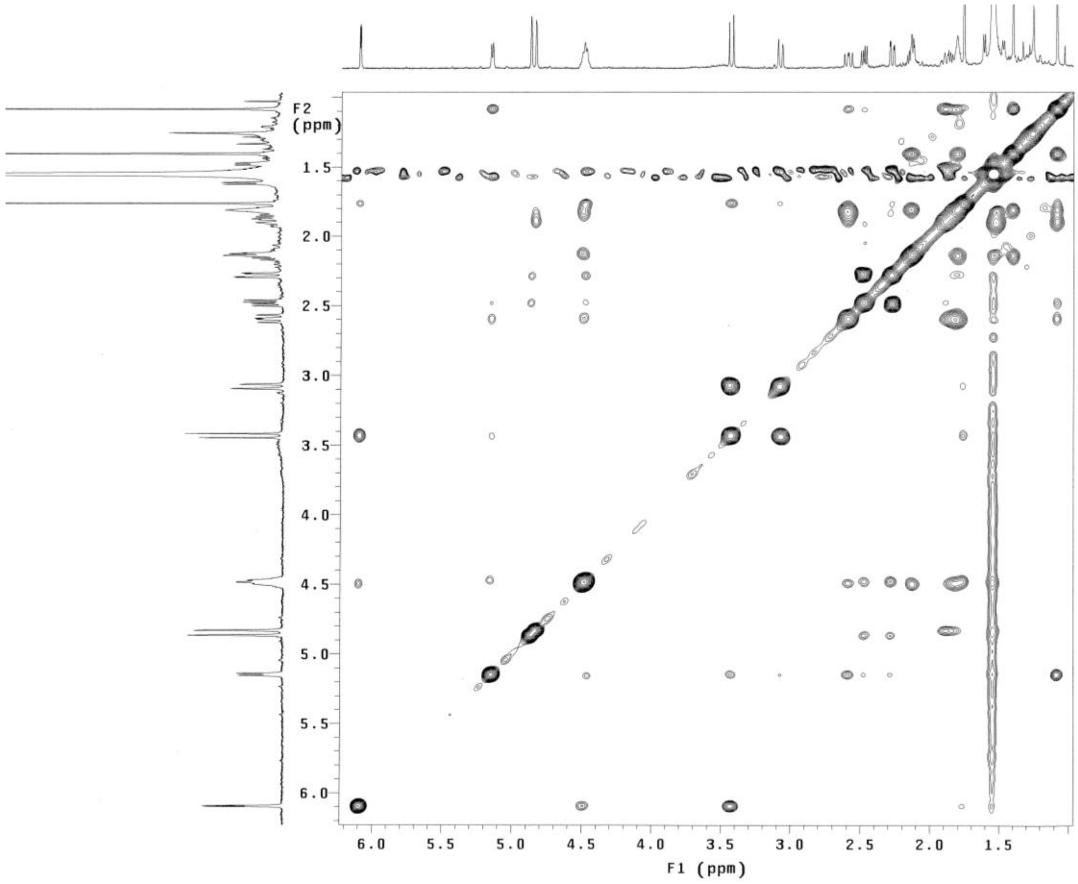

Figure S77. NOESY spectrum of **11** in $CDCl_3$

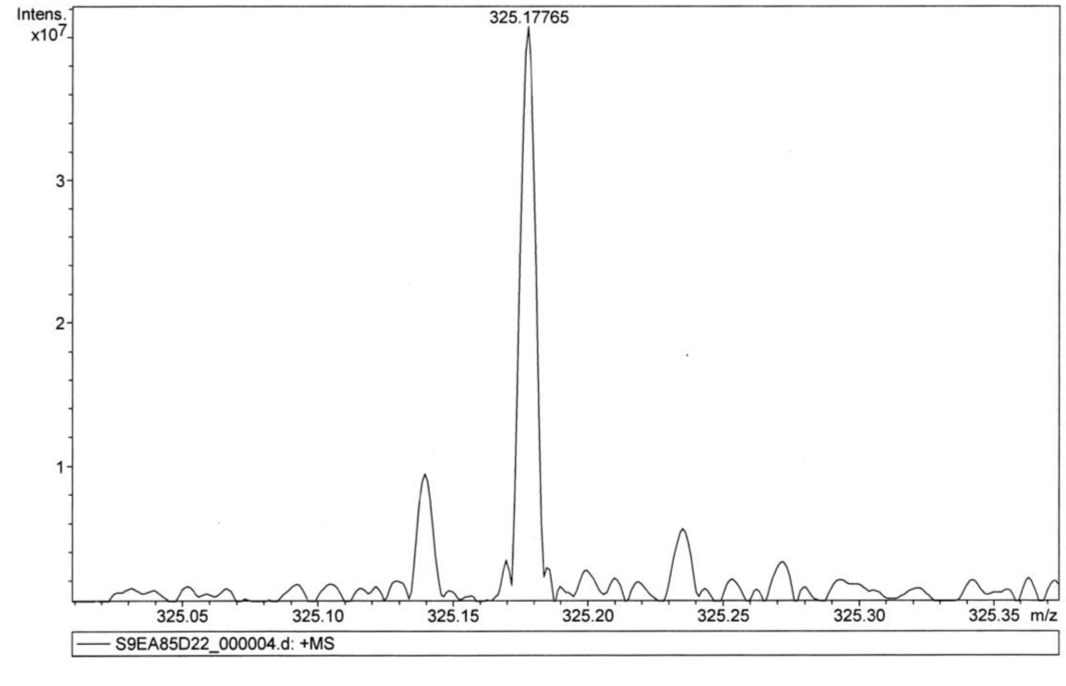

Meas. m/z	#	Formula	Score	m/z	err [mDa]	err [ppm]	mSigma	rdb	e⁻ Conf	N-Rule
325.17765	1	C 19 H 26 Na O 3	100.00	325.17742	-0.24	-0.73	15.1	6.5	even	ok

Figure S78. HRESIMS spectrum of **12**

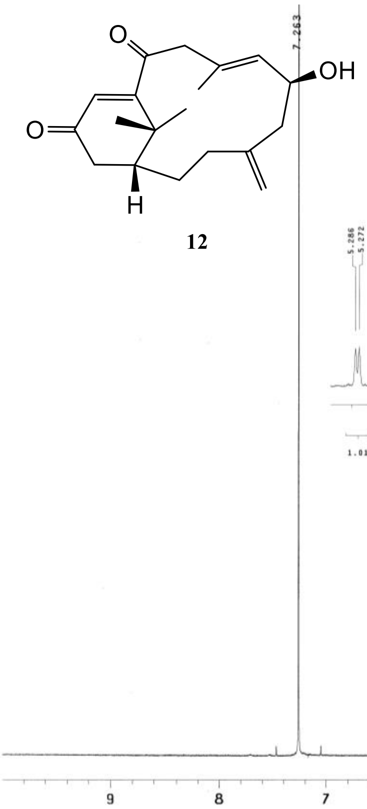

12

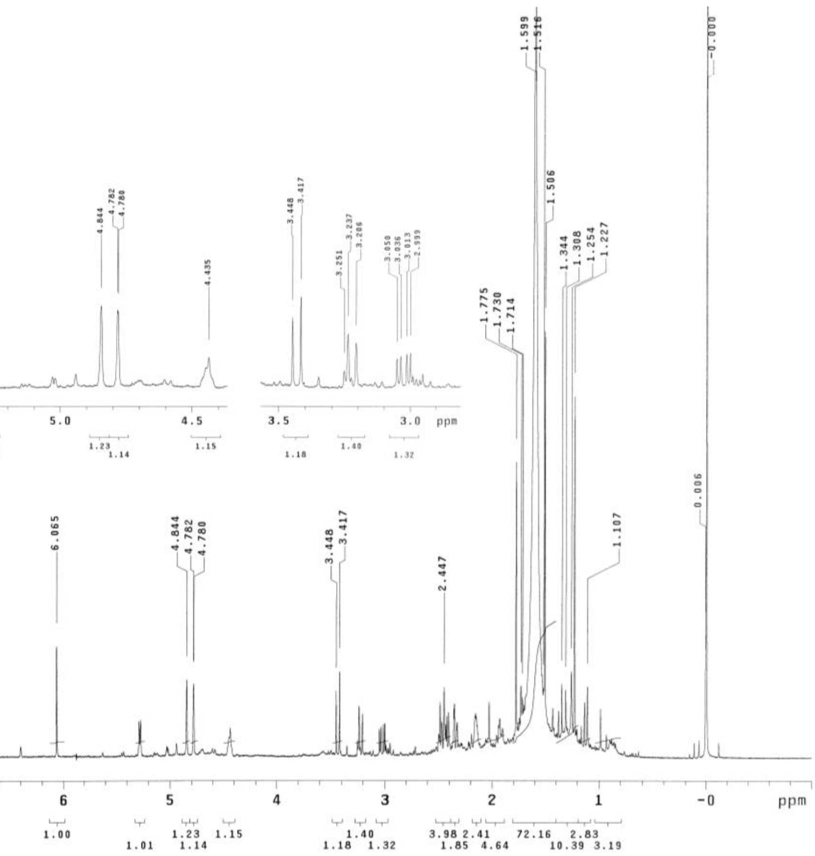

Figure S79. ^{1}H NMR spectrum of **12** in $CDCl_3$ at 500 MHz

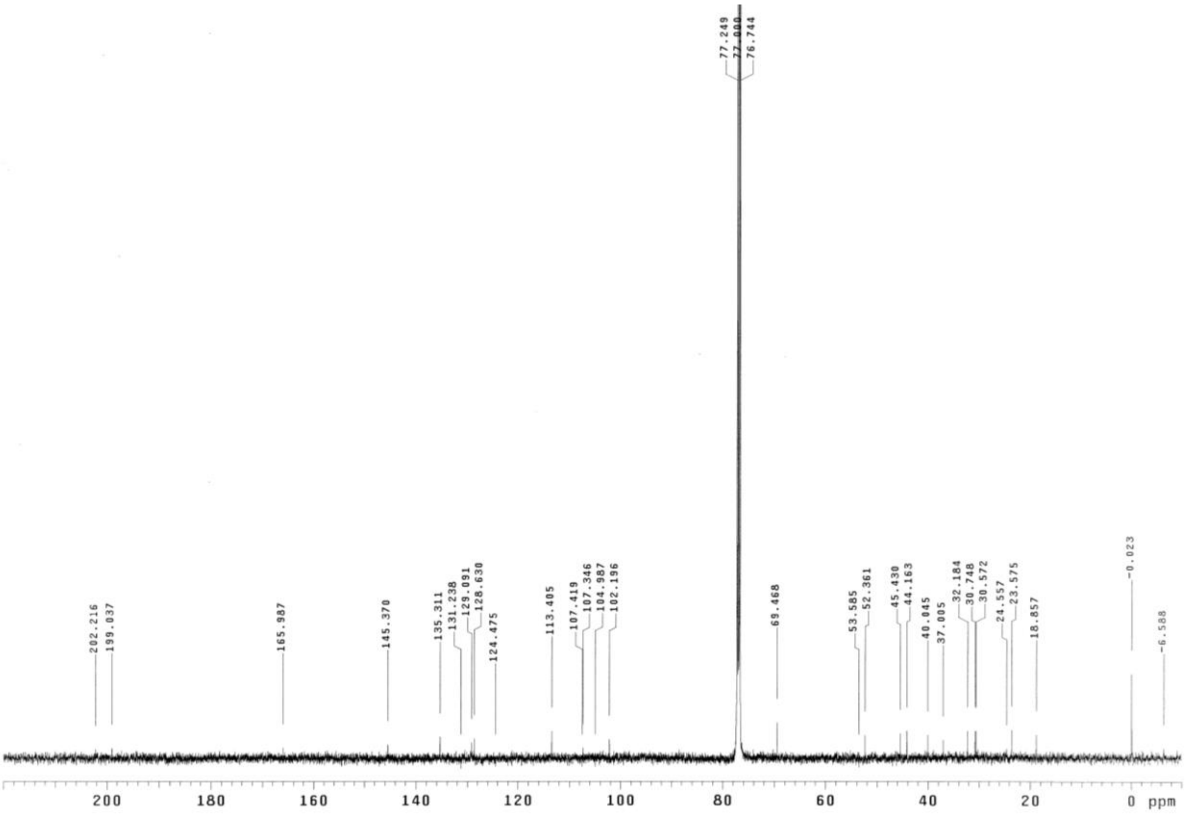

Figure S80. ^{13}C NMR spectrum of **12** in $CDCl_3$ at 125 MHz

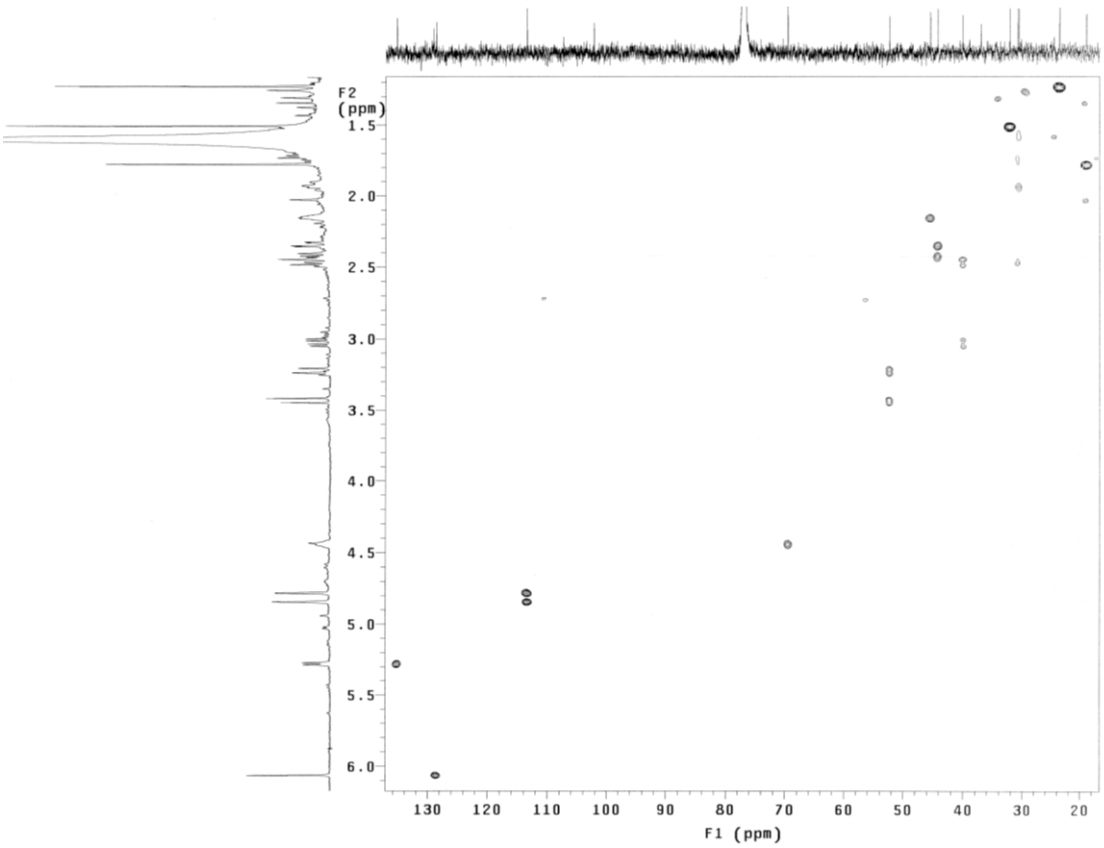

Figure S81. HSQC spectrum of **12** in $CDCl_3$

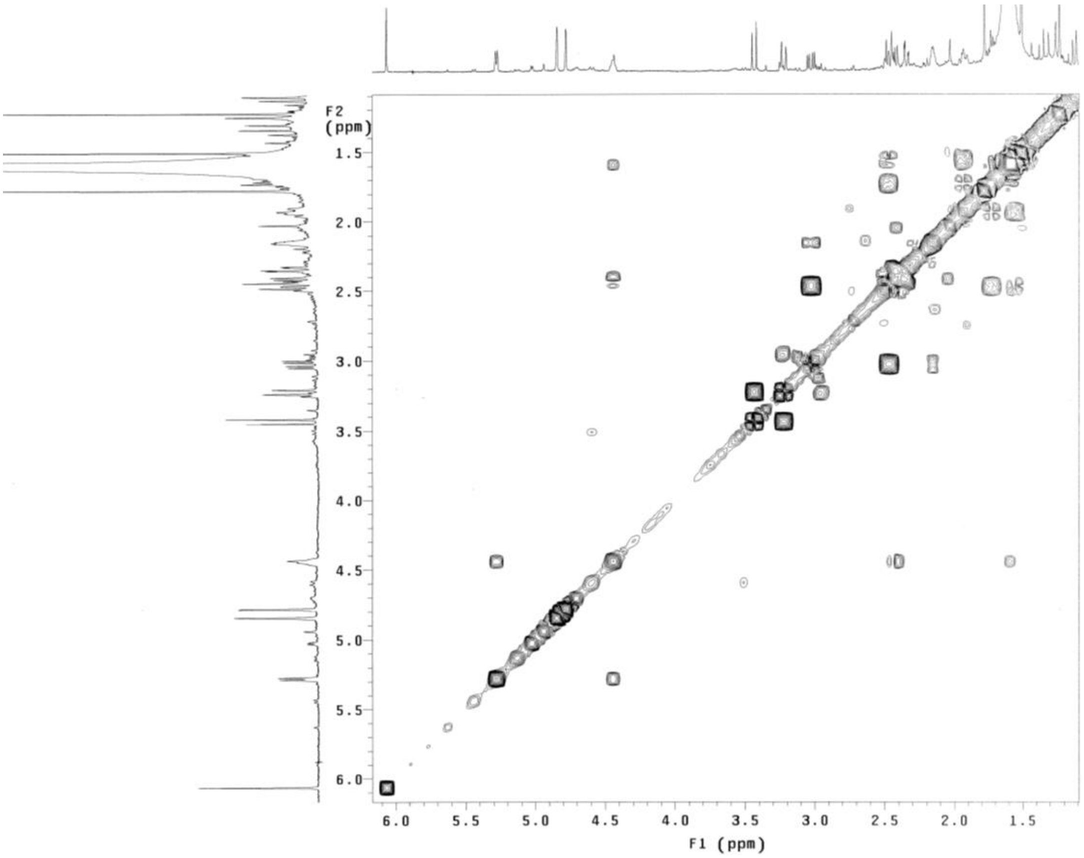

Figure S82. COSY spectrum of **12** in $CDCl_3$

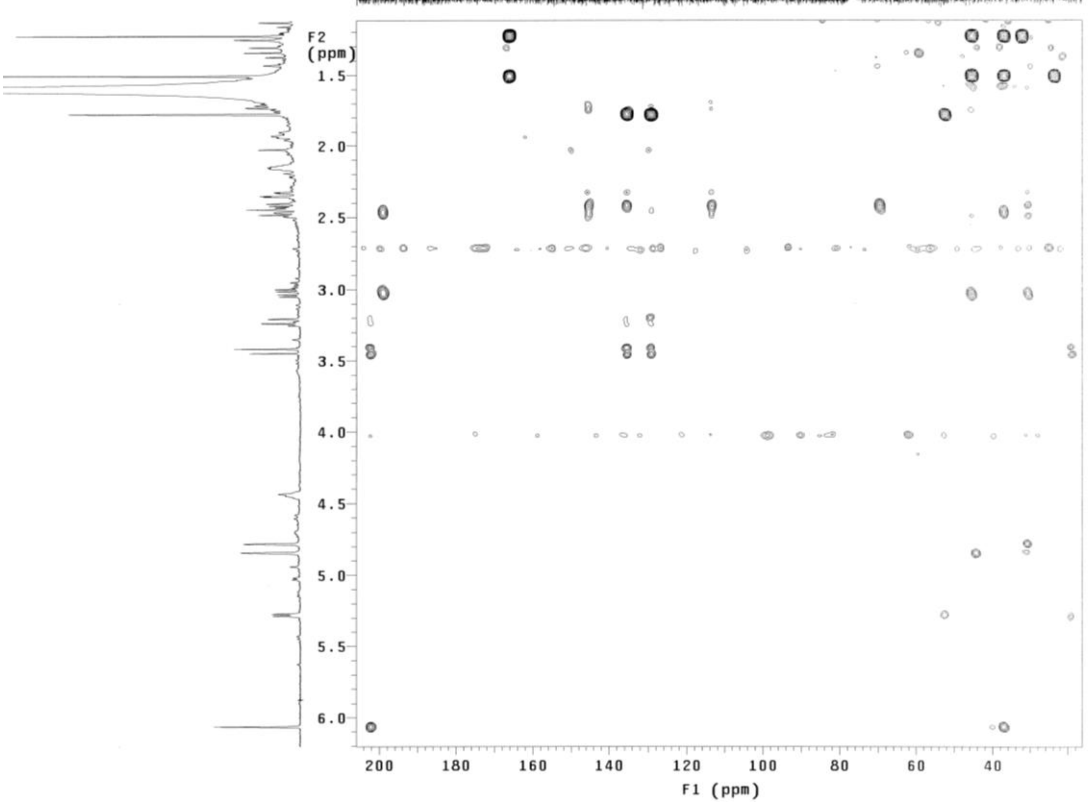

Figure S83. HMBC spectrum of **12** in $CDCl_3$

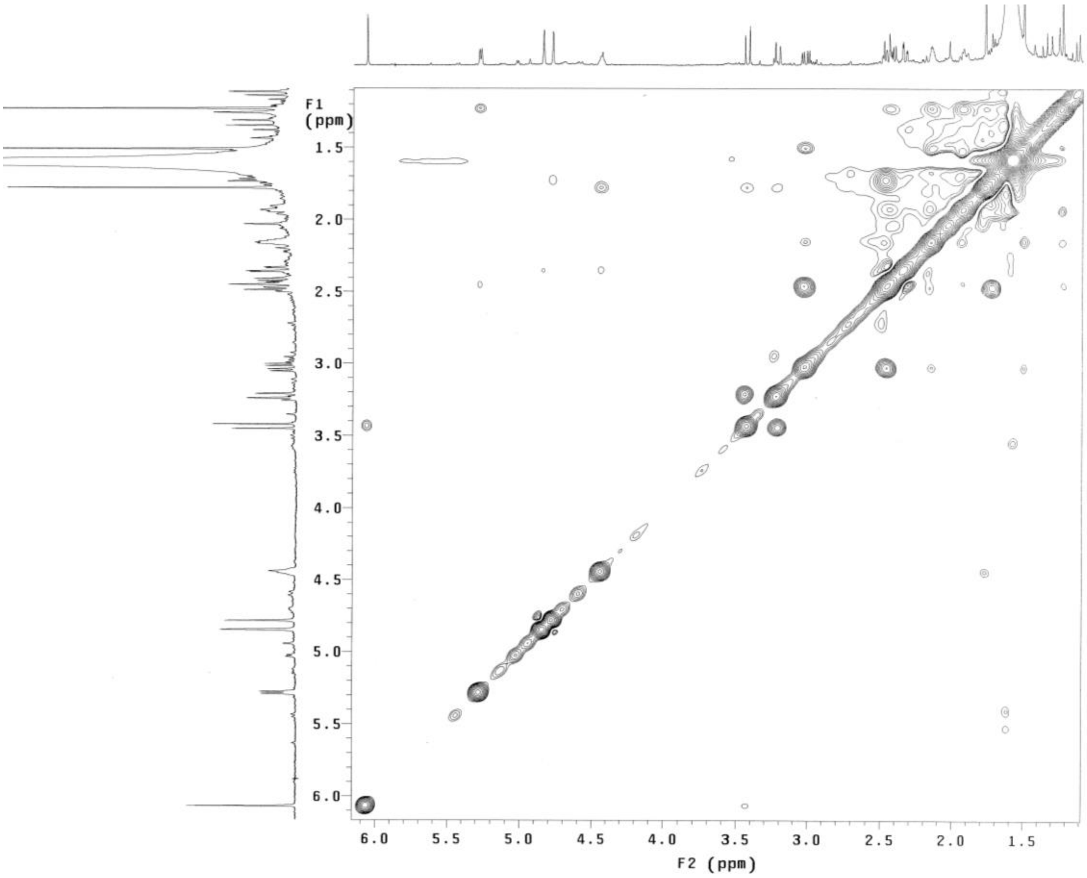

Figure S84. NOESY spectrum of **12** in $CDCl_3$

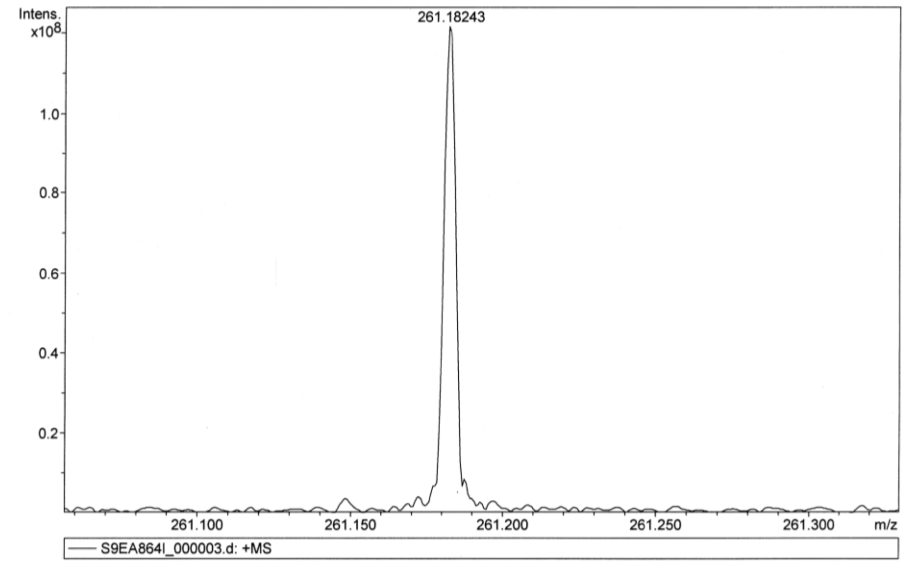

Meas. m/z	#	Formula	Score	m/z	err [mDa]	err [ppm]	mSigma	rdb	e⁻ Conf	N-Rule
261.18243	1	C 15 H 26 Na O 2	100.00	261.18250	0.07	0.29	10.1	2.5	even	ok

Figure S85. HRESIMS spectrum of **13**

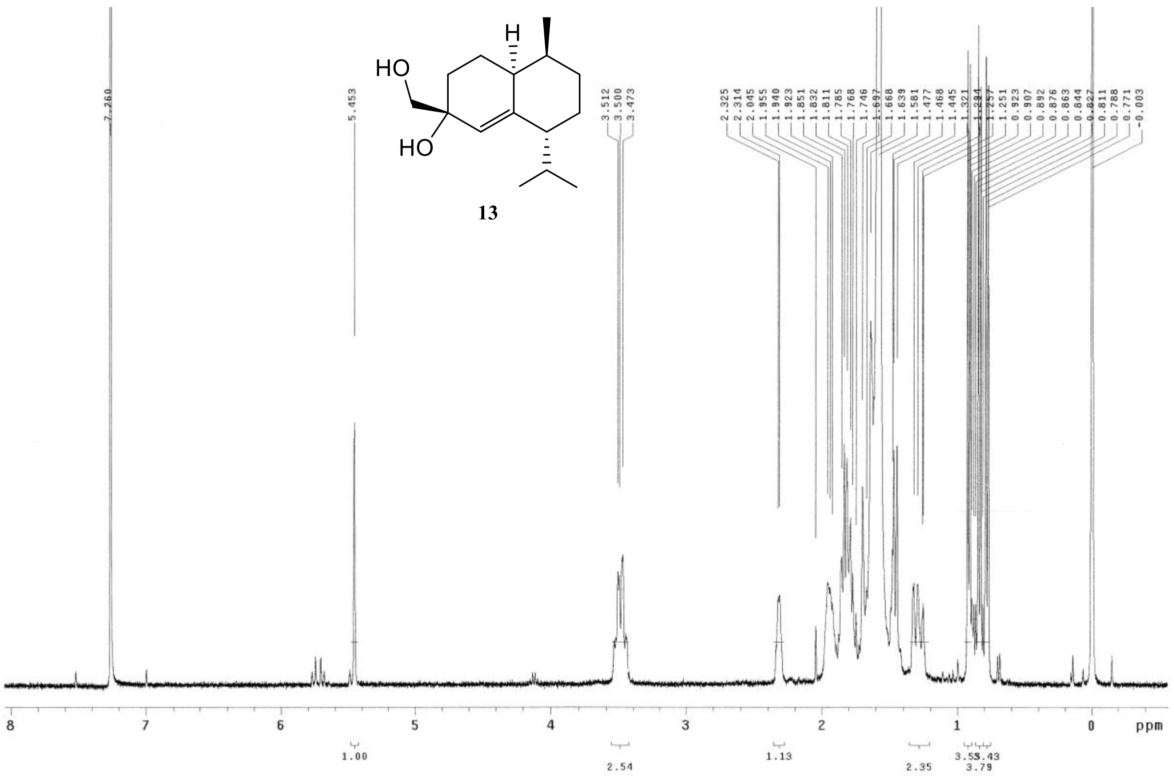

Figure S86. ^{1}H NMR spectrum of **13** in $CDCl_3$ at 400 MHz

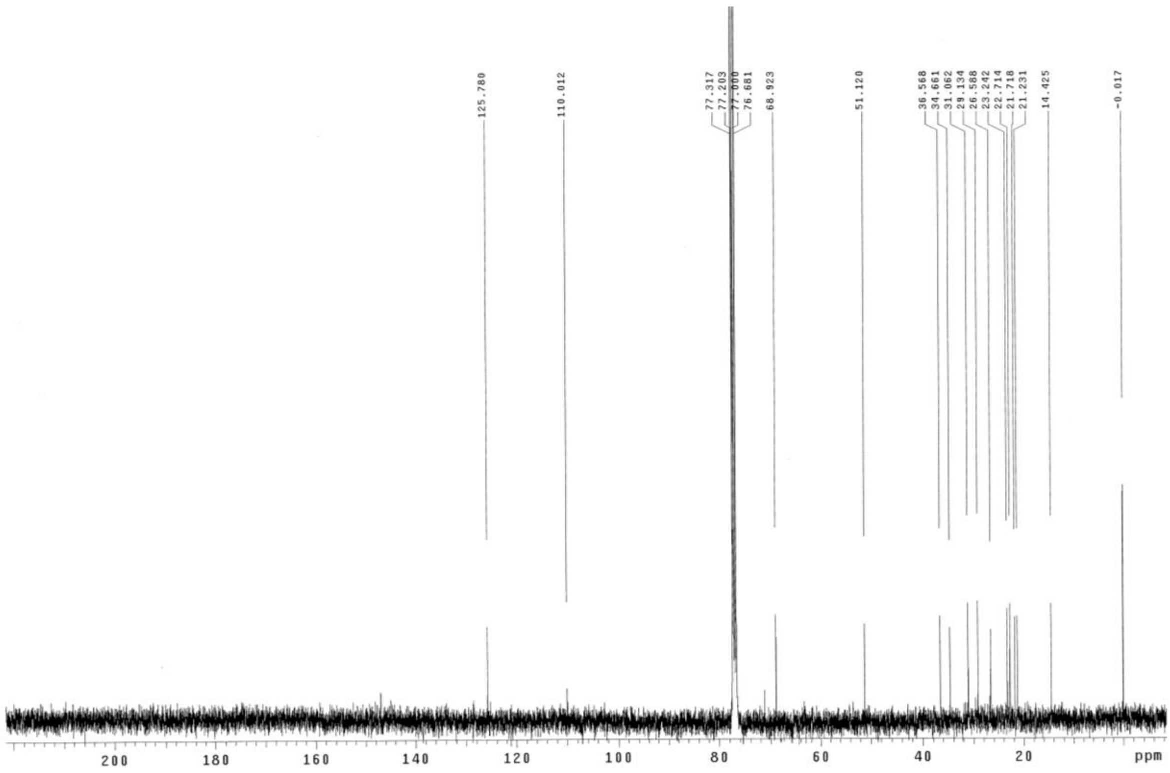

Figure S87. ^{13}C NMR spectrum of **13** in $CDCl_3$ at 100 MHz

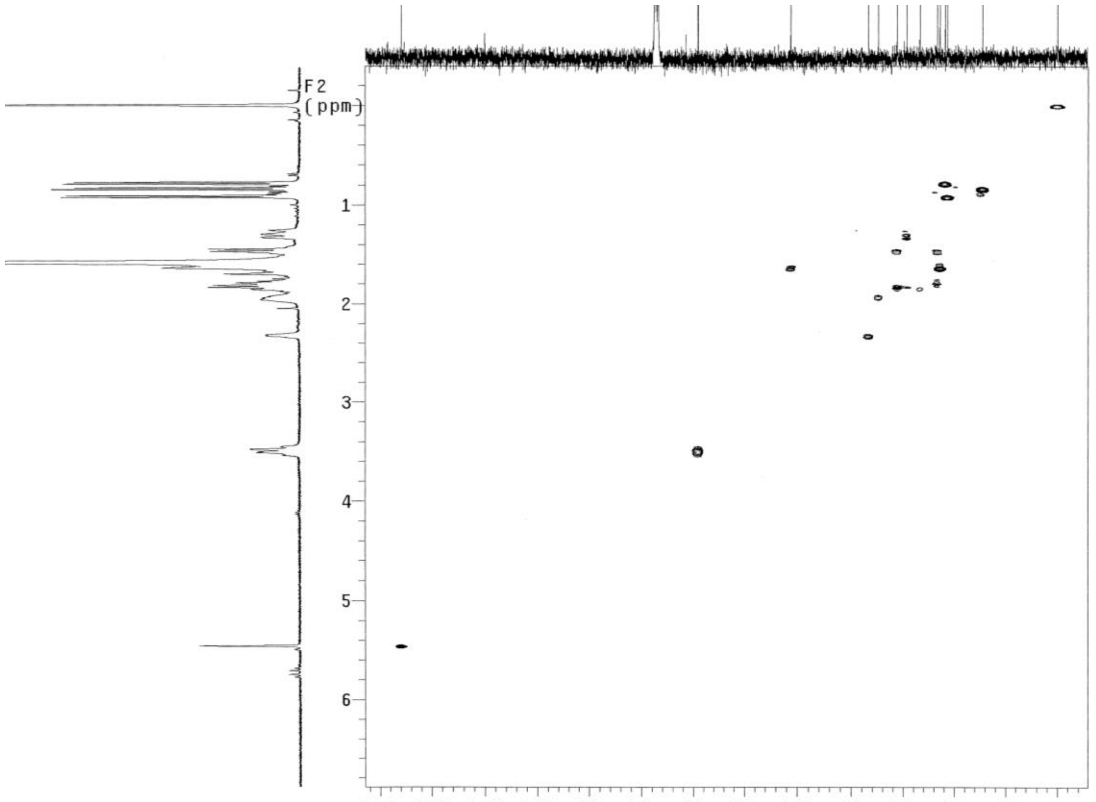

Figure S88. HSQC spectrum of **13** in $CDCl_3$

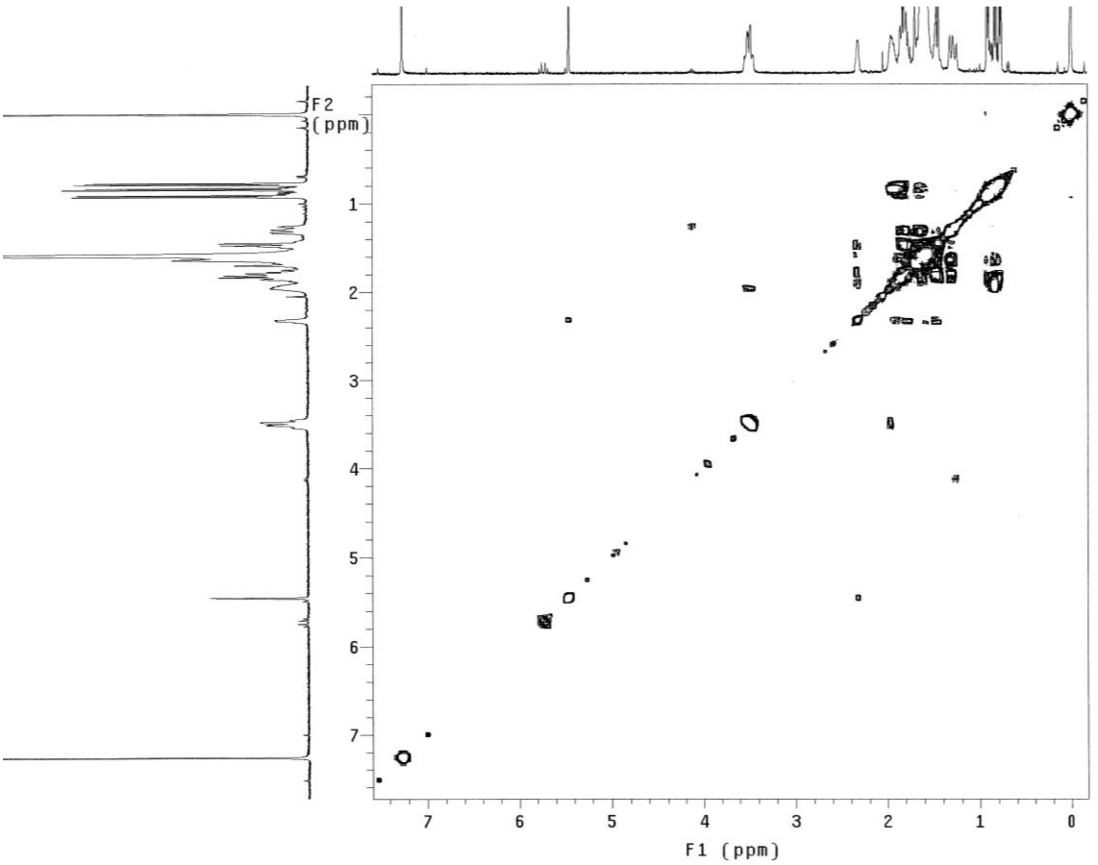

Figure S89. COSY spectrum of **13** in $CDCl_3$

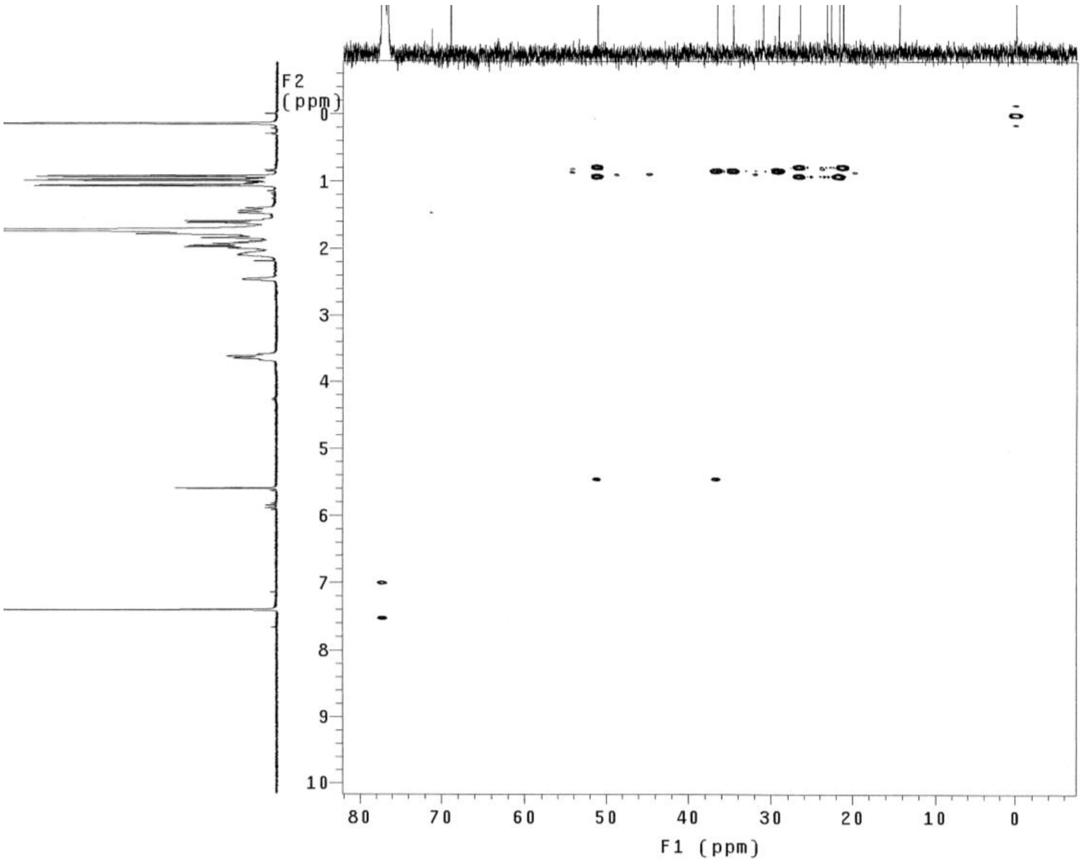

Figure S90. HMBC spectrum of **13** in $CDCl_3$

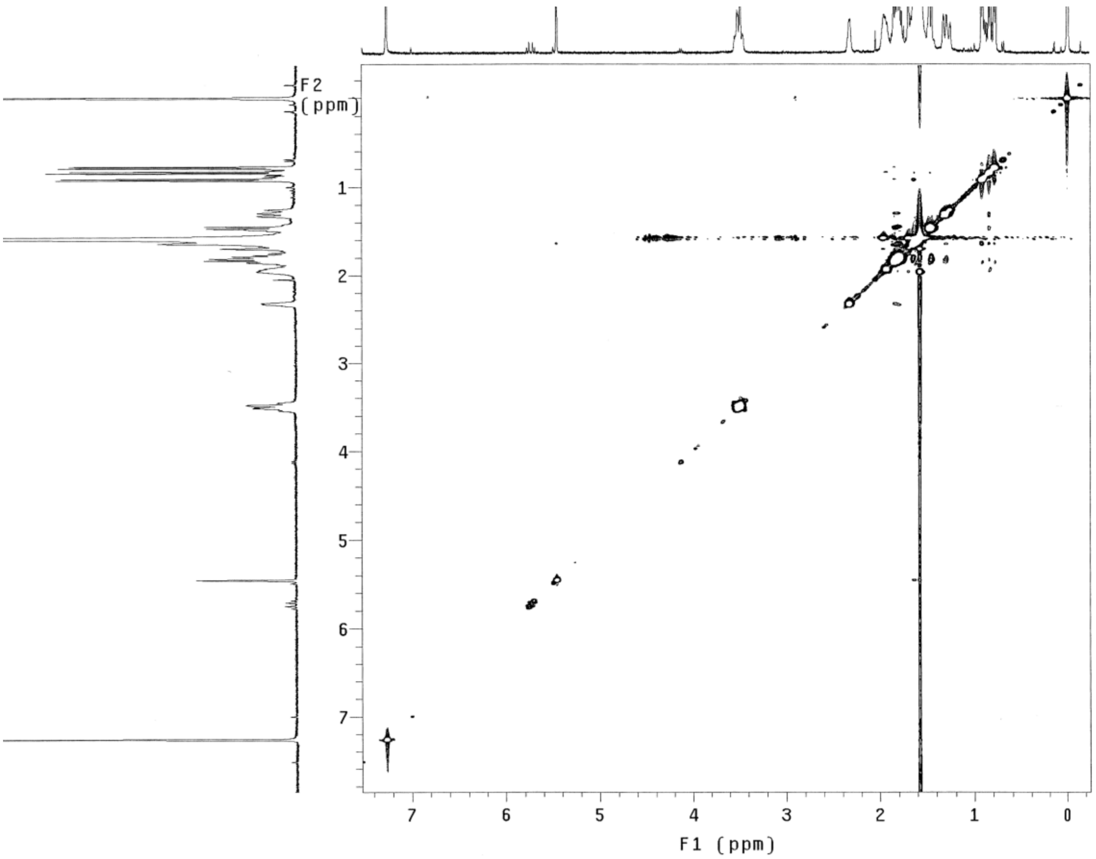

Figure S91. NOESY spectrum of **13** in $CDCl_3$

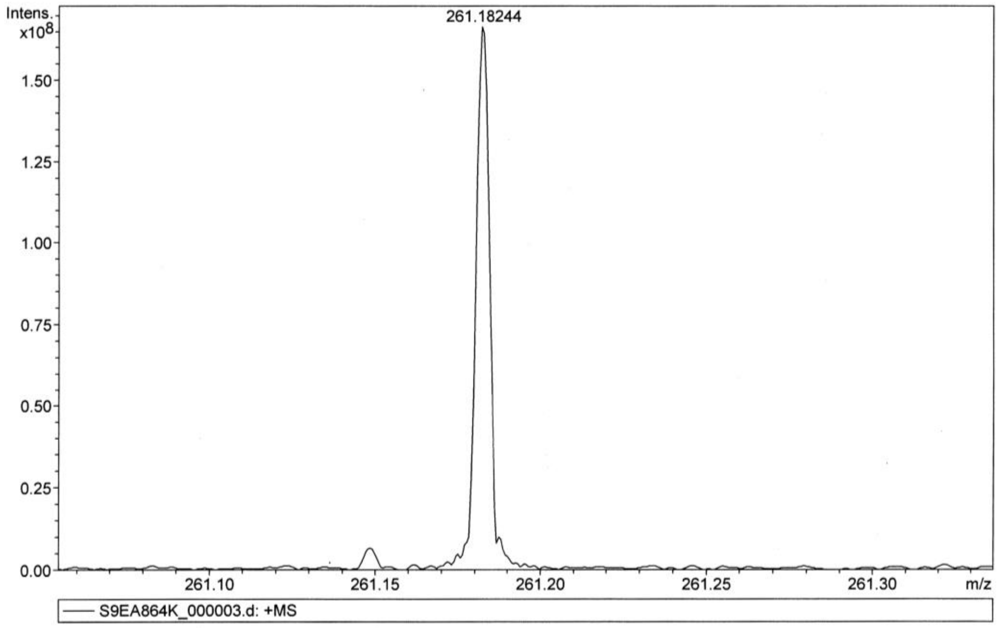

Meas. m/z	#	Formula	Score	m/z	err [mDa]	err [ppm]	mSigma	rdb	e⁻ Conf	N-Rule
261.18244	1	C 15 H 26 Na O 2	100.00	261.18250	0.06	0.24	9.7	2.5	even	ok

Figure S92. HRESIMS spectrum of **14**

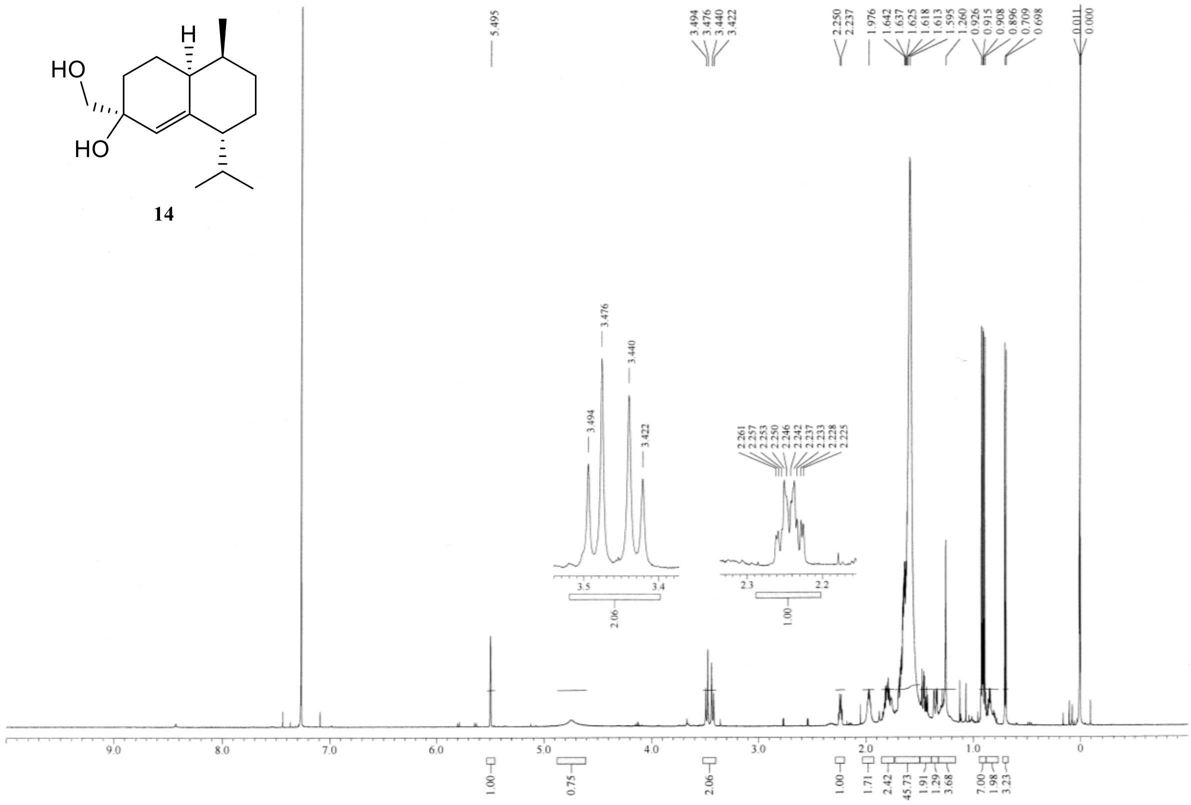

Figure S93. ^{1}H NMR spectrum of **14** in $CDCl_3$ at 600 MHz

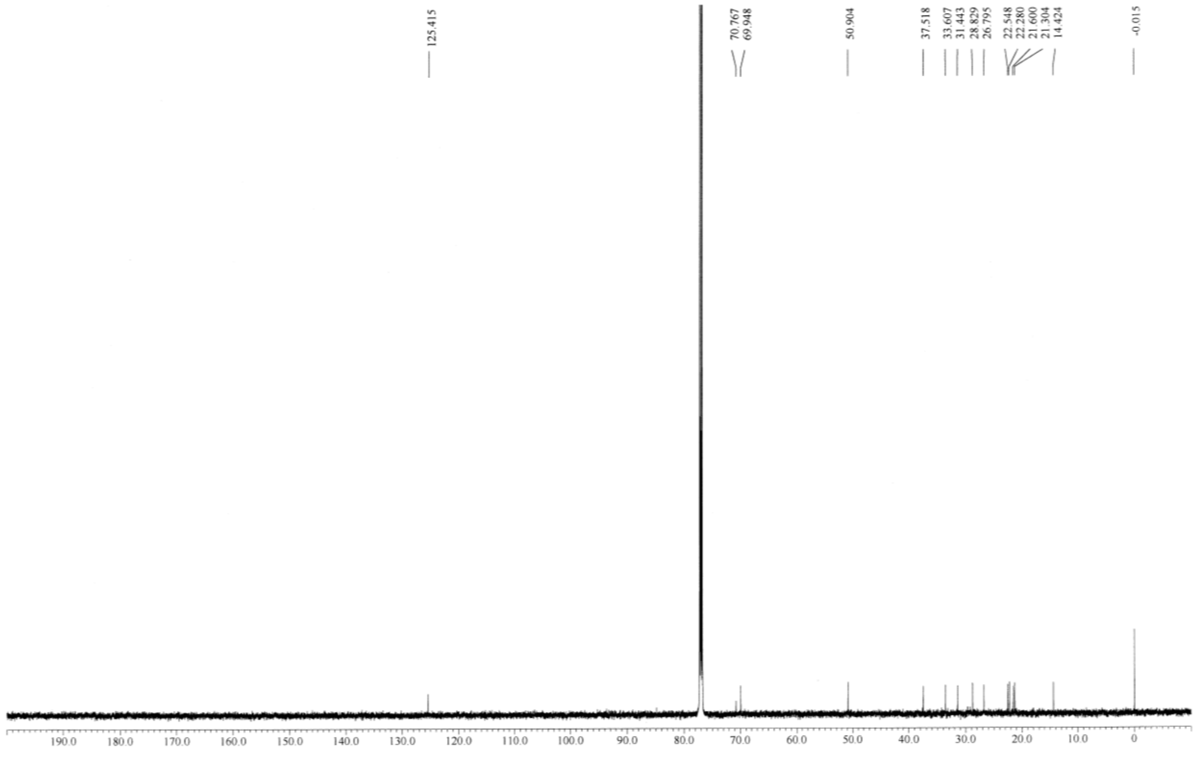

Figure S94. ^{13}C NMR spectrum of **14** in $CDCl_3$ at 150 MHz

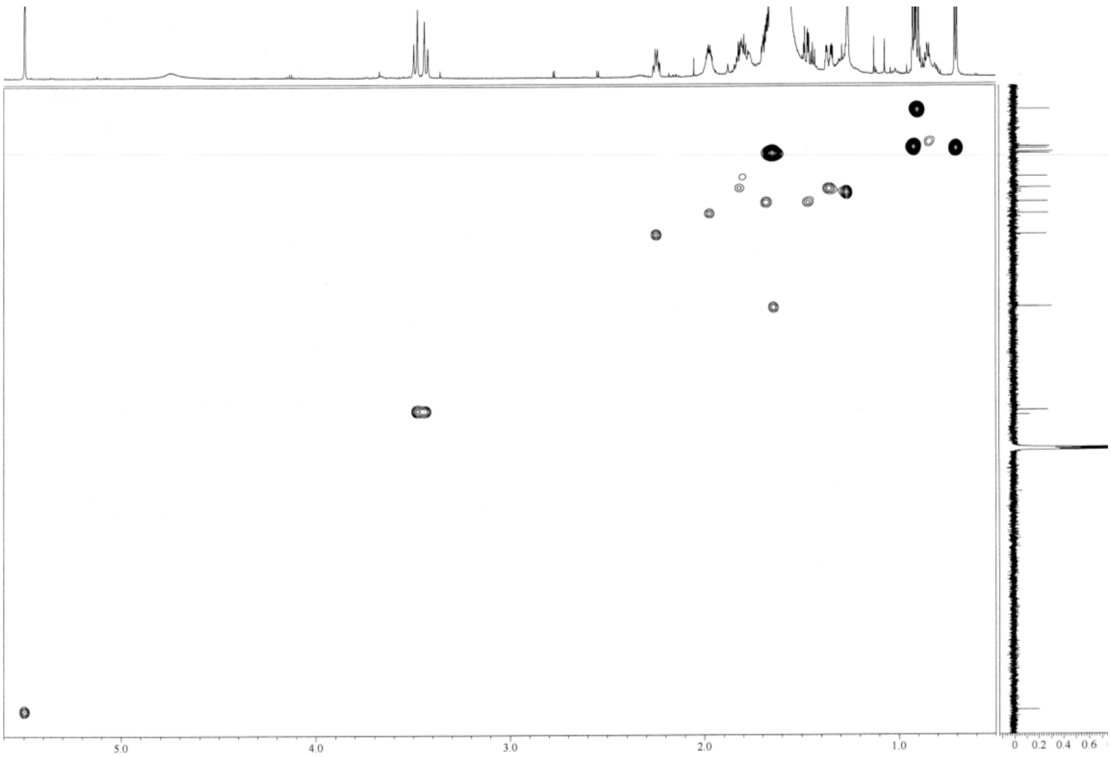

Figure S95. HSQC spectrum of **14** in $CDCl_3$

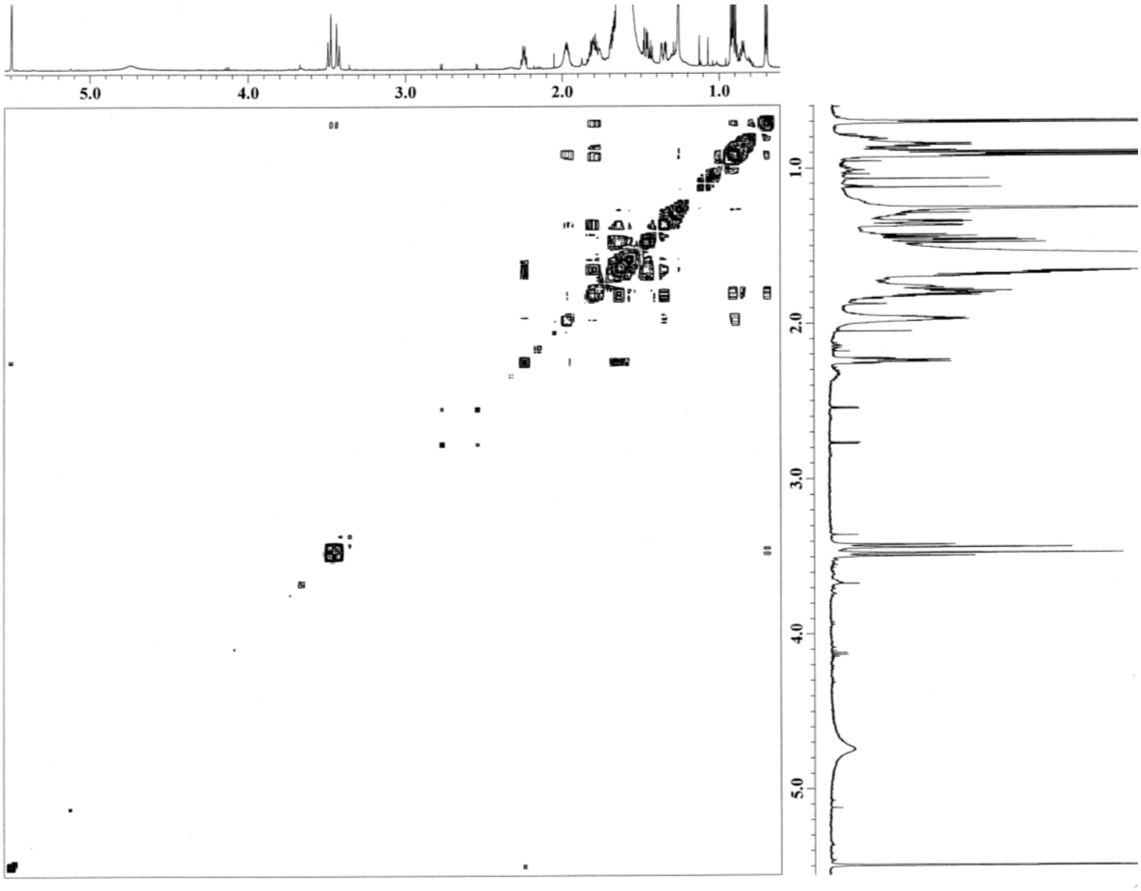

Figure S96. COSY spectrum of **14** in $CDCl_3$

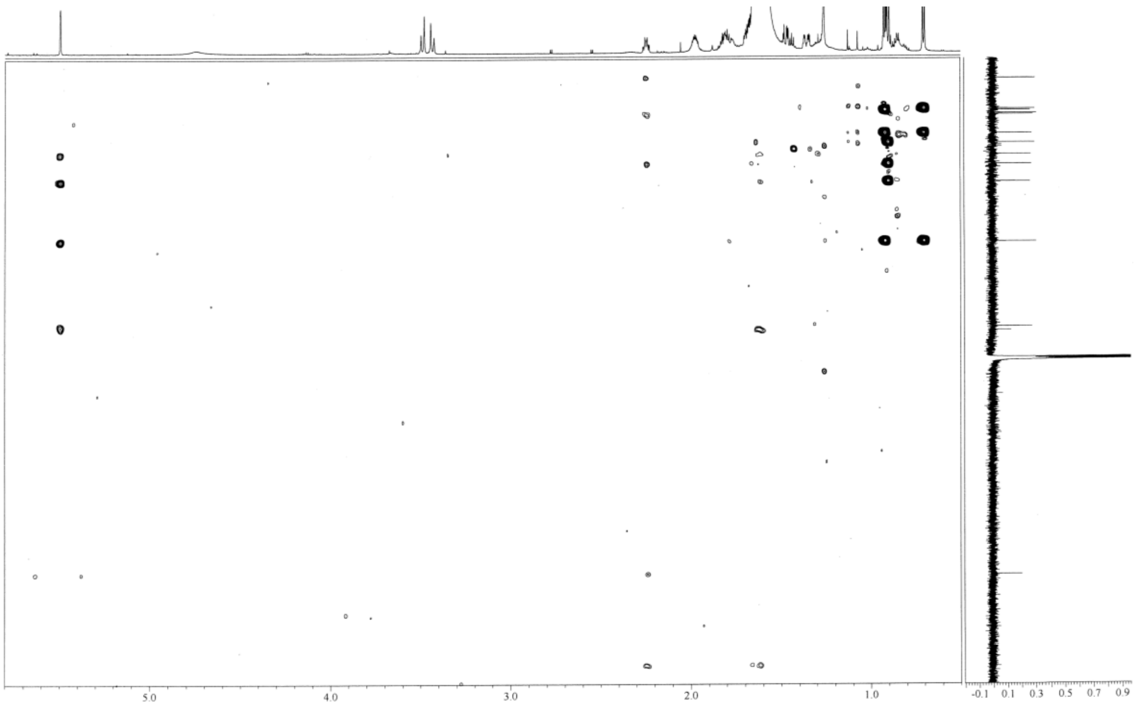

Figure S97. HMBC spectrum of **14** in $CDCl_3$

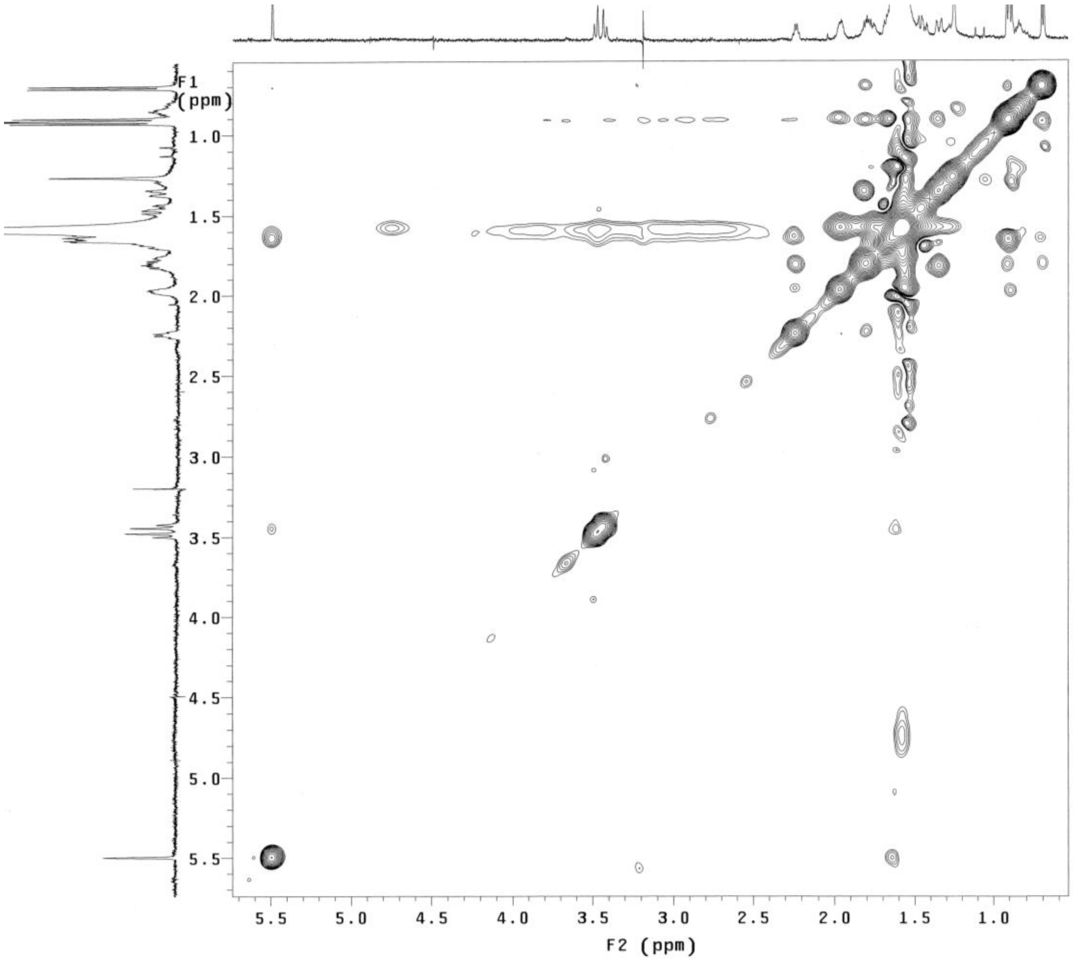

Figure S98. NOESY spectrum of **14** in $CDCl_3$

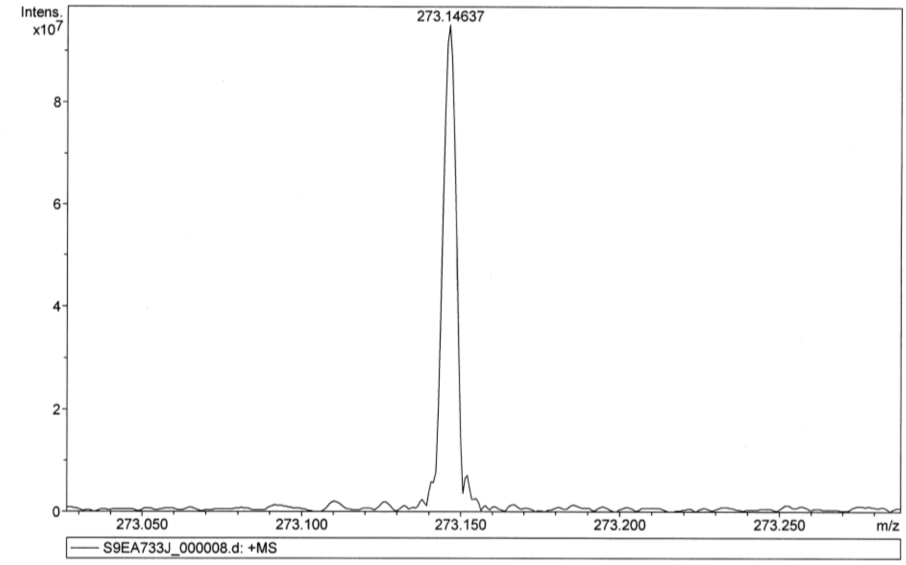

Meas. m/z	#	Formula	Score	m/z	err [mDa]	err [ppm]	mSigma	rdb	e⁻ Conf	N-Rule
273.14637	1	C 15 H 22 Na O 3	100.00	273.14612	-0.25	-0.92	15.0	4.5	even	ok

Figure S99. HRESIMS spectrum of **15**

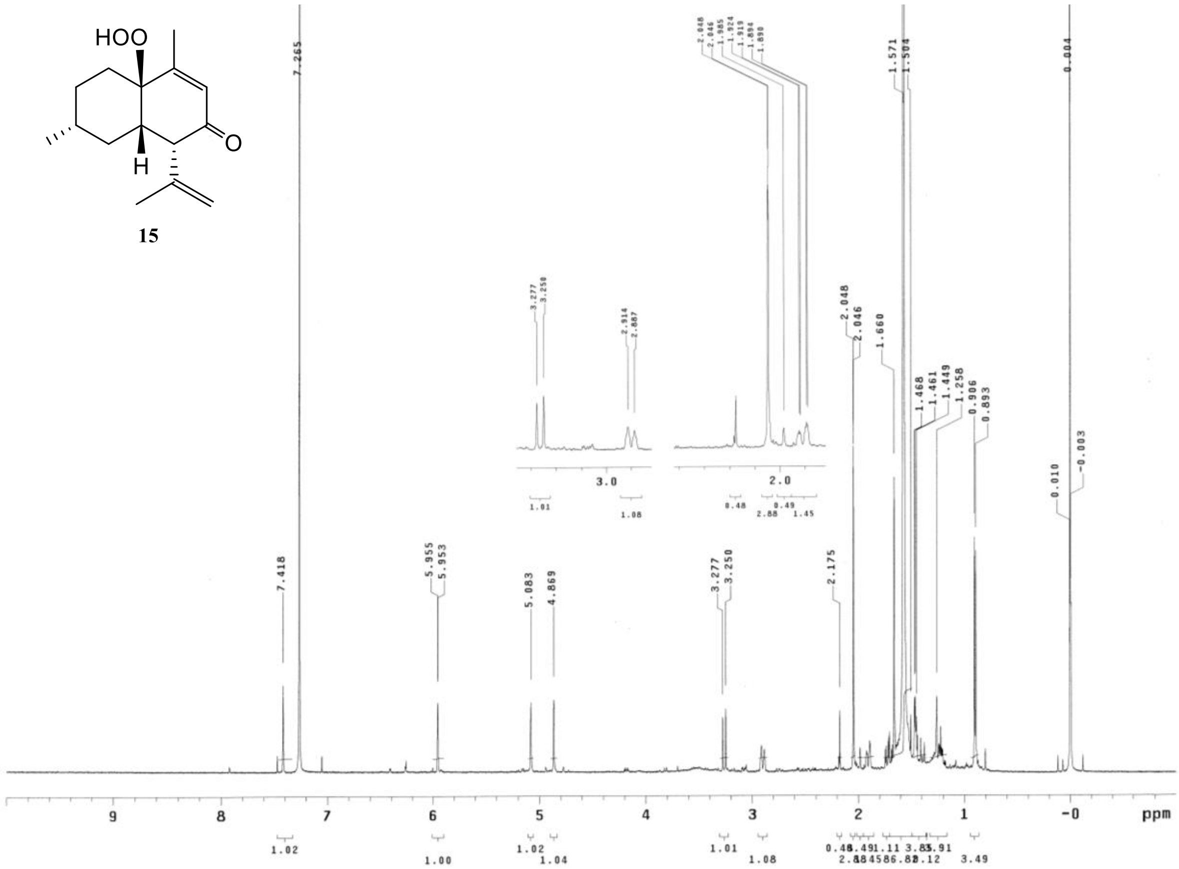

Figure S100. ^{1}H NMR spectrum of **15** in $CDCl_3$ at 500 MHz

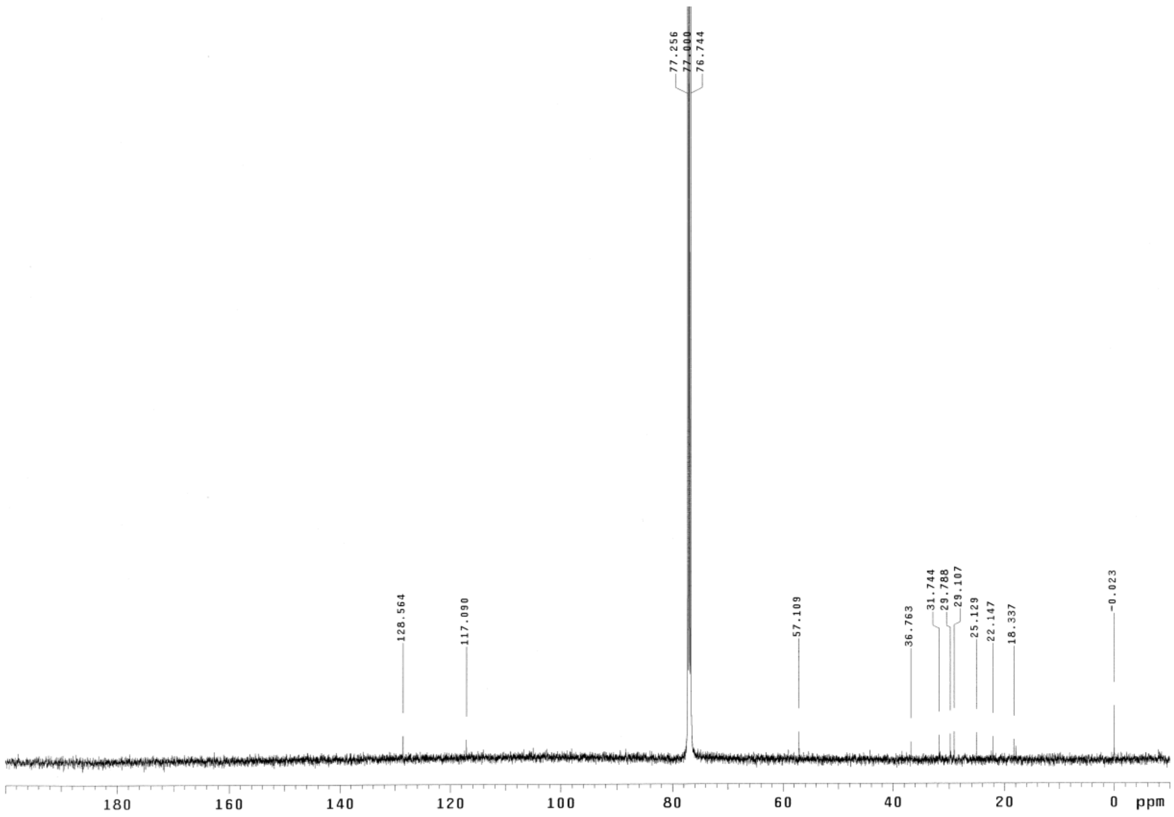

Figure S101. ^{13}C NMR spectrum of **15** in $CDCl_3$ at 125 MHz

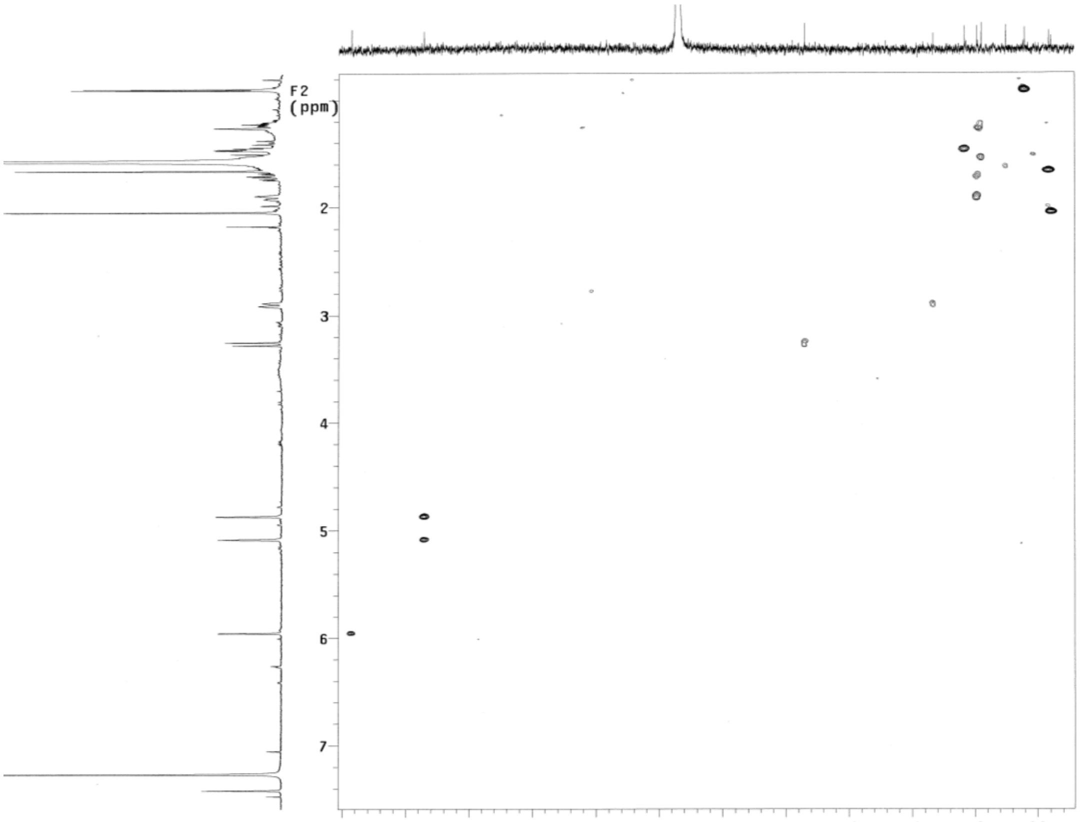

Figure S102. HSQC spectrum of **15** in $CDCl_3$

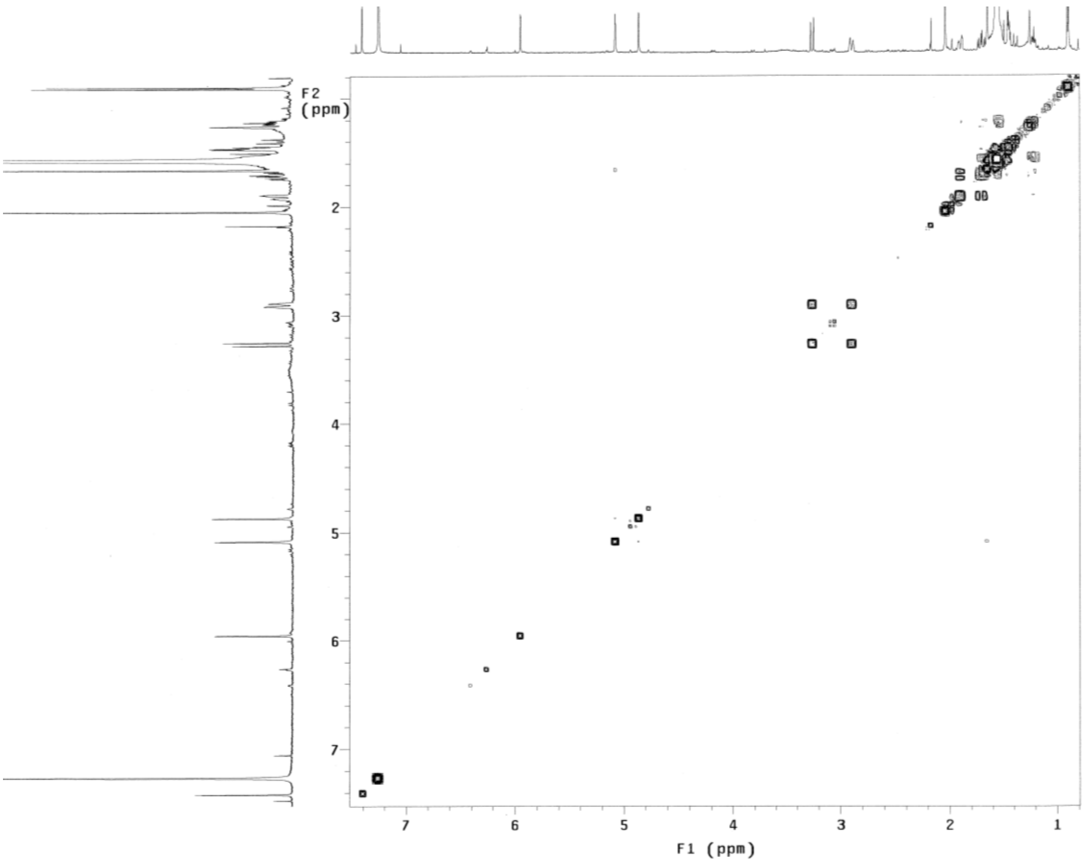

Figure S103. COSY spectrum of **15** in $CDCl_3$

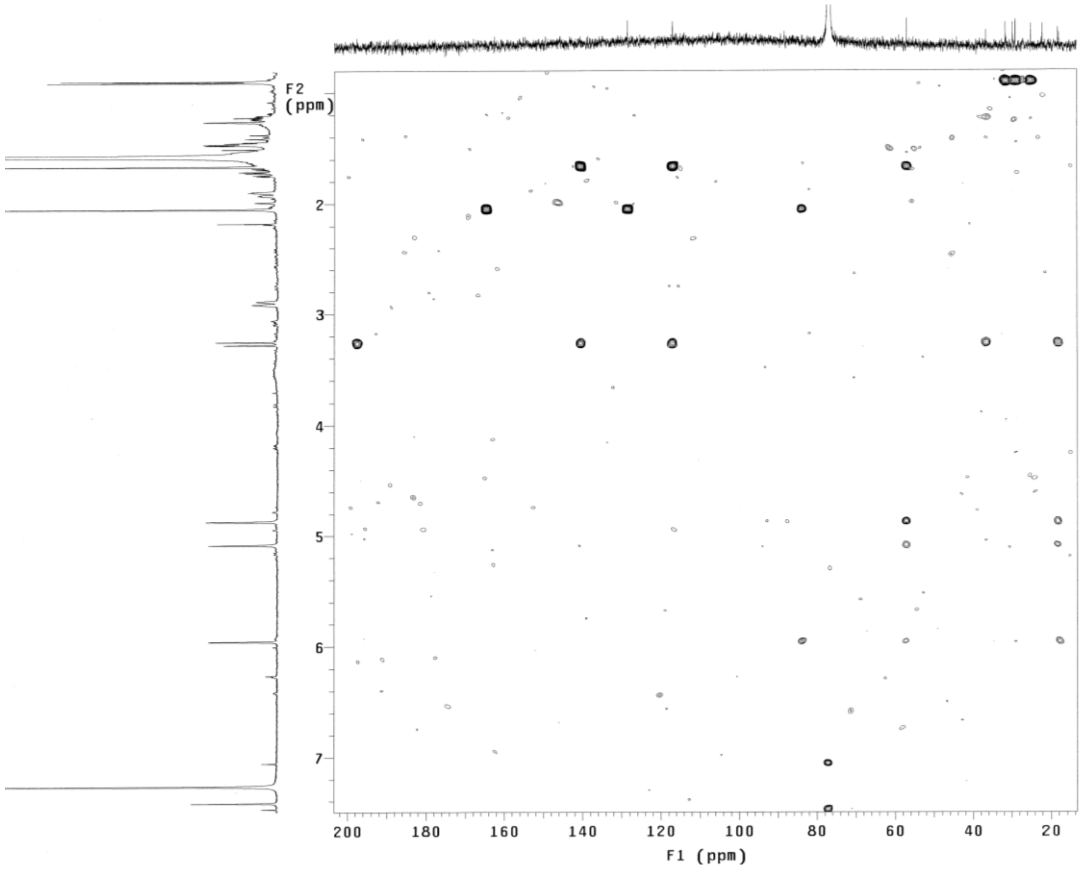

Figure S104. HMBC spectrum of **15** in $CDCl_3$

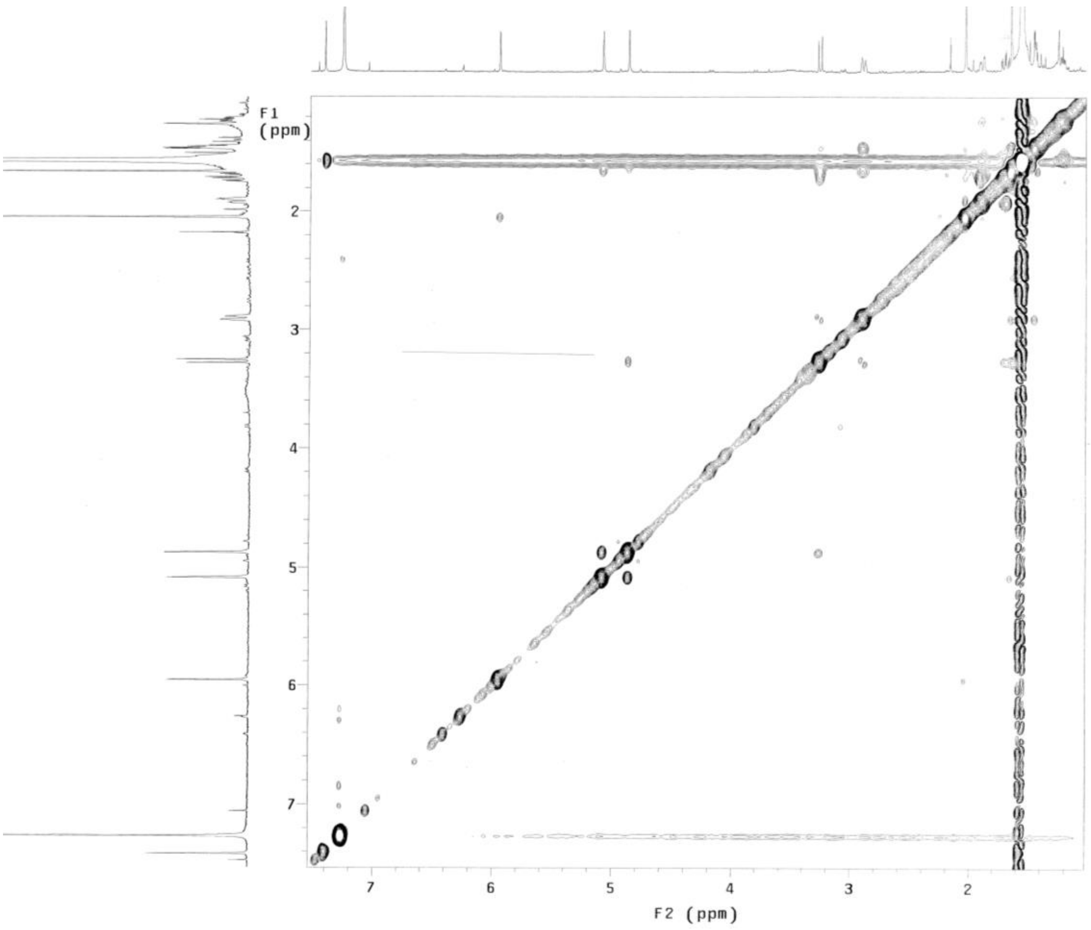

Figure S105. NOESY spectrum of **15** in $CDCl_3$

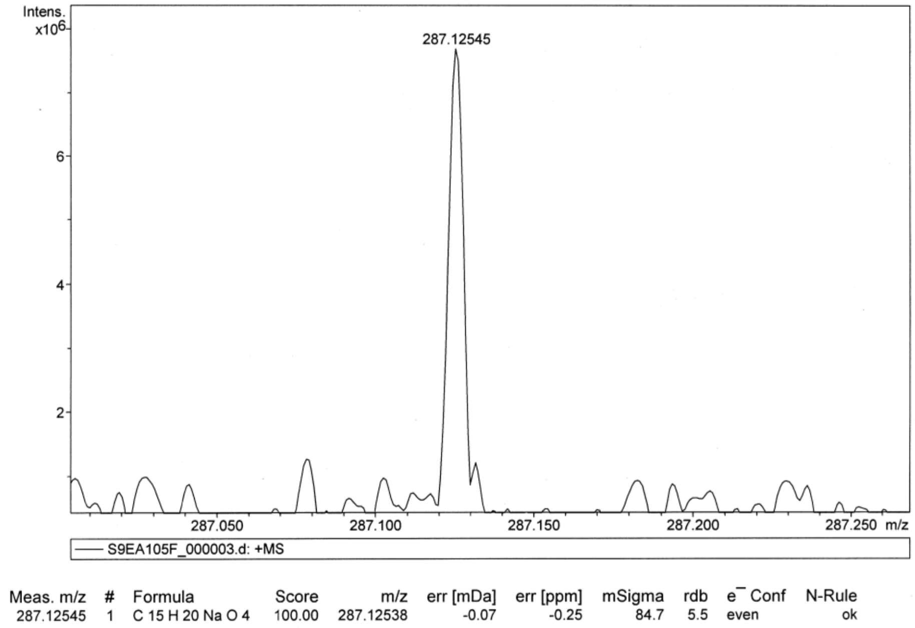

Figure S106. HRESIMS spectrum of **16**

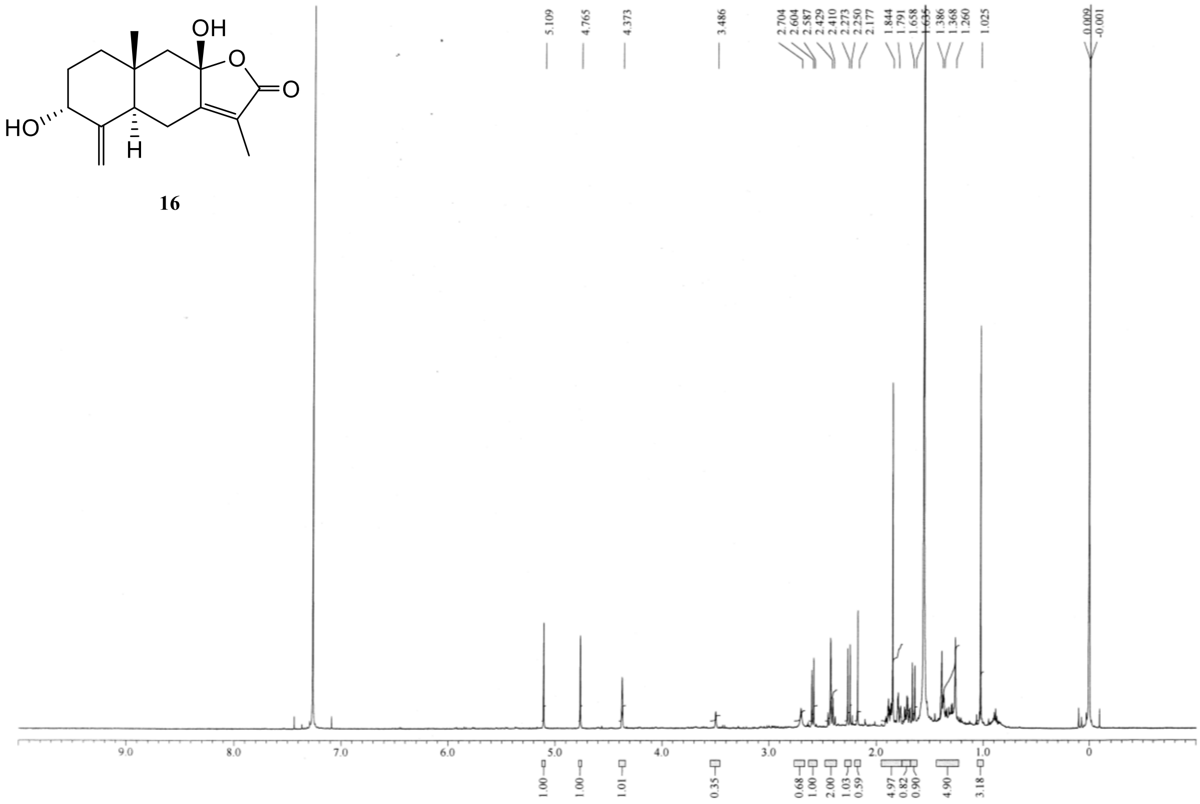

Figure S107. ^{1}H NMR spectrum of **16** in $CDCl_3$ at 600 MHz

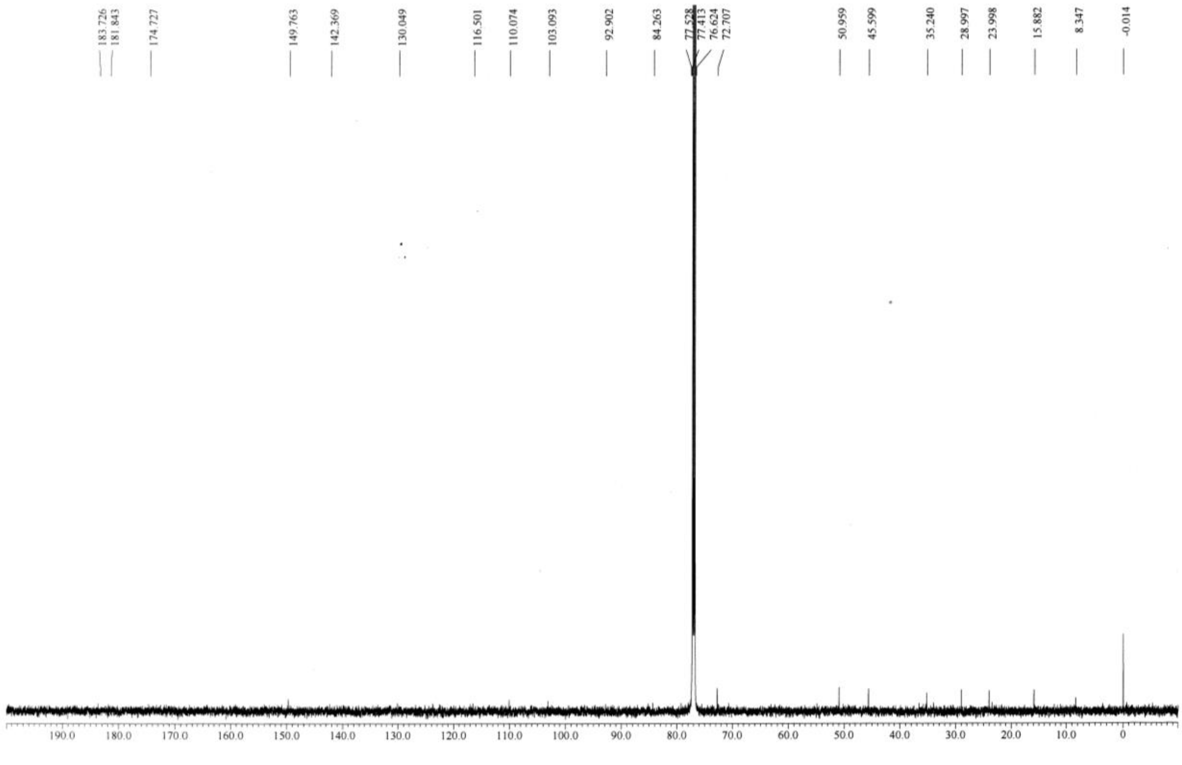

Figure S108. ^{13}C NMR spectrum of **16** in $CDCl_3$ at 150 MHz

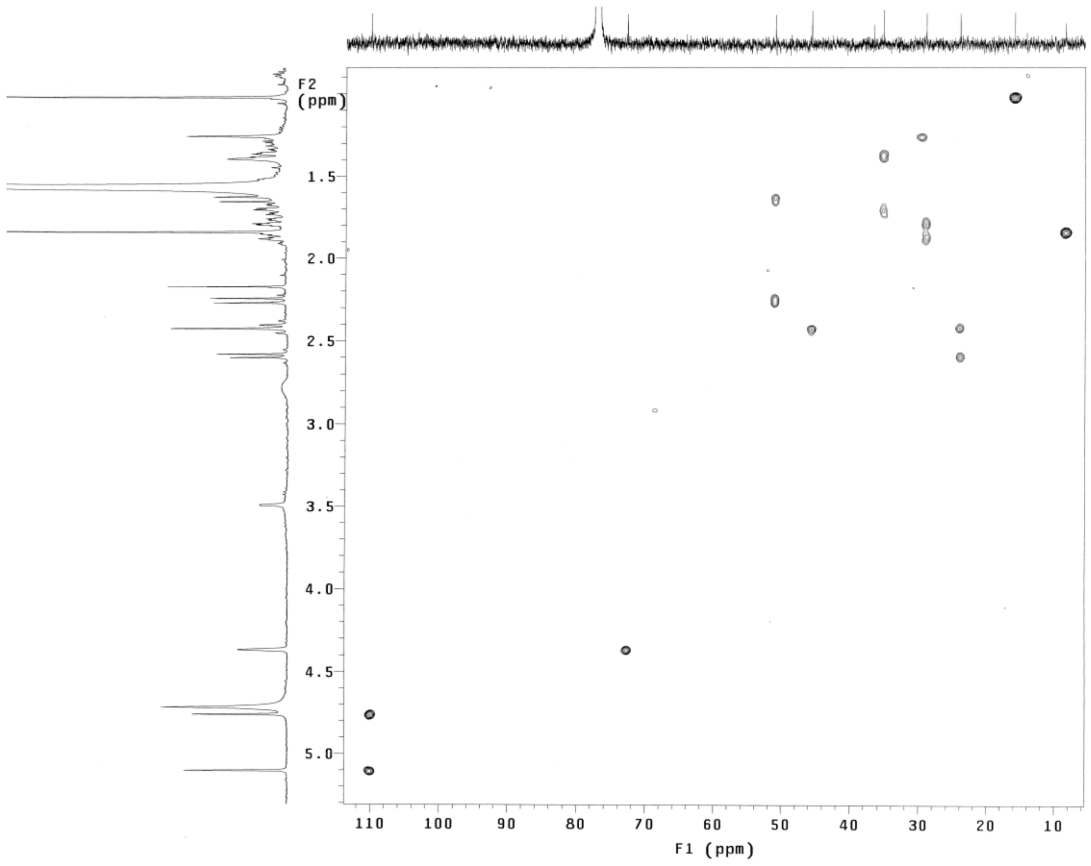

Figure S109. HSQC spectrum of **16** in $CDCl_3$

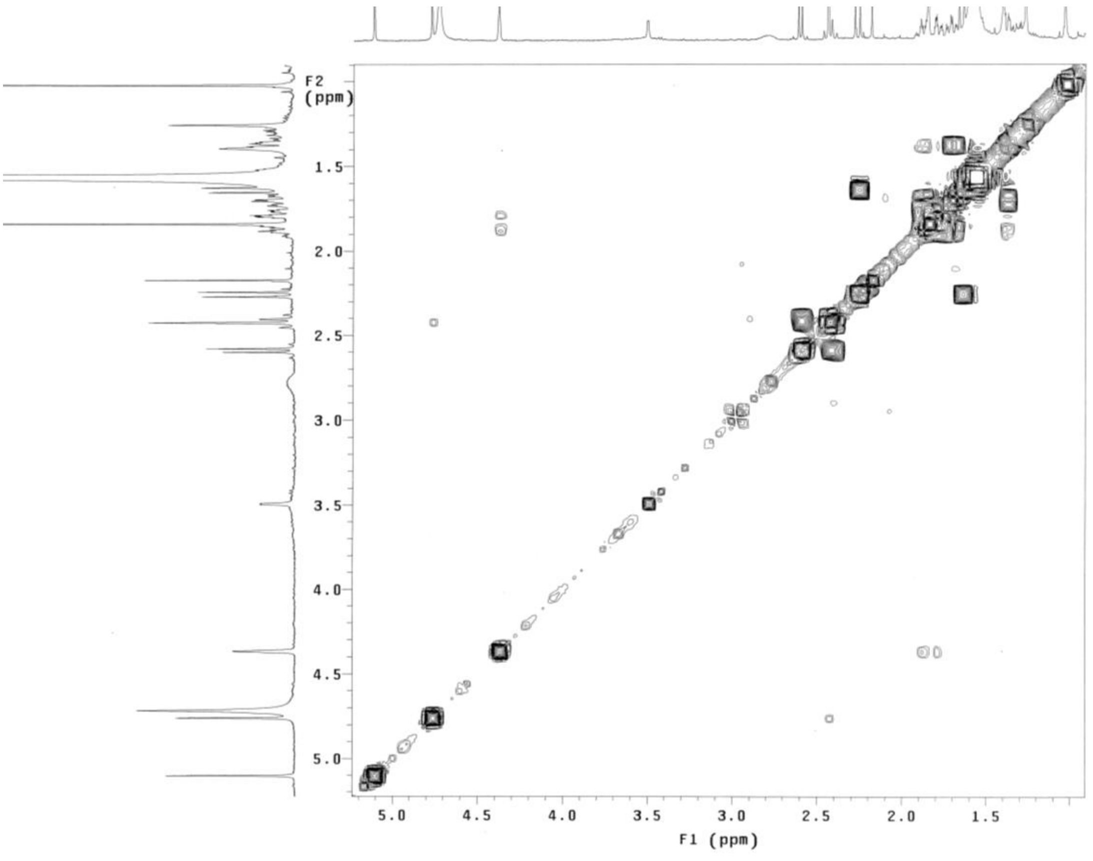

Figure S110. COSY spectrum of **16** in $CDCl_3$

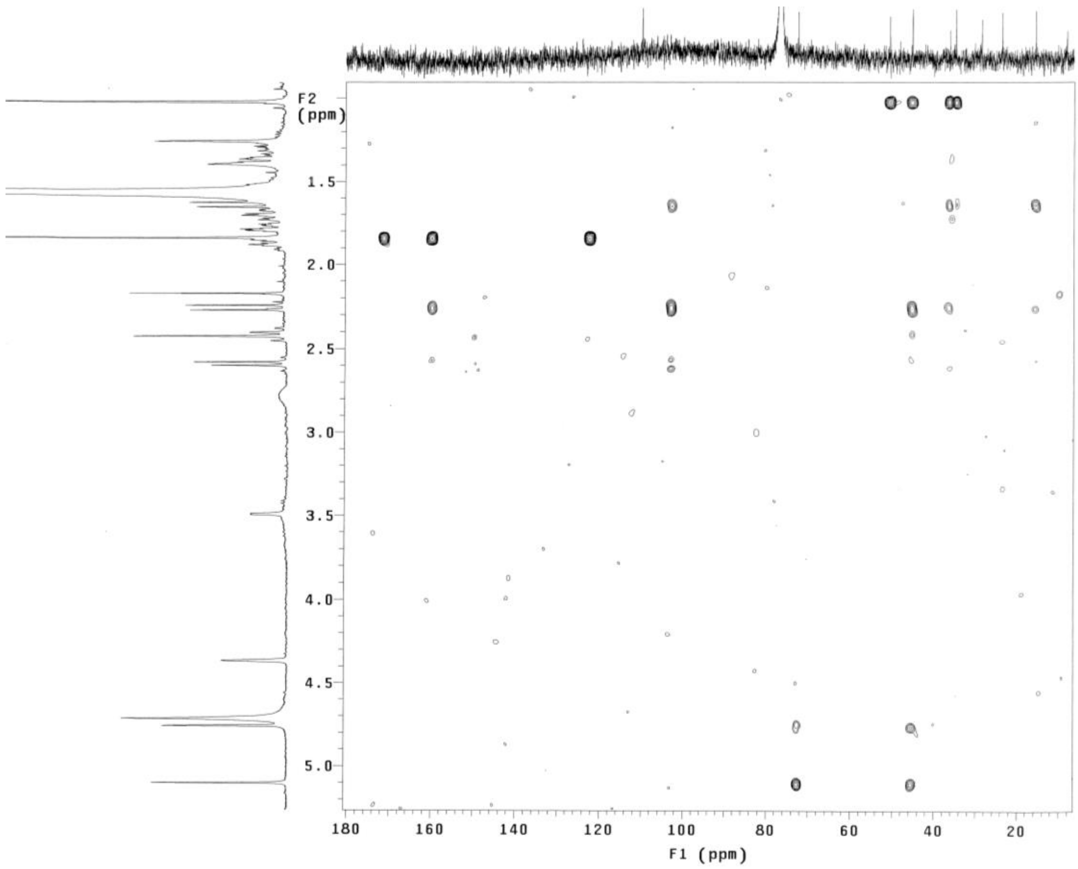

Figure S111. HMBC spectrum of **16** in $CDCl_3$

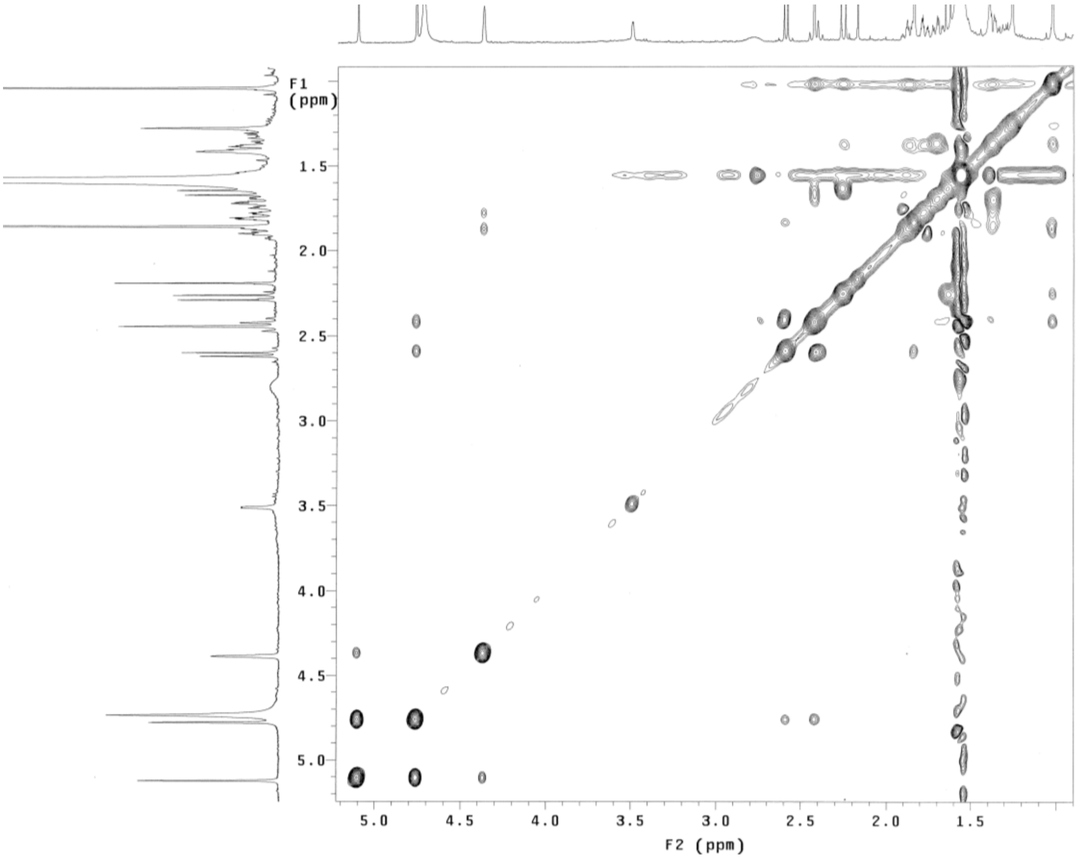

Figure S112. NOESY spectrum of **16** in $CDCl_3$

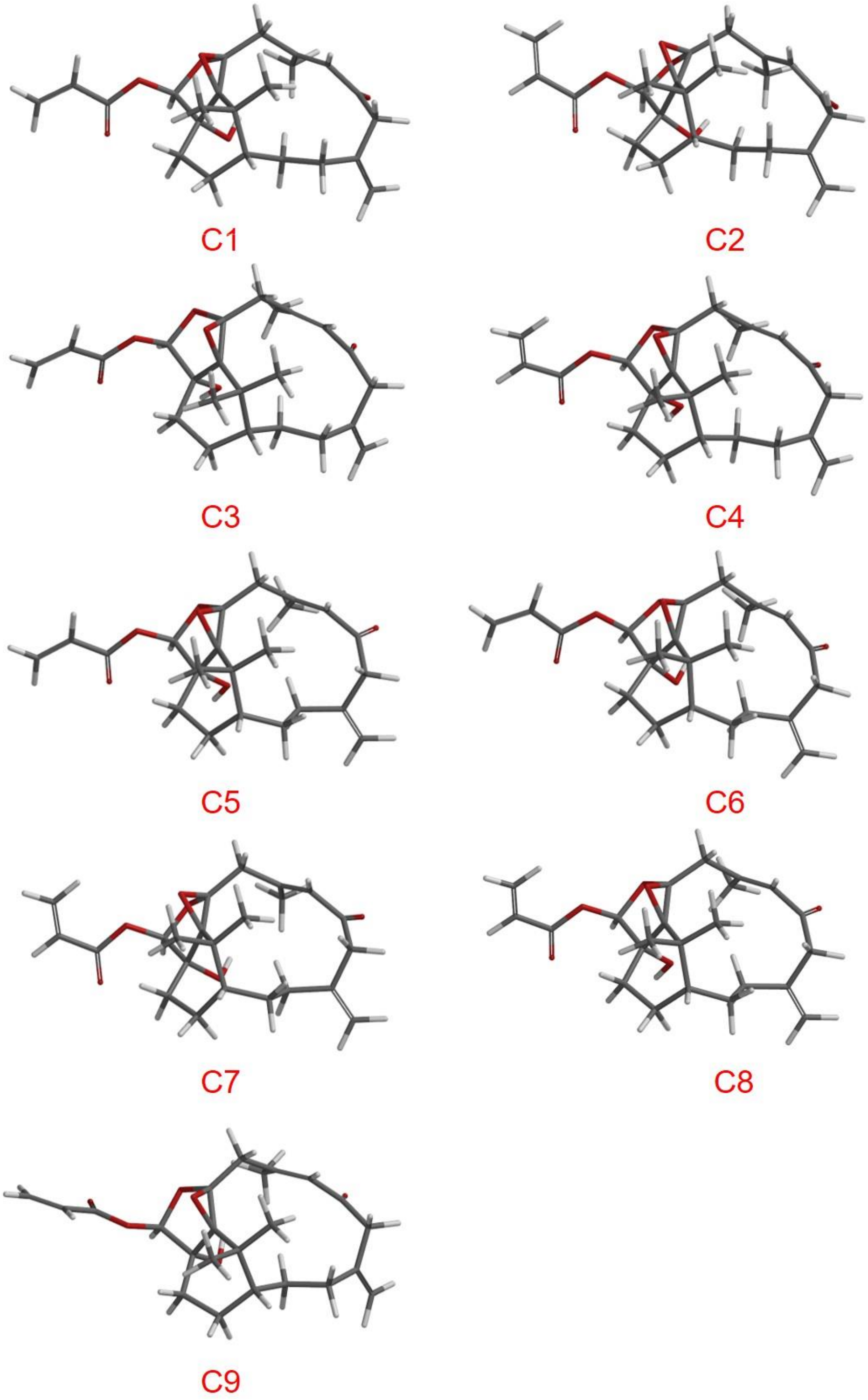

Figure S113. MMFF lowest energy conformers for **1**.

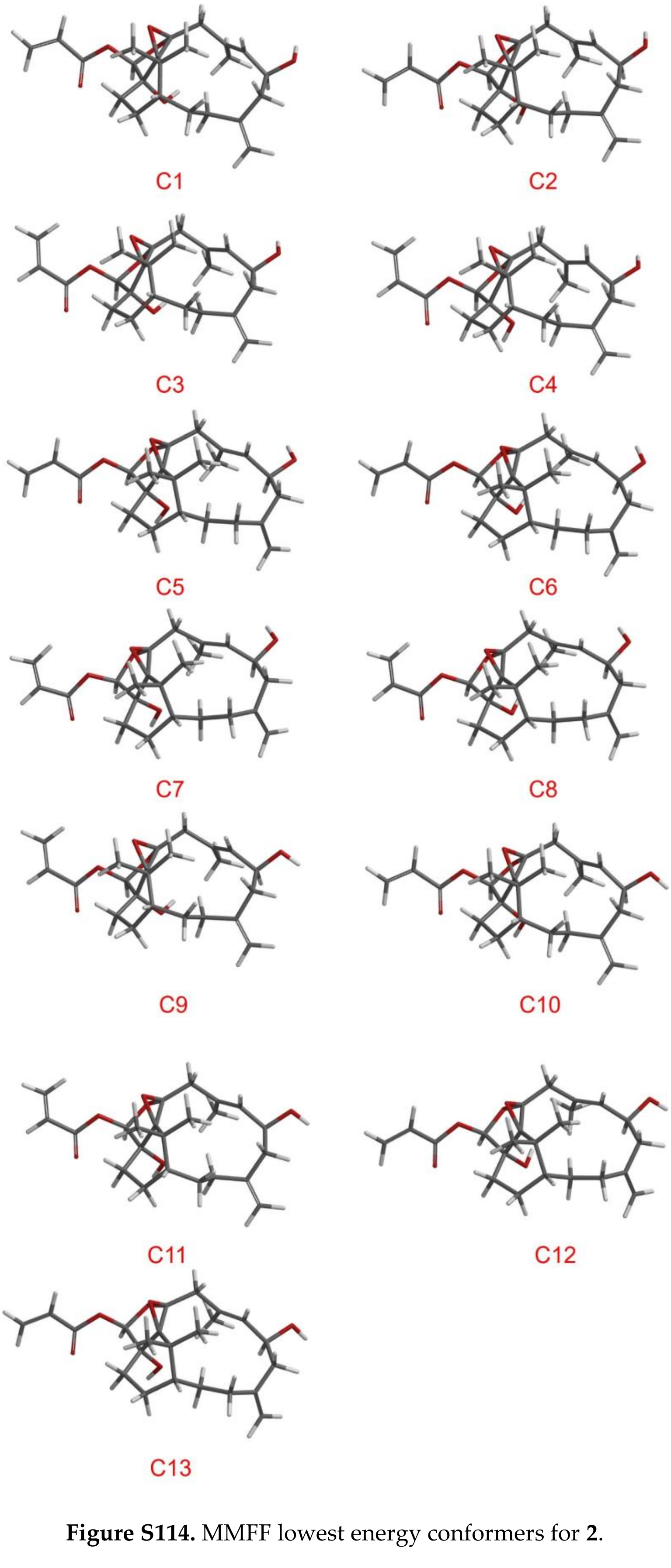

Figure S114. MMFF lowest energy conformers for **2**.

Table S1. ^1H and ^{13}C NMR spectroscopic data of compounds **17**and **18**.

No.	17		18	
	^1H [1]	^{13}C [2]	^1H [3]	^{13}C [4]
1	1.91, m	43.0 (CH) [6]	1.63, m	43.0 (CH) [6]
2	1.66, m; 1.57, m	29.0 (CH_2)	1.95, m; 1.52, m	30.6 (CH_2)
3	1.99, m; 1.84 m	33.5 (CH_2)	2.69, t (12.8) [5]<br>1.91, m	30.6 (CH_2)
4		146.2 (C)		147.0 (C)
5	2.49, dd (13.5, 2.5) [5]<br>2.29, dd (13.5, 9.0)	44.3 (CH_2)	2.46, dd (12.8, 2.4)<br>2.30, dd (12.8, 7.6)	44.7 (CH_2)
6	4.48, t (9.0)	68.7 (CH)	4.44, d (6.0)	69.2 (CH)
7	5.40, d (9.0)	134.4 (CH)	5.13, d (7.6)	131.1 (CH)
8		130.5 (C)		130.8 (C)
9	3.23, d (13.5)<br>2.86, d (13.5)	46.3 (CH_2)	3.43, d (15.6)<br>3.04, d (15.6)	51.0 (CH_2)
10		169.7 (C)		202.2 (C)
11		211.1 (C)		147.9 (C)
12	5.08, dd (6.0, 4.0)	72.2 (CH)	6.21, br s	135.5 (CH)
13	2.40, dt (22.0, 7.0)<br>1.89, m	26.4 (CH_2)	2.29, 2H, m	23.9 (CH_2)
14	1.97, m; 1.65, m	20.4 (CH_2)	2.24, m; 1.66, m	22.9 (CH_2)
15		47.6 (C)		35.3 (C)
16	1.23, 3H, s	27.8 (CH_3)	1.28, 3H, s	32.8 (CH_3)
17	1.17, 3H, s	23.1 (CH_3)	1.15, 3H, s	24.5 (CH_3)
18	4.87, 4.85, both s	112.2 (CH_2)	4.84, 4.81, both s	112.6 (CH_2)
19	1.74, 3H, s	16.9 (CH_3)	1.75, 3H, s	18.9 (CH_3)

[1] Spectrum recorded at 500 MHz in $CDCl_3$.
[2] Spectrum recorded at 125 MHz in $CDCl_3$.
[3] Spectrum recorded at 400 MHz in $CDCl_3$.
[4] Spectrum recorded at 100 MHz in $CDCl_3$.
[5] *J* values are in Hz.
[6] Attached protons were deduced by HSQC experiments

Table S2. Energy analysis for MMFF conformational searching of compounds **1** and **2**

Conf.	1		2	
	rel. E (kcal/mol)	Boltzmann Dist.	rel. E (kcal/mol)	Boltzmann Dist.
C1	0.00	0.223	0.00	0.261
C2	0.31	0.133	0.30	0.156
C3	0.33	0.127	0.96	0.052
C4	0.71	0.067	1.16	0.037
C5	1.08	0.036	1.21	0.034
C6	1.11	0.034	1.48	0.021
C7	1.40	0.021	1.59	0.018
C8	1.45	0.019	1.80	0.013
C9	1.83	0.010	2.07	0.008
C10			2.18	0.007
C11			2.56	0.003
C12			2.81	0.002
C13			2.99	0.002
